# Supplementary material for: The Arp2/3 complex controls the development of homeostatic microglia
Source: EMBO Rep. 2026 Feb 27;27(7):1696–719. doi: 10.1038/s44319-026-00721-8 (PMC13076794; doi:10.1038/s44319-026-00721-8)
Supplement: Supplementary file 2 — Dataset EV1 [file 44319_2026_721_MOESM2_ESM.pdf]

Figure	Cohen's *d*	*P* value
1B, Ramification	Cortex: 2.195603535<br>CC: 3.178566571	0.0046<br>0.0049
1B, Cell territory	Cortex: 3.113389387<br>CC: 3.235261776	0.0237<br>0.0078
1D	3wks: -5.178598483<br>12wks: -7.497742529	0.0006<br>0.00001
2C	13.67697292	9.69×10^{-4}
2D	10.1631322	0.01891
3B	27.2957285	0.00001
4C	-10.319203	0.025
5H	-5.5773576	0.0017
6D	pSMAD2: -3.9226957<br>pSMAD3: -6.8645225	0.0059<br>0.0002
EV1C	3wks: -0.734386001<br>12wks: -0.293545381	>0.9999<br>>0.9999
EV1E, Ramifivation	Cortex: 1.004740624<br>CC: 0.532511366	>0.9999<br>>0.9999
EV1E, Cell territory	Cortex: 1.149157934<br>CC: -0.535153072	>0.9999<br>>0.9999
EV3B, Ramification	Ctrl: F vs M, Cortex: -0.522573882<br>Ctrl: F vs M, CC: -0.748829235	>0.9999<br>>0.9999
EV3B, Ramification	KO: F vs M, Cortex: -0.140152368<br>KO: F vs M, CC: 1.161829638	>0.9999<br>>0.9999
EV3B, Cell territory	Ctrl: F vs M, Cortex: -0.2031523<br>Ctrl: F vs M, CC: -1.3922604	>0.9999<br>>0.9999
EV3B, Cell territory	KO: F vs M, Cortex: 0.49971122<br>KO: F vs M, CC: 0.5149625	>0.9999<br>>0.9999
