# Supplementary figures and images for: The Arp2/3 complex controls the development of homeostatic microglia (part 1 of 2)
Source: EMBO Rep. 2026 Feb 27;27(7):1696–719. doi: 10.1038/s44319-026-00721-8 (PMC13076794; doi:10.1038/s44319-026-00721-8)

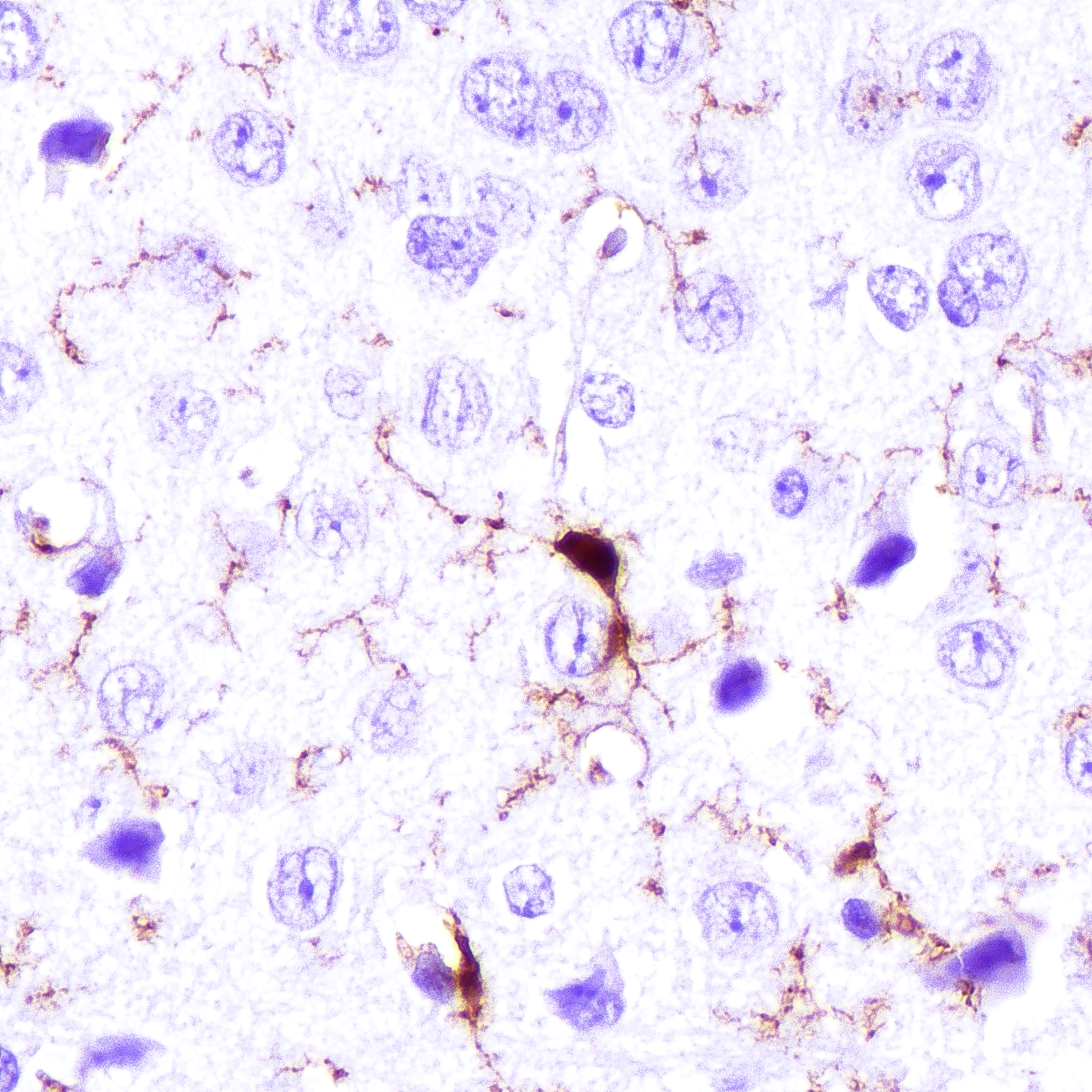

Supplement: Supplementary file 6 — Source data Fig. 1 [file 44319_2026_721_MOESM6_ESM.zip › 1C/DAB images-Different brain regions/Controls/Ctx_LayerV-VI.tif]

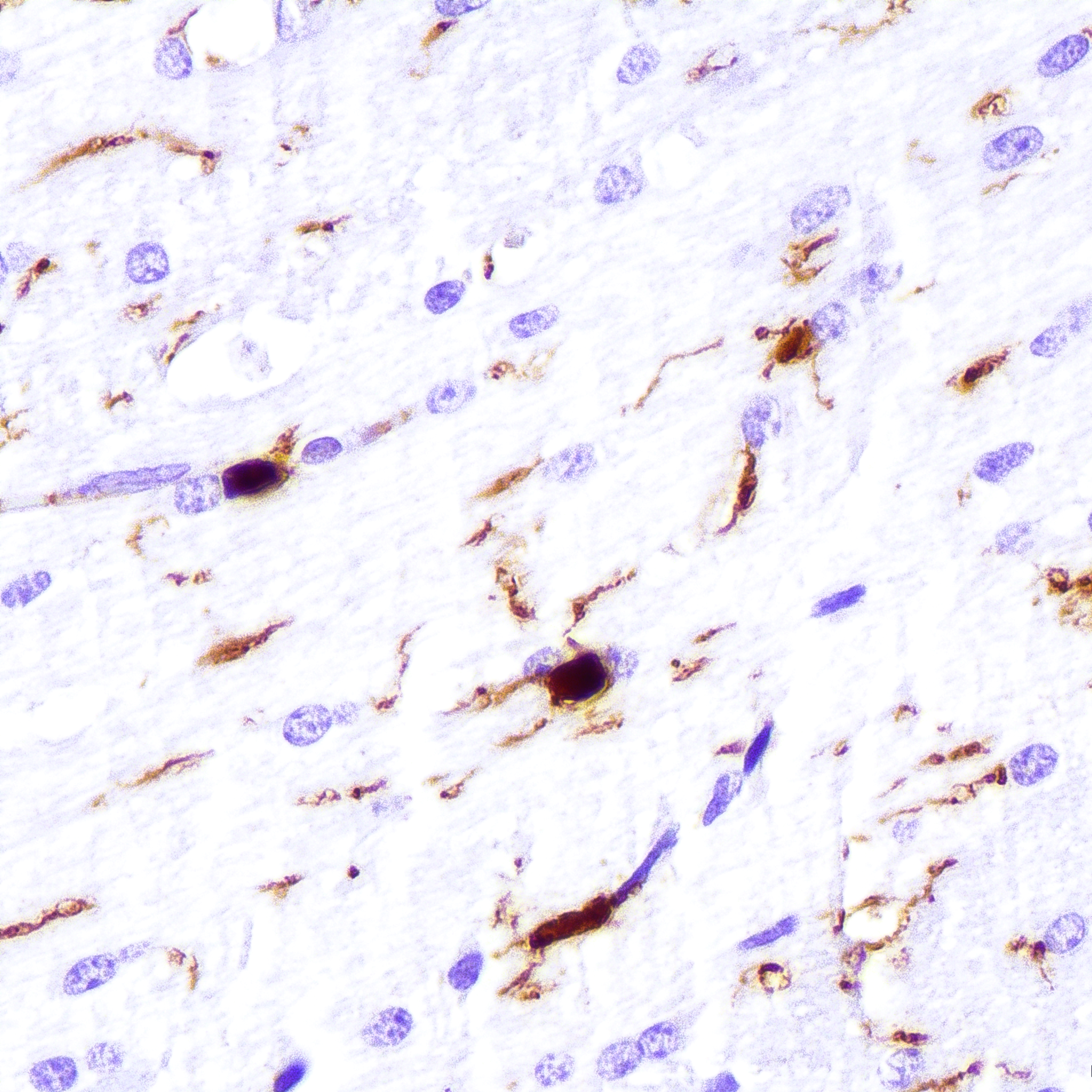

Supplement: Supplementary file 6 — Source data Fig. 1 [file 44319_2026_721_MOESM6_ESM.zip › 1C/DAB images-Different brain regions/Controls/CC.tif]

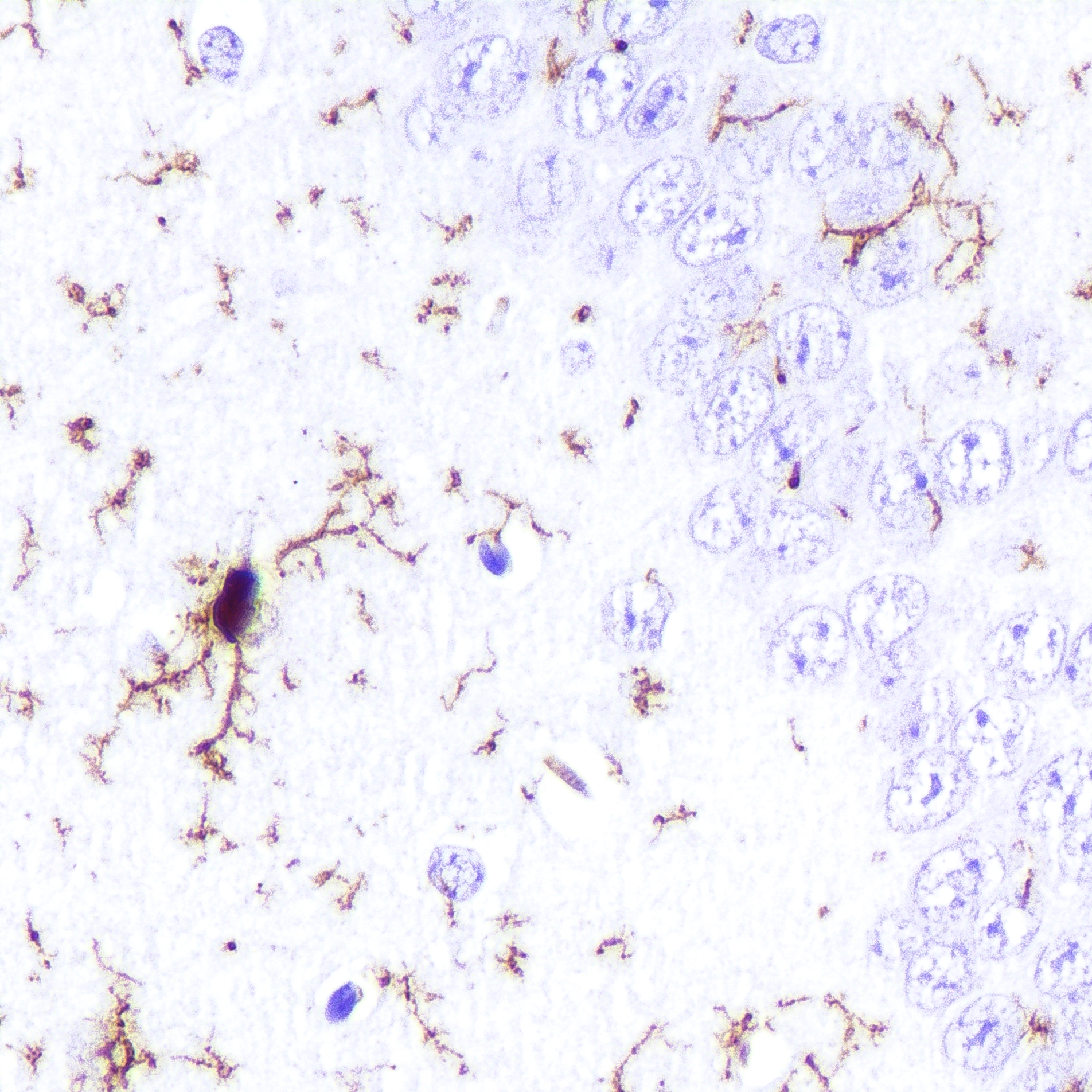

Supplement: Supplementary file 6 — Source data Fig. 1 [file 44319_2026_721_MOESM6_ESM.zip › 1C/DAB images-Different brain regions/Controls/hippocampus.tif]

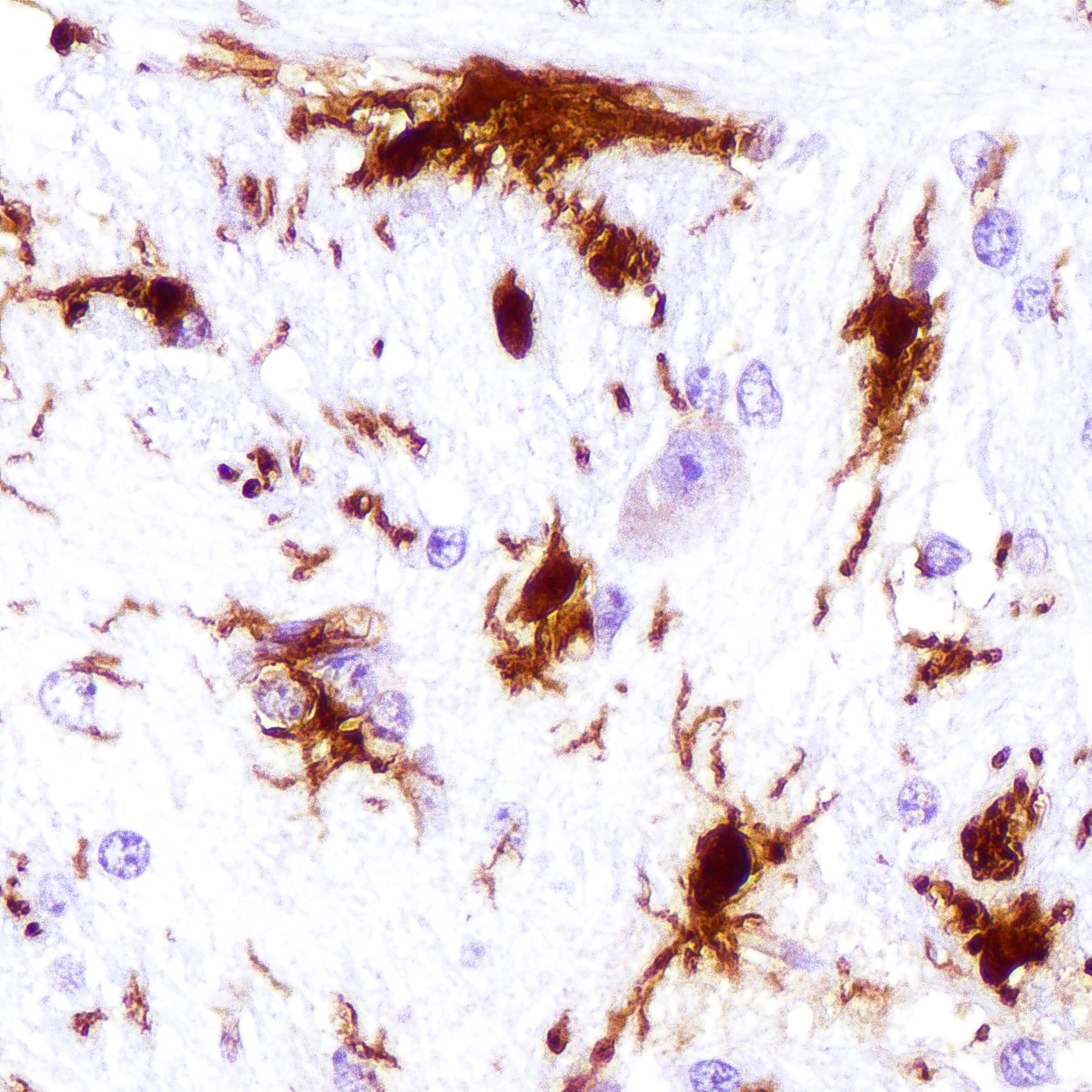

Supplement: Supplementary file 6 — Source data Fig. 1 [file 44319_2026_721_MOESM6_ESM.zip › 1C/DAB images-Different brain regions/Knockout/striatum-2.tif]

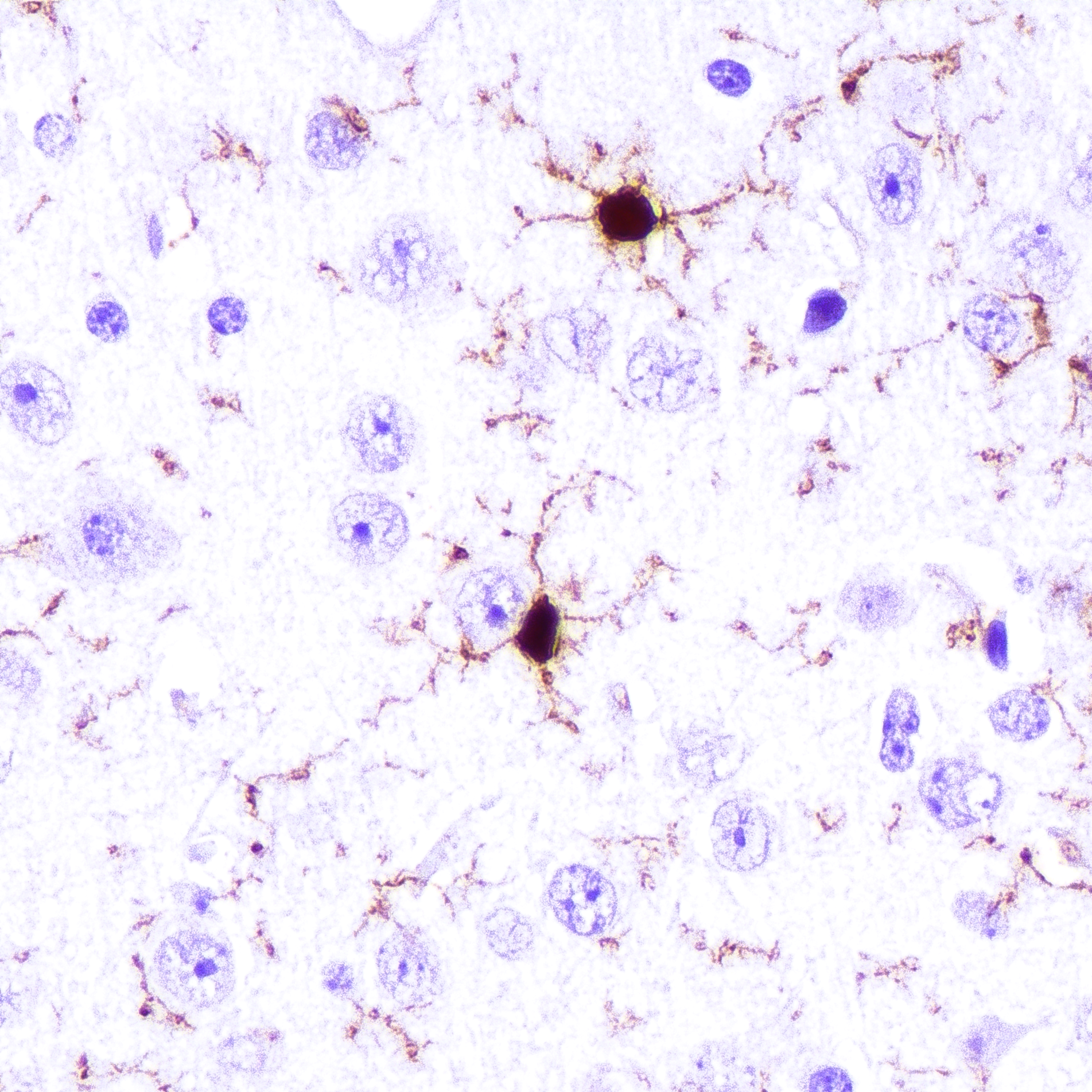

Supplement: Supplementary file 6 — Source data Fig. 1 [file 44319_2026_721_MOESM6_ESM.zip › 1C/DAB images-Different brain regions/Controls/Ctx_LayerII-IV.tif]

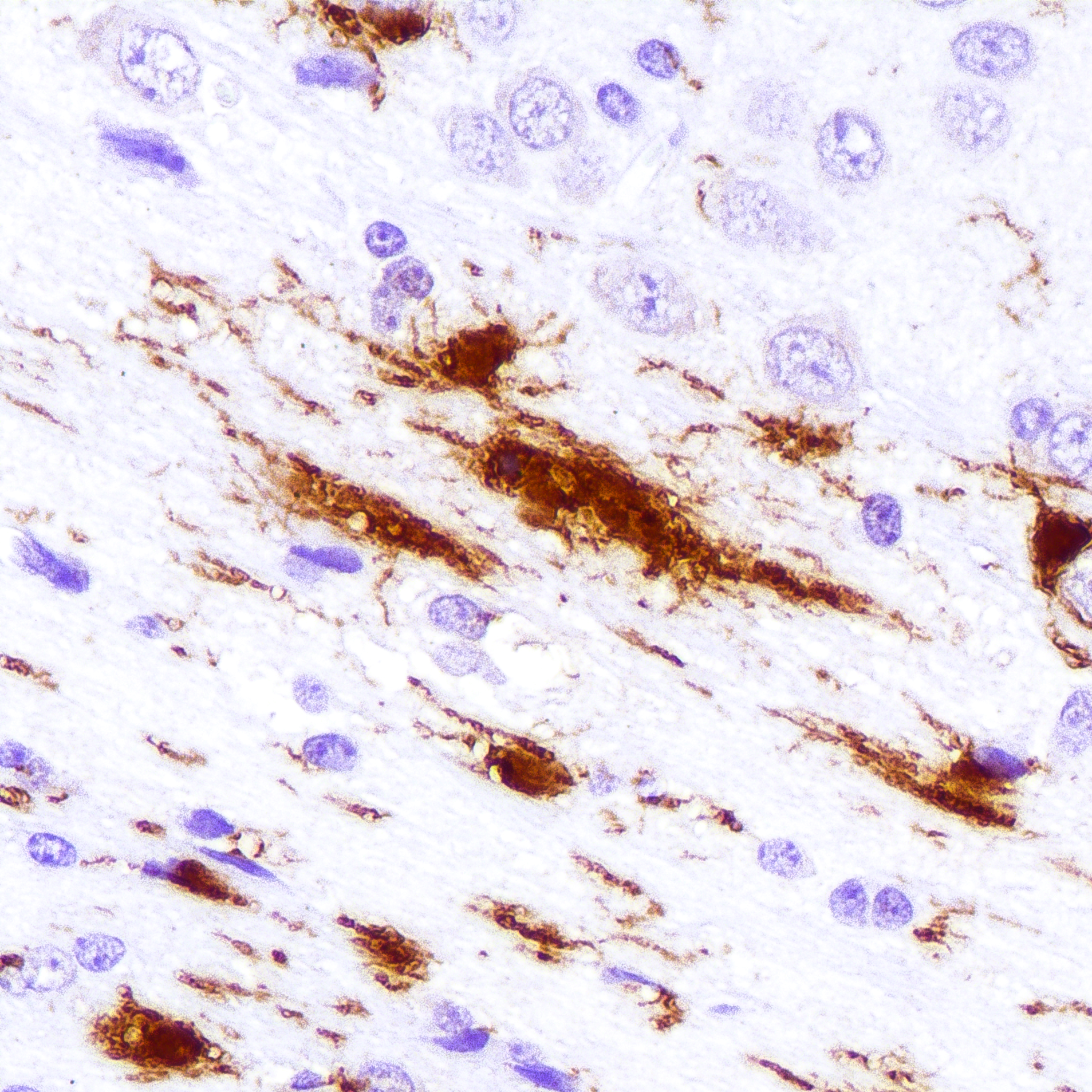

Supplement: Supplementary file 6 — Source data Fig. 1 [file 44319_2026_721_MOESM6_ESM.zip › 1C/DAB images-Different brain regions/Knockout/CC-2.tif]

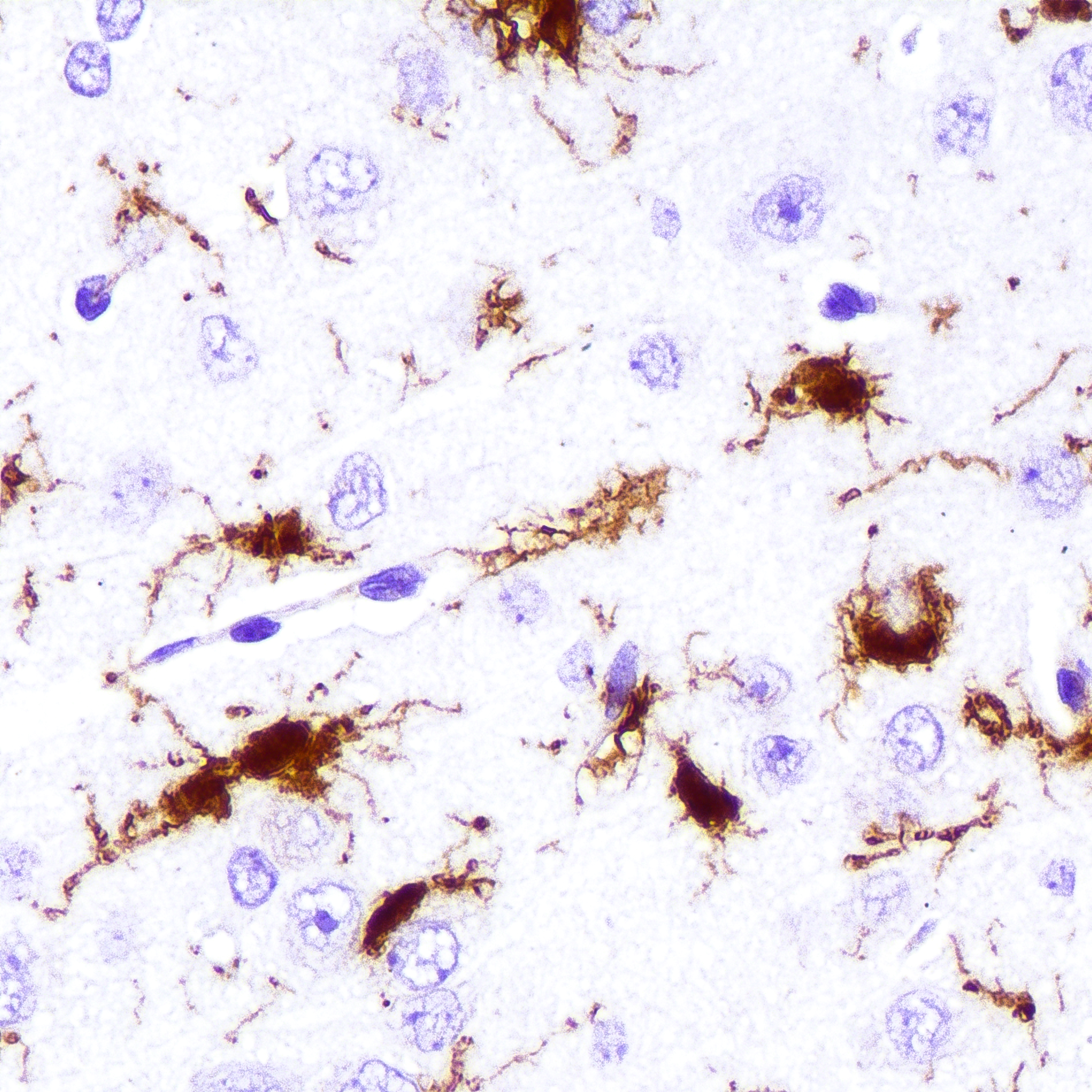

Supplement: Supplementary file 6 — Source data Fig. 1 [file 44319_2026_721_MOESM6_ESM.zip › 1C/DAB images-Different brain regions/Knockout/Ctx_LayerII-IV-2.tif]

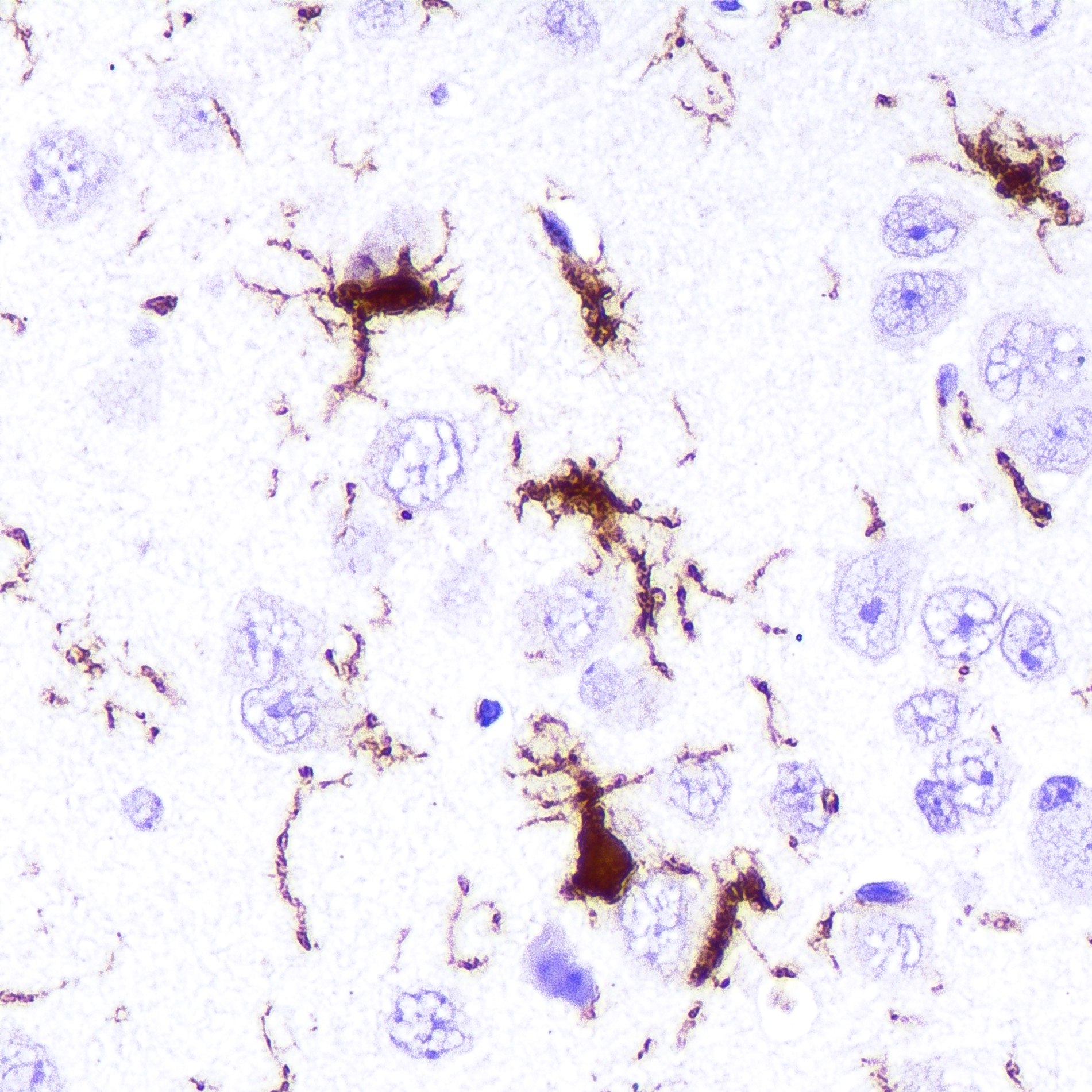

Supplement: Supplementary file 6 — Source data Fig. 1 [file 44319_2026_721_MOESM6_ESM.zip › 1C/DAB images-Different brain regions/Knockout/Ctx_LayerV-VI-2.tif]

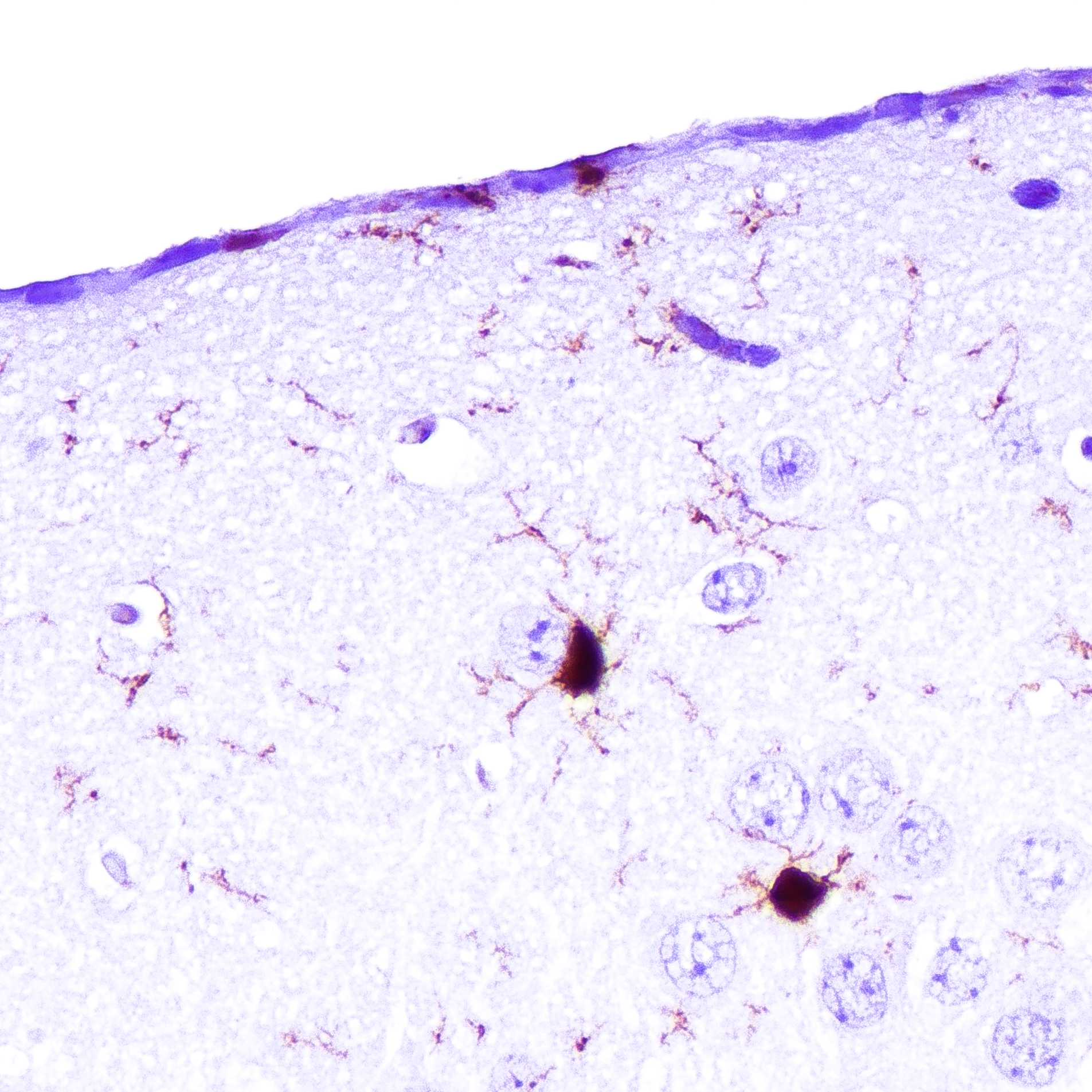

Supplement: Supplementary file 6 — Source data Fig. 1 [file 44319_2026_721_MOESM6_ESM.zip › 1C/DAB images-Different brain regions/Controls/Ctx_LayerI.tif]

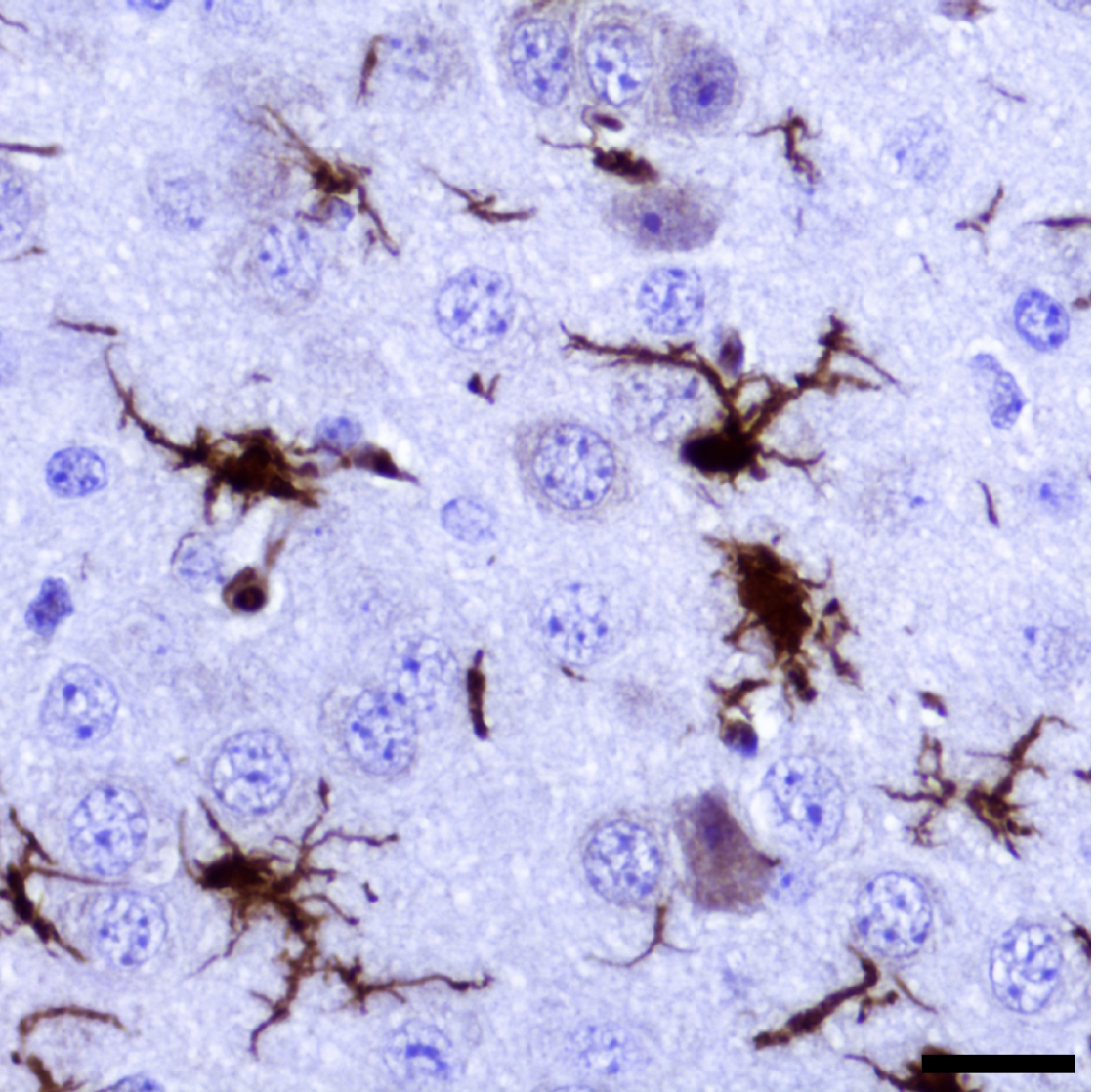

Supplement: Supplementary file 6 — Source data Fig. 1 [file 44319_2026_721_MOESM6_ESM.zip › 1A/DAB images-Microglia/Arpc4-KO/Screenshot 2025-09-01 at 13.46.53.tif]

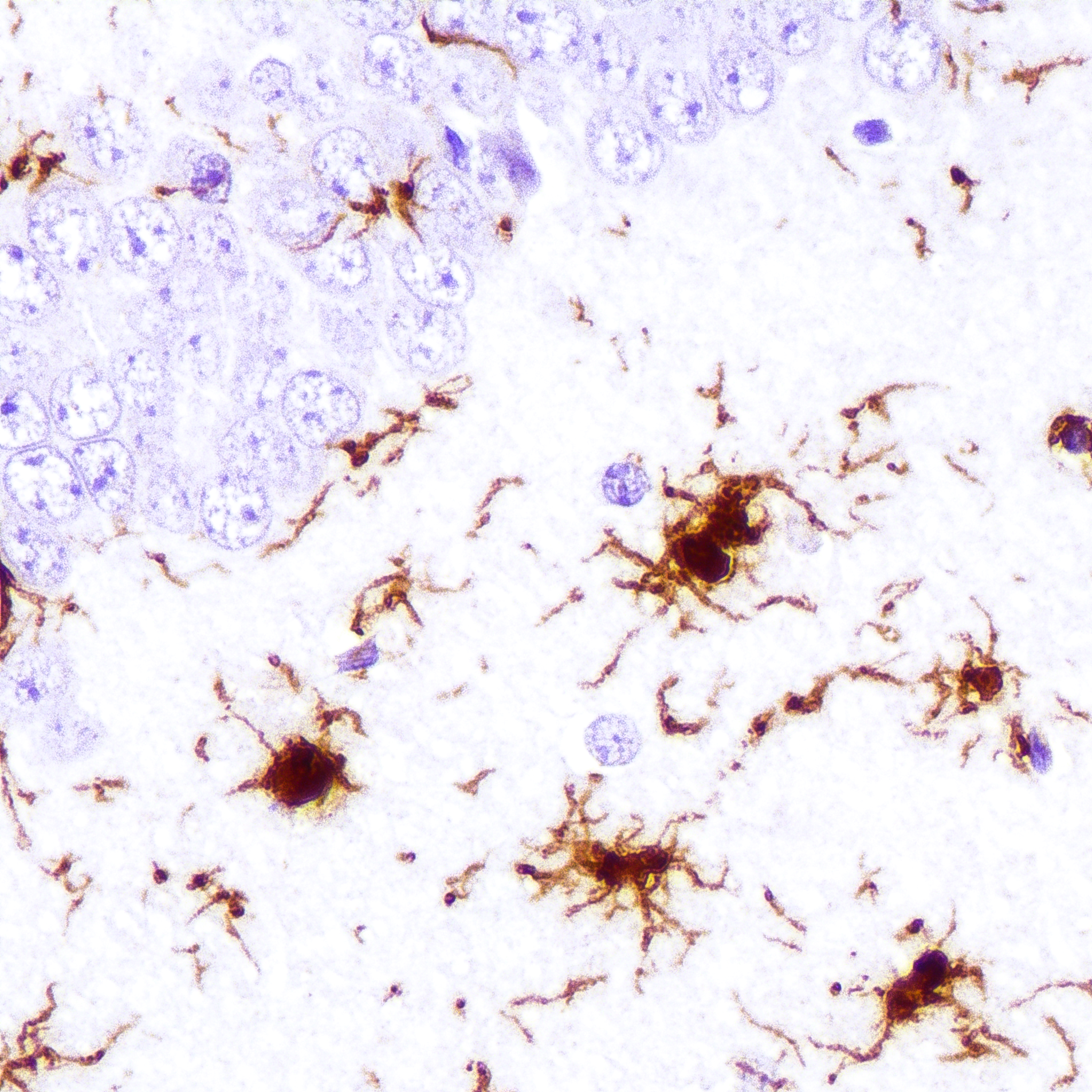

Supplement: Supplementary file 6 — Source data Fig. 1 [file 44319_2026_721_MOESM6_ESM.zip › 1C/DAB images-Different brain regions/Knockout/hippocampus-2.tif]

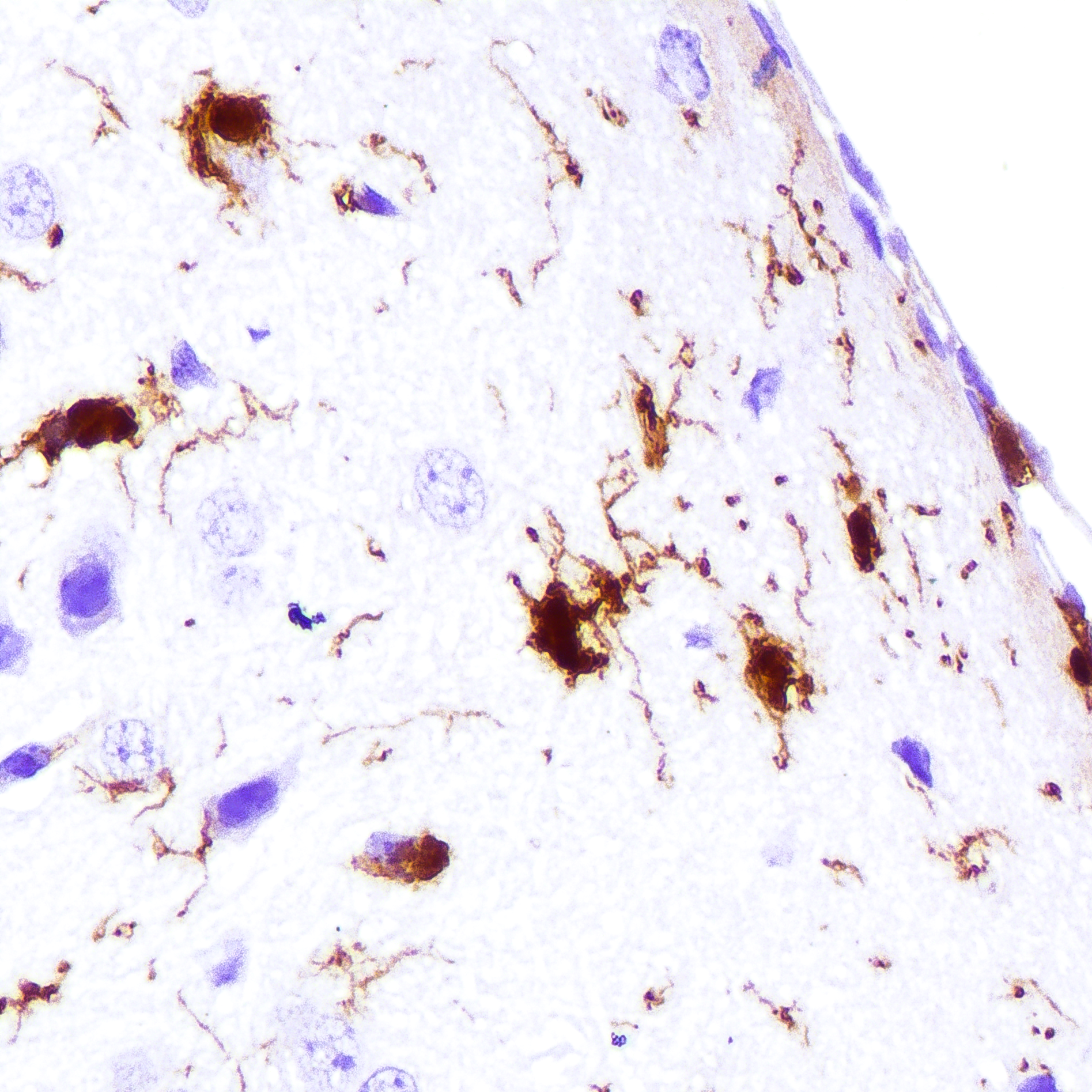

Supplement: Supplementary file 6 — Source data Fig. 1 [file 44319_2026_721_MOESM6_ESM.zip › 1C/DAB images-Different brain regions/Knockout/Ctx_LayerI-2.tif]

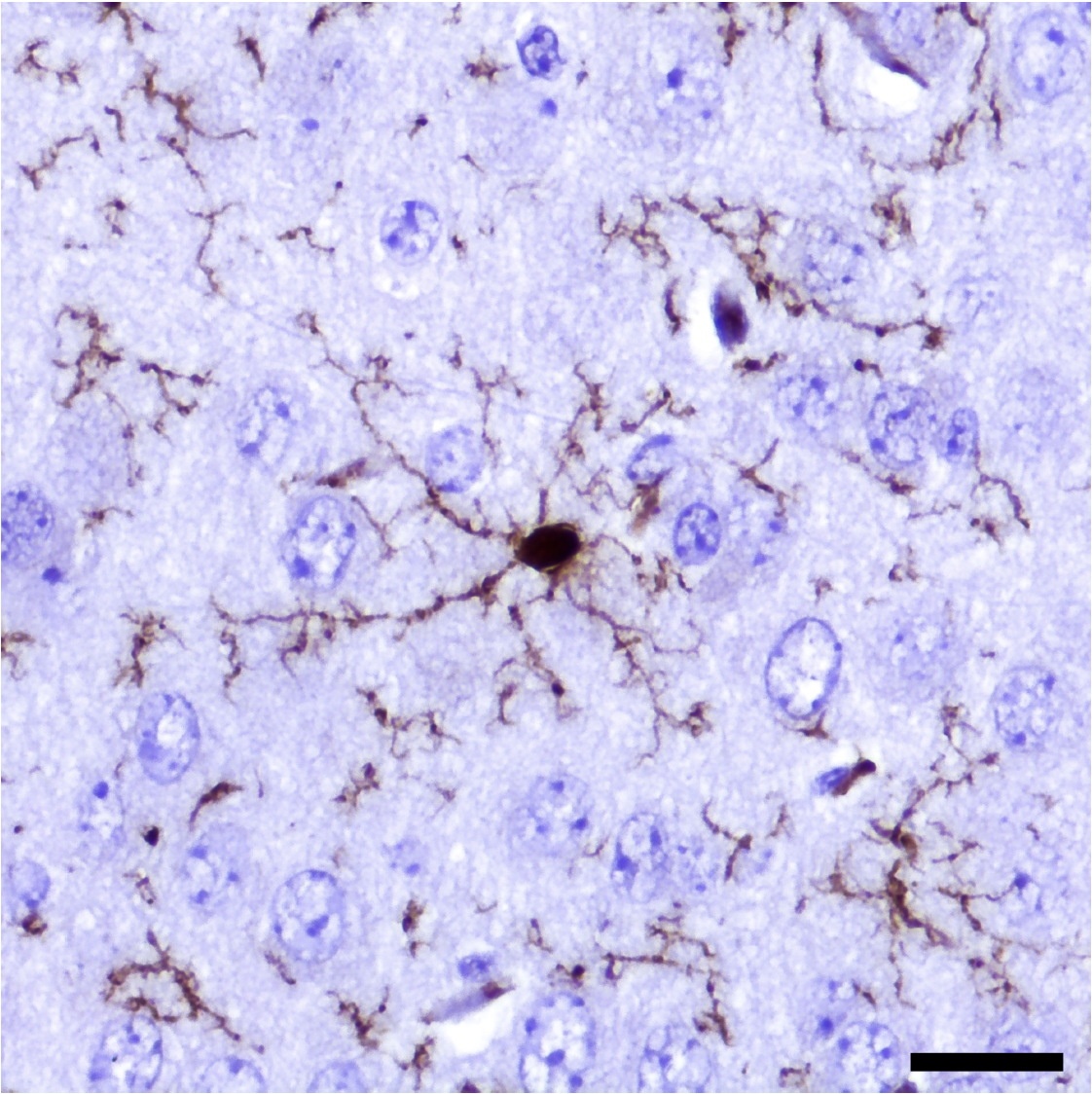

Supplement: Supplementary file 6 — Source data Fig. 1 [file 44319_2026_721_MOESM6_ESM.zip › 1A/DAB images-Microglia/Control/Screenshot 2025-09-01 at 13.46.41.tif]

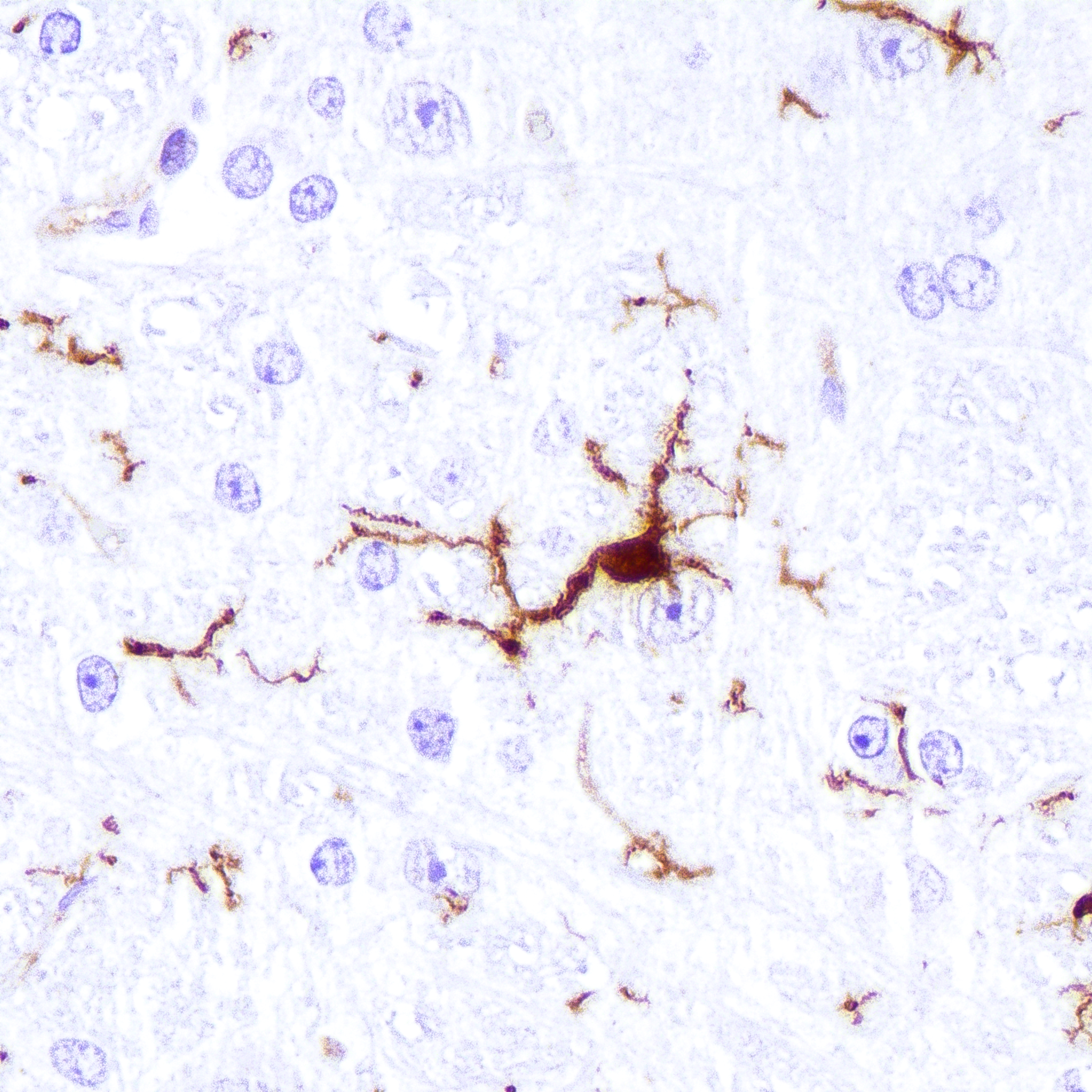

Supplement: Supplementary file 6 — Source data Fig. 1 [file 44319_2026_721_MOESM6_ESM.zip › 1C/DAB images-Different brain regions/Controls/striatum.tif]

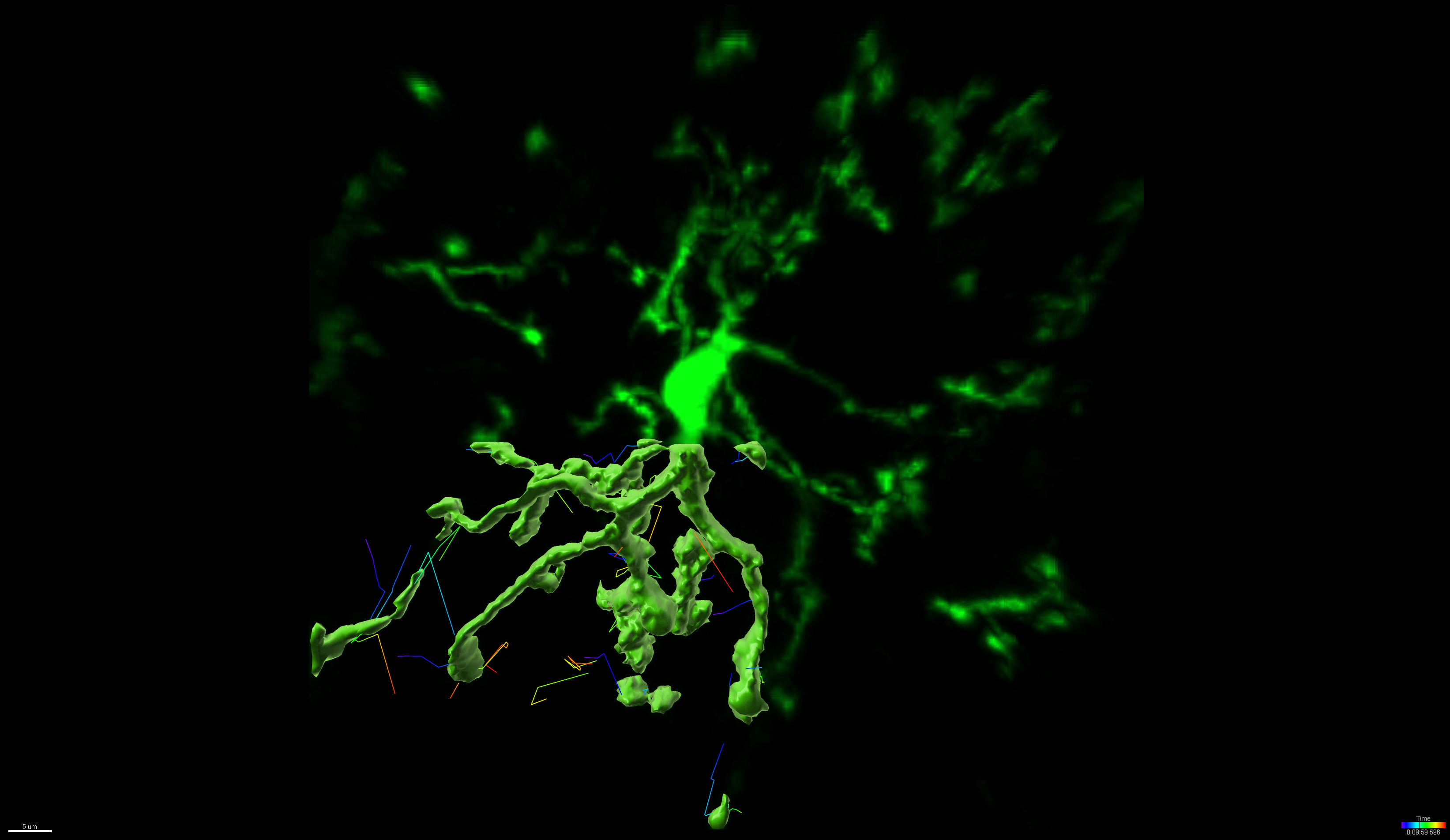

Supplement: Supplementary file 7 — Source data Fig. 2 [file 44319_2026_721_MOESM7_ESM.zip › 2A/Control/Zoom-in/10min/GFP-surface.tif]

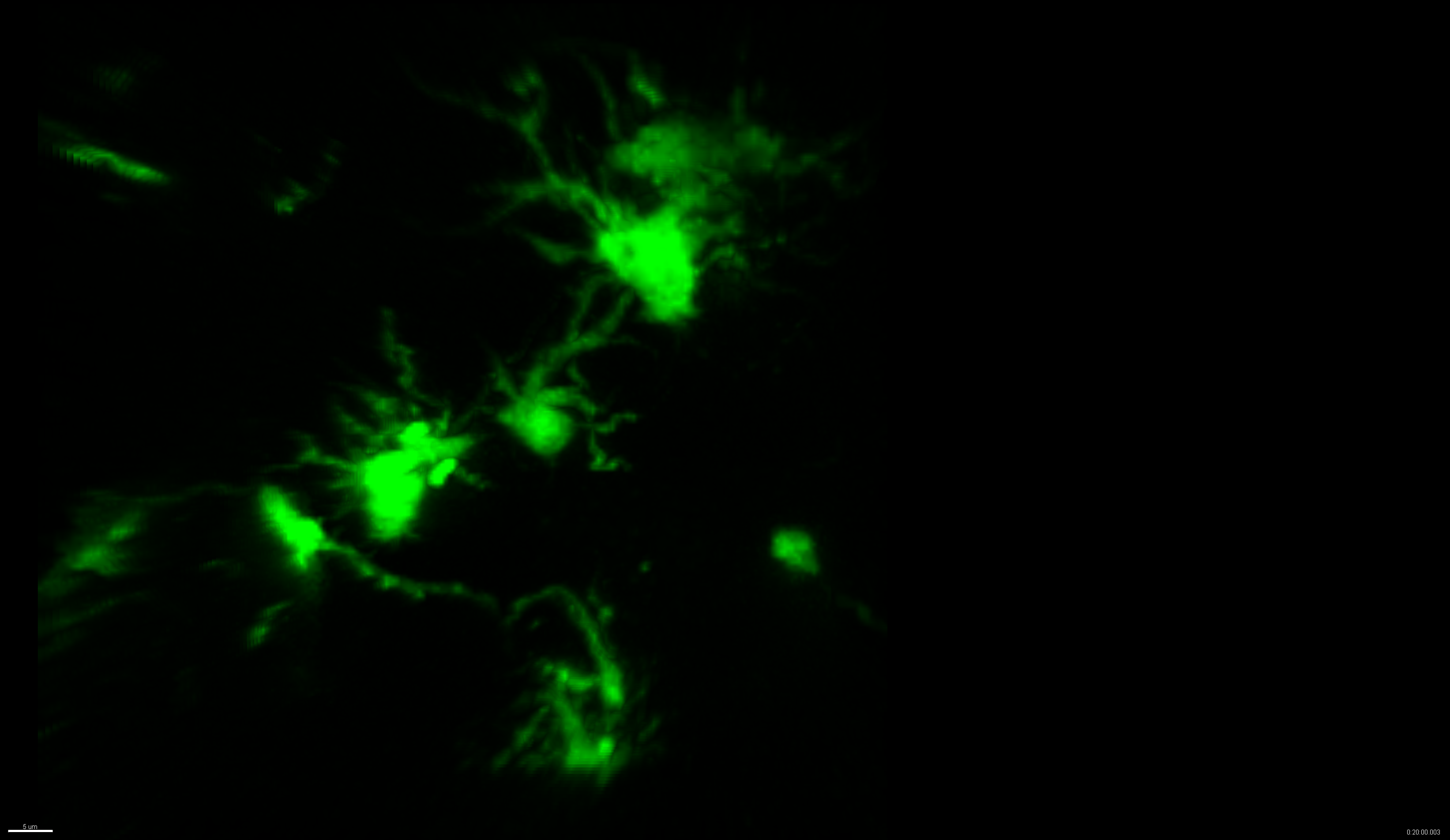

Supplement: Supplementary file 7 — Source data Fig. 2 [file 44319_2026_721_MOESM7_ESM.zip › 2B/KO/Zoom-in/20min/GFP-original.tif]

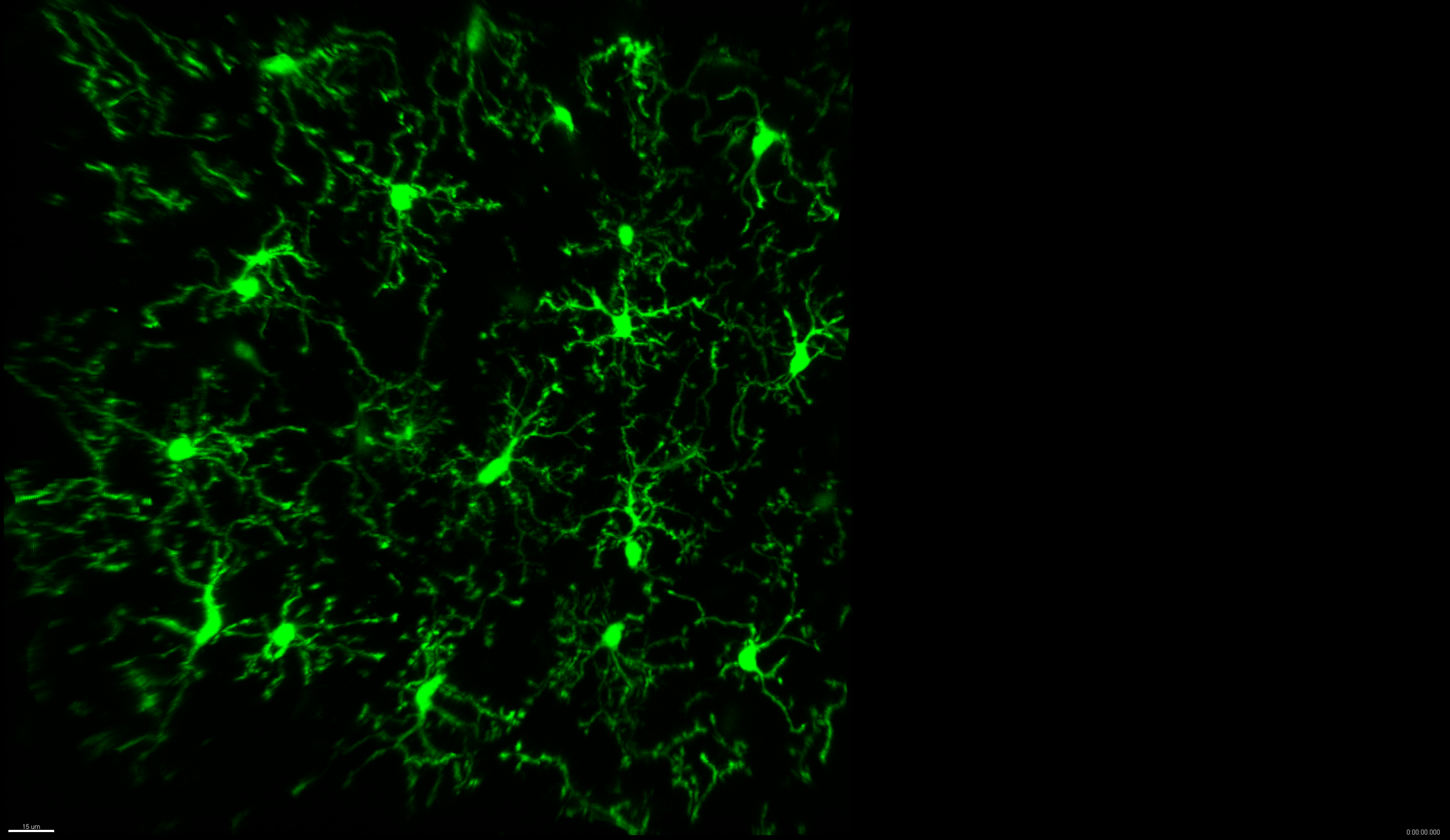

Supplement: Supplementary file 7 — Source data Fig. 2 [file 44319_2026_721_MOESM7_ESM.zip › 2A/Control/Overview/0 min_controlarp-gfp_LAE3059_101022_sl1001_2025-09-09T09-59-10.836.tif]

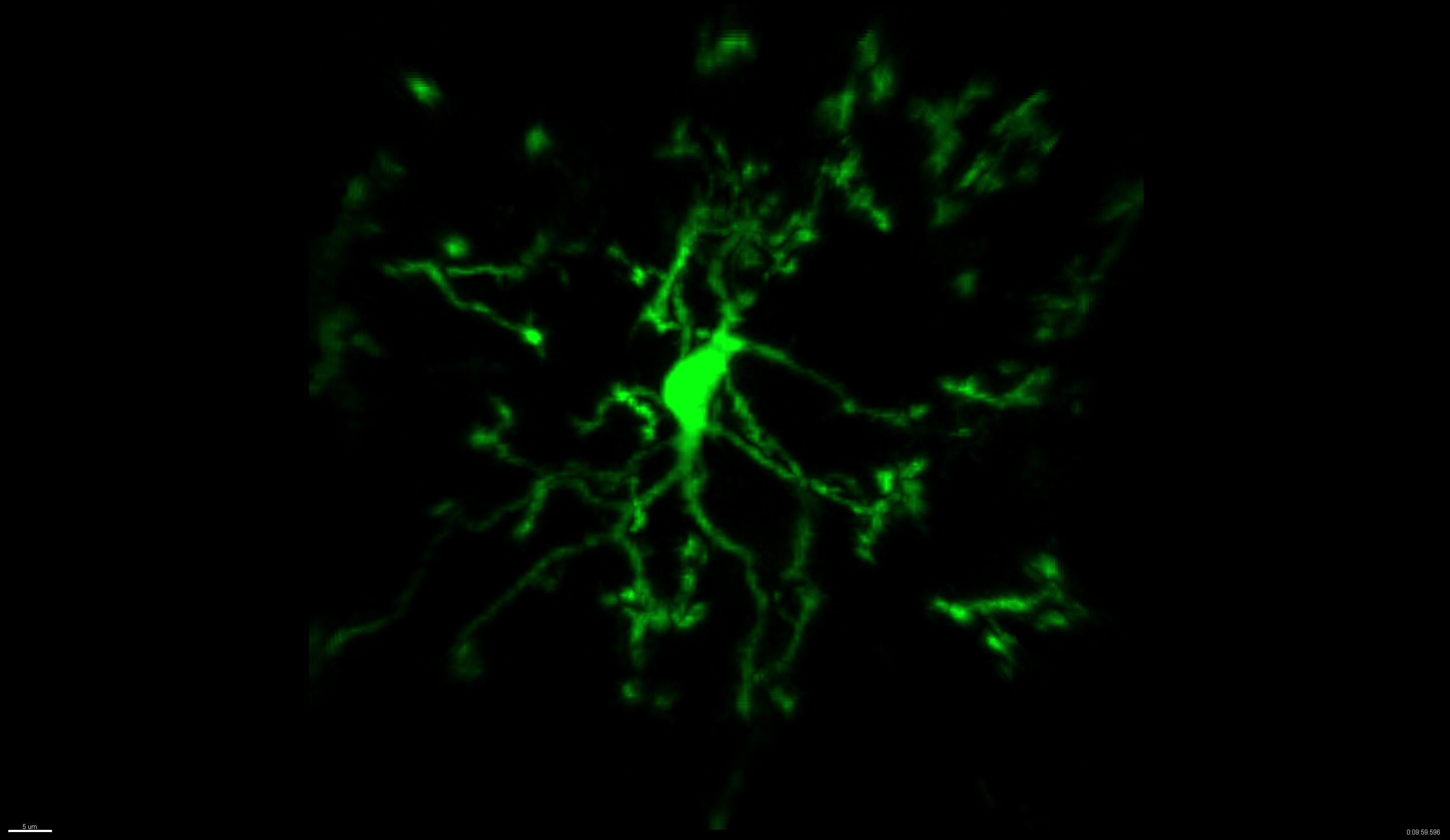

Supplement: Supplementary file 7 — Source data Fig. 2 [file 44319_2026_721_MOESM7_ESM.zip › 2A/Control/Zoom-in/10min/GFP-original.tif]

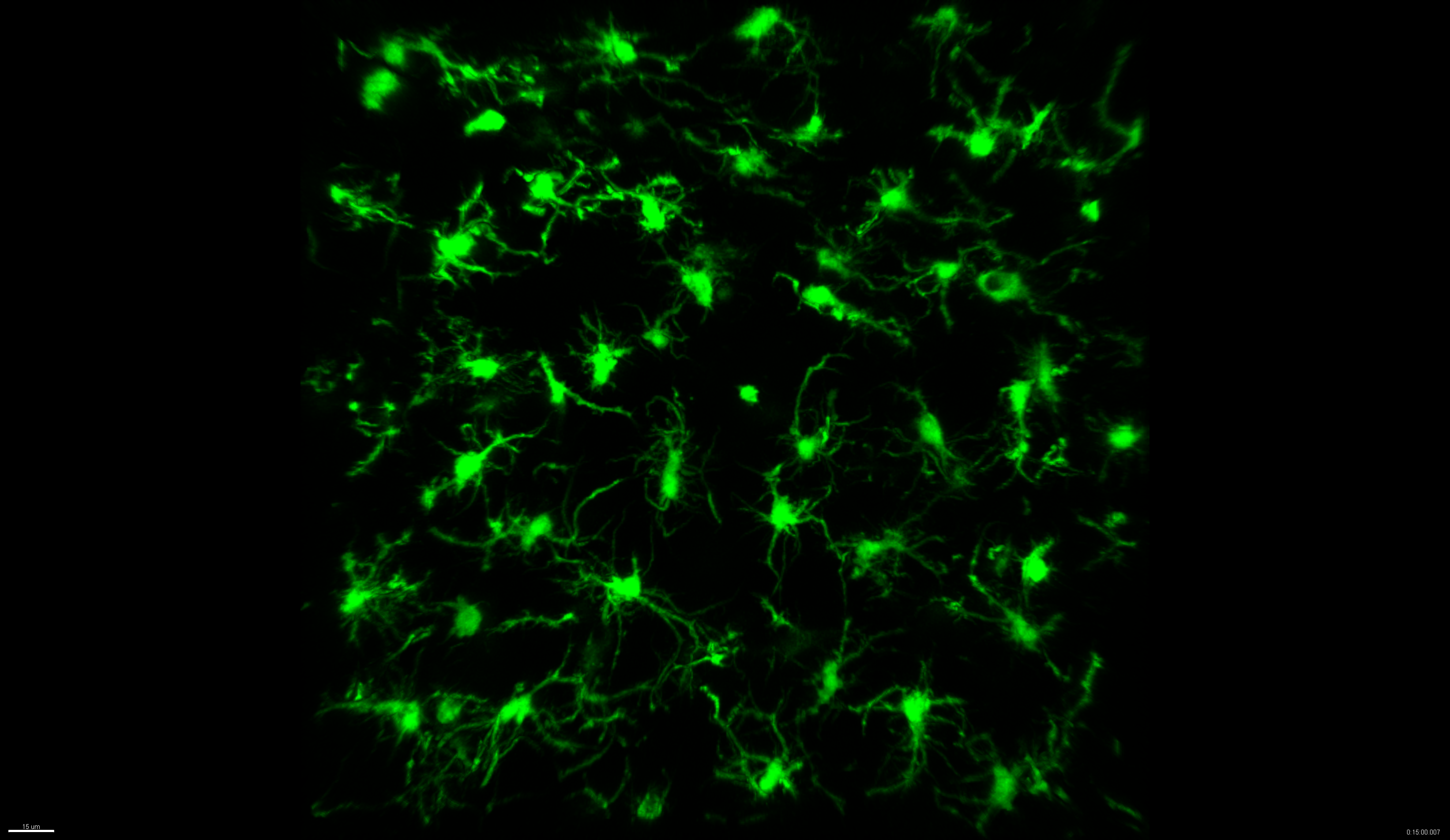

Supplement: Supplementary file 7 — Source data Fig. 2 [file 44319_2026_721_MOESM7_ESM.zip › 2B/KO/Overview/15min_arp-gfp_acsf_noTTX_121022_sl10023_2025-09-09T10-12-19.539.tif]

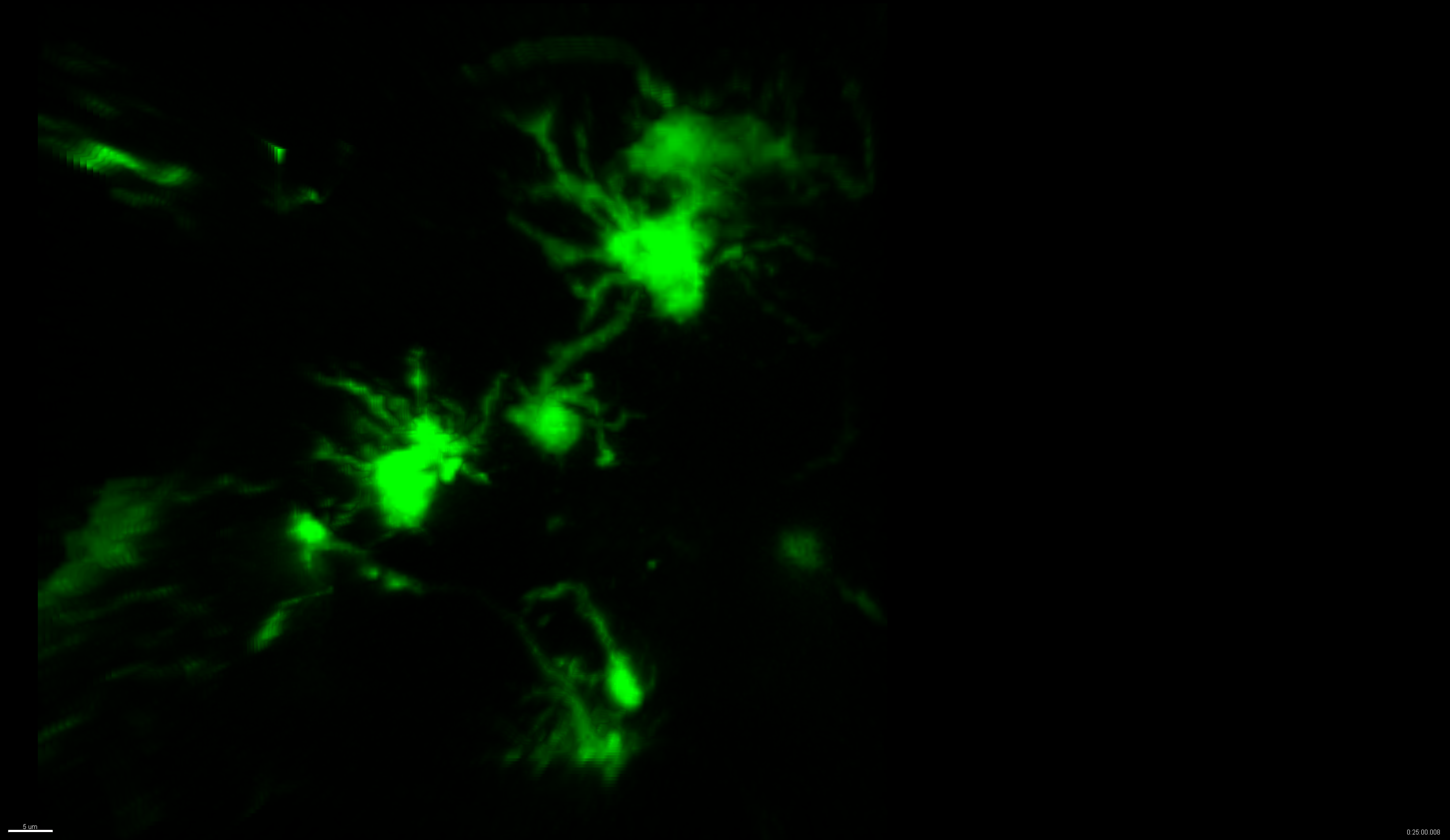

Supplement: Supplementary file 7 — Source data Fig. 2 [file 44319_2026_721_MOESM7_ESM.zip › 2B/KO/Zoom-in/25min/GFP-original.tif]

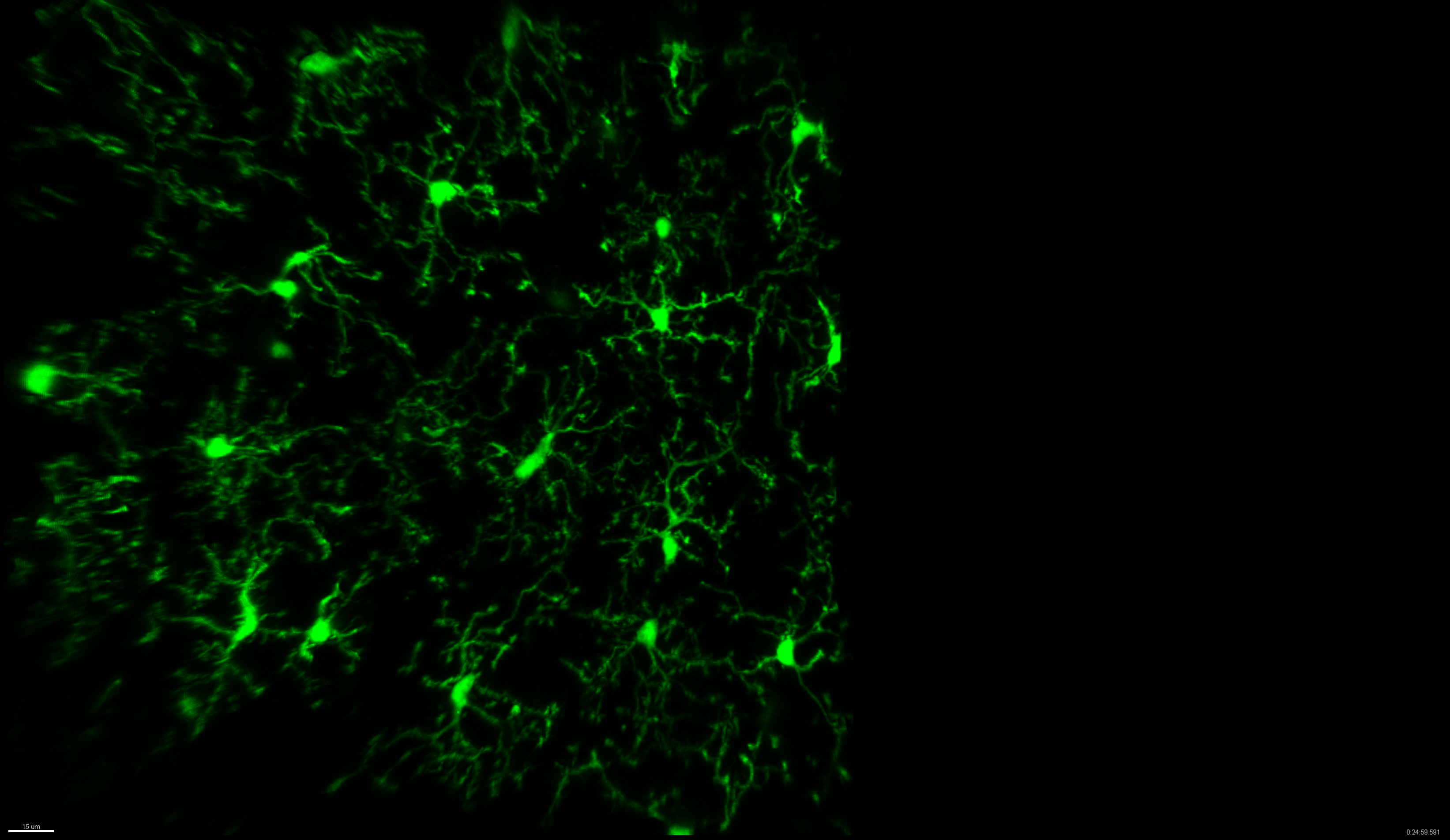

Supplement: Supplementary file 7 — Source data Fig. 2 [file 44319_2026_721_MOESM7_ESM.zip › 2A/Control/Overview/25min_controlarp-gfp_LAE3059_101022_sl1001_2025-09-09T09-59-37.611.tif]

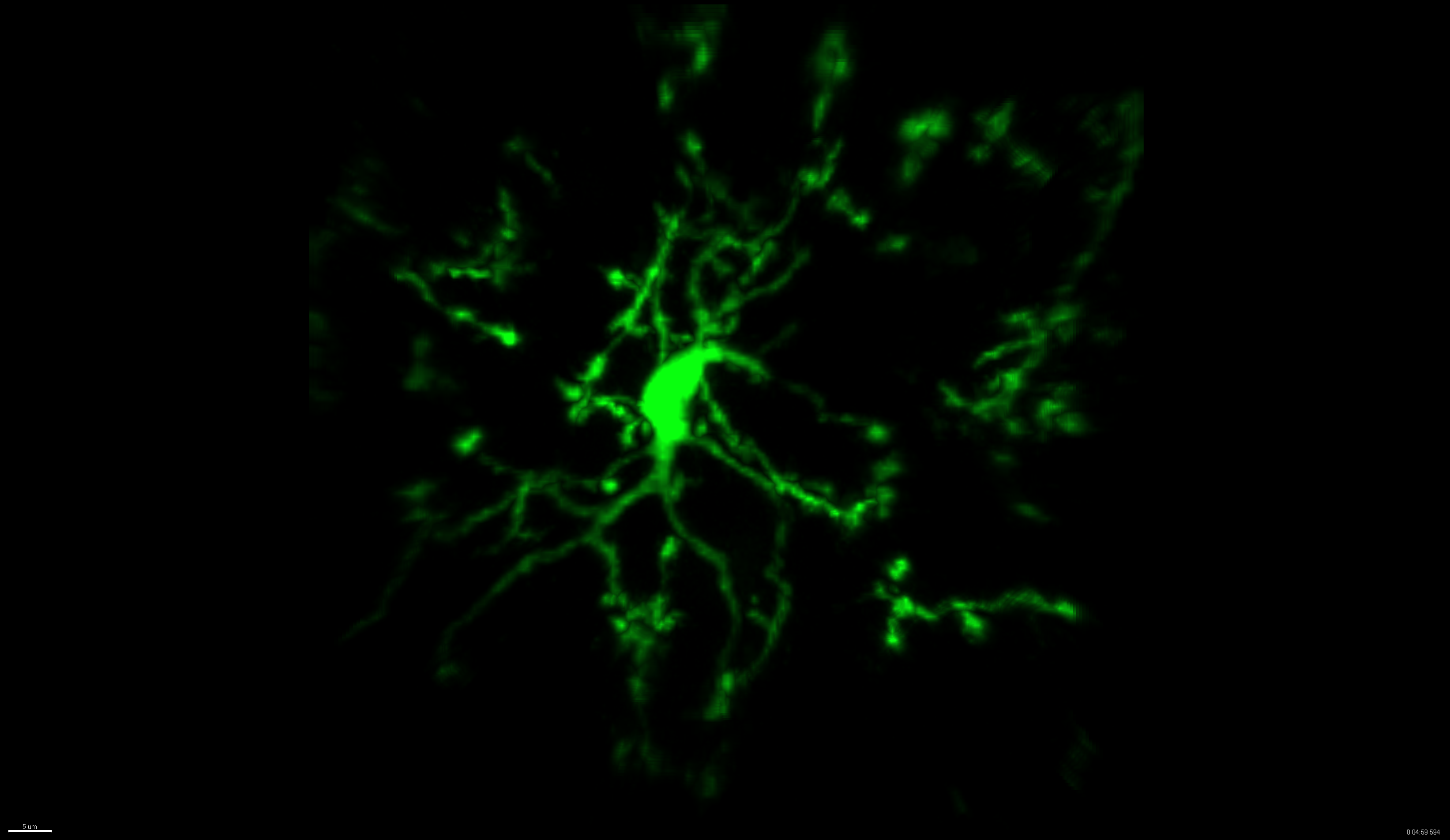

Supplement: Supplementary file 7 — Source data Fig. 2 [file 44319_2026_721_MOESM7_ESM.zip › 2A/Control/Zoom-in/5min/GFP-original.tif]

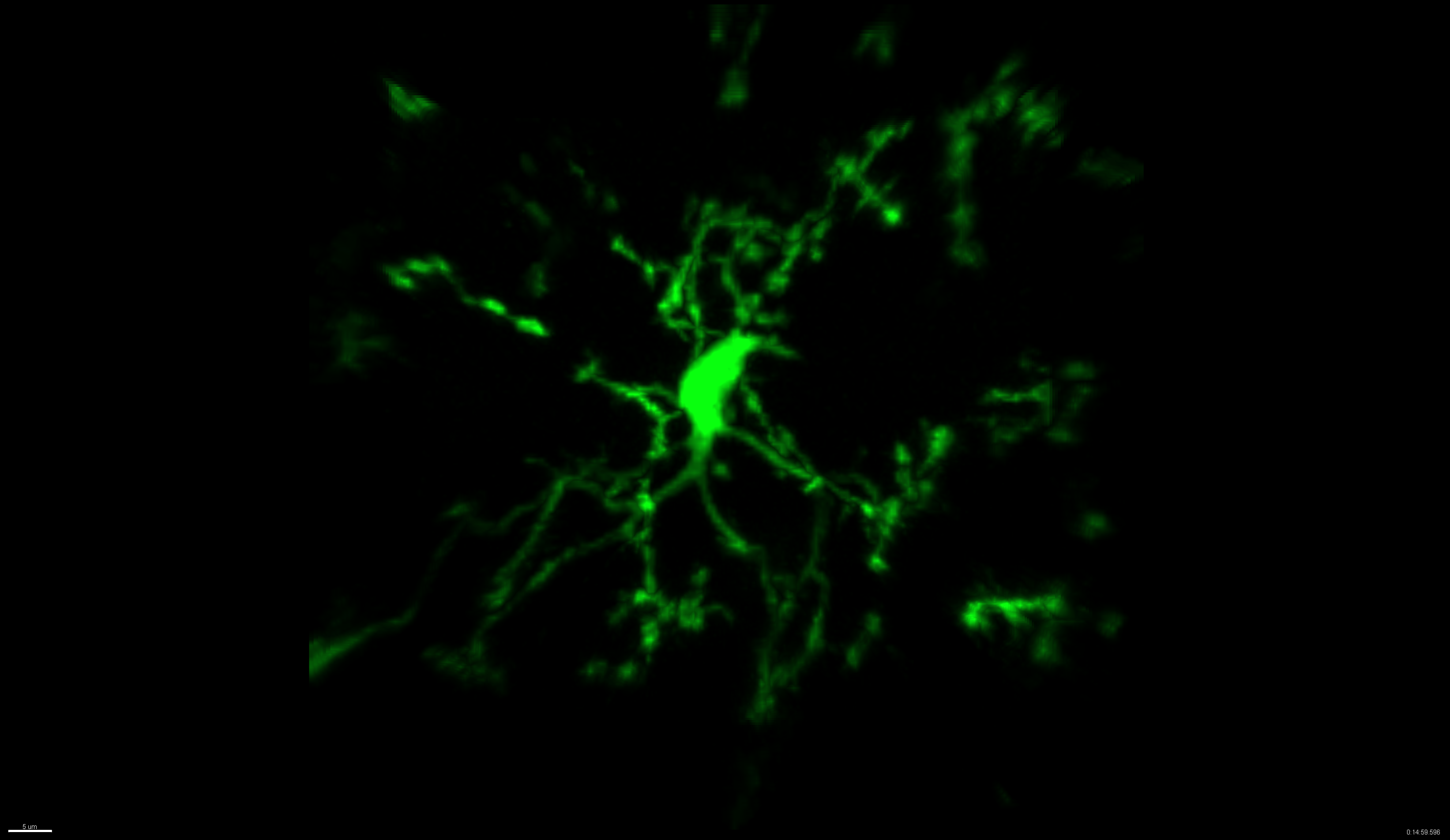

Supplement: Supplementary file 7 — Source data Fig. 2 [file 44319_2026_721_MOESM7_ESM.zip › 2A/Control/Zoom-in/15min/GFP-original.tif]

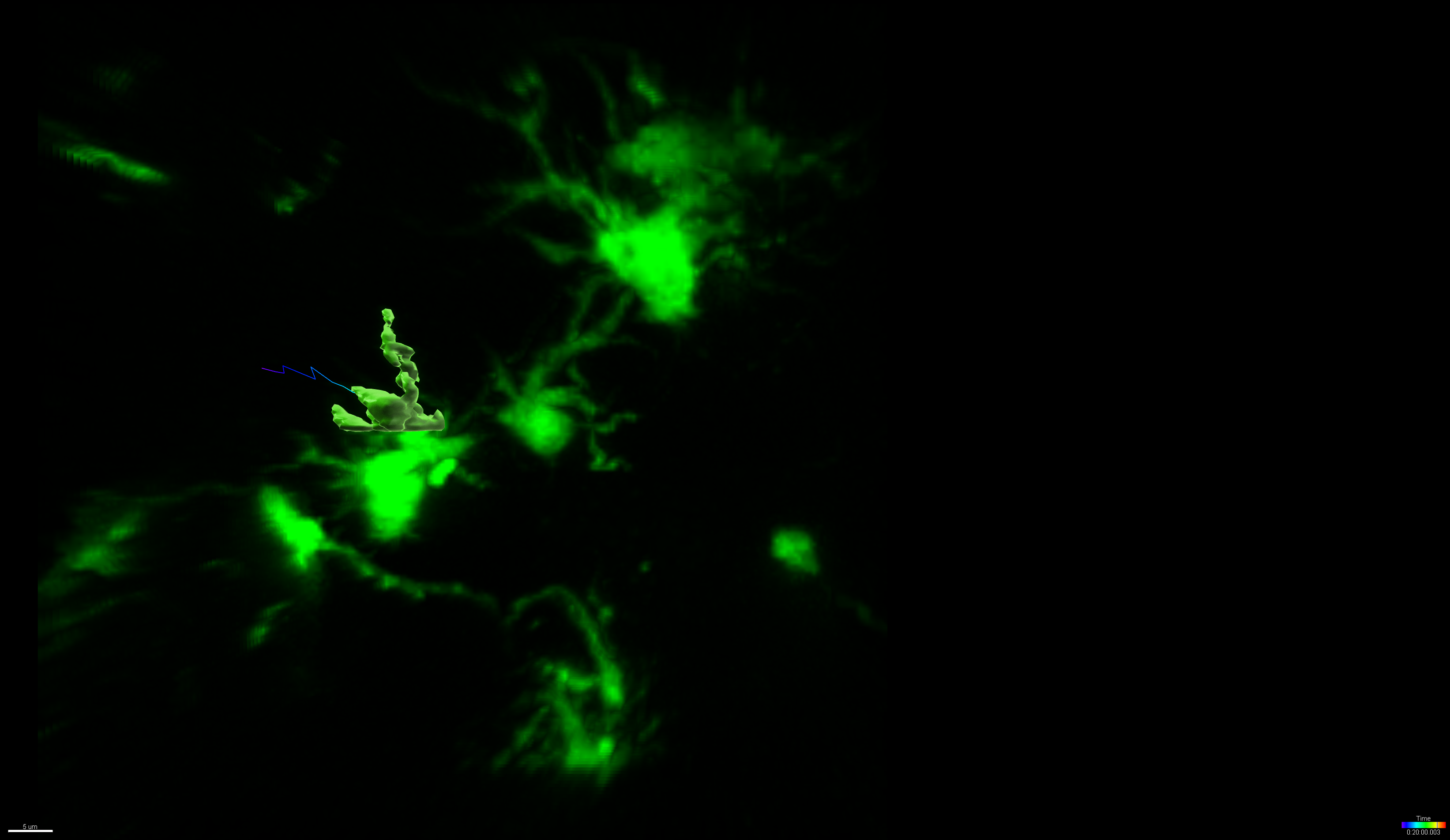

Supplement: Supplementary file 7 — Source data Fig. 2 [file 44319_2026_721_MOESM7_ESM.zip › 2B/KO/Zoom-in/20min/GFP-surface.tif]

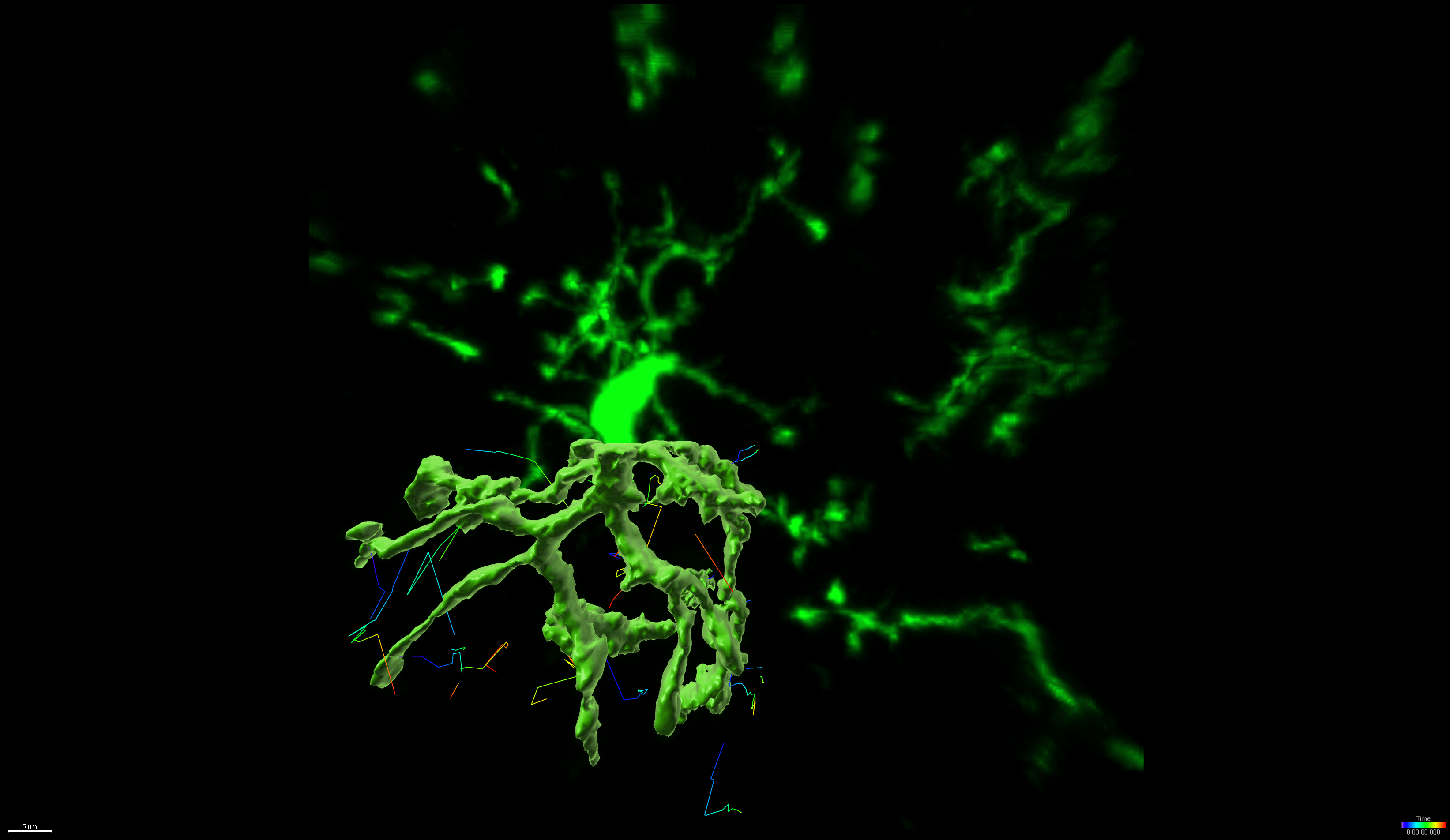

Supplement: Supplementary file 7 — Source data Fig. 2 [file 44319_2026_721_MOESM7_ESM.zip › 2A/Control/Zoom-in/0min/GFP-surface.tif]

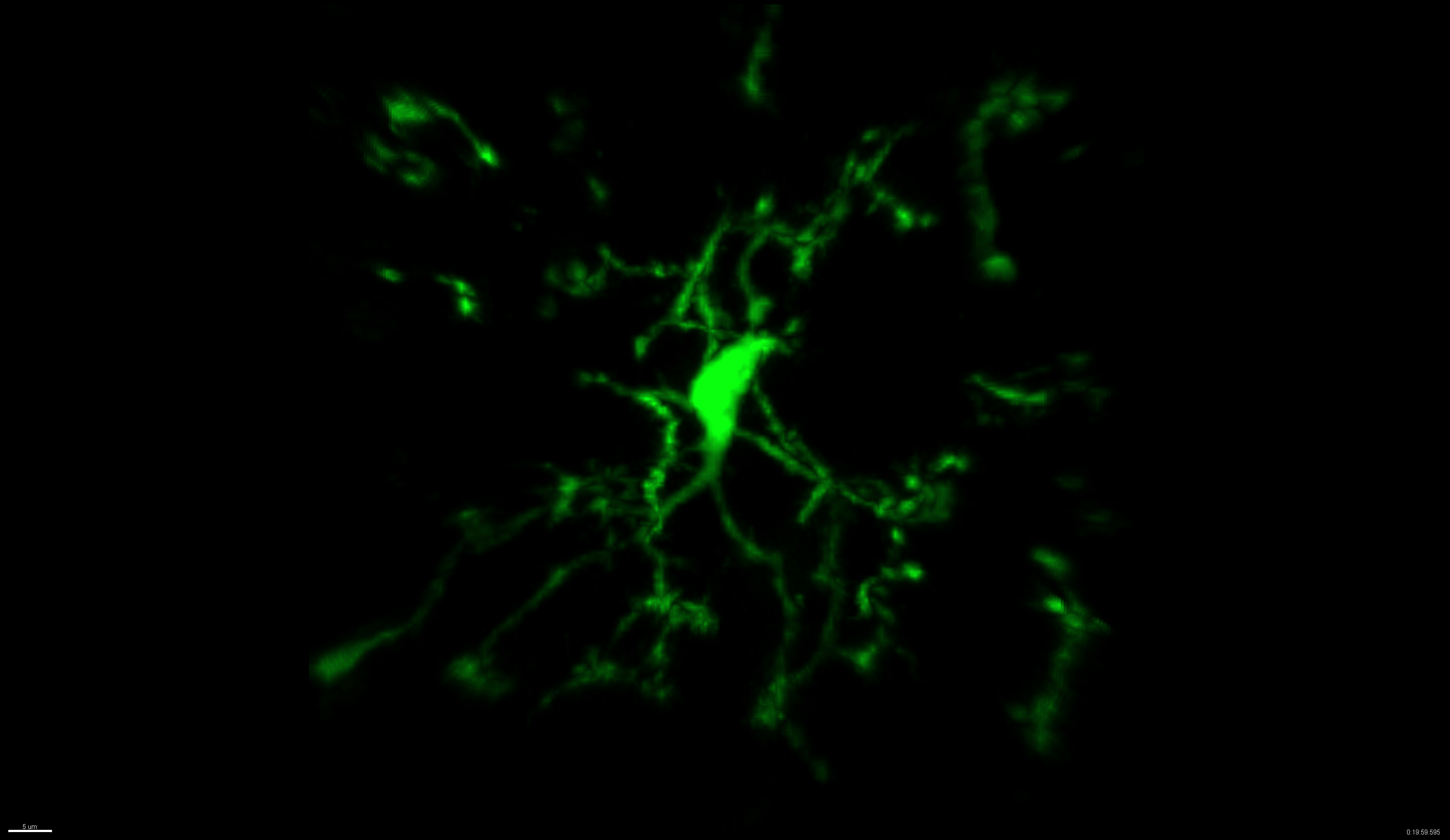

Supplement: Supplementary file 7 — Source data Fig. 2 [file 44319_2026_721_MOESM7_ESM.zip › 2A/Control/Zoom-in/20min/GFP-original.tif]

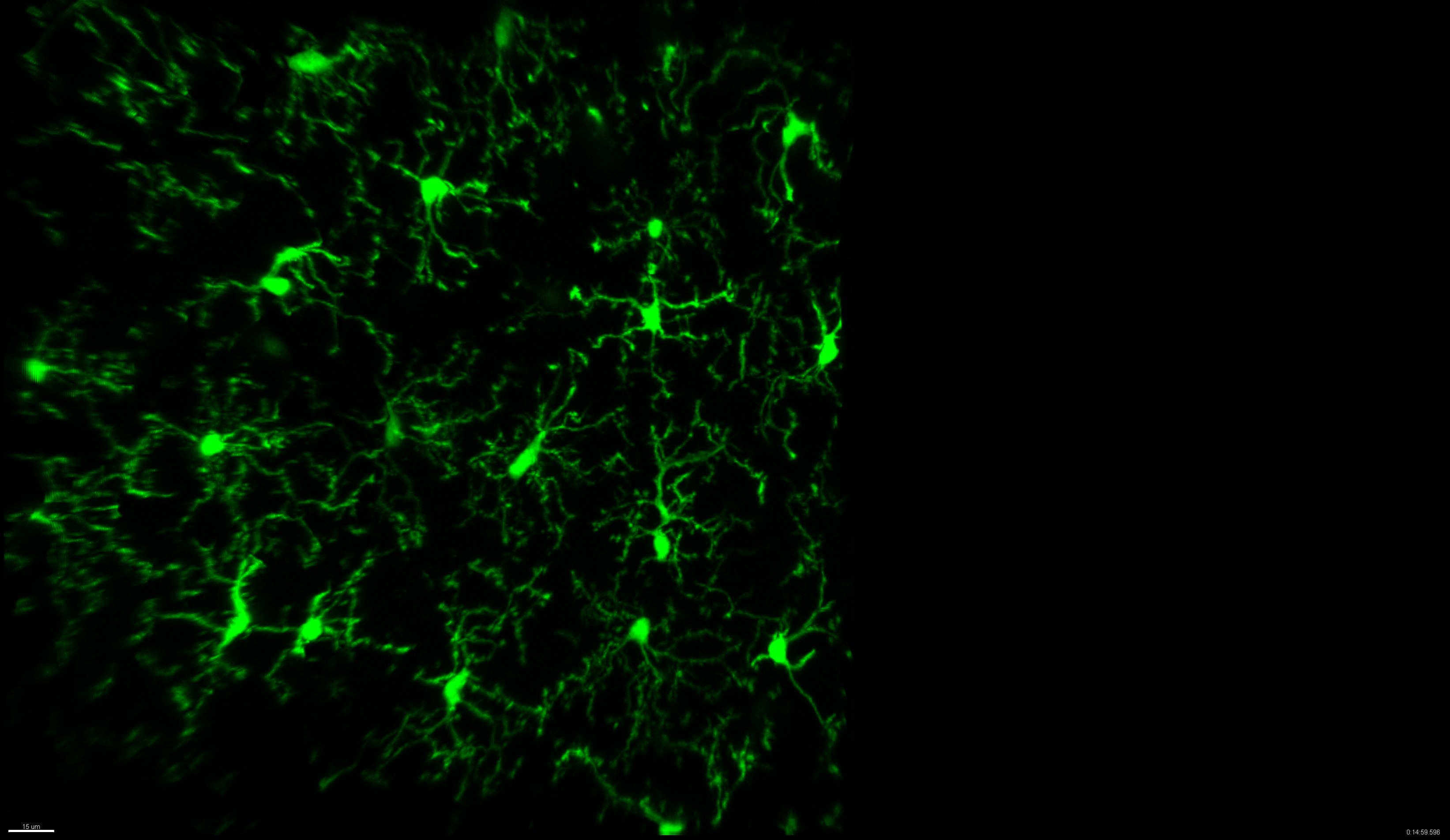

Supplement: Supplementary file 7 — Source data Fig. 2 [file 44319_2026_721_MOESM7_ESM.zip › 2A/Control/Overview/15min_controlarp-gfp_LAE3059_101022_sl1001_2025-09-09T09-59-25.189.tif]

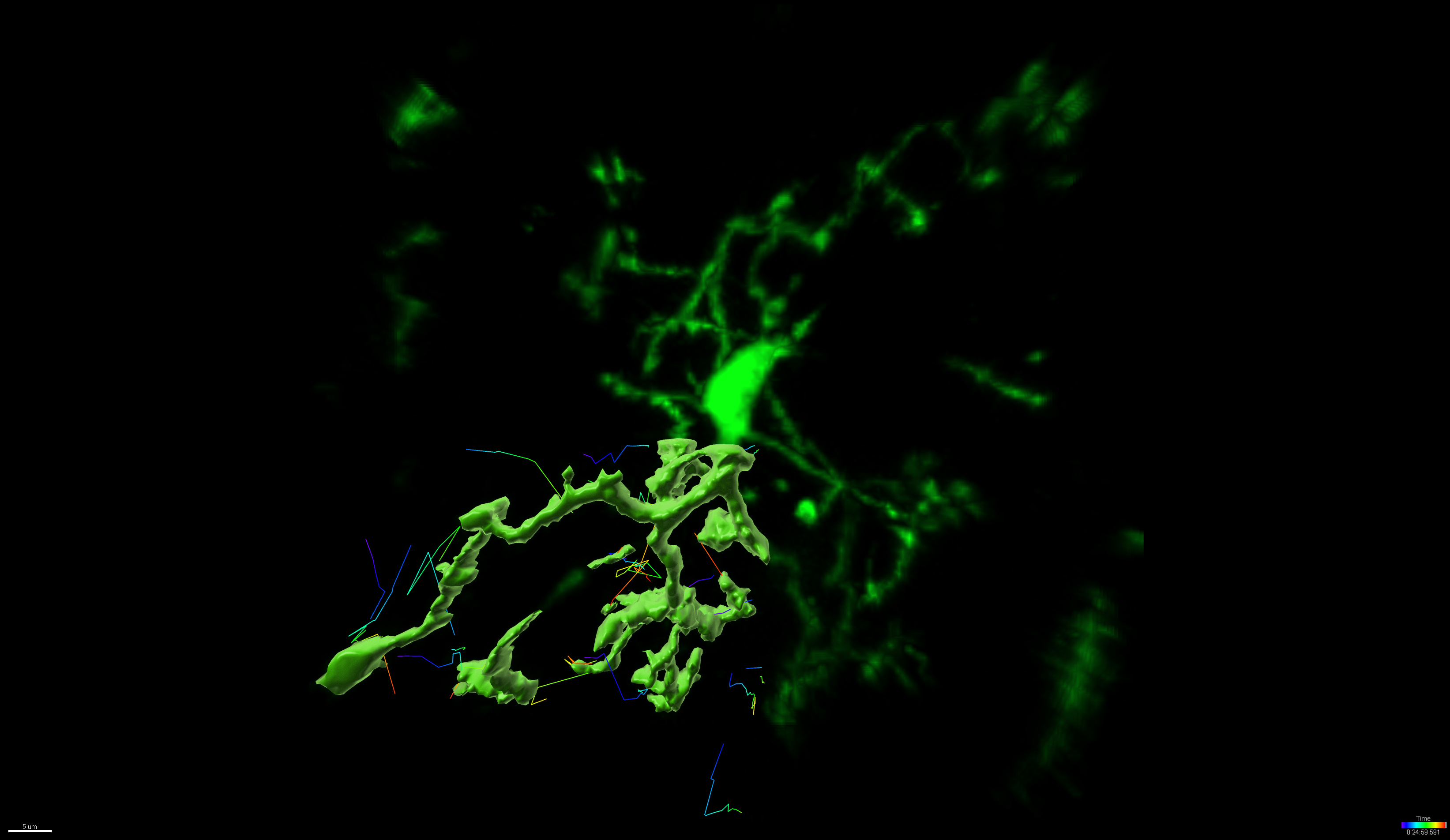

Supplement: Supplementary file 7 — Source data Fig. 2 [file 44319_2026_721_MOESM7_ESM.zip › 2A/Control/Zoom-in/25min/GFP-surface.tif]

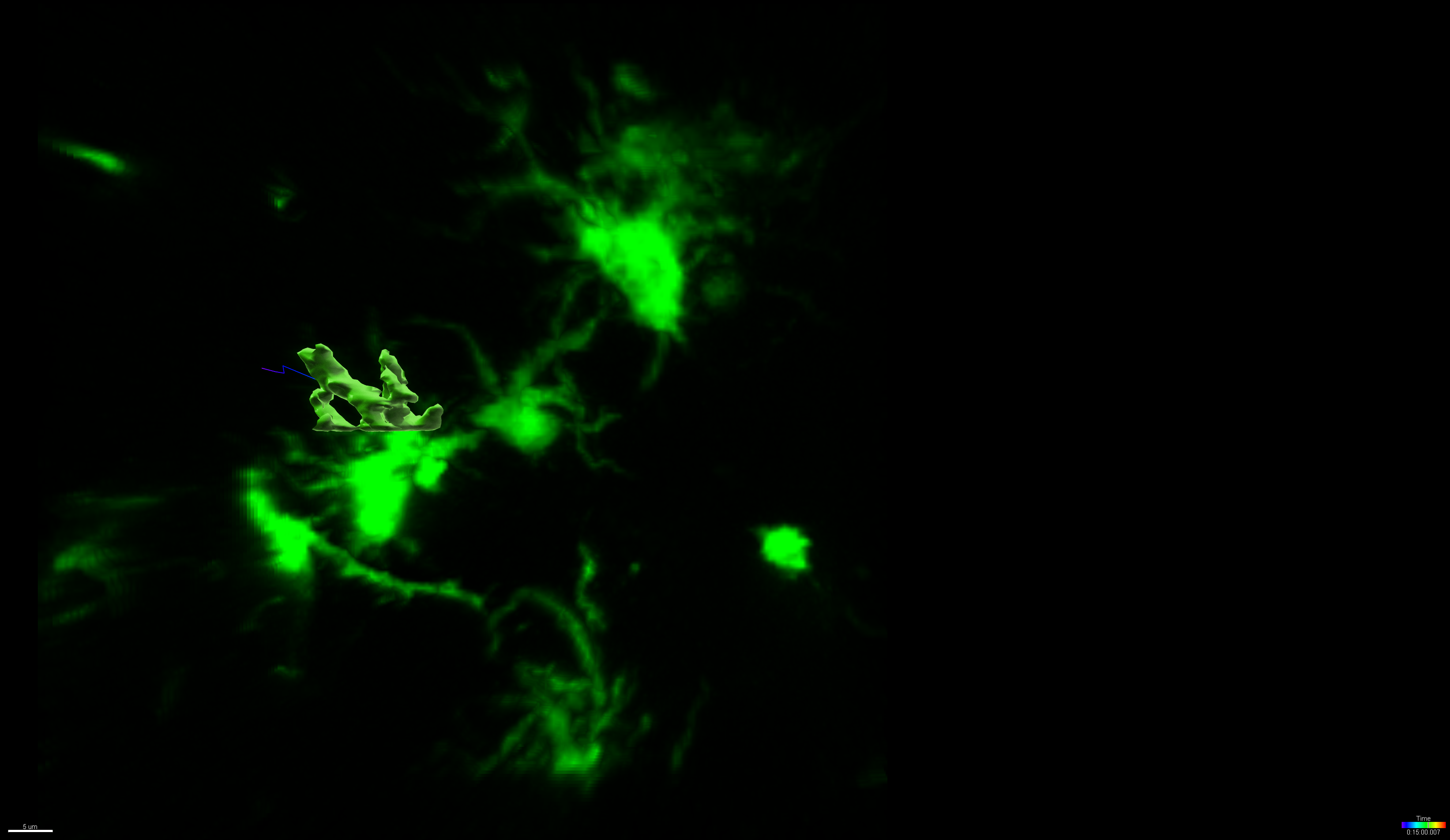

Supplement: Supplementary file 7 — Source data Fig. 2 [file 44319_2026_721_MOESM7_ESM.zip › 2B/KO/Zoom-in/15min/GFP-surface.tif]

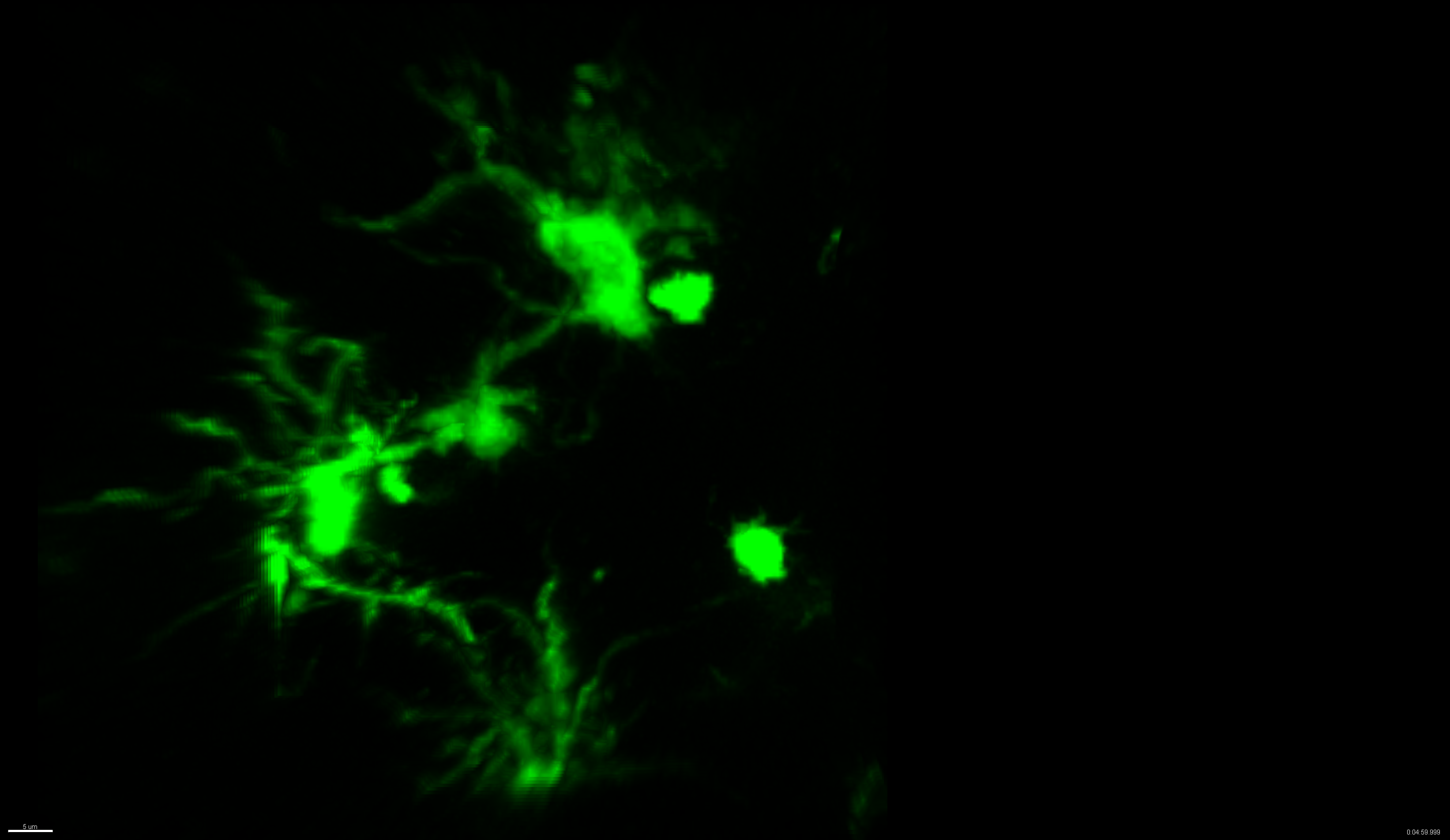

Supplement: Supplementary file 7 — Source data Fig. 2 [file 44319_2026_721_MOESM7_ESM.zip › 2B/KO/Zoom-in/5min/GFP-original.tif]

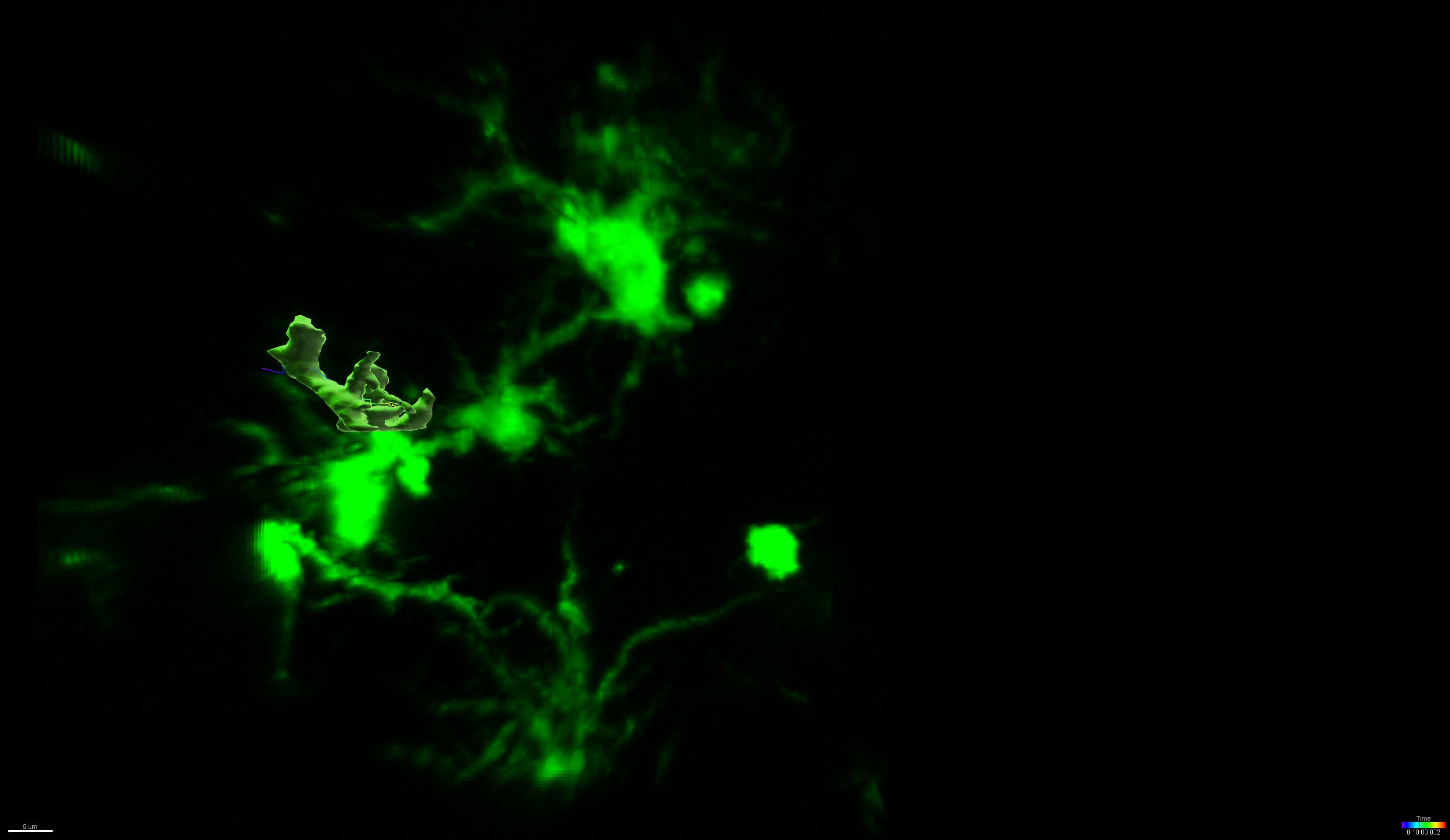

Supplement: Supplementary file 7 — Source data Fig. 2 [file 44319_2026_721_MOESM7_ESM.zip › 2B/KO/Zoom-in/10min/GFP-surface.tif]

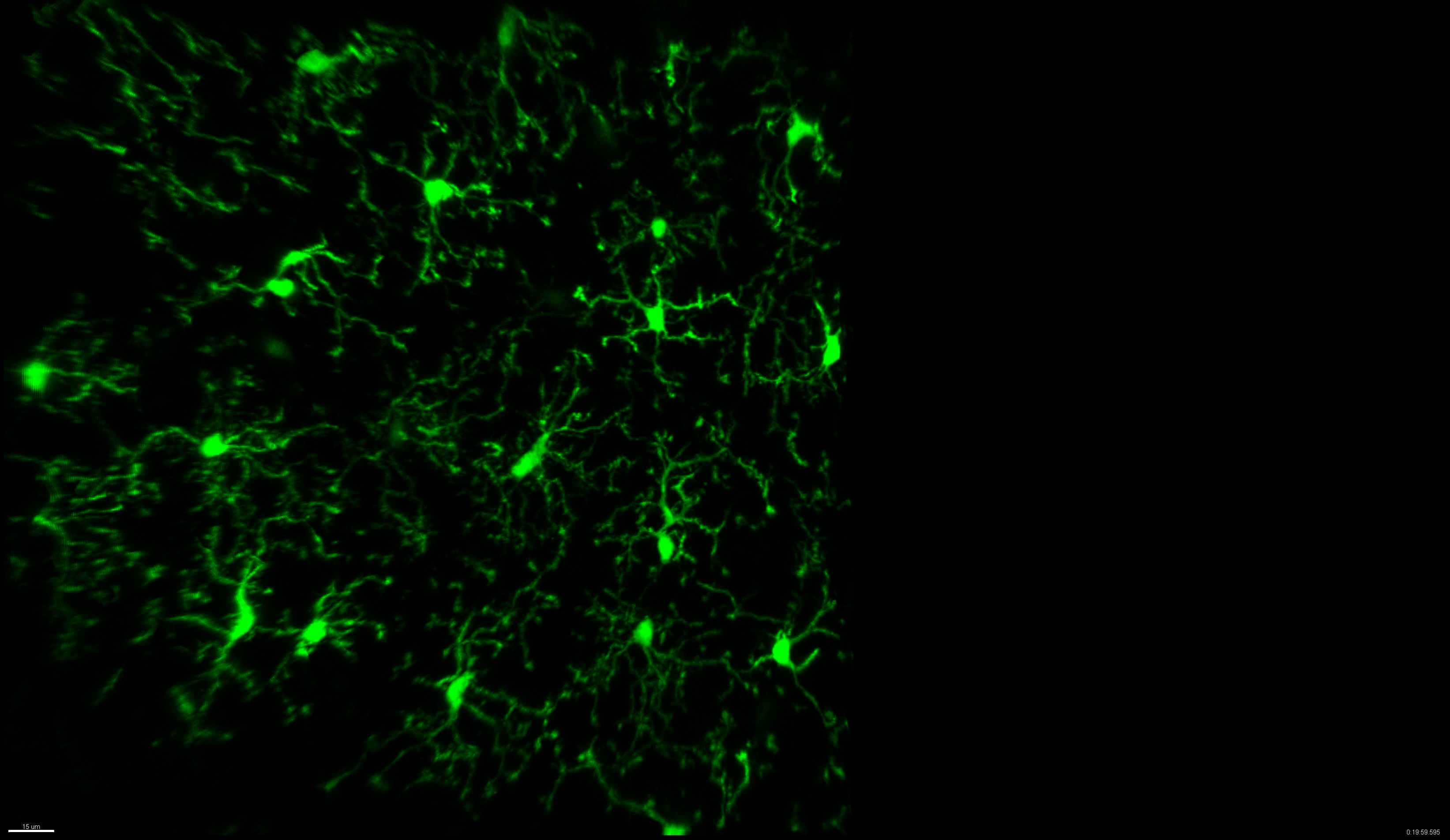

Supplement: Supplementary file 7 — Source data Fig. 2 [file 44319_2026_721_MOESM7_ESM.zip › 2A/Control/Overview/20min_controlarp-gfp_LAE3059_101022_sl1001_2025-09-09T09-59-30.098.tif]

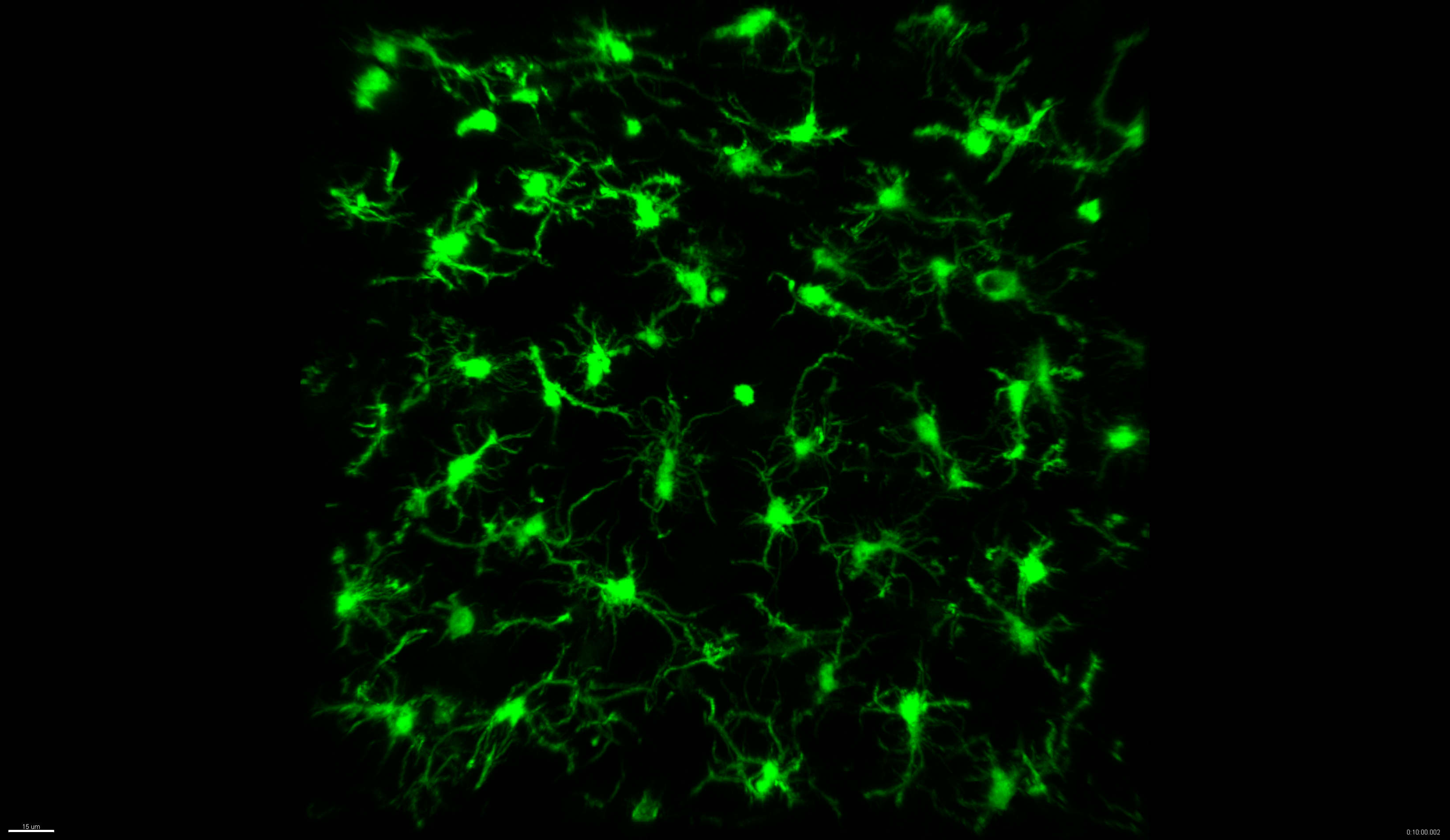

Supplement: Supplementary file 7 — Source data Fig. 2 [file 44319_2026_721_MOESM7_ESM.zip › 2B/KO/Overview/10min_arp-gfp_acsf_noTTX_121022_sl10023_2025-09-09T10-12-15.482.tif]

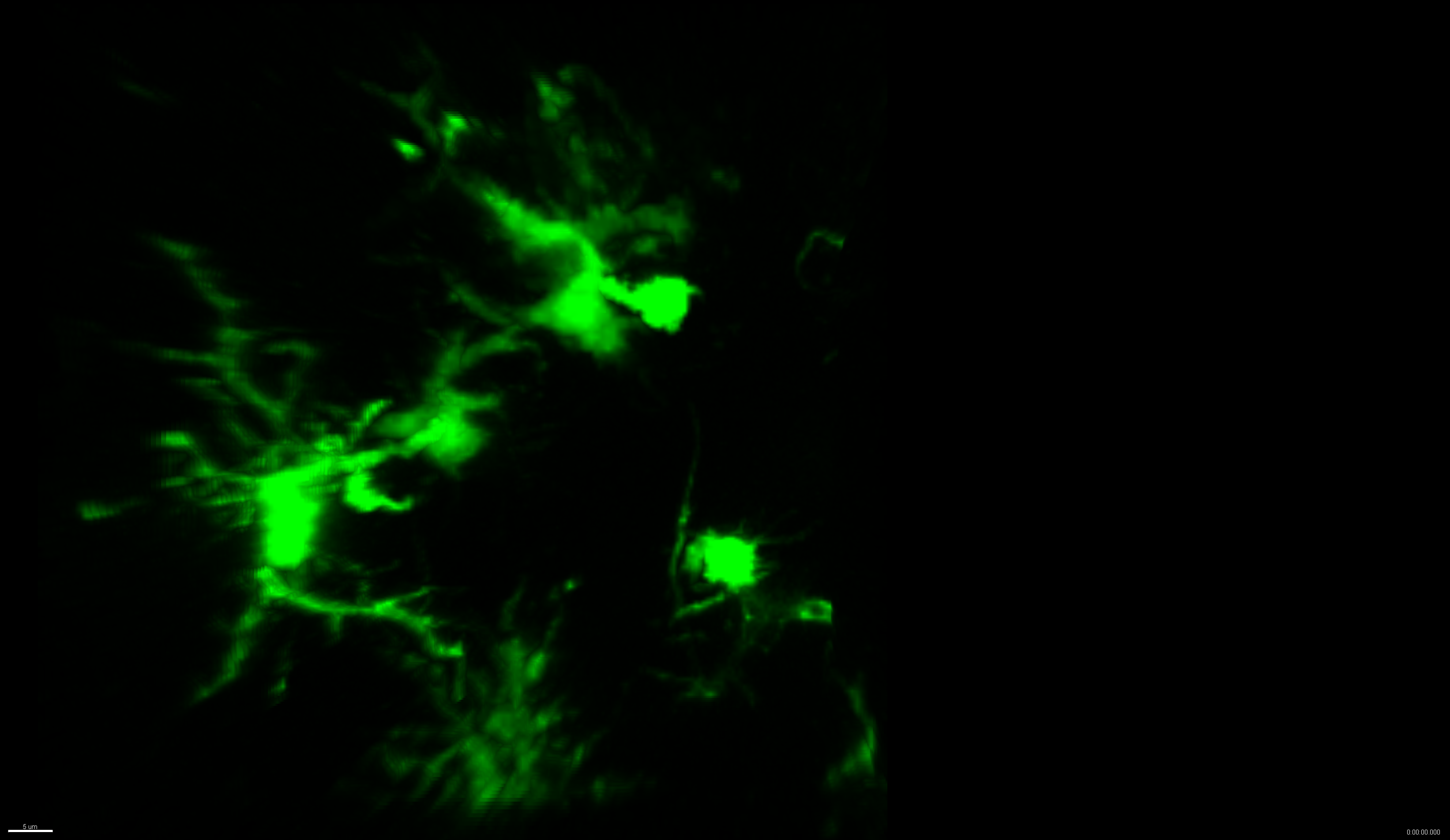

Supplement: Supplementary file 7 — Source data Fig. 2 [file 44319_2026_721_MOESM7_ESM.zip › 2B/KO/Zoom-in/0min/GFP-original.tif]

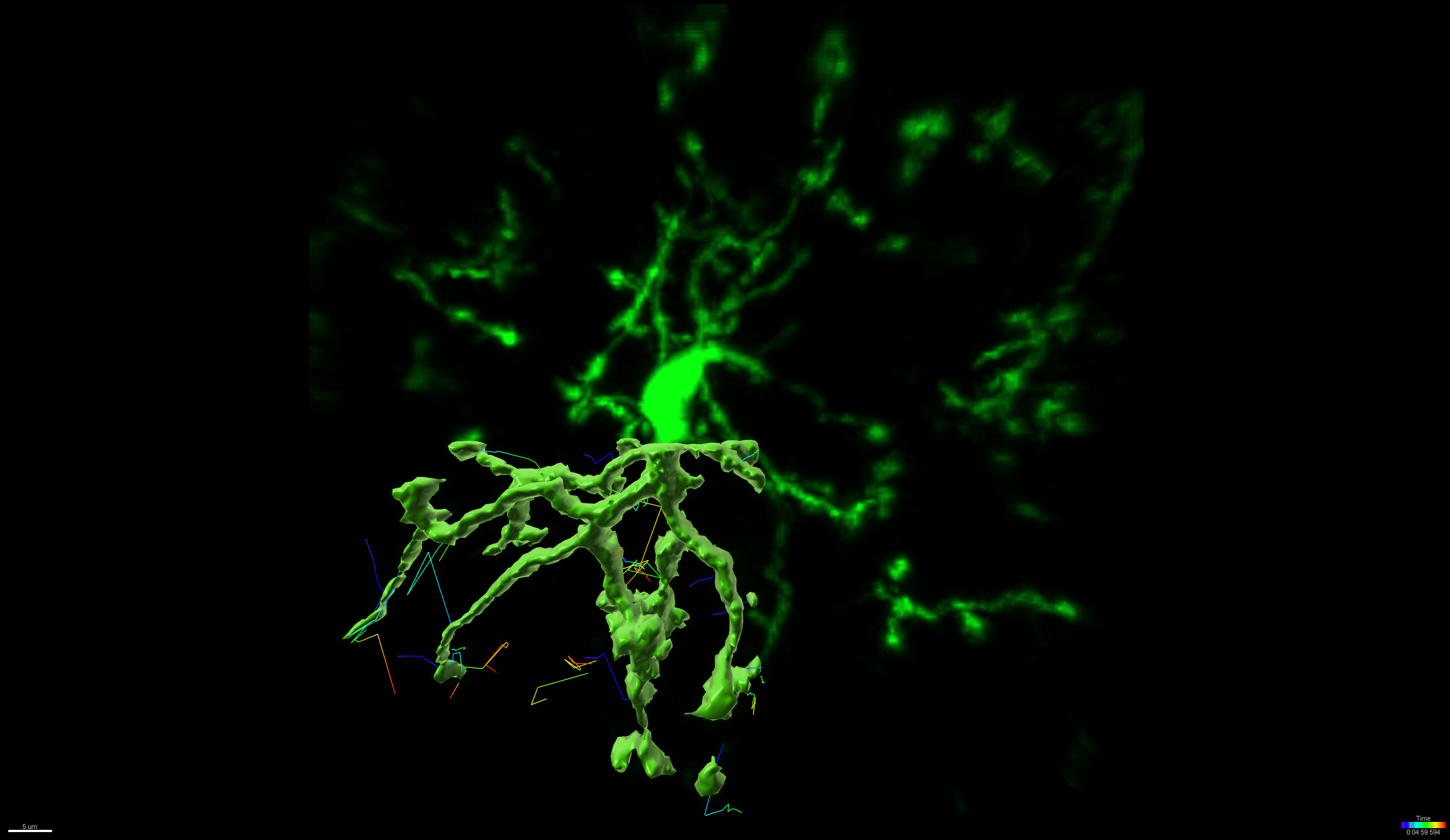

Supplement: Supplementary file 7 — Source data Fig. 2 [file 44319_2026_721_MOESM7_ESM.zip › 2A/Control/Zoom-in/5min/GFP-surface.tif]

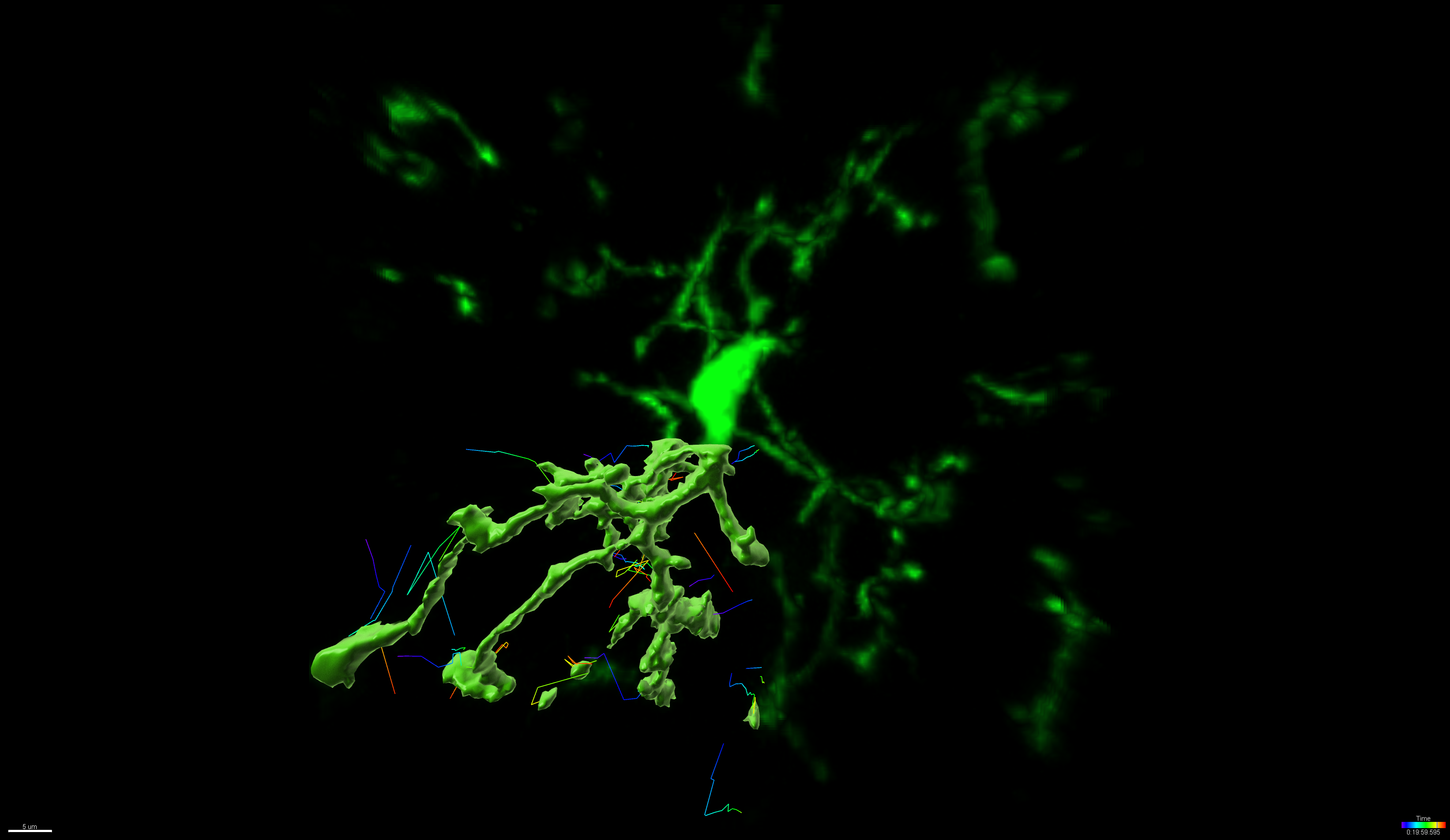

Supplement: Supplementary file 7 — Source data Fig. 2 [file 44319_2026_721_MOESM7_ESM.zip › 2A/Control/Zoom-in/20min/GFP-surface.tif]

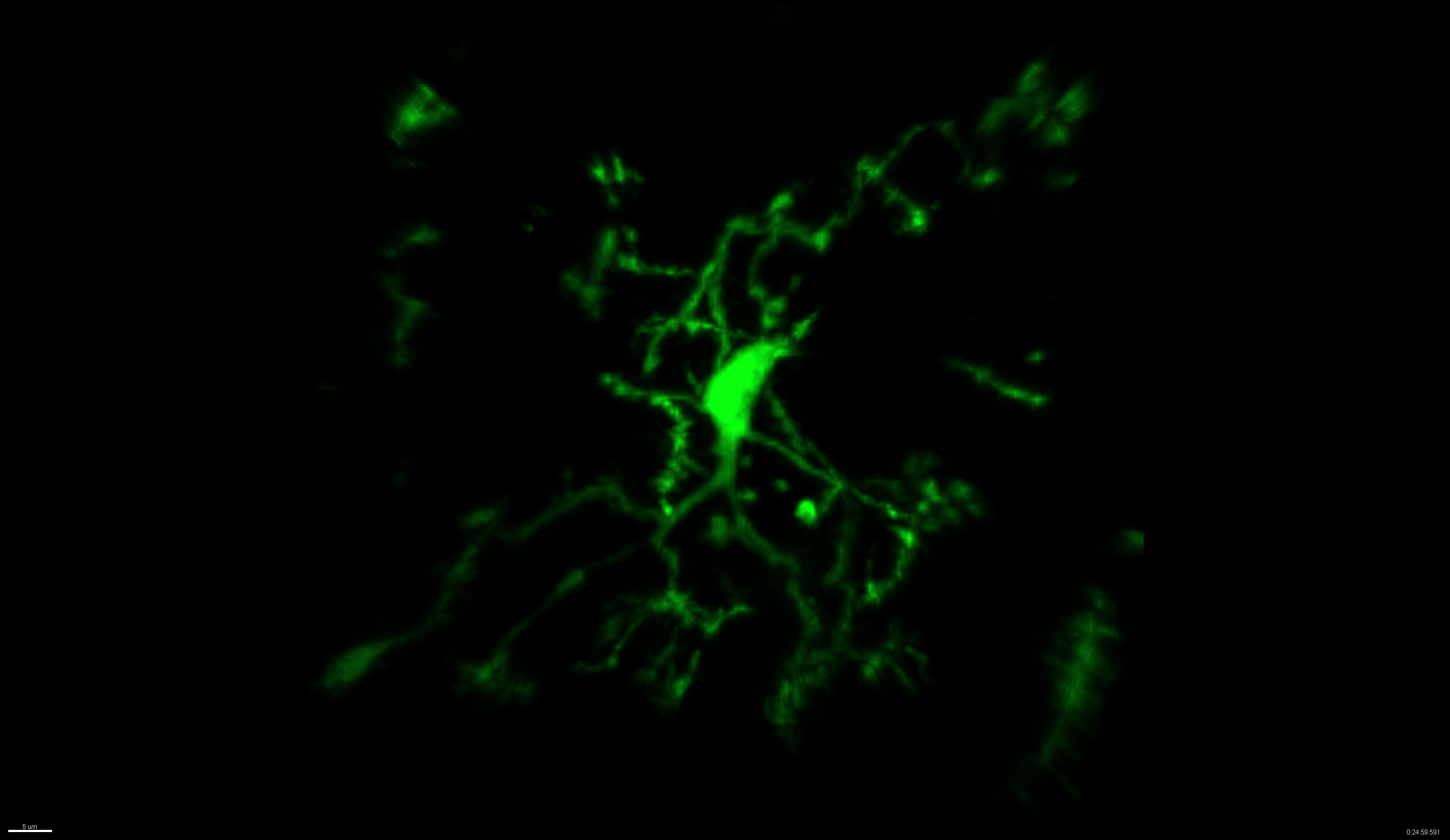

Supplement: Supplementary file 7 — Source data Fig. 2 [file 44319_2026_721_MOESM7_ESM.zip › 2A/Control/Zoom-in/25min/GFP-original.tif]

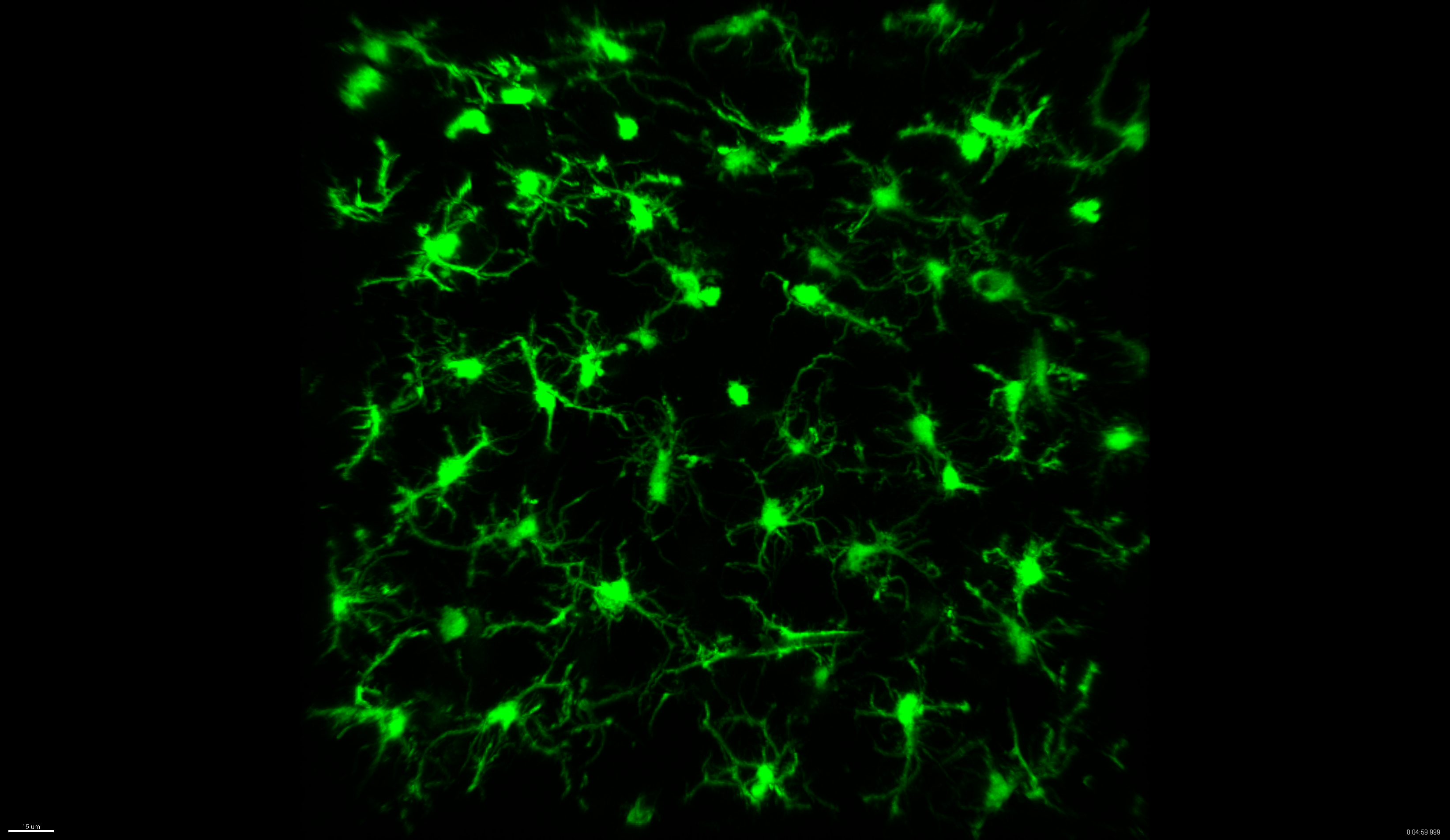

Supplement: Supplementary file 7 — Source data Fig. 2 [file 44319_2026_721_MOESM7_ESM.zip › 2B/KO/Overview/5min_arp-gfp_acsf_noTTX_121022_sl10023_2025-09-09T10-12-12.280.tif]

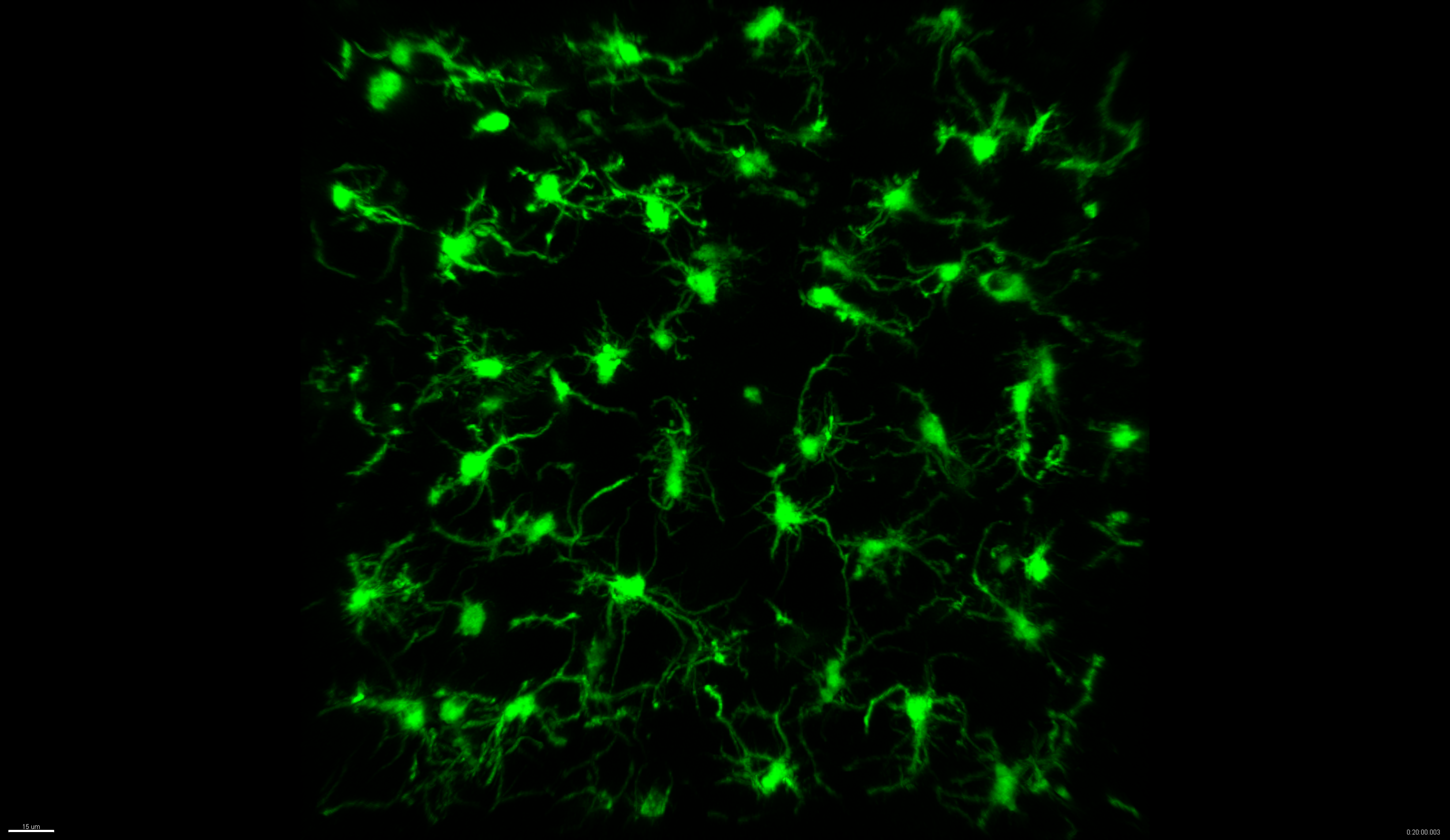

Supplement: Supplementary file 7 — Source data Fig. 2 [file 44319_2026_721_MOESM7_ESM.zip › 2B/KO/Overview/20min_arp-gfp_acsf_noTTX_121022_sl10023_2025-09-09T10-12-23.619.tif]

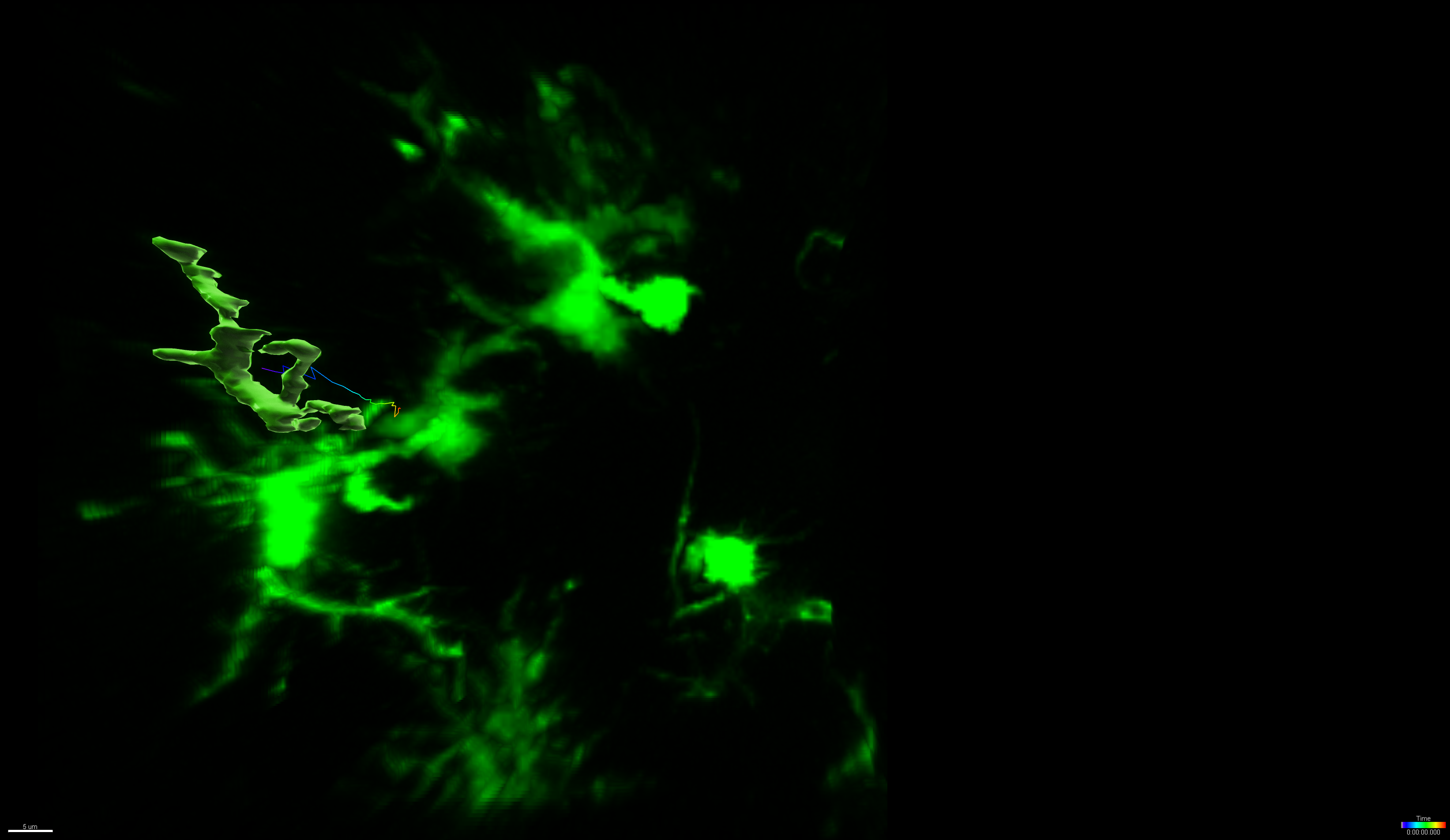

Supplement: Supplementary file 7 — Source data Fig. 2 [file 44319_2026_721_MOESM7_ESM.zip › 2B/KO/Zoom-in/0min/GFP-surface.tif]

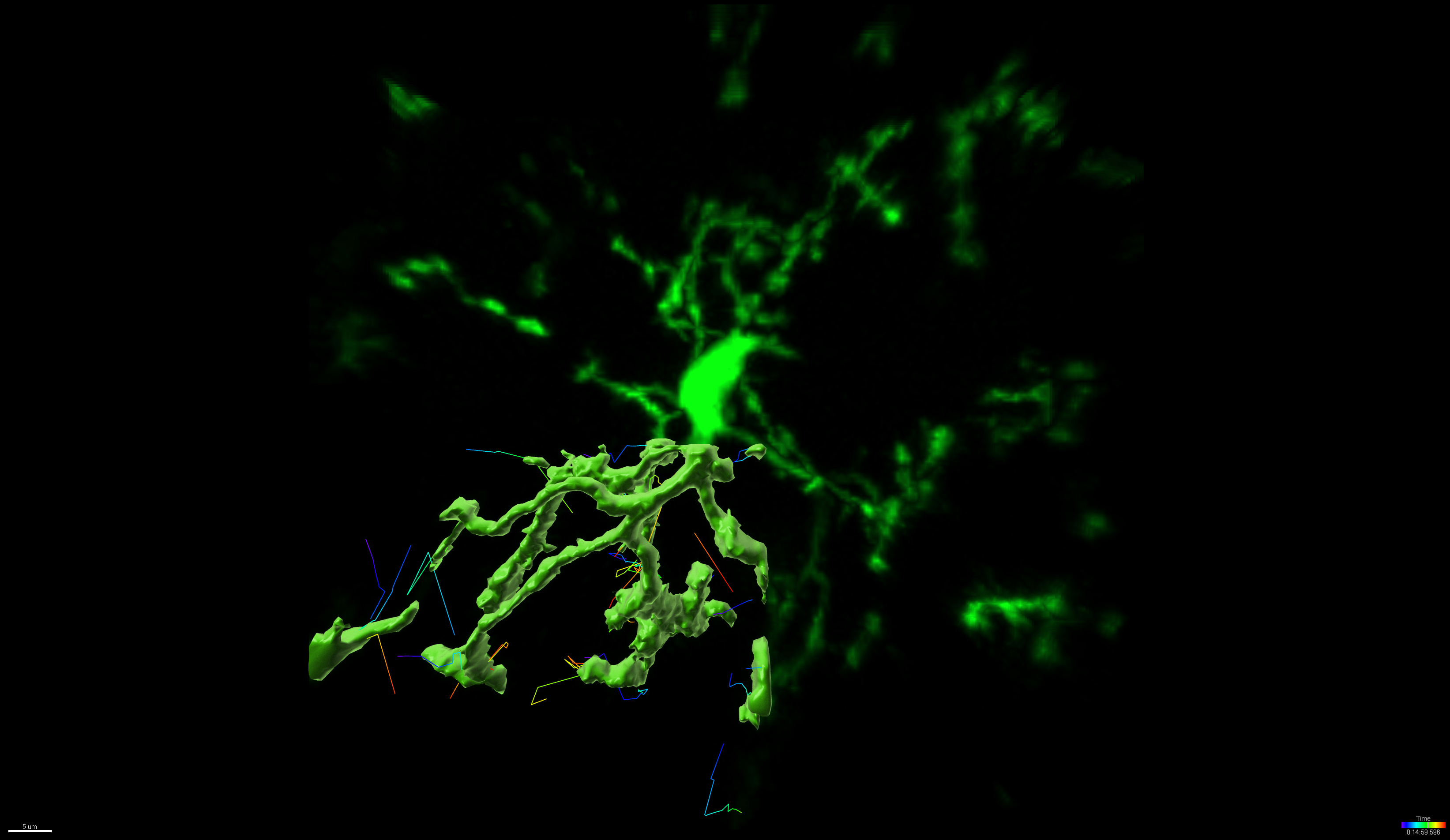

Supplement: Supplementary file 7 — Source data Fig. 2 [file 44319_2026_721_MOESM7_ESM.zip › 2A/Control/Zoom-in/15min/GFP-surface.tif]

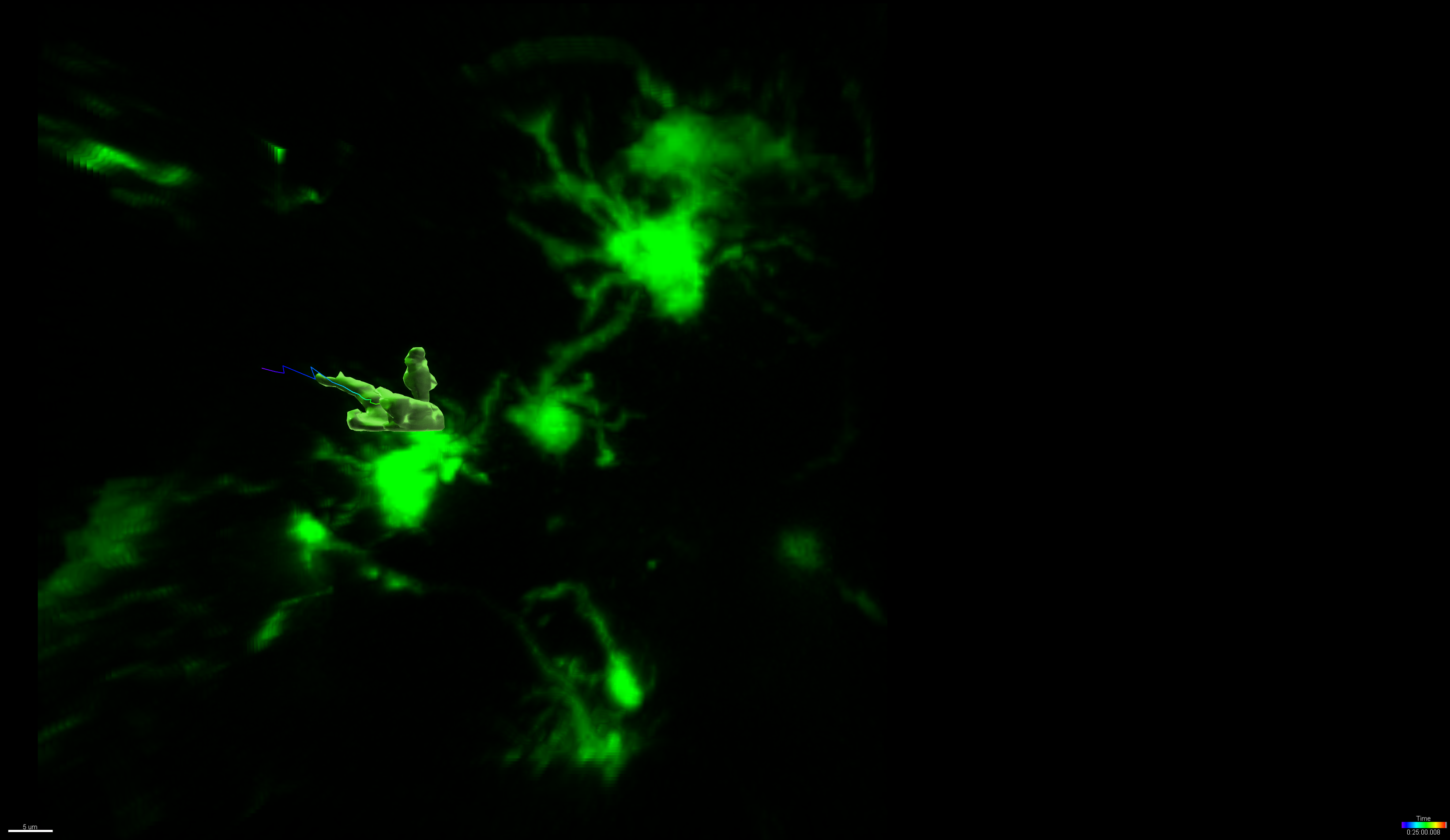

Supplement: Supplementary file 7 — Source data Fig. 2 [file 44319_2026_721_MOESM7_ESM.zip › 2B/KO/Zoom-in/25min/GFP-surface.tif]

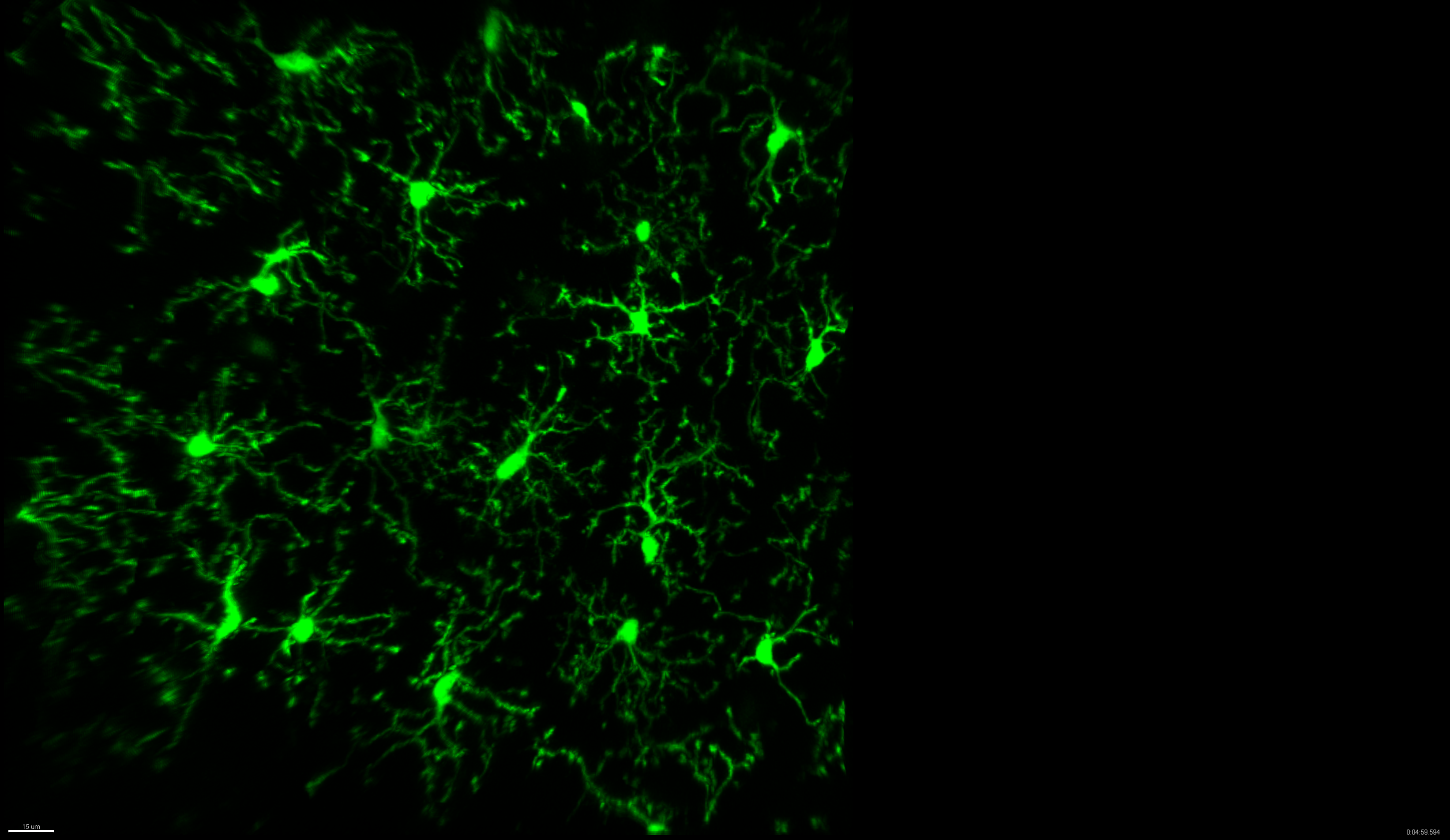

Supplement: Supplementary file 7 — Source data Fig. 2 [file 44319_2026_721_MOESM7_ESM.zip › 2A/Control/Overview/5min_controlarp-gfp_LAE3059_101022_sl1001_2025-09-09T09-59-17.560.tif]

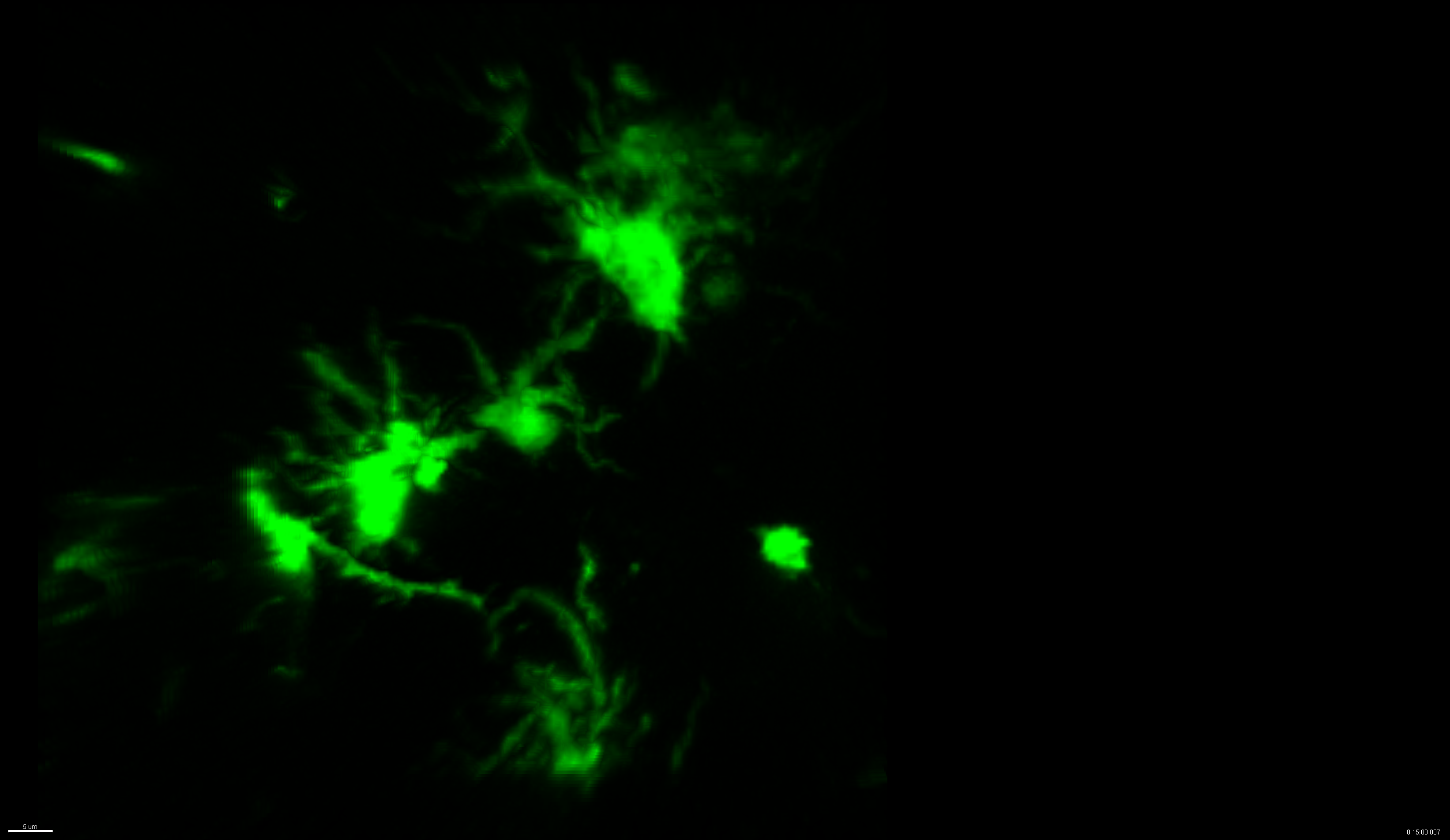

Supplement: Supplementary file 7 — Source data Fig. 2 [file 44319_2026_721_MOESM7_ESM.zip › 2B/KO/Zoom-in/15min/GFP-original.tif]

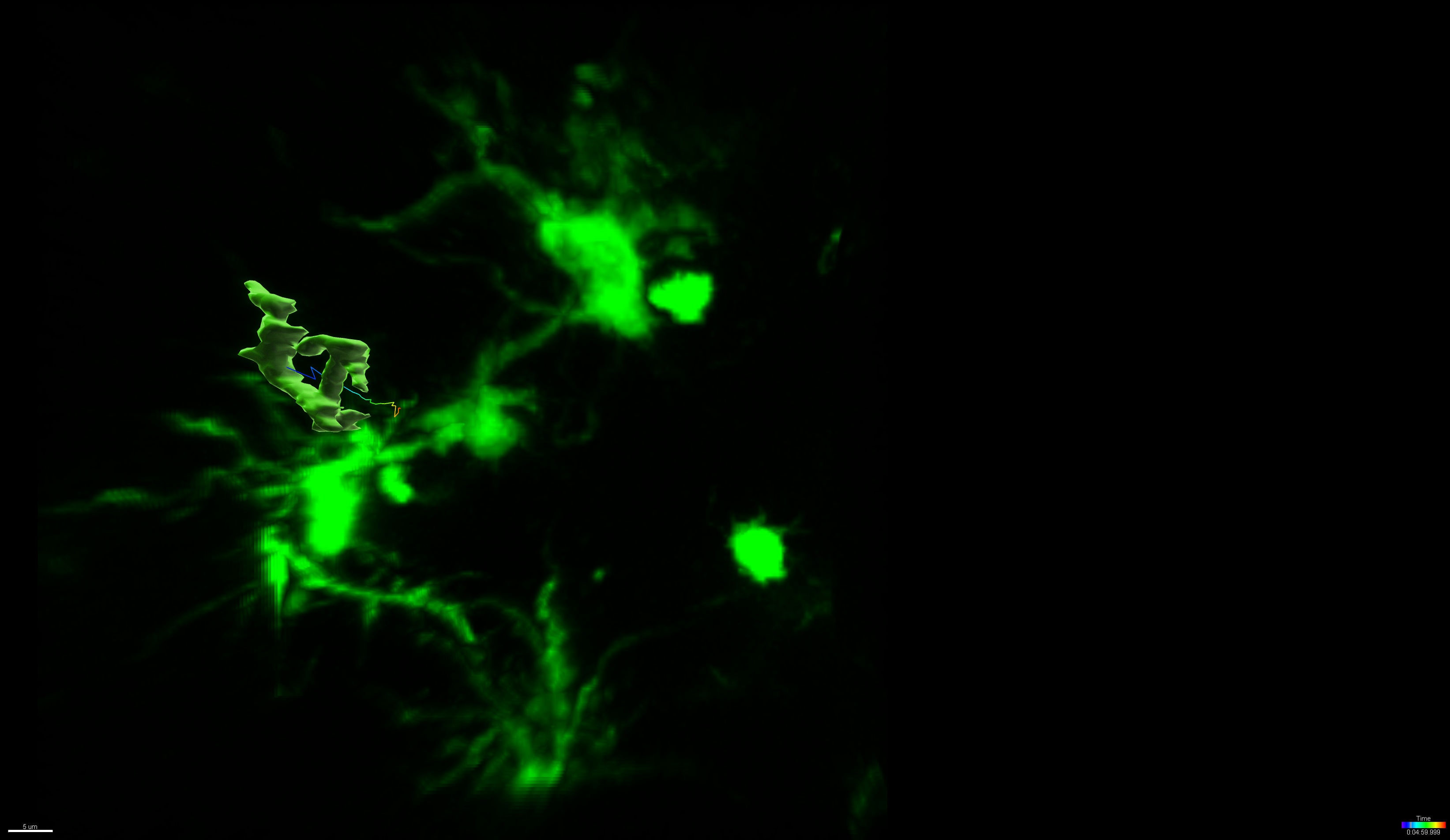

Supplement: Supplementary file 7 — Source data Fig. 2 [file 44319_2026_721_MOESM7_ESM.zip › 2B/KO/Zoom-in/5min/GFP-surface.tif]

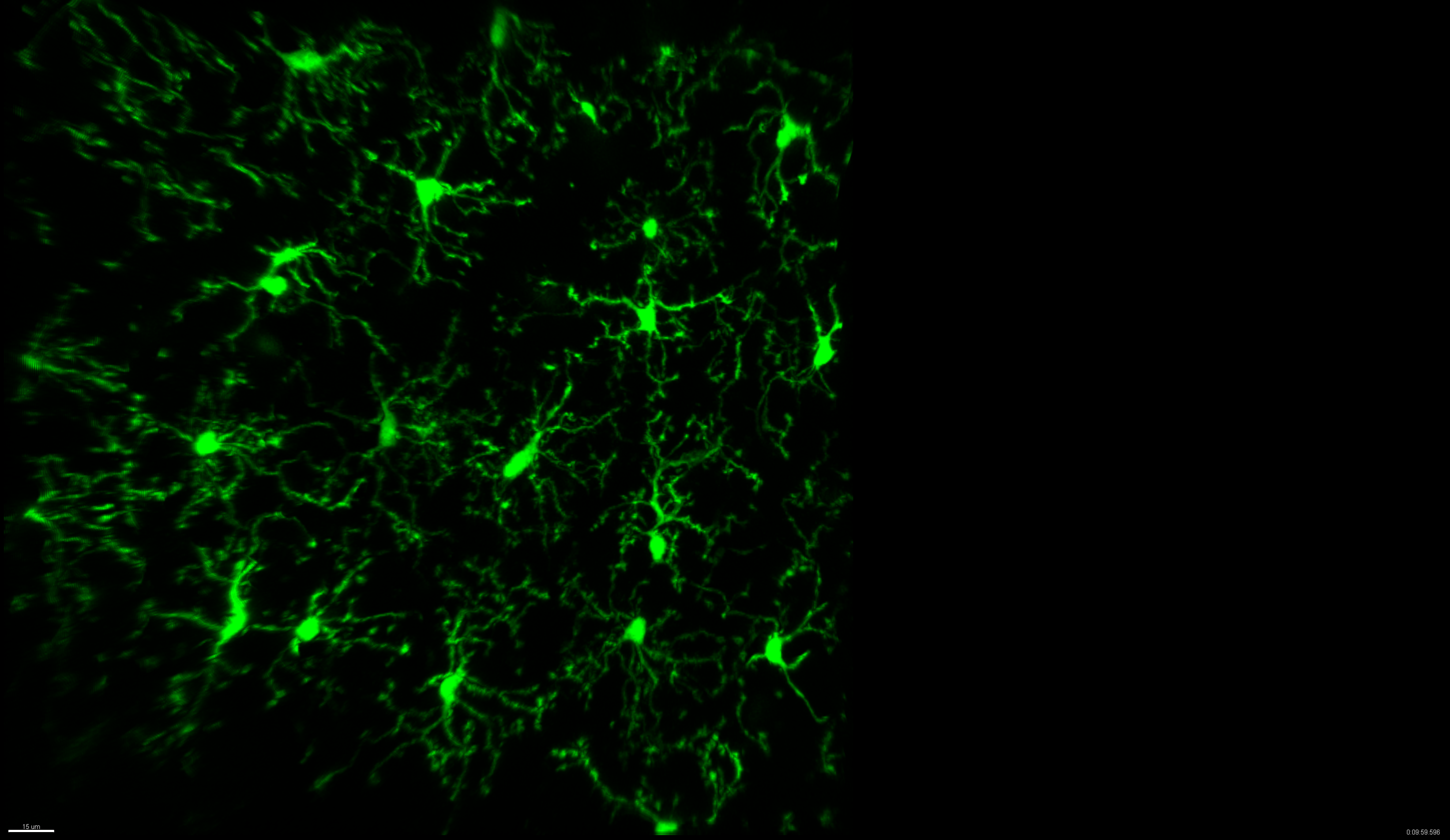

Supplement: Supplementary file 7 — Source data Fig. 2 [file 44319_2026_721_MOESM7_ESM.zip › 2A/Control/Overview/10min_controlarp-gfp_LAE3059_101022_sl1001_2025-09-09T09-59.tif]

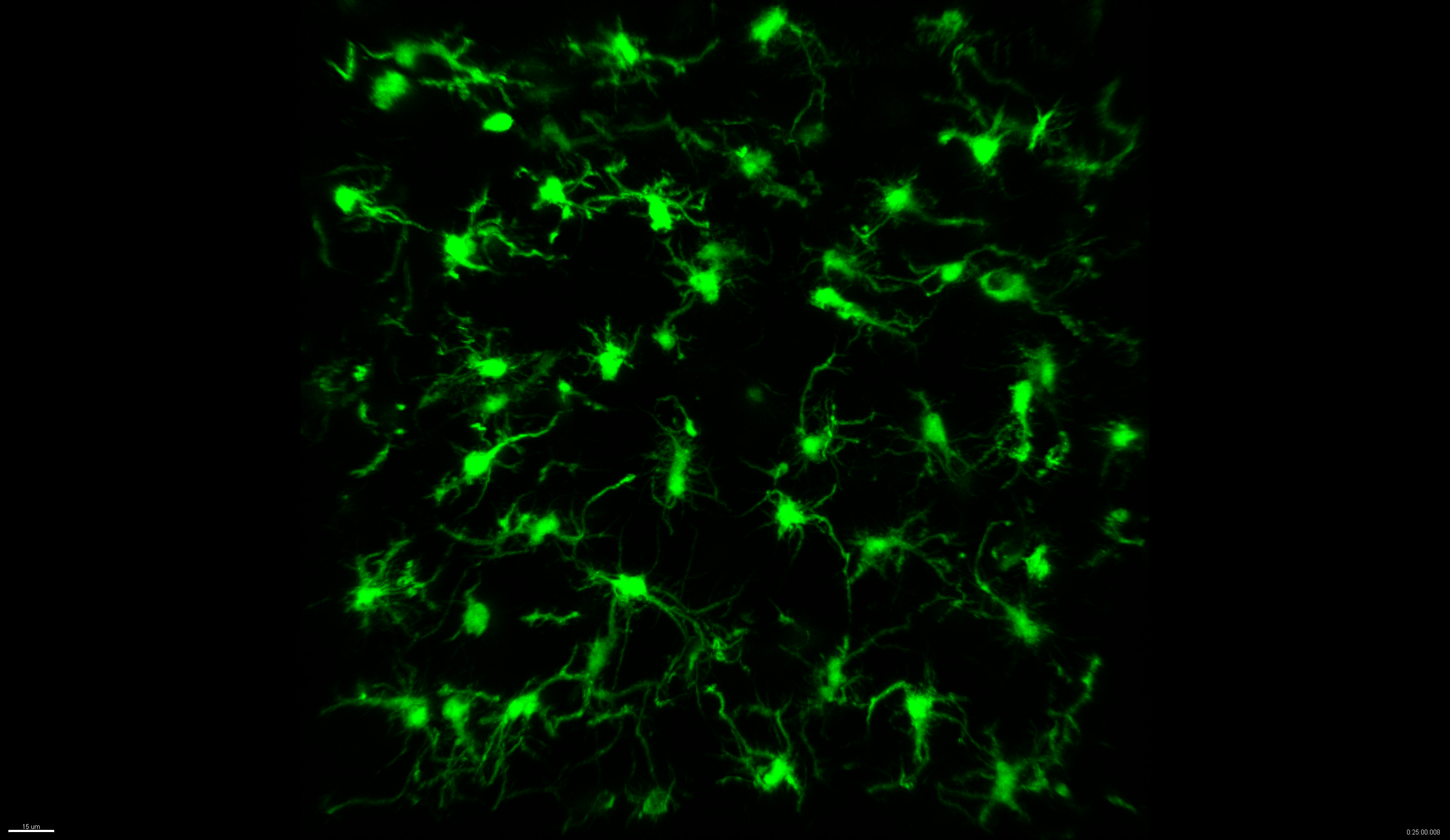

Supplement: Supplementary file 7 — Source data Fig. 2 [file 44319_2026_721_MOESM7_ESM.zip › 2B/KO/Overview/25min_arp-gfp_acsf_noTTX_121022_sl10023_2025-09-09T10-12-29.922.tif]

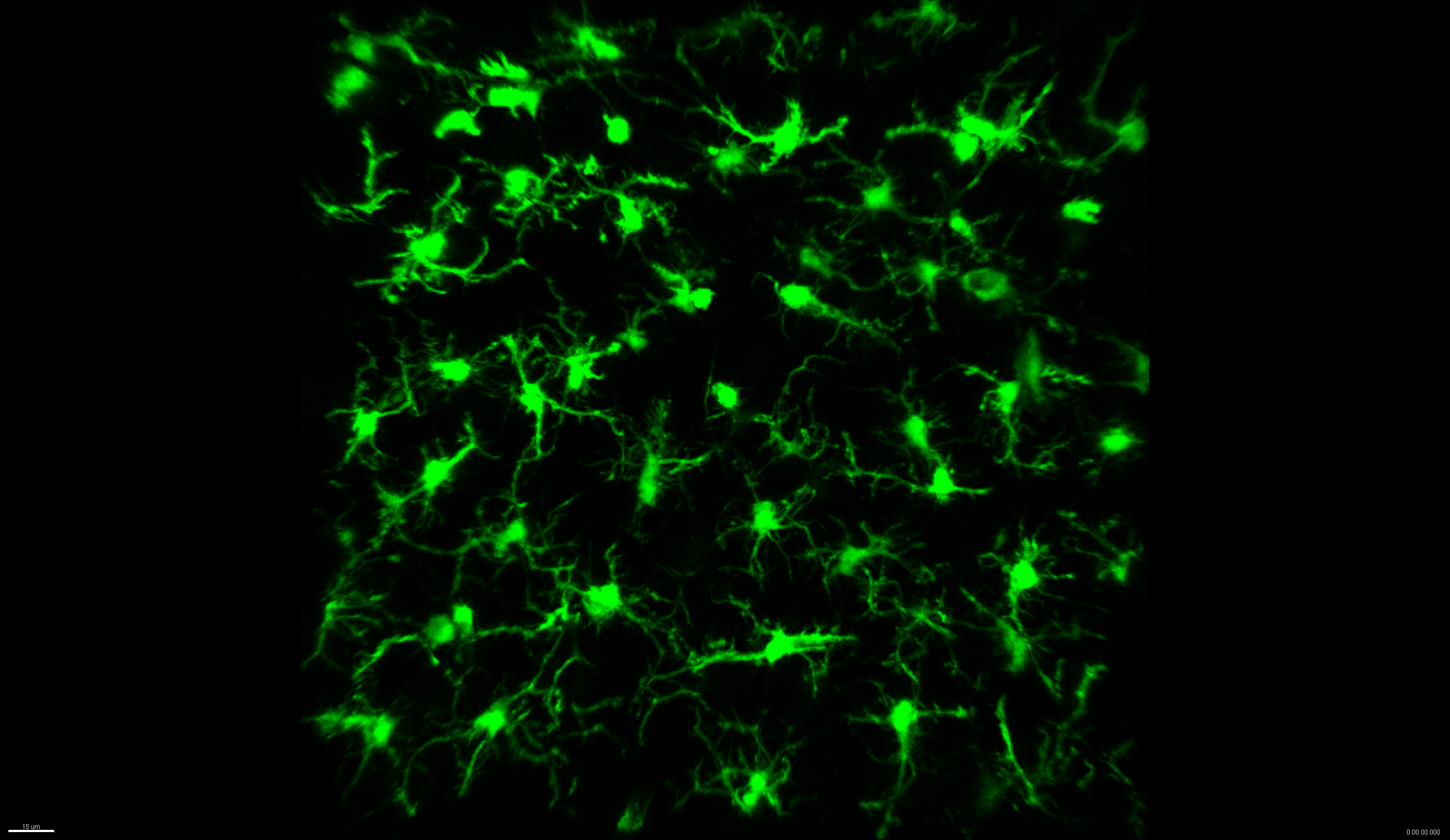

Supplement: Supplementary file 7 — Source data Fig. 2 [file 44319_2026_721_MOESM7_ESM.zip › 2B/KO/Overview/0min_arp-gfp_acsf_noTTX_121022_sl10023_2025-09-09T10-12-08.352.tif]

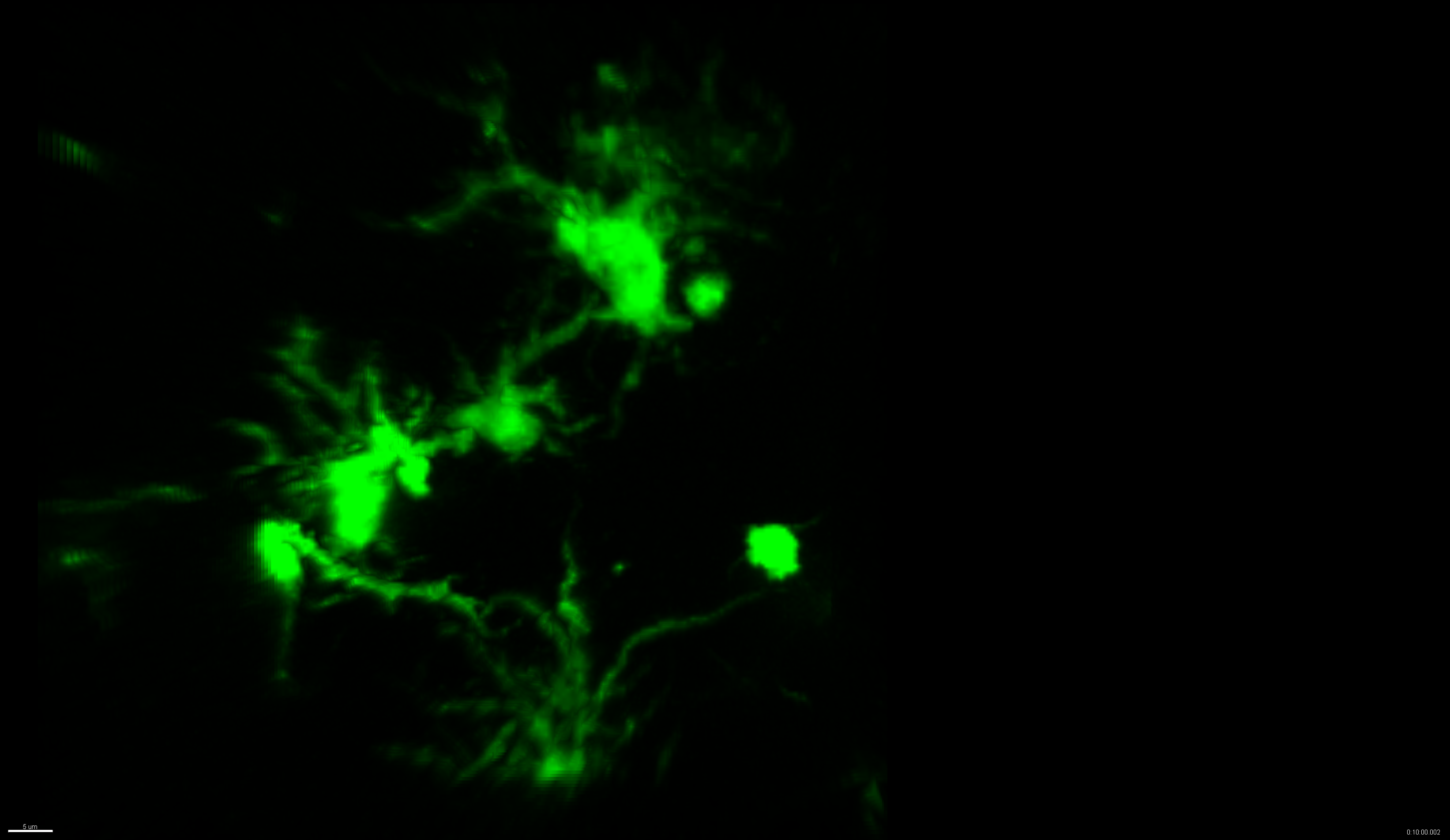

Supplement: Supplementary file 7 — Source data Fig. 2 [file 44319_2026_721_MOESM7_ESM.zip › 2B/KO/Zoom-in/10min/GFP-original.tif]

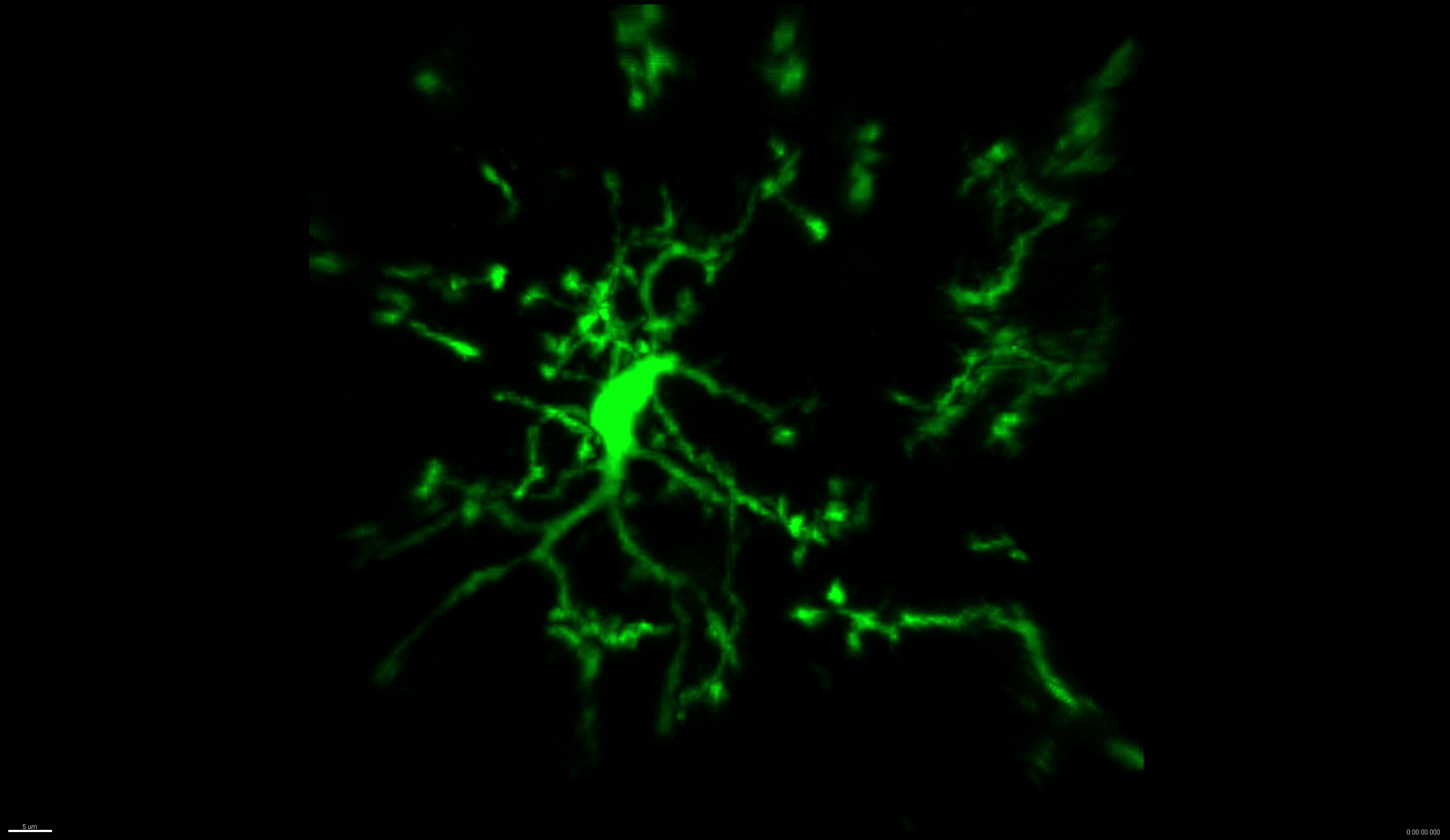

Supplement: Supplementary file 7 — Source data Fig. 2 [file 44319_2026_721_MOESM7_ESM.zip › 2A/Control/Zoom-in/0min/GFP-original.tif]

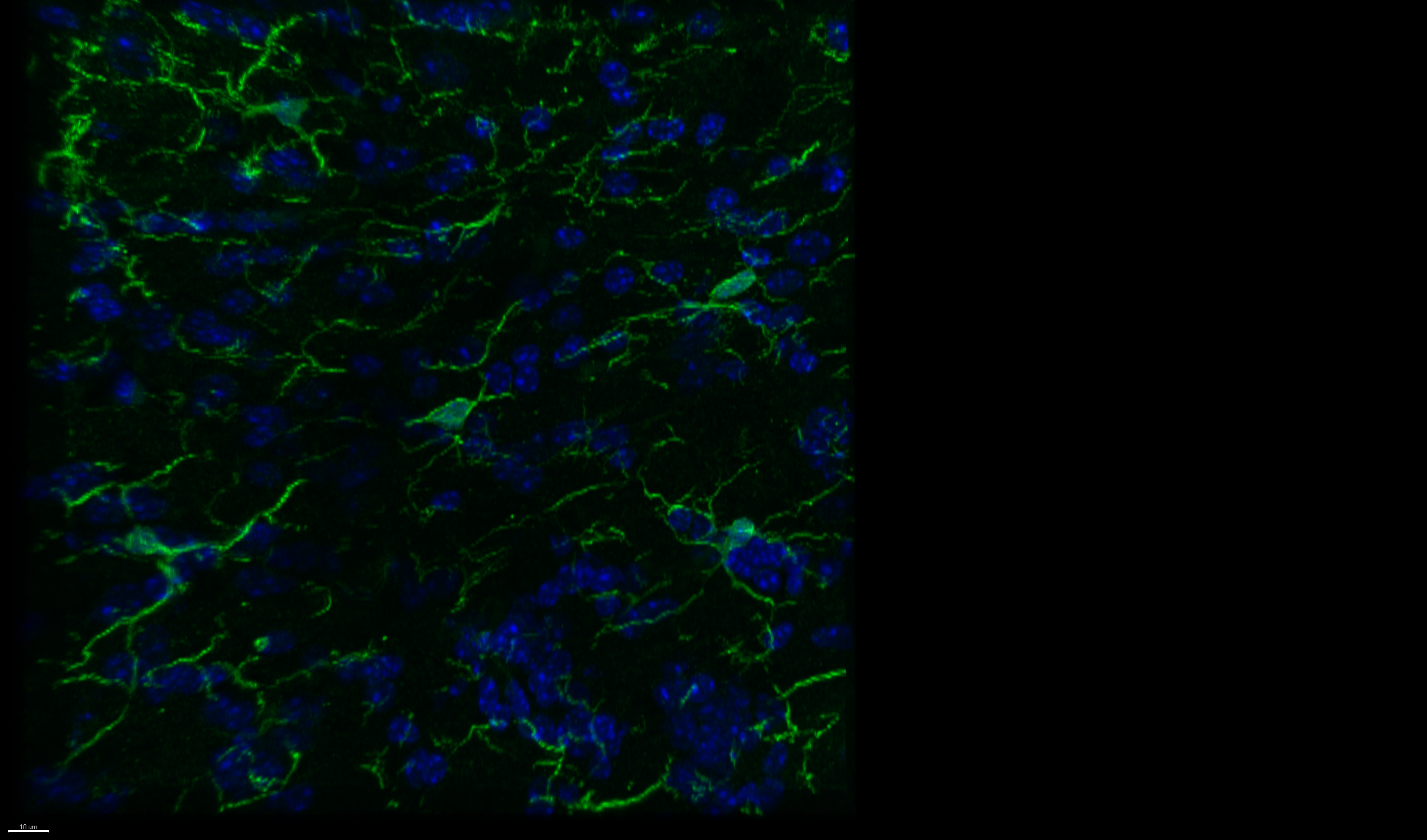

Supplement: Supplementary file 8 — Source data Fig. 3 [file 44319_2026_721_MOESM8_ESM.zip › 3G/Galectin3-CC-Ctrl/IBA1-58-CC.tif]

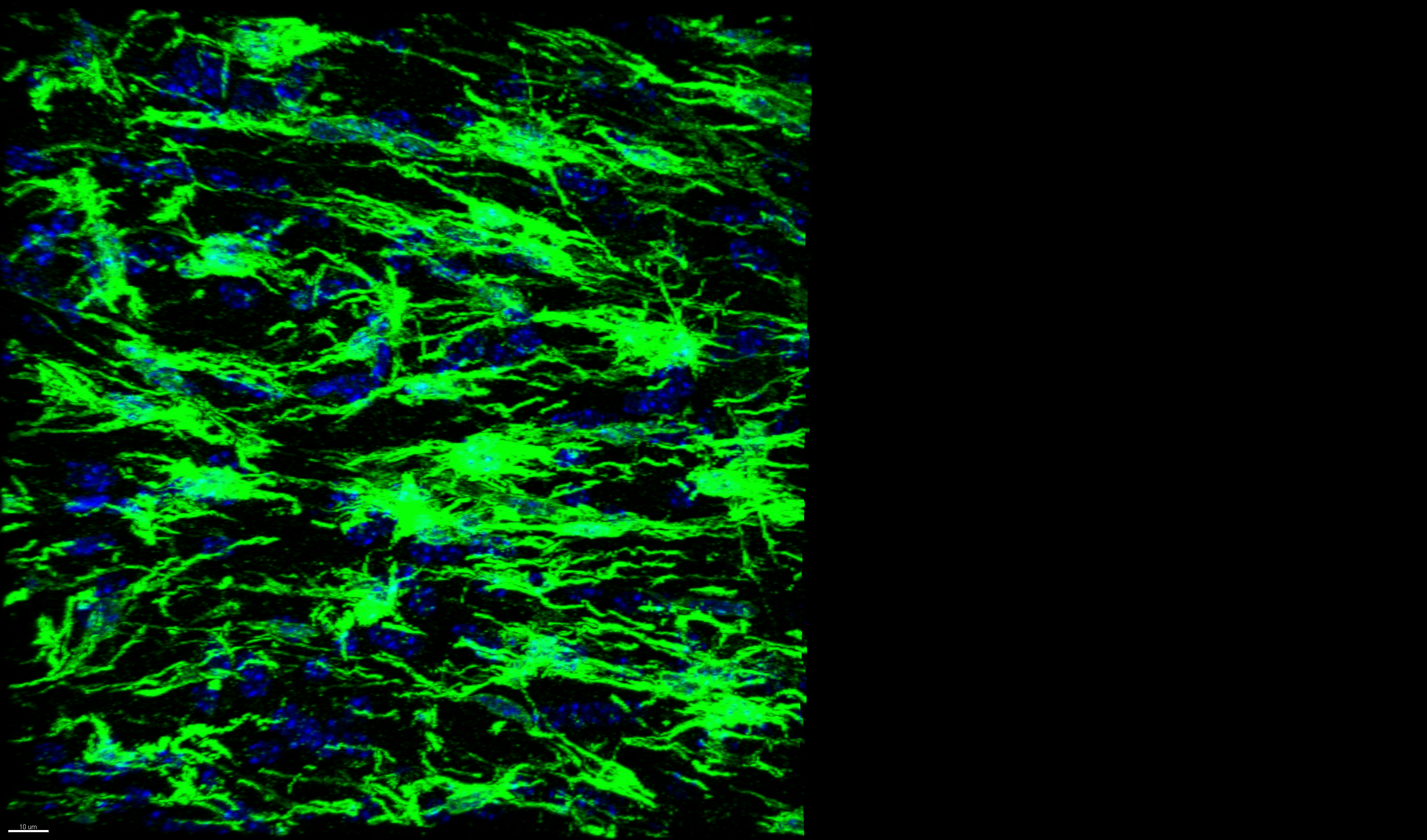

Supplement: Supplementary file 8 — Source data Fig. 3 [file 44319_2026_721_MOESM8_ESM.zip › 3G/Galectin3-CC-KO/IBA1-Image 14-.tif]

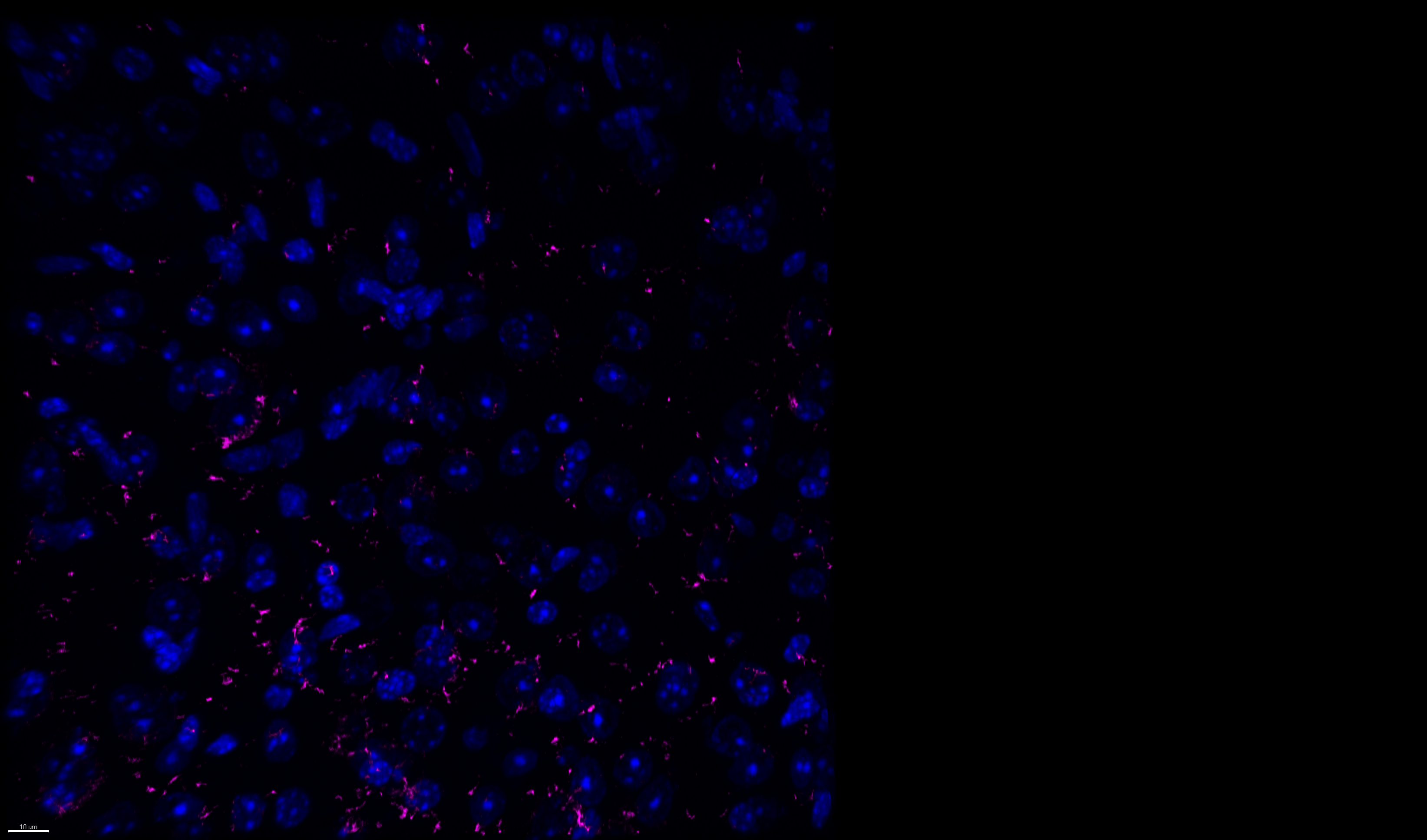

Supplement: Supplementary file 8 — Source data Fig. 3 [file 44319_2026_721_MOESM8_ESM.zip › 3G/Galectin3-Cortex-Ctrl/Galectin3-58-Ctx-.tif]

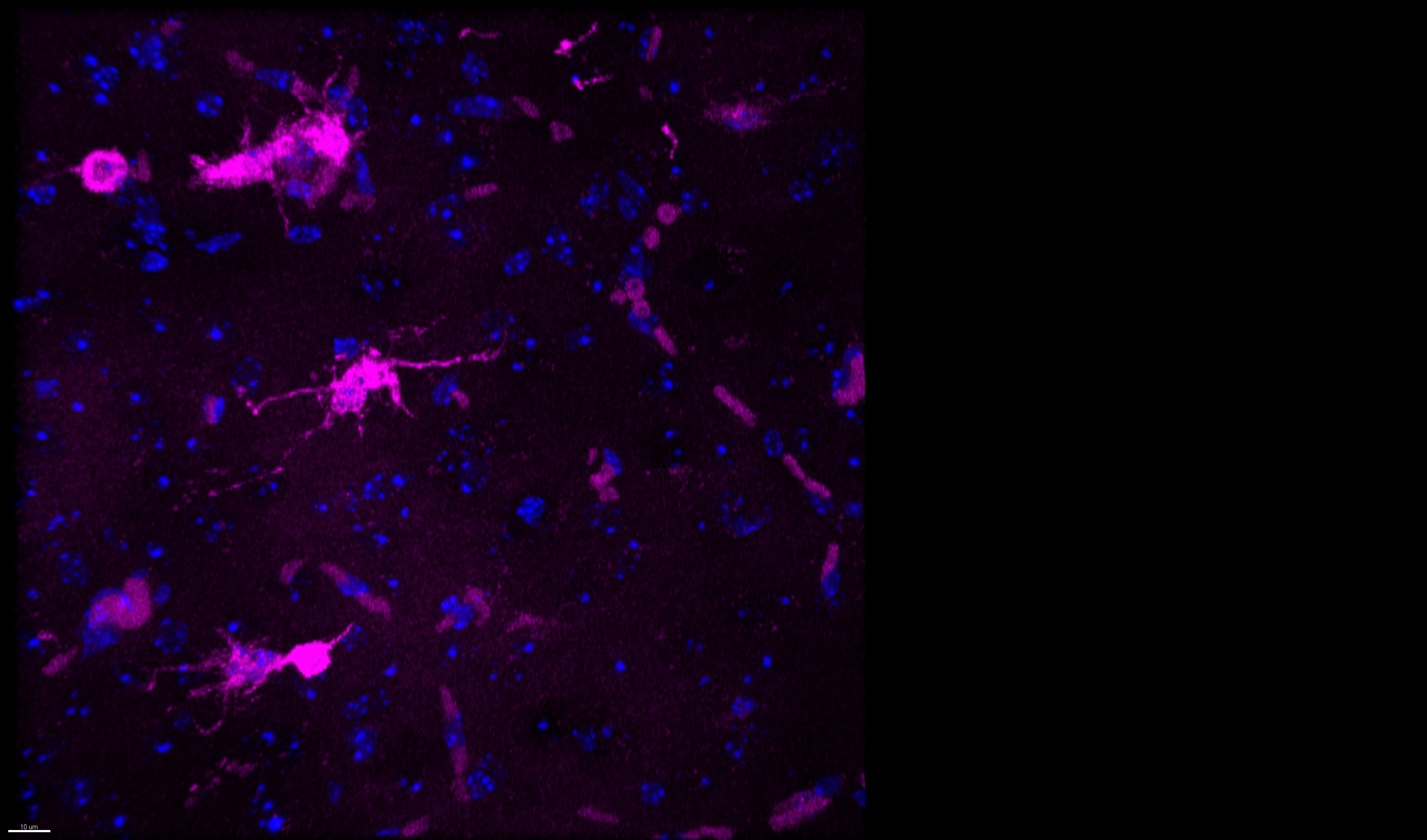

Supplement: Supplementary file 8 — Source data Fig. 3 [file 44319_2026_721_MOESM8_ESM.zip › 3G/Galectin3-Cortex-KO/Galectin3-Image 16.tif]

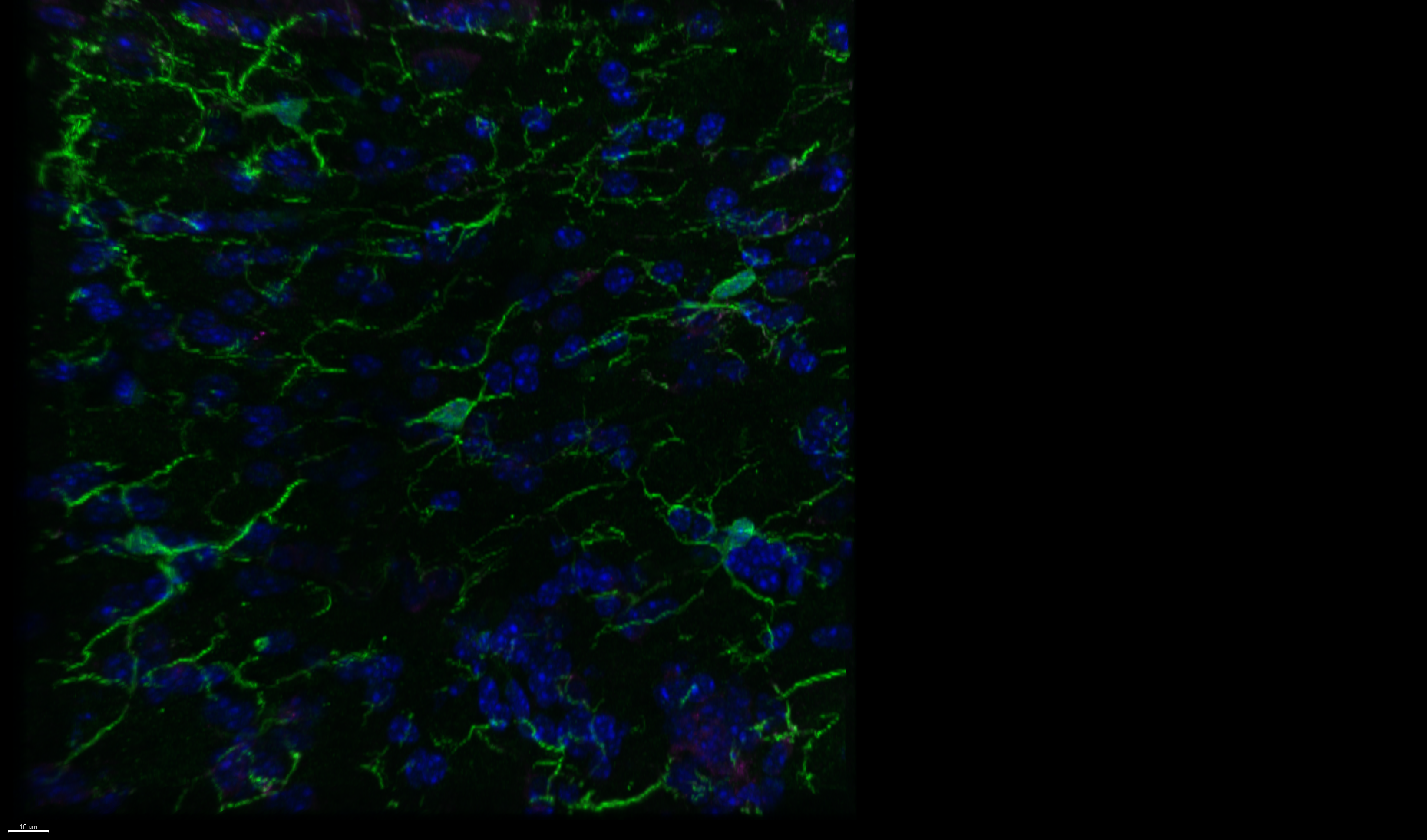

Supplement: Supplementary file 8 — Source data Fig. 3 [file 44319_2026_721_MOESM8_ESM.zip › 3G/Galectin3-CC-Ctrl/Iba1-Gal3-58-CC-.tif]

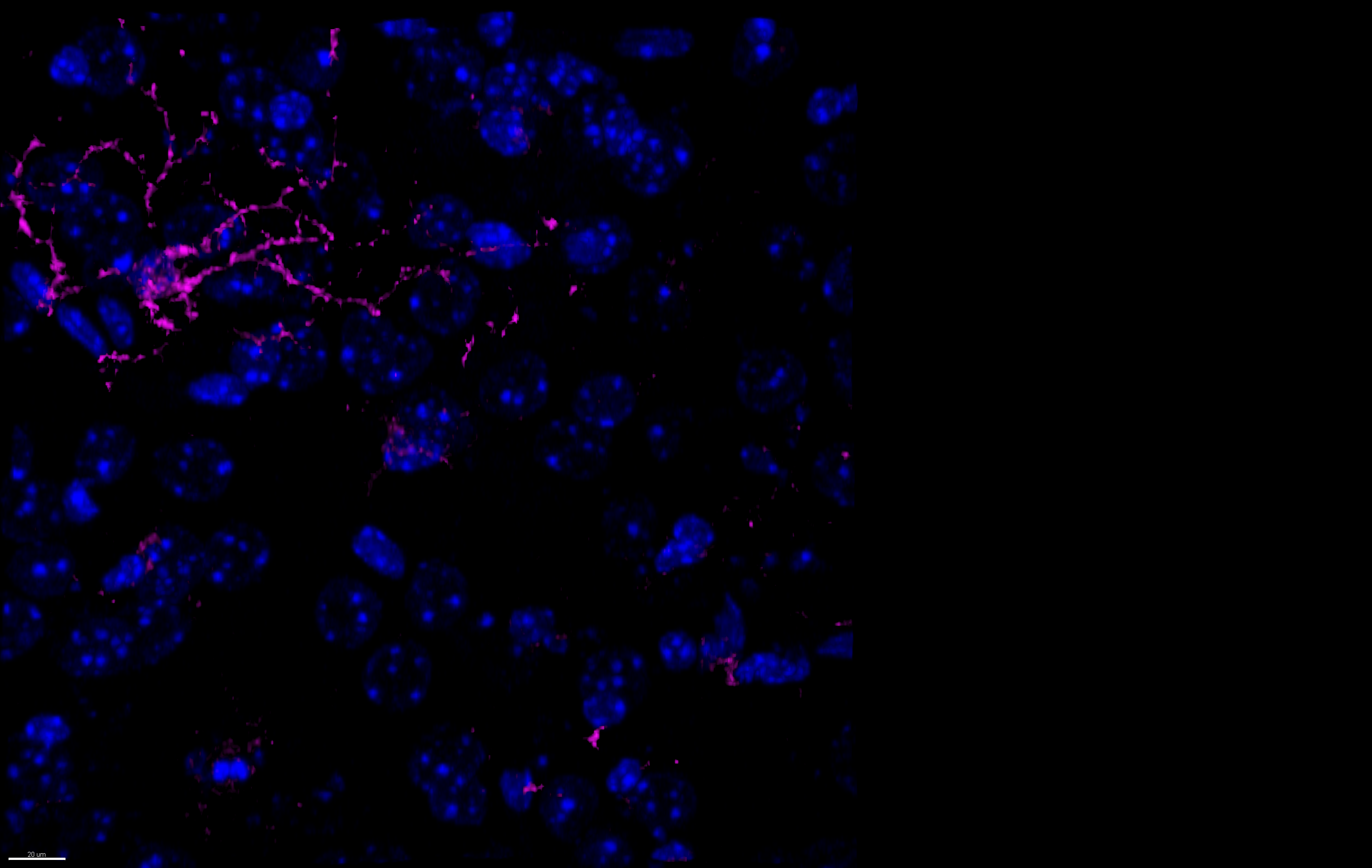

Supplement: Supplementary file 8 — Source data Fig. 3 [file 44319_2026_721_MOESM8_ESM.zip › 3B/CD11b-KO/CD11b masked.tif]

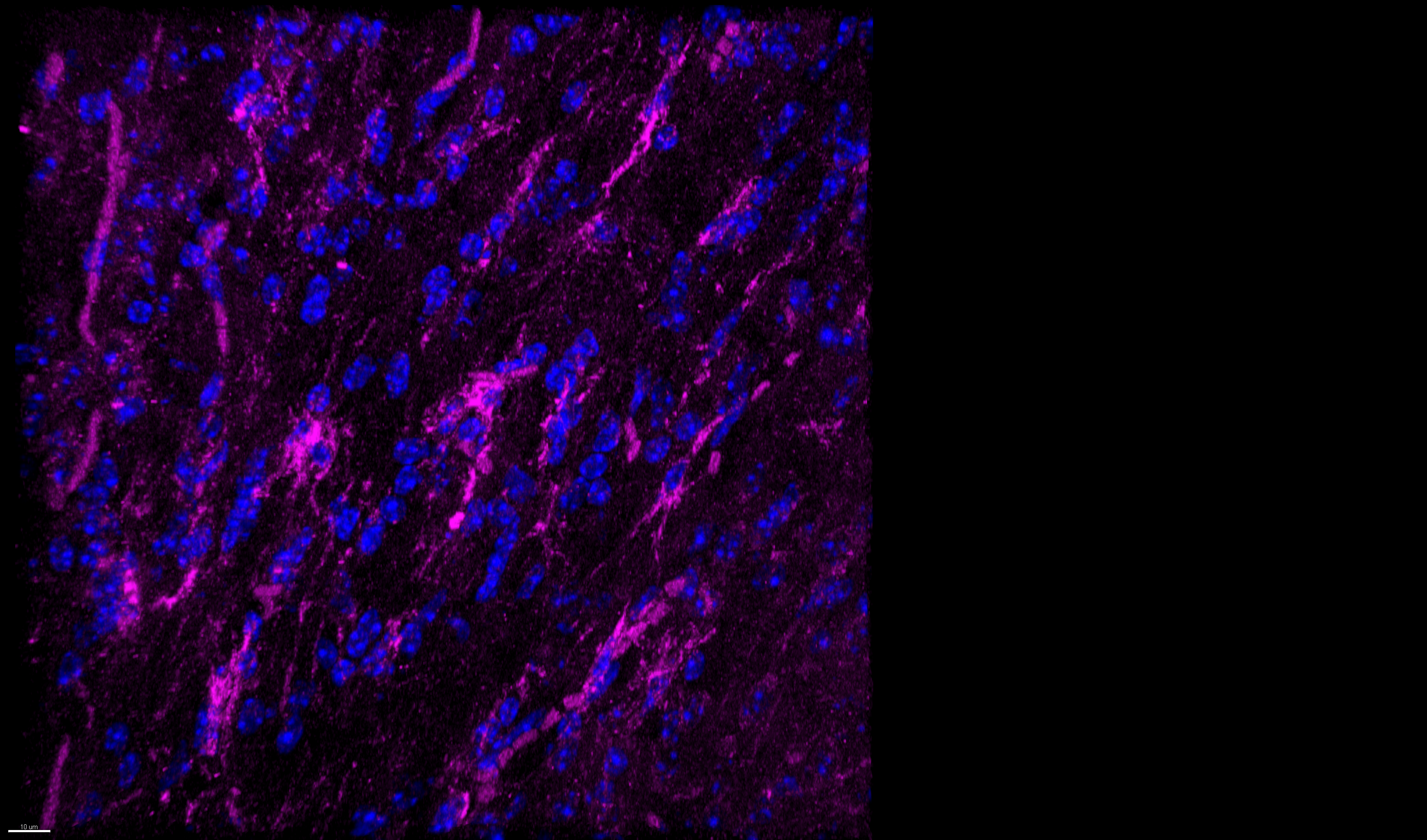

Supplement: Supplementary file 8 — Source data Fig. 3 [file 44319_2026_721_MOESM8_ESM.zip › 3G/AXL-CC-KO/AXLoriginal_Image 6.tif]

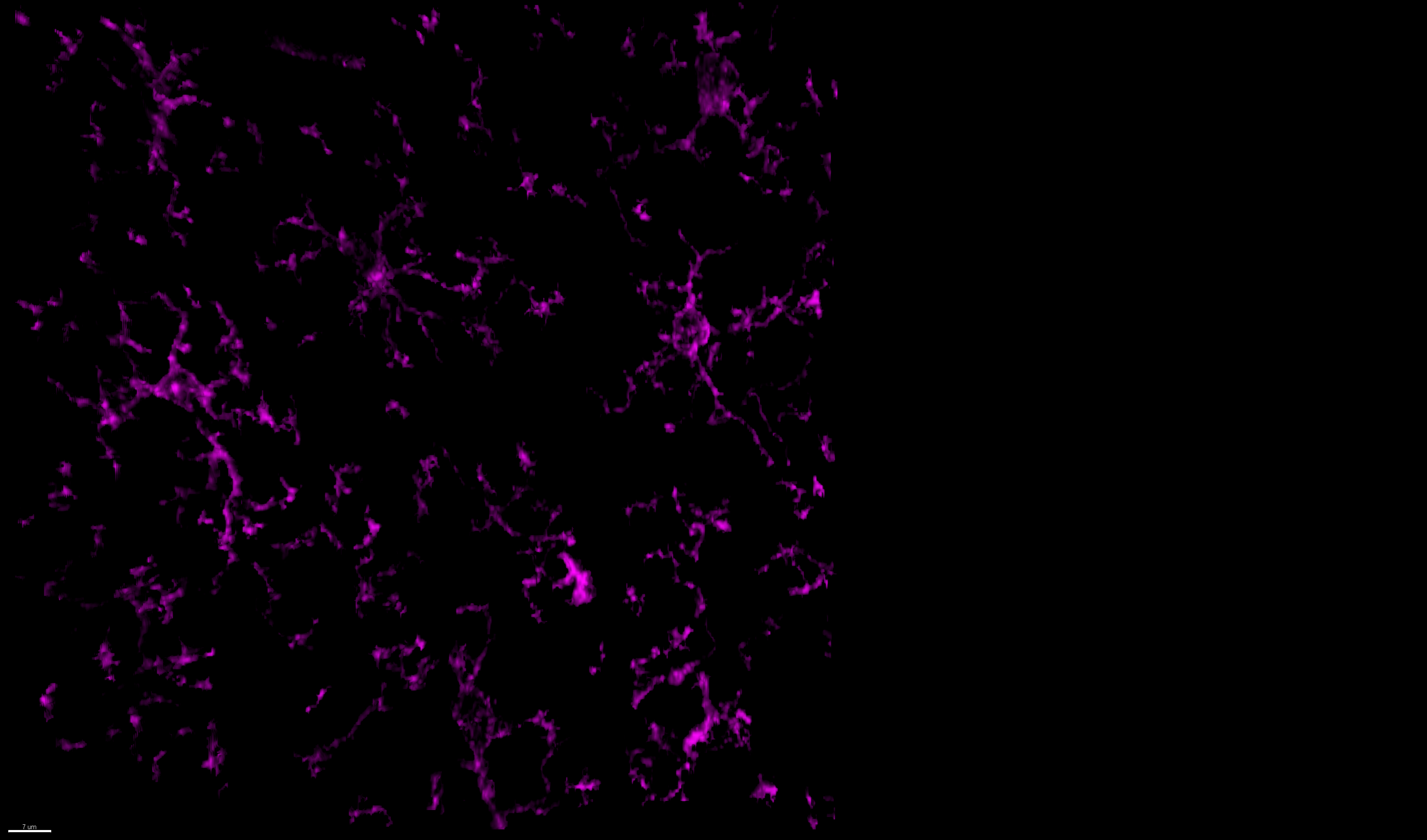

Supplement: Supplementary file 8 — Source data Fig. 3 [file 44319_2026_721_MOESM8_ESM.zip › 3B/CD11b-Ctrl/CD11b masked.tif]

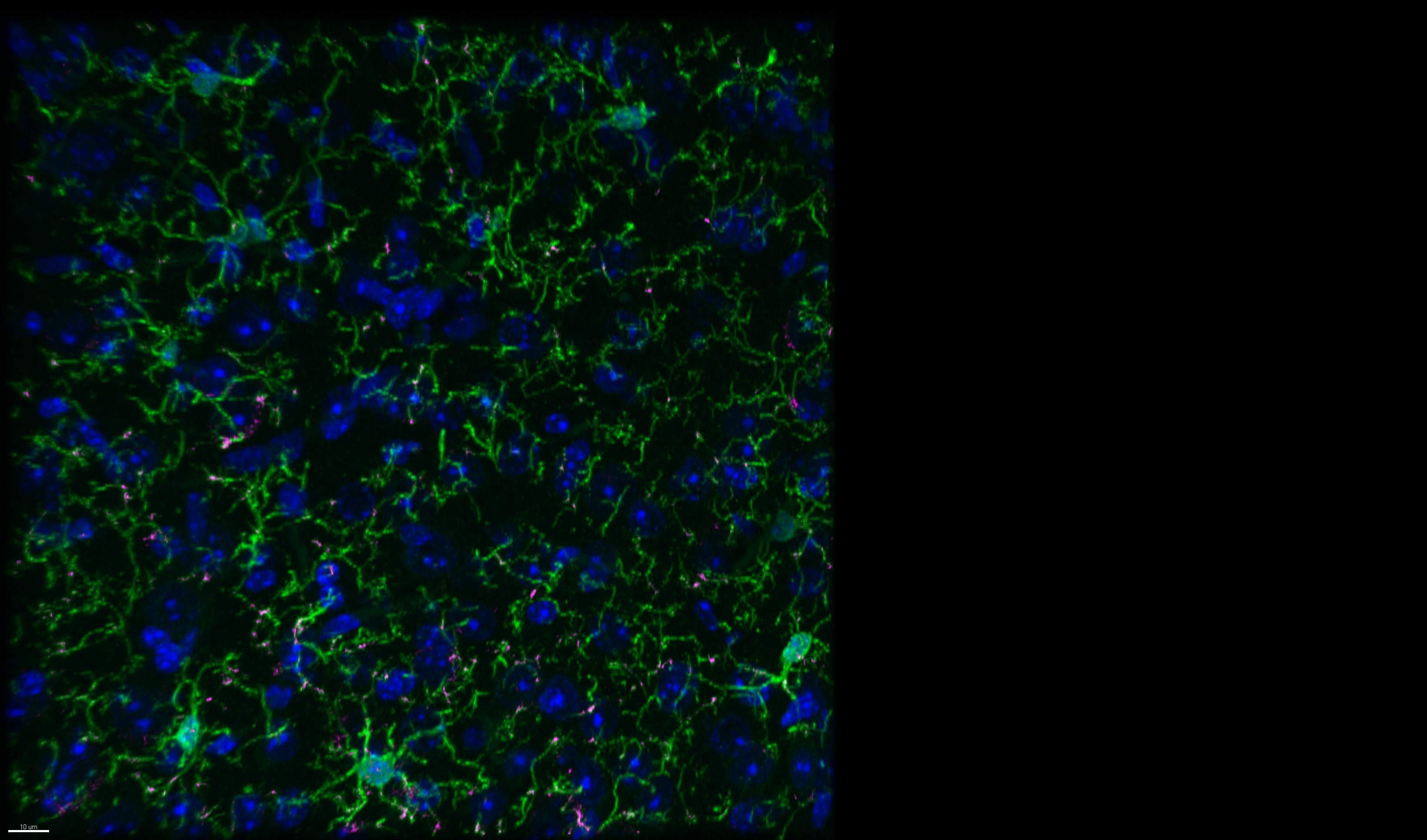

Supplement: Supplementary file 8 — Source data Fig. 3 [file 44319_2026_721_MOESM8_ESM.zip › 3G/Galectin3-Cortex-Ctrl/Iba1-Gal3-58-Ctx-.tif]

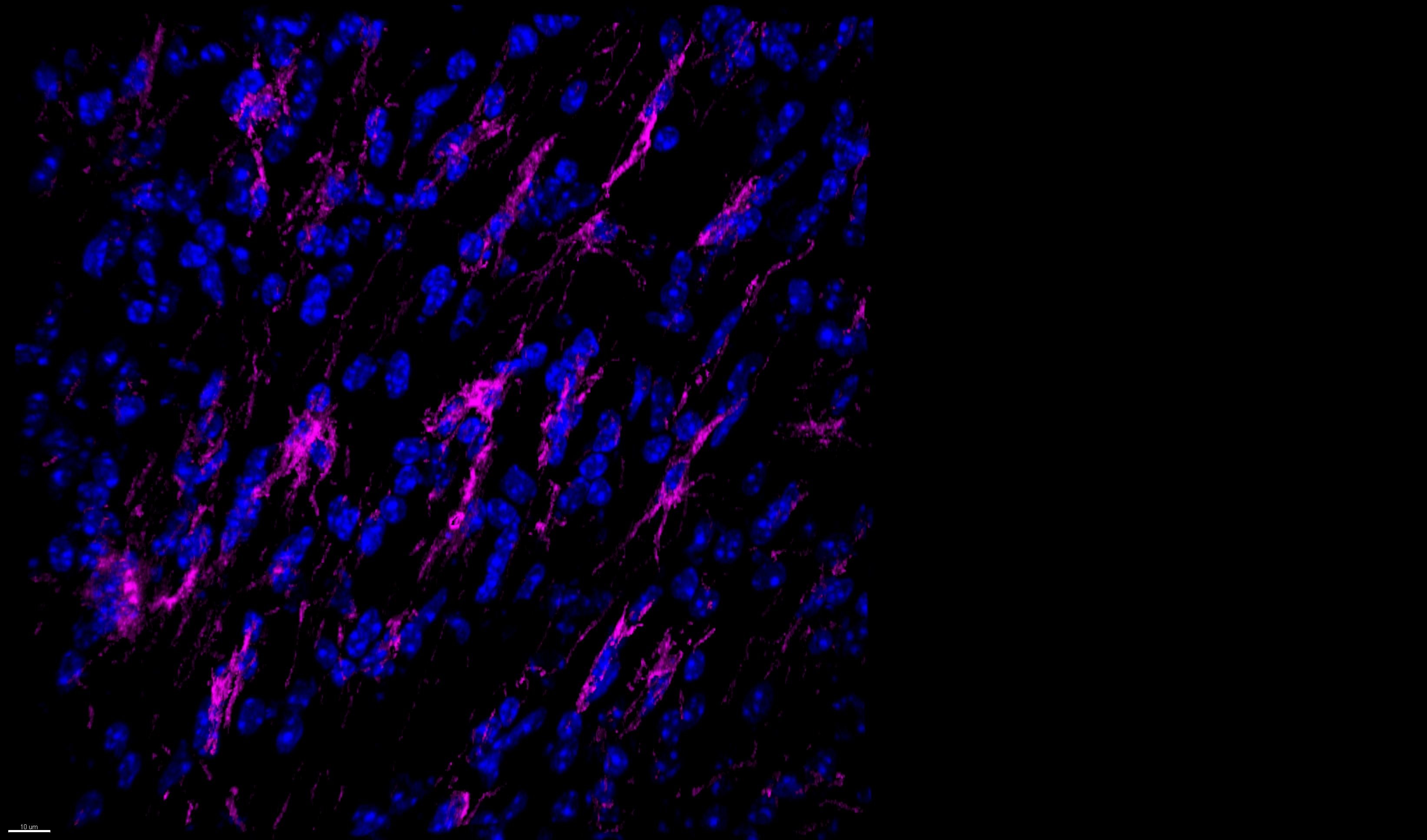

Supplement: Supplementary file 8 — Source data Fig. 3 [file 44319_2026_721_MOESM8_ESM.zip › 3G/AXL-CC-KO/AXL Masked_Image 6.tif]

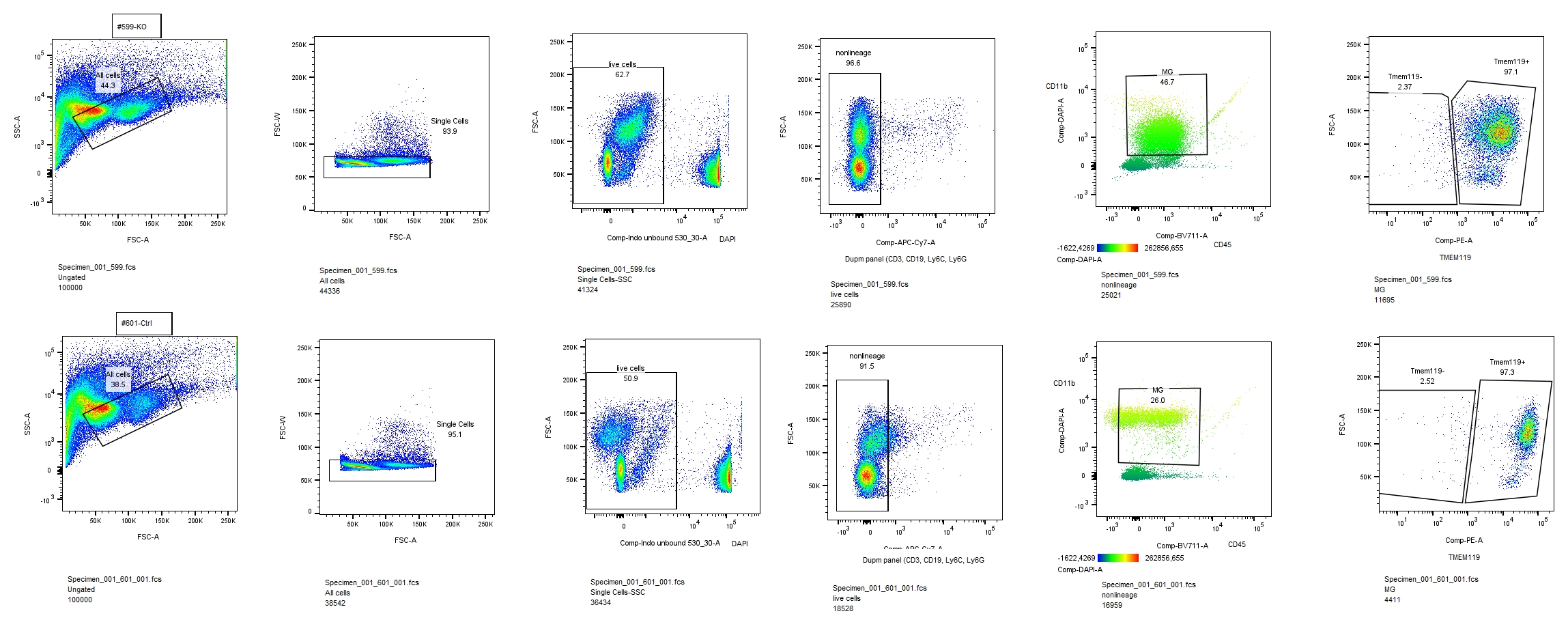

Supplement: Supplementary file 8 — Source data Fig. 3 [file 44319_2026_721_MOESM8_ESM.zip › 3I/Gating strategyTMEM119 flow cytometry.jpg]

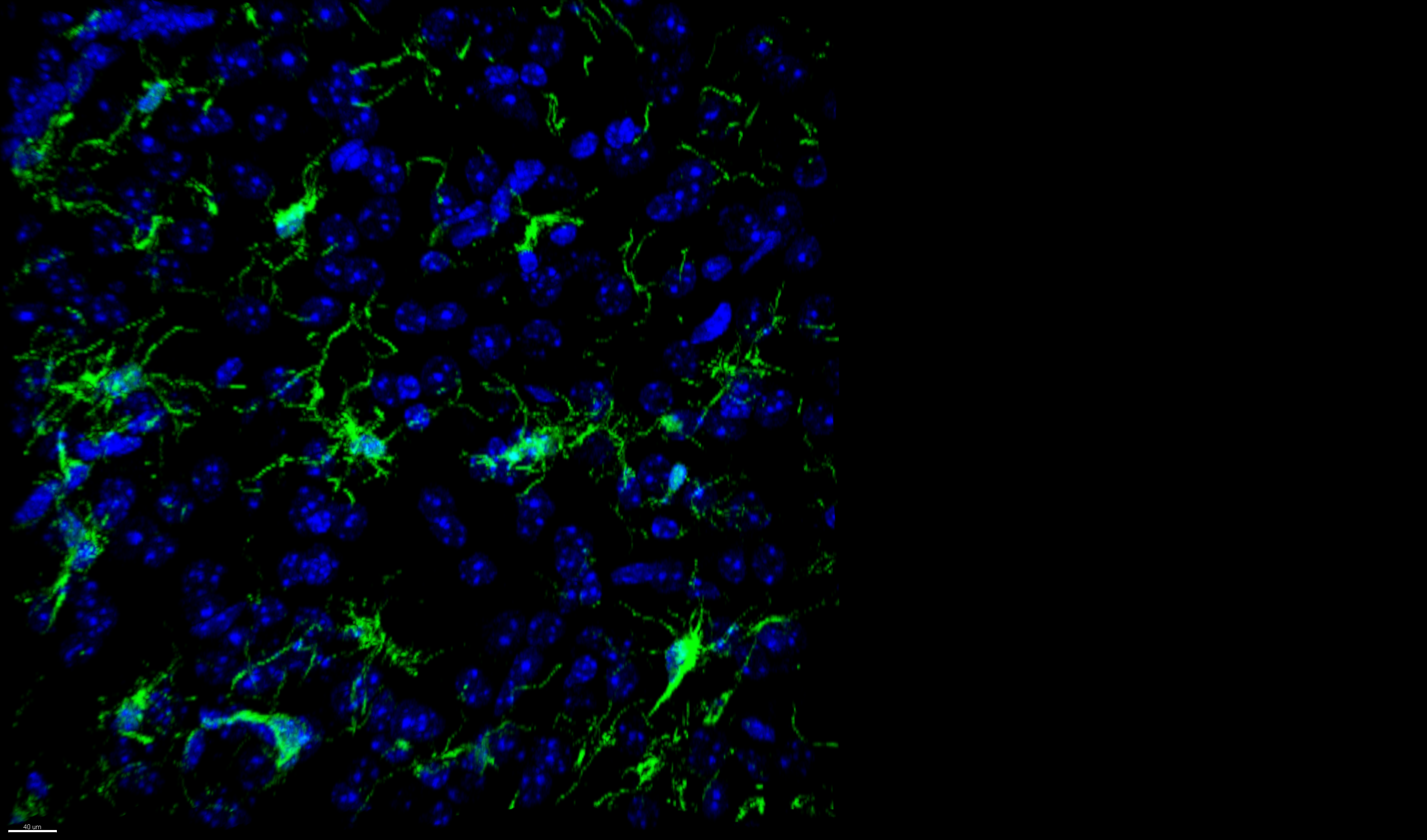

Supplement: Supplementary file 8 — Source data Fig. 3 [file 44319_2026_721_MOESM8_ESM.zip › 3G/AXL-Cotrex-KO/IBA1-Image 1_.tif]

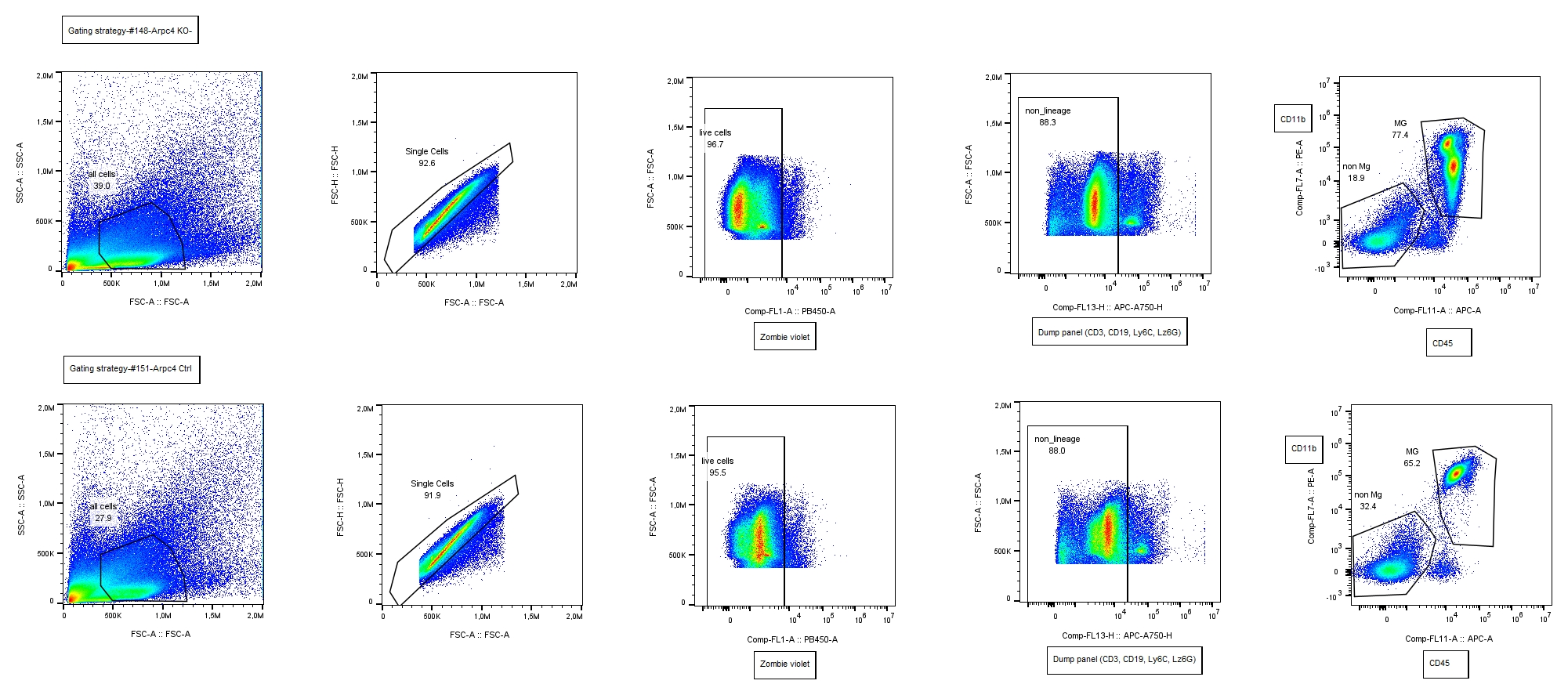

Supplement: Supplementary file 8 — Source data Fig. 3 [file 44319_2026_721_MOESM8_ESM.zip › 3A/Gating strategy-microglia-Arpc4 KO vs Ctrl.jpg]

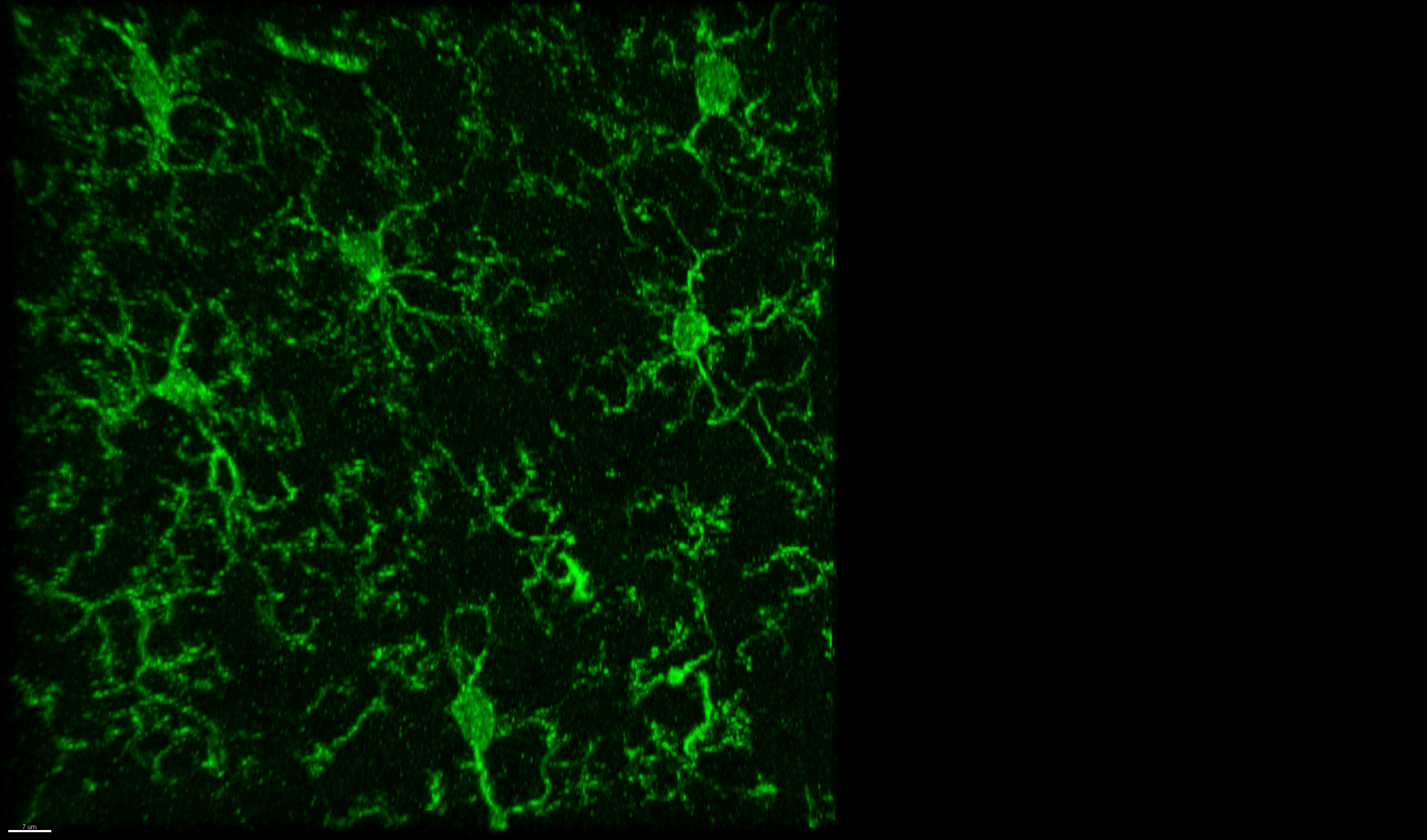

Supplement: Supplementary file 8 — Source data Fig. 3 [file 44319_2026_721_MOESM8_ESM.zip › 3B/CD11b-Ctrl/IBA1.tif]

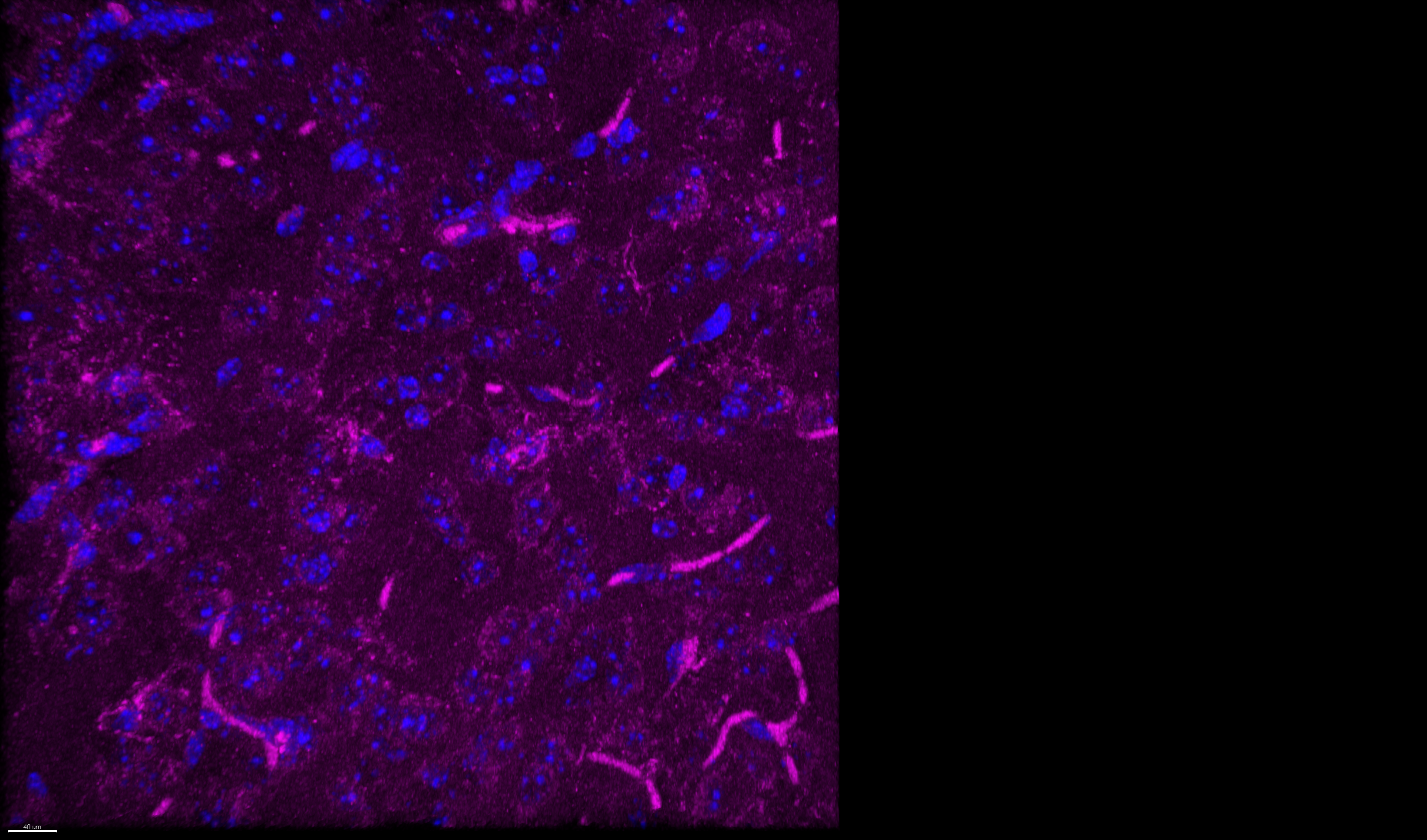

Supplement: Supplementary file 8 — Source data Fig. 3 [file 44319_2026_721_MOESM8_ESM.zip › 3G/AXL-Cotrex-KO/AXL-original_Image 1.tif]

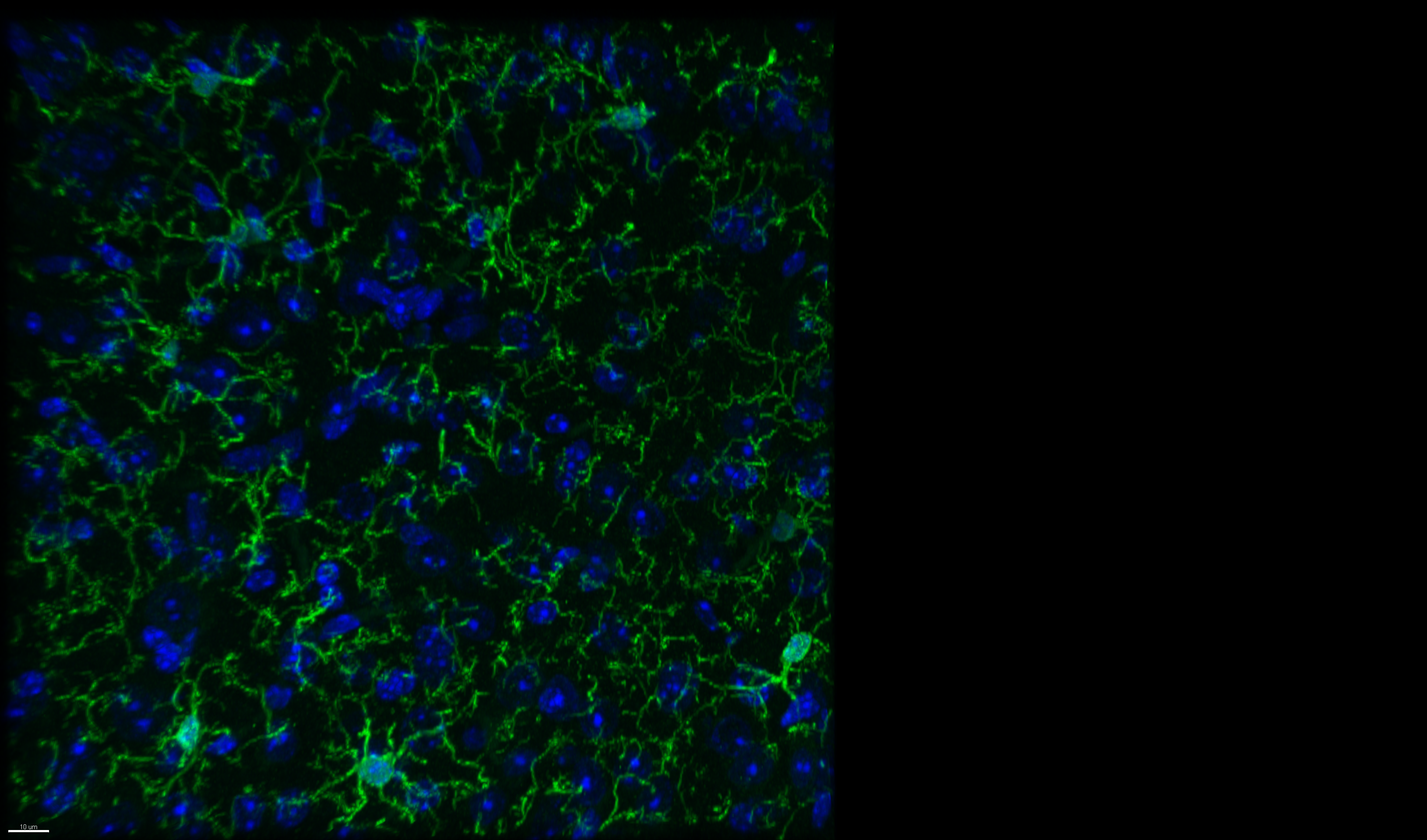

Supplement: Supplementary file 8 — Source data Fig. 3 [file 44319_2026_721_MOESM8_ESM.zip › 3G/Galectin3-Cortex-Ctrl/IBA1-58-Ctx-.tif]

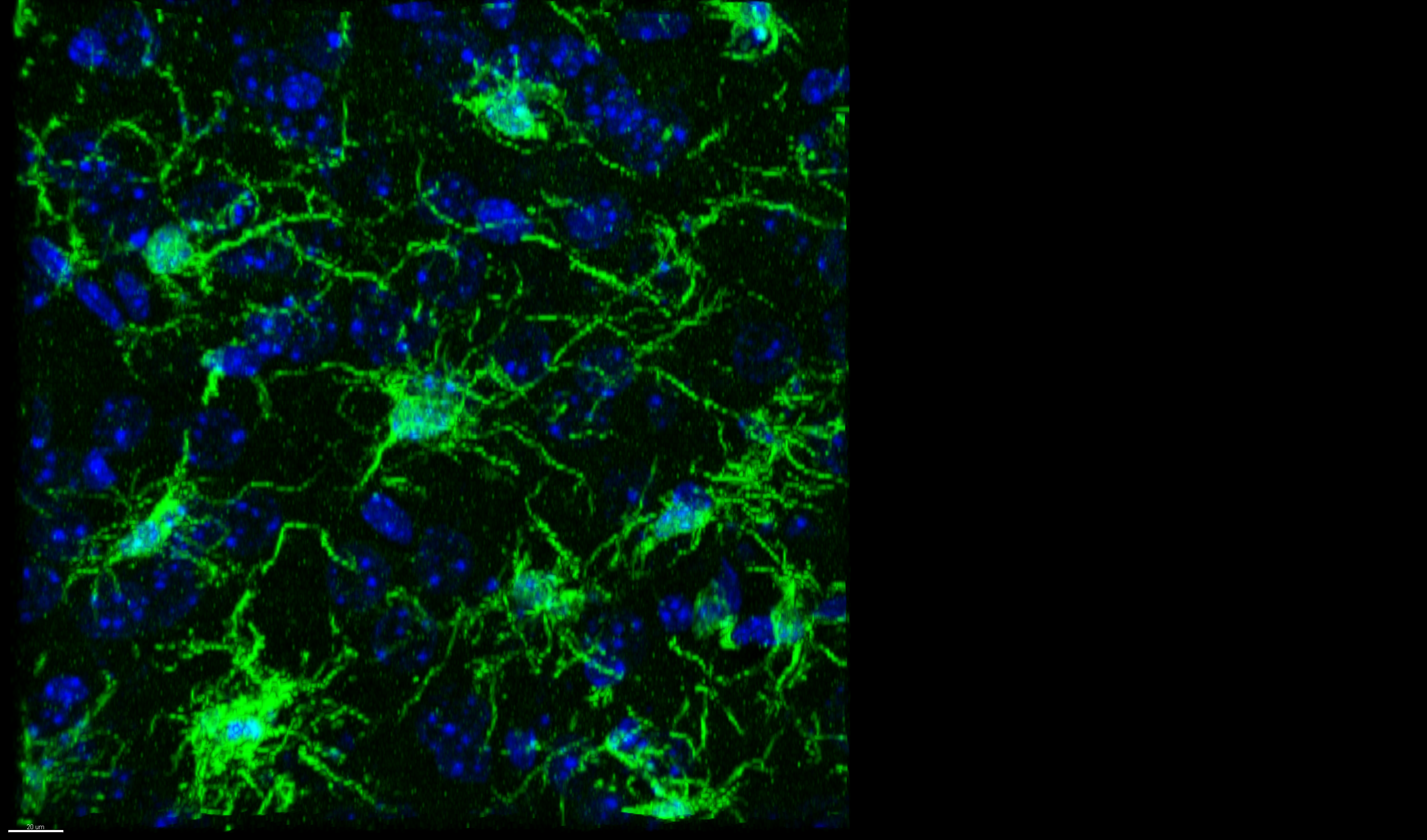

Supplement: Supplementary file 8 — Source data Fig. 3 [file 44319_2026_721_MOESM8_ESM.zip › 3B/CD11b-KO/IBA1-.tif]

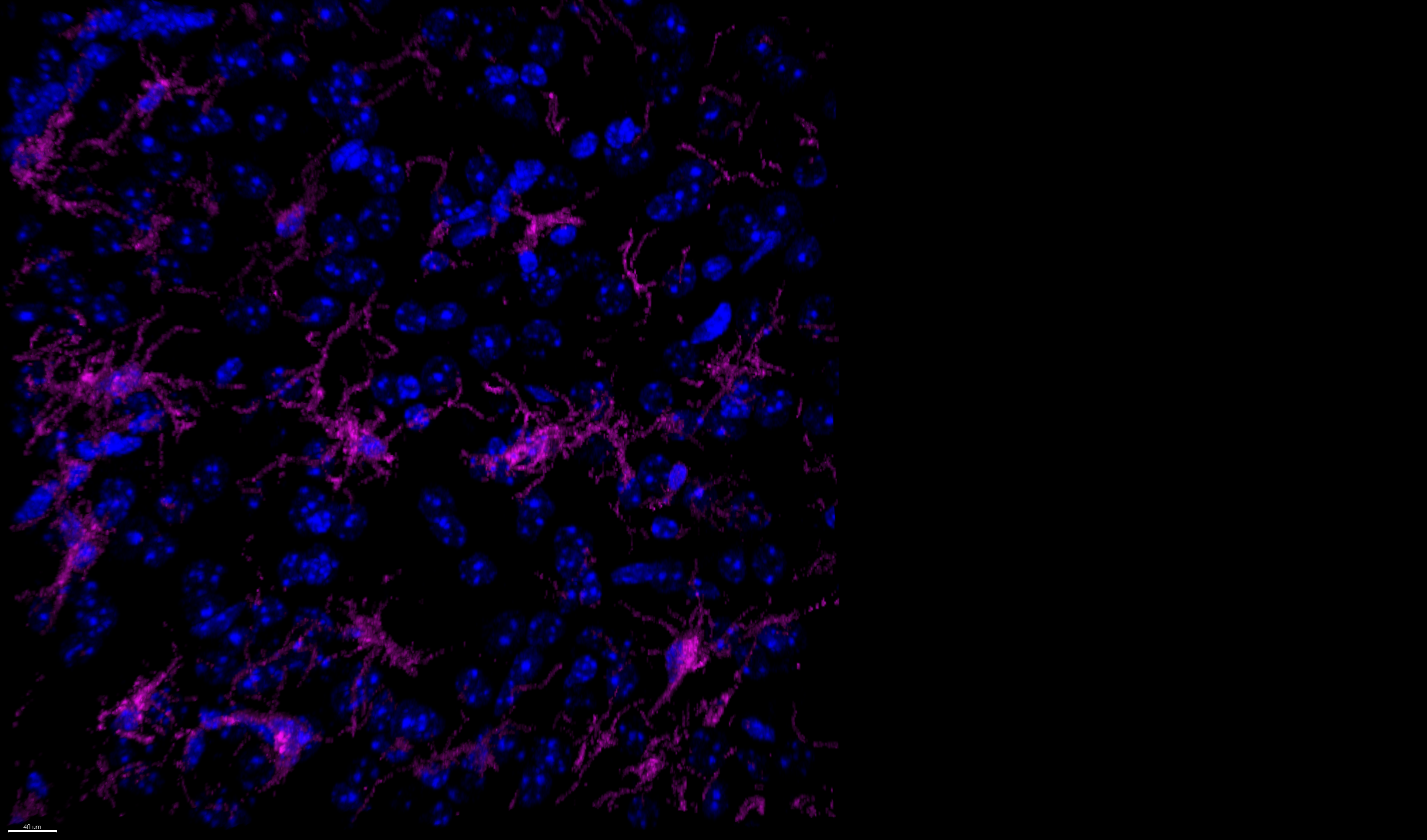

Supplement: Supplementary file 8 — Source data Fig. 3 [file 44319_2026_721_MOESM8_ESM.zip › 3G/AXL-Cotrex-KO/AXLmasked_Image 1.tif]

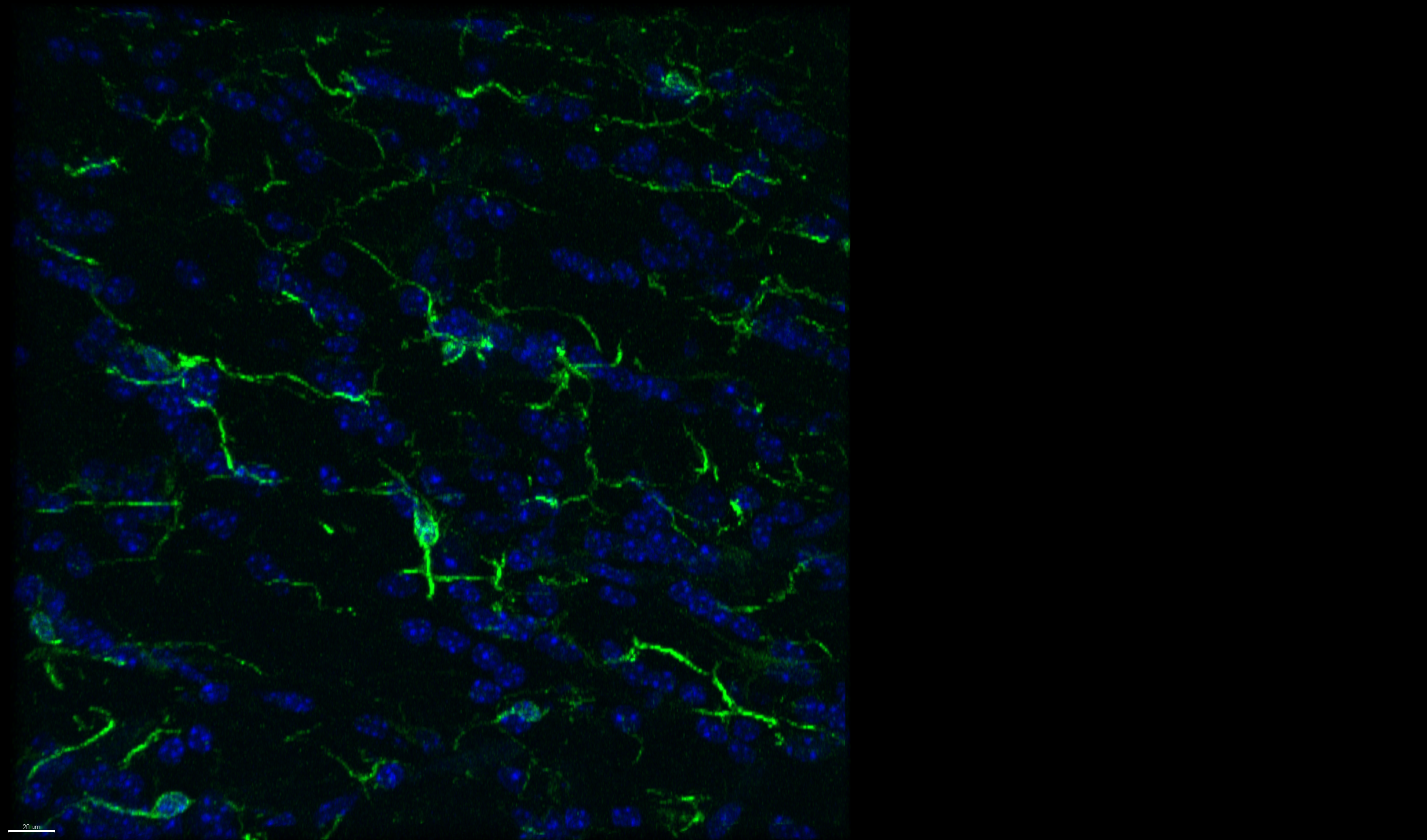

Supplement: Supplementary file 8 — Source data Fig. 3 [file 44319_2026_721_MOESM8_ESM.zip › 3G/AXL-CC-Ctrl/IBA1Image 16_.tif]

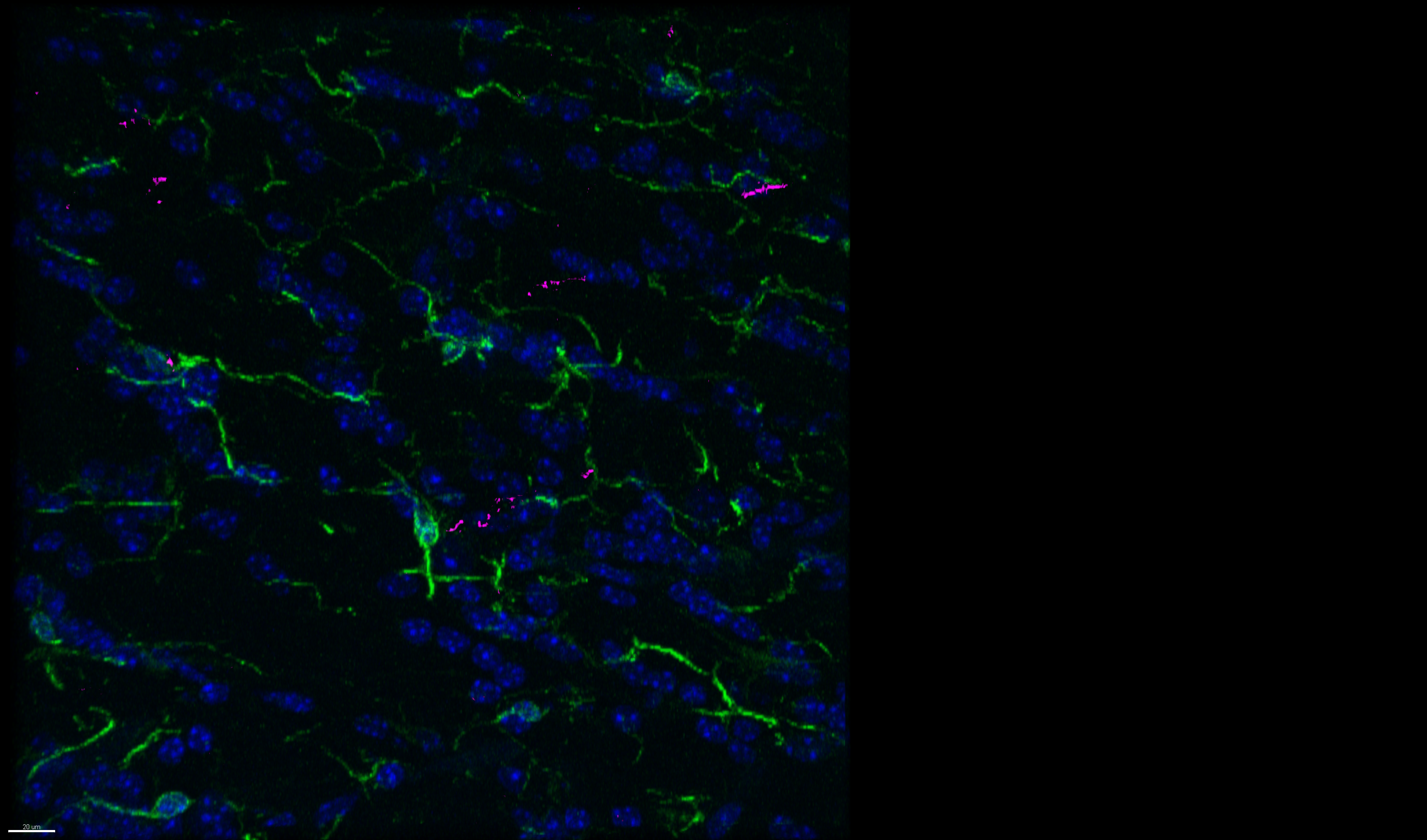

Supplement: Supplementary file 8 — Source data Fig. 3 [file 44319_2026_721_MOESM8_ESM.zip › 3G/AXL-CC-Ctrl/AXL-Image 16_.tif]

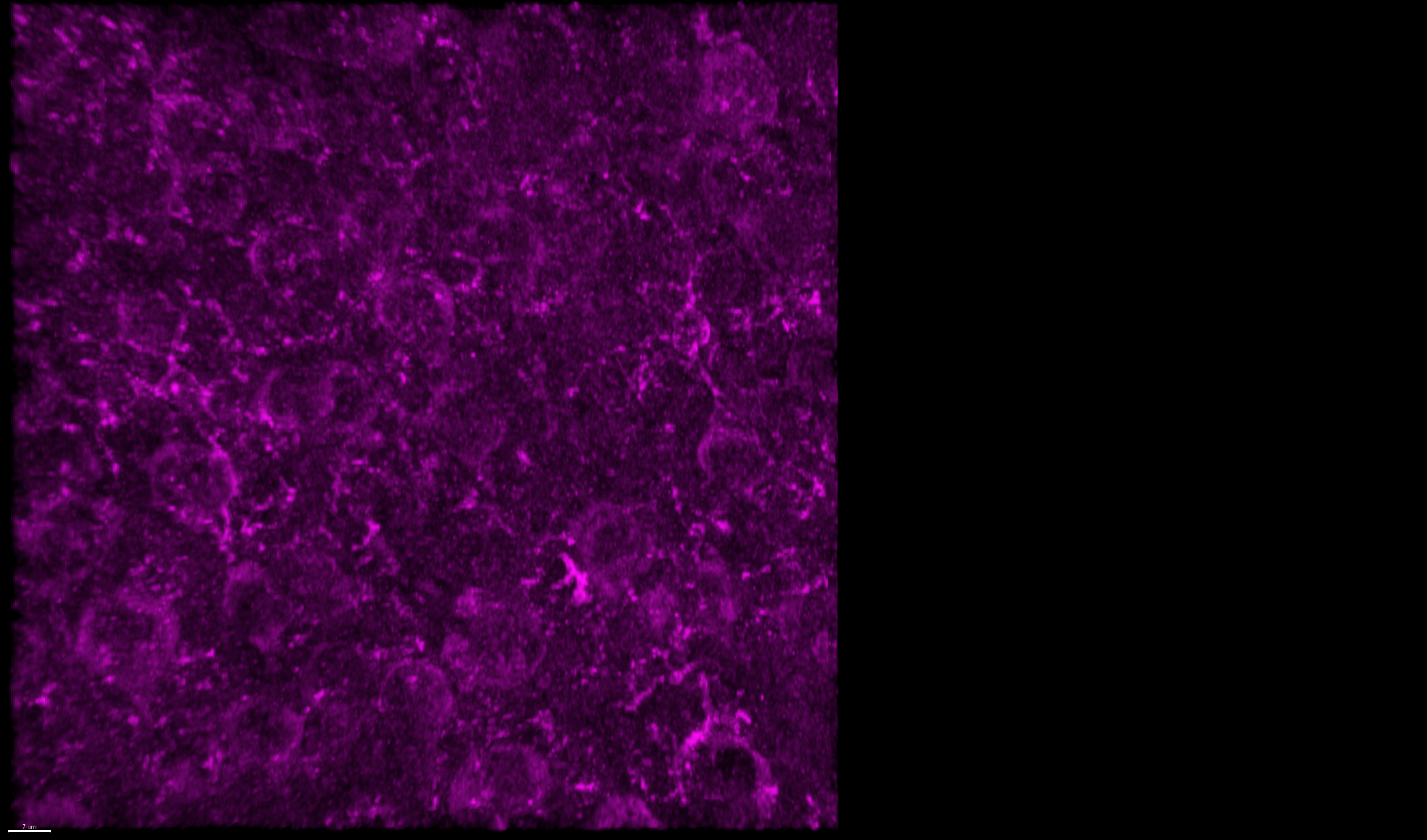

Supplement: Supplementary file 8 — Source data Fig. 3 [file 44319_2026_721_MOESM8_ESM.zip › 3B/CD11b-Ctrl/CD11b original.tif]

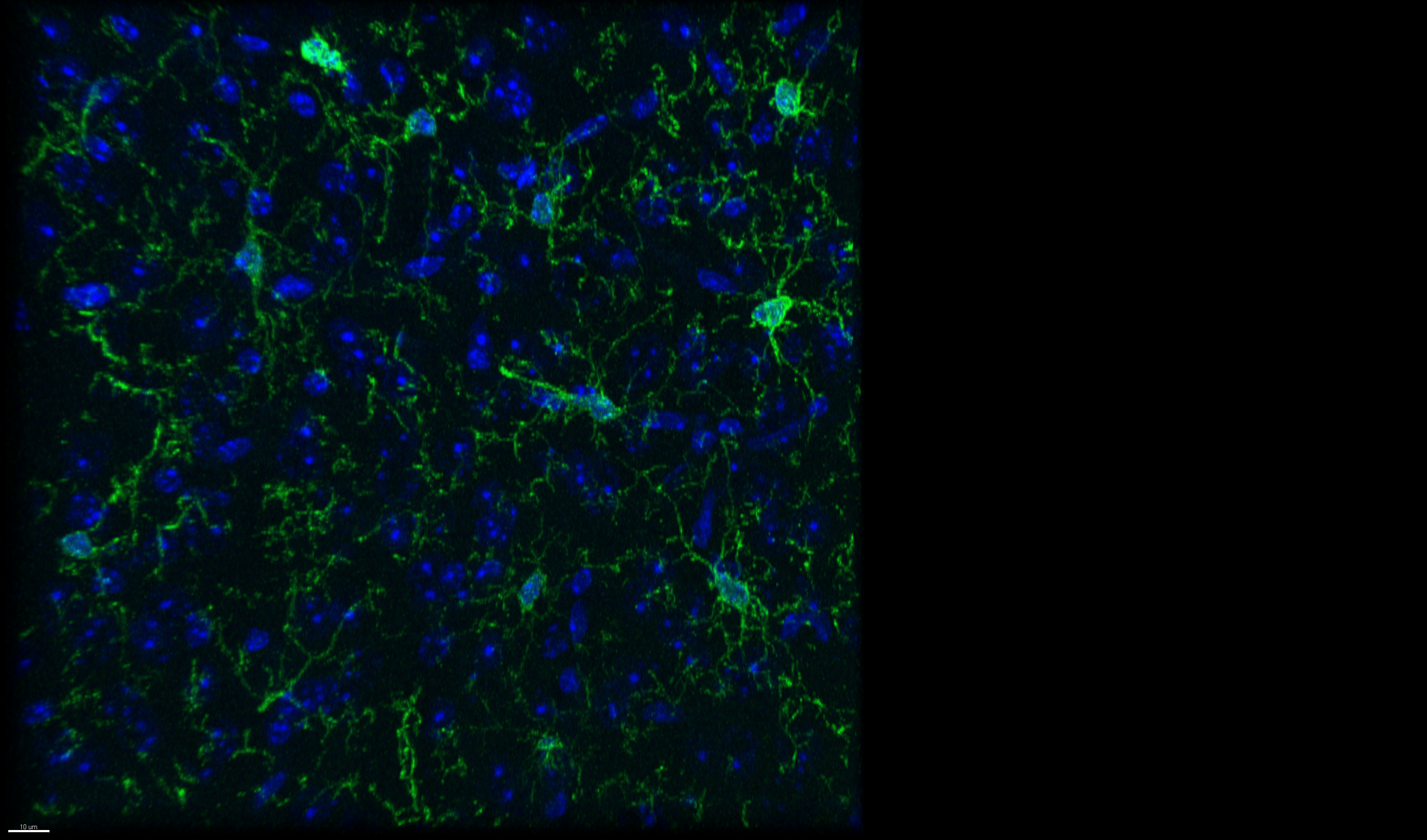

Supplement: Supplementary file 8 — Source data Fig. 3 [file 44319_2026_721_MOESM8_ESM.zip › 3G/AXL-Cotrex-Ctrl/IBA1-Image 1.tif]

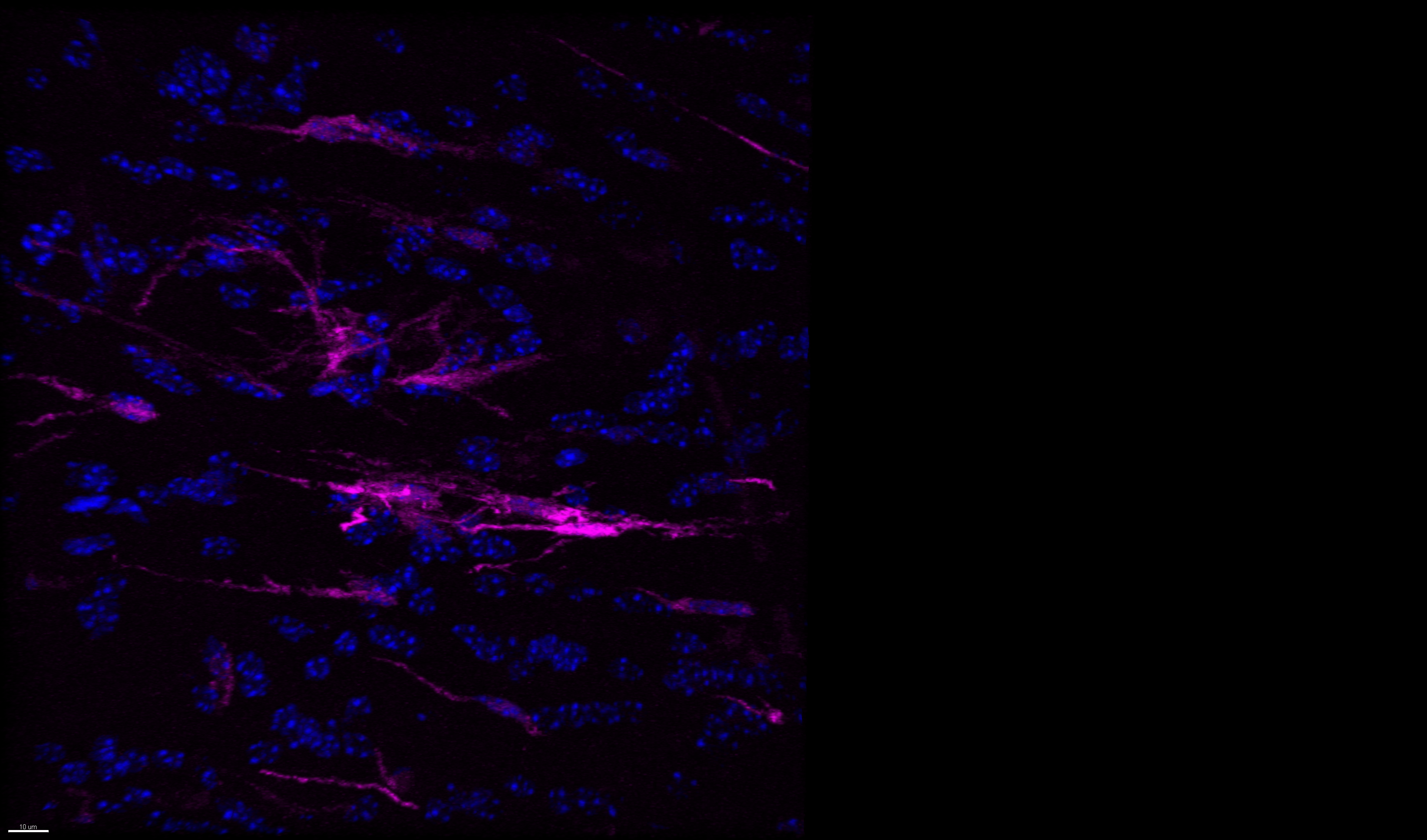

Supplement: Supplementary file 8 — Source data Fig. 3 [file 44319_2026_721_MOESM8_ESM.zip › 3G/Galectin3-CC-KO/Galectin3-Image 14.tif]

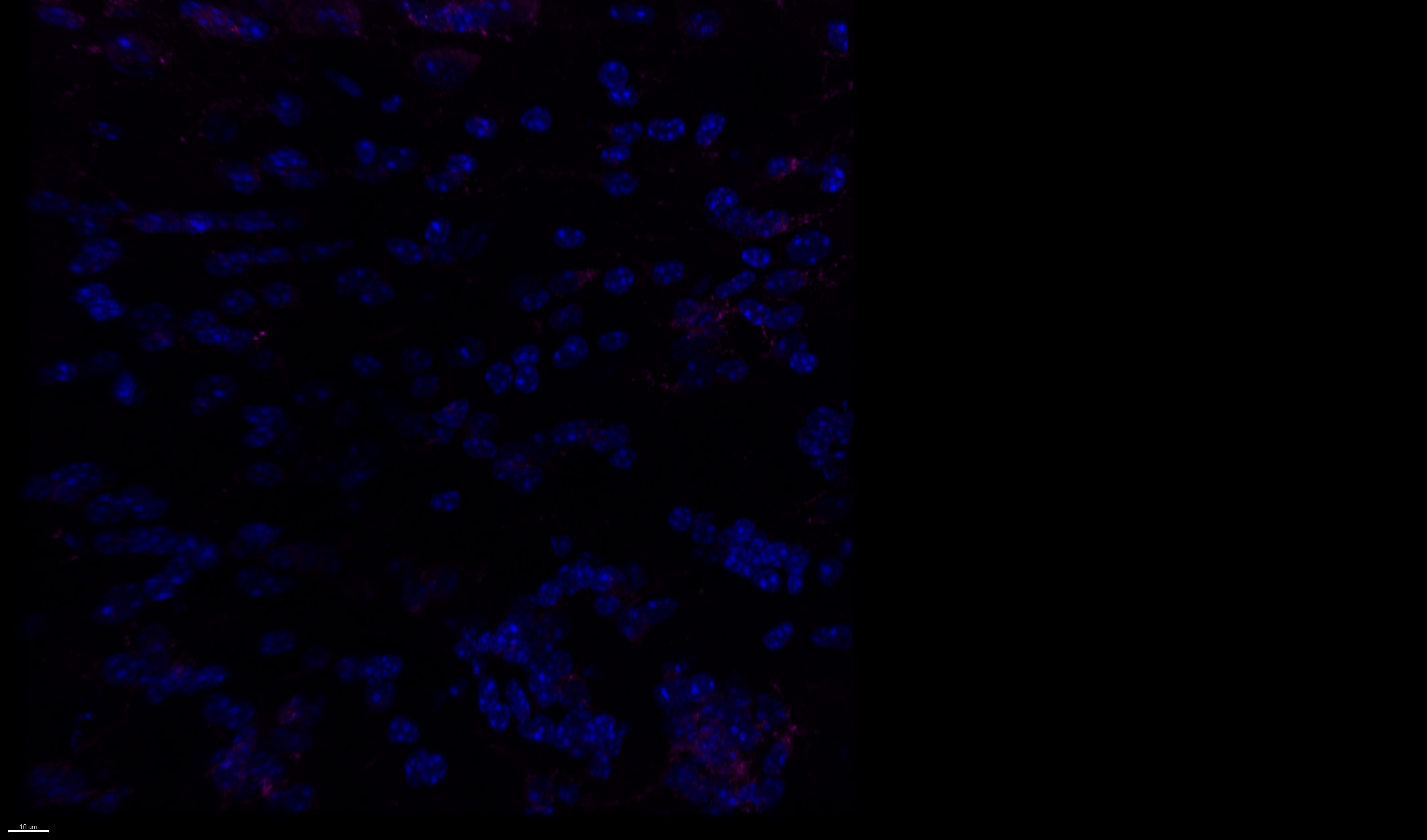

Supplement: Supplementary file 8 — Source data Fig. 3 [file 44319_2026_721_MOESM8_ESM.zip › 3G/Galectin3-CC-Ctrl/Galectin3-58-CC-.tif]

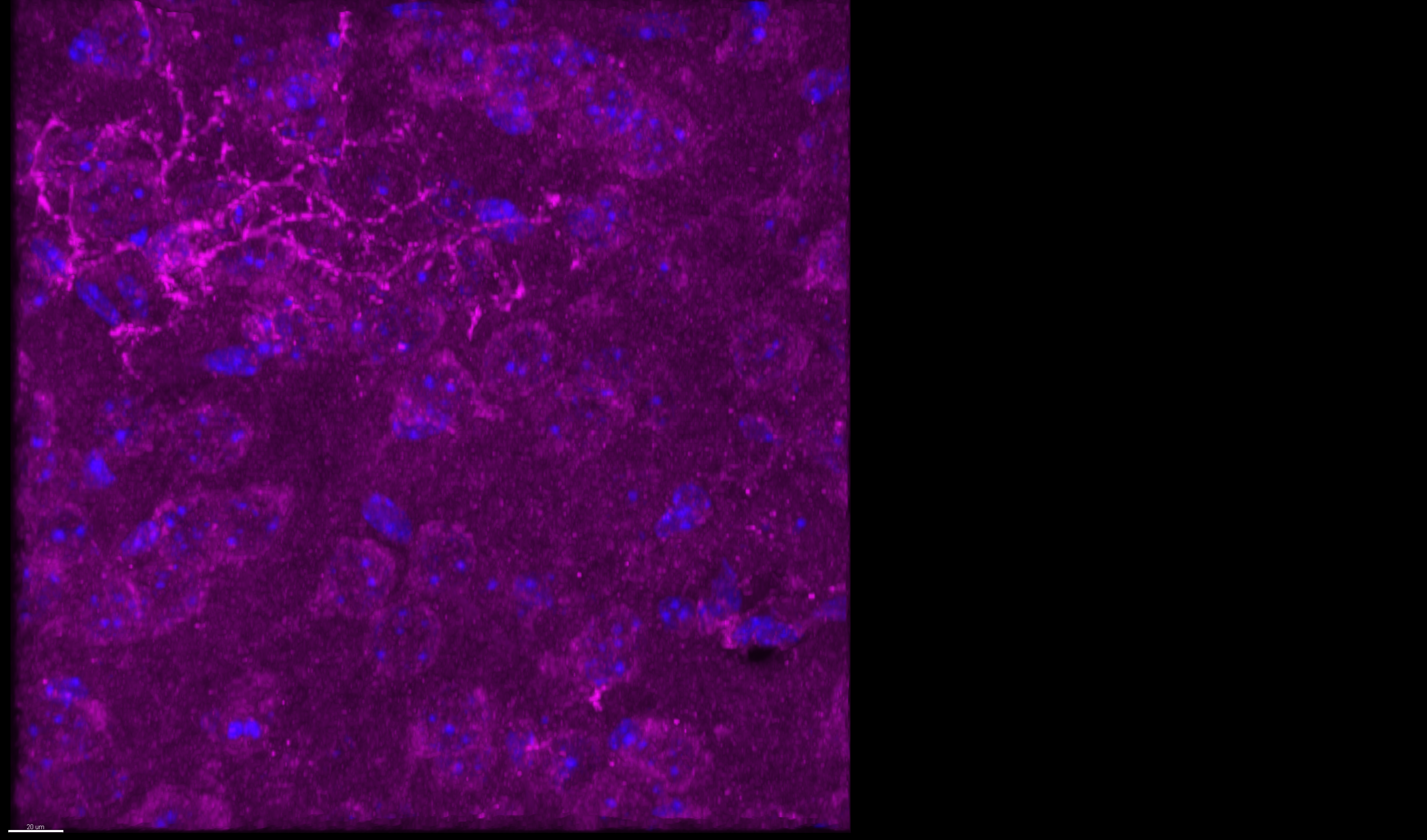

Supplement: Supplementary file 8 — Source data Fig. 3 [file 44319_2026_721_MOESM8_ESM.zip › 3B/CD11b-KO/CD11b original.tif]

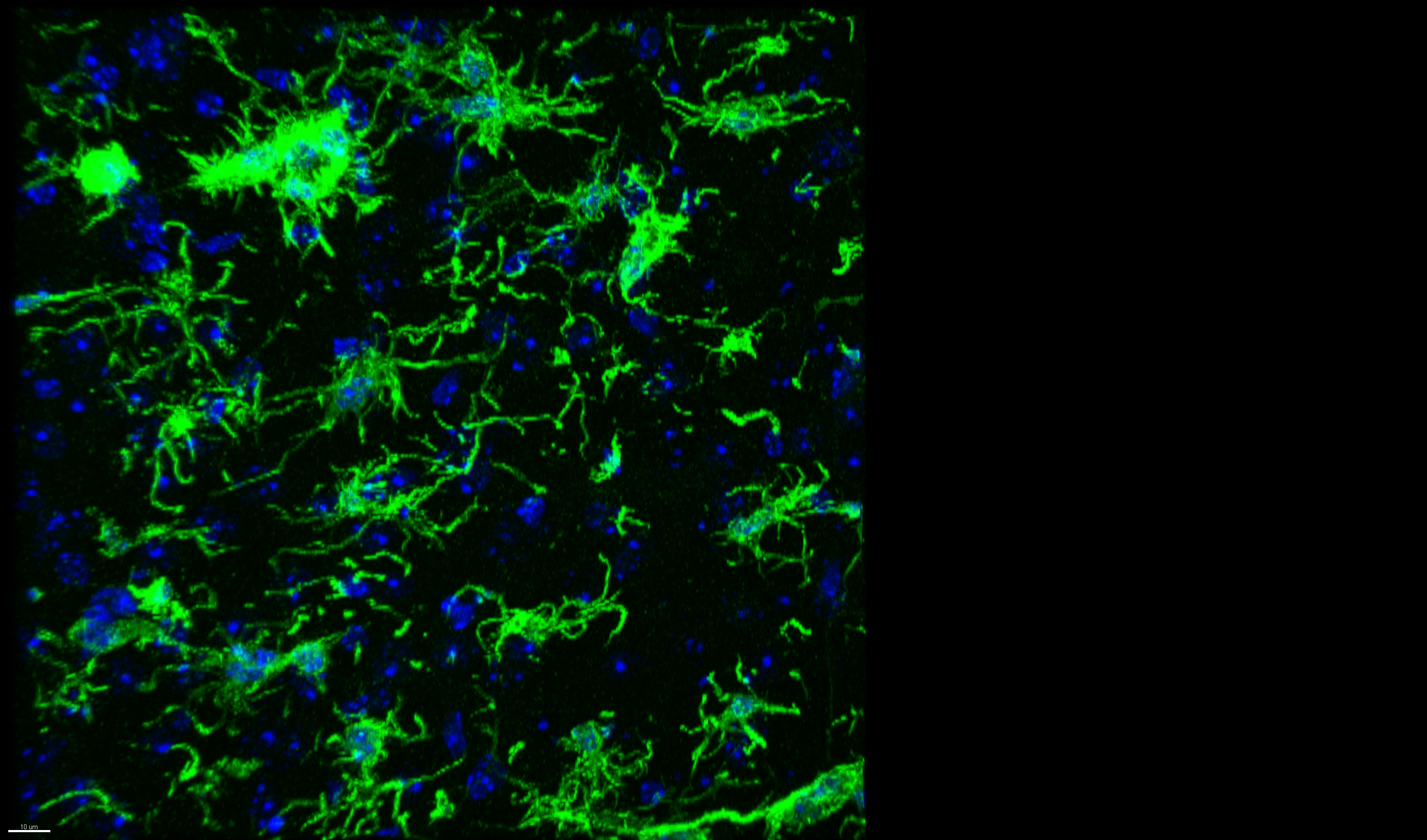

Supplement: Supplementary file 8 — Source data Fig. 3 [file 44319_2026_721_MOESM8_ESM.zip › 3G/Galectin3-Cortex-KO/IBA1-Image 16.tif]

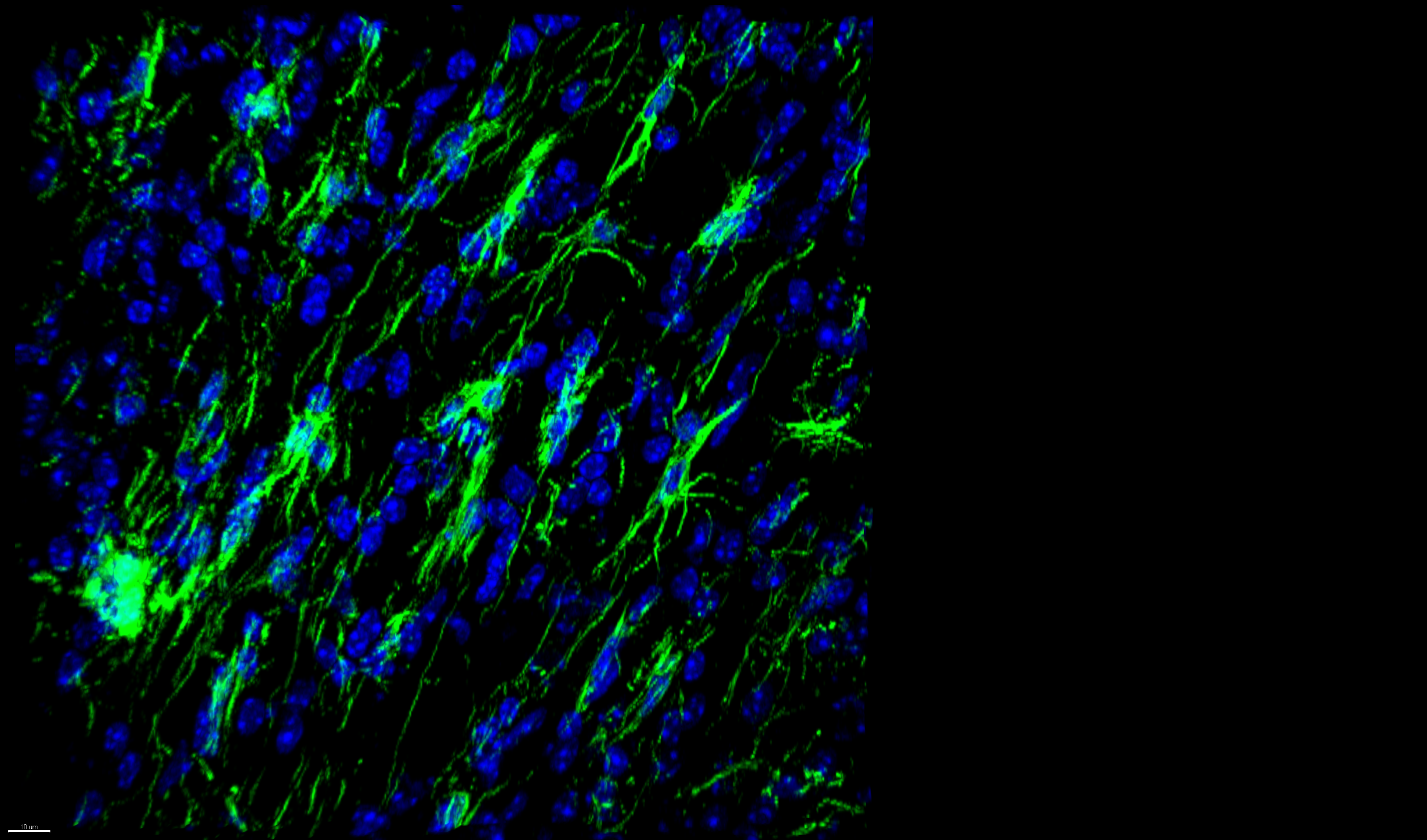

Supplement: Supplementary file 8 — Source data Fig. 3 [file 44319_2026_721_MOESM8_ESM.zip › 3G/AXL-CC-KO/IBA1_Image 6_.tif]

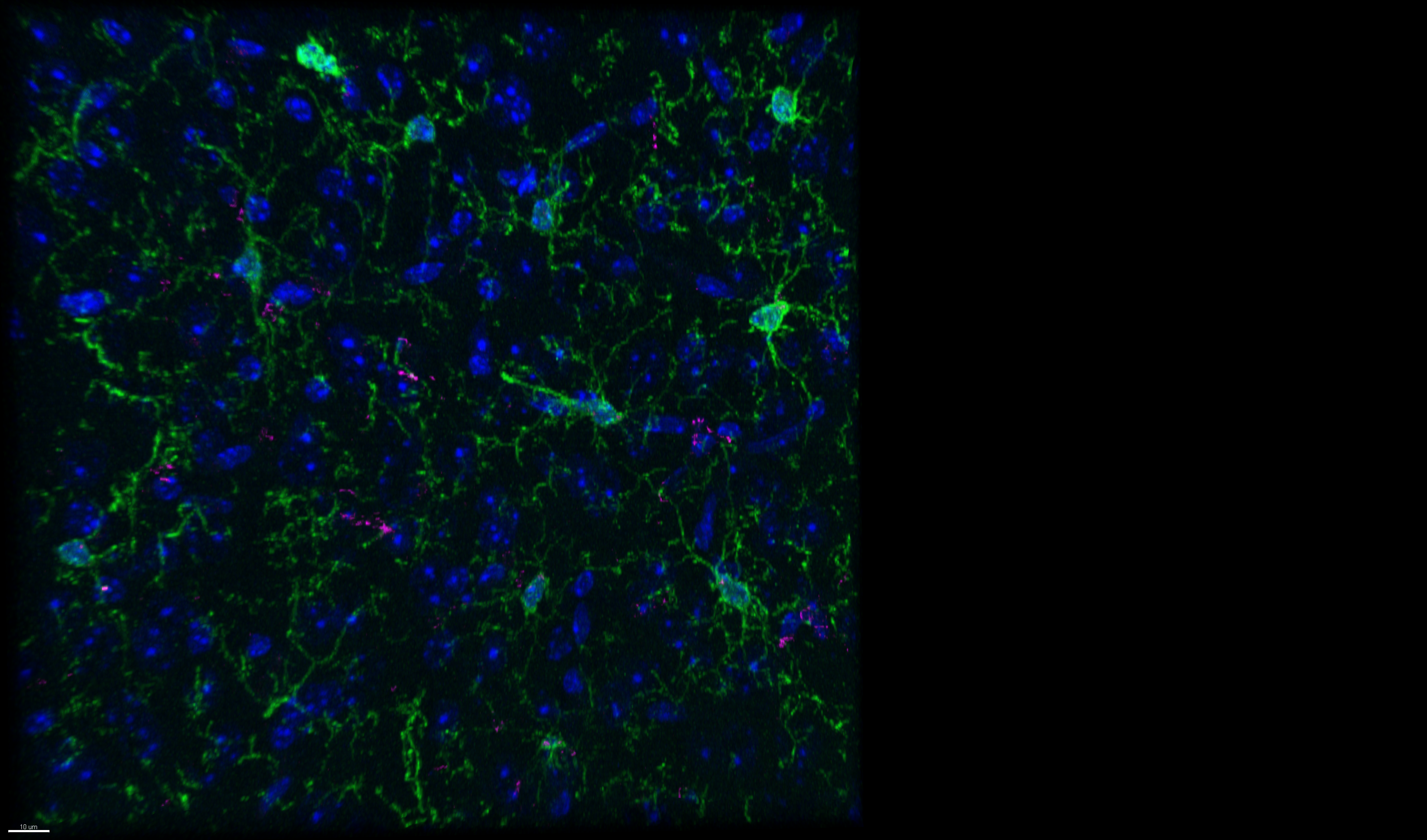

Supplement: Supplementary file 8 — Source data Fig. 3 [file 44319_2026_721_MOESM8_ESM.zip › 3G/AXL-Cotrex-Ctrl/AXL-Image 1_.tif]

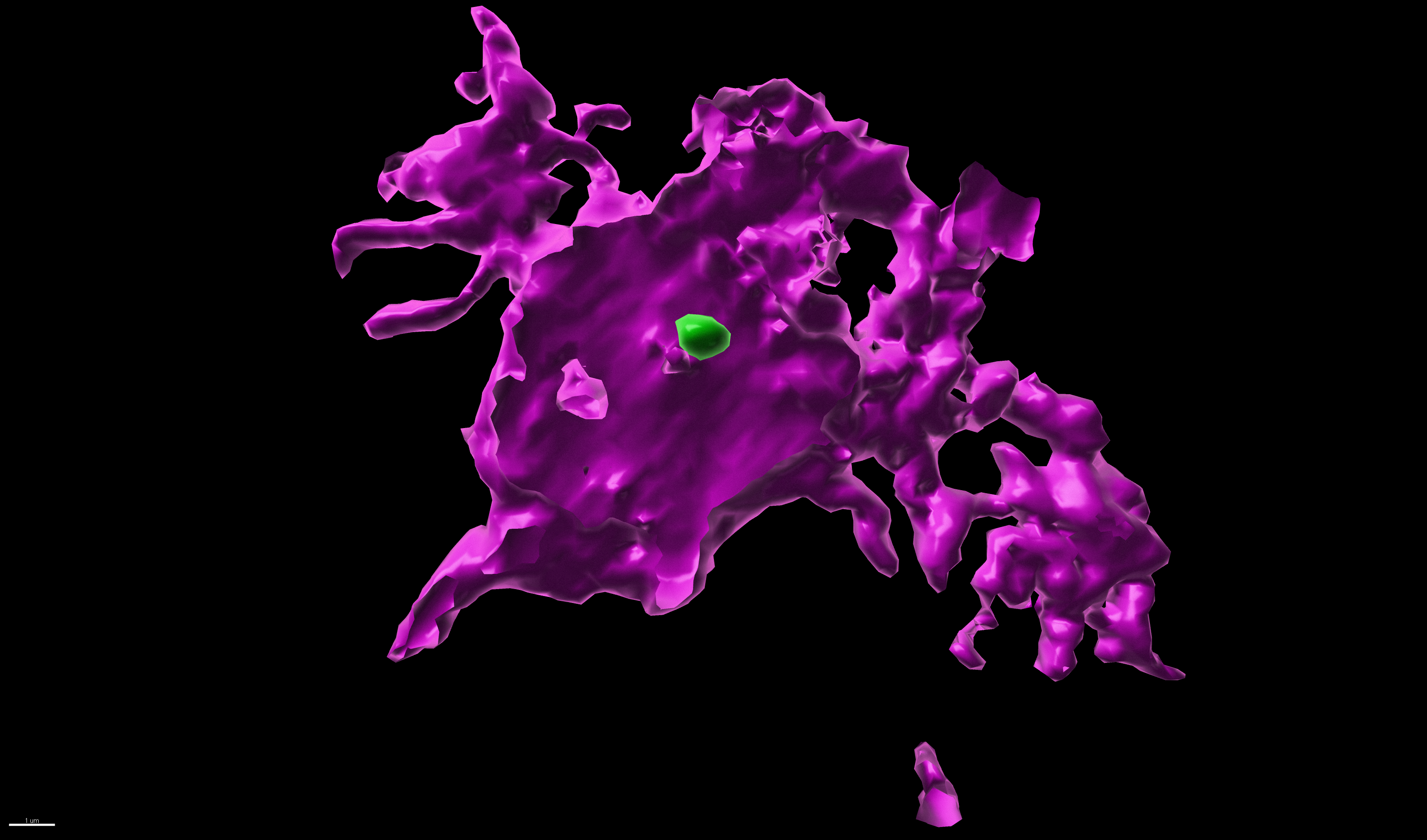

Supplement: Supplementary file 10 — Source data Fig. 5 [file 44319_2026_721_MOESM10_ESM.zip › 5H/KO-Images/3D recunstructions/IBA1-MBP-Clipped.tif]

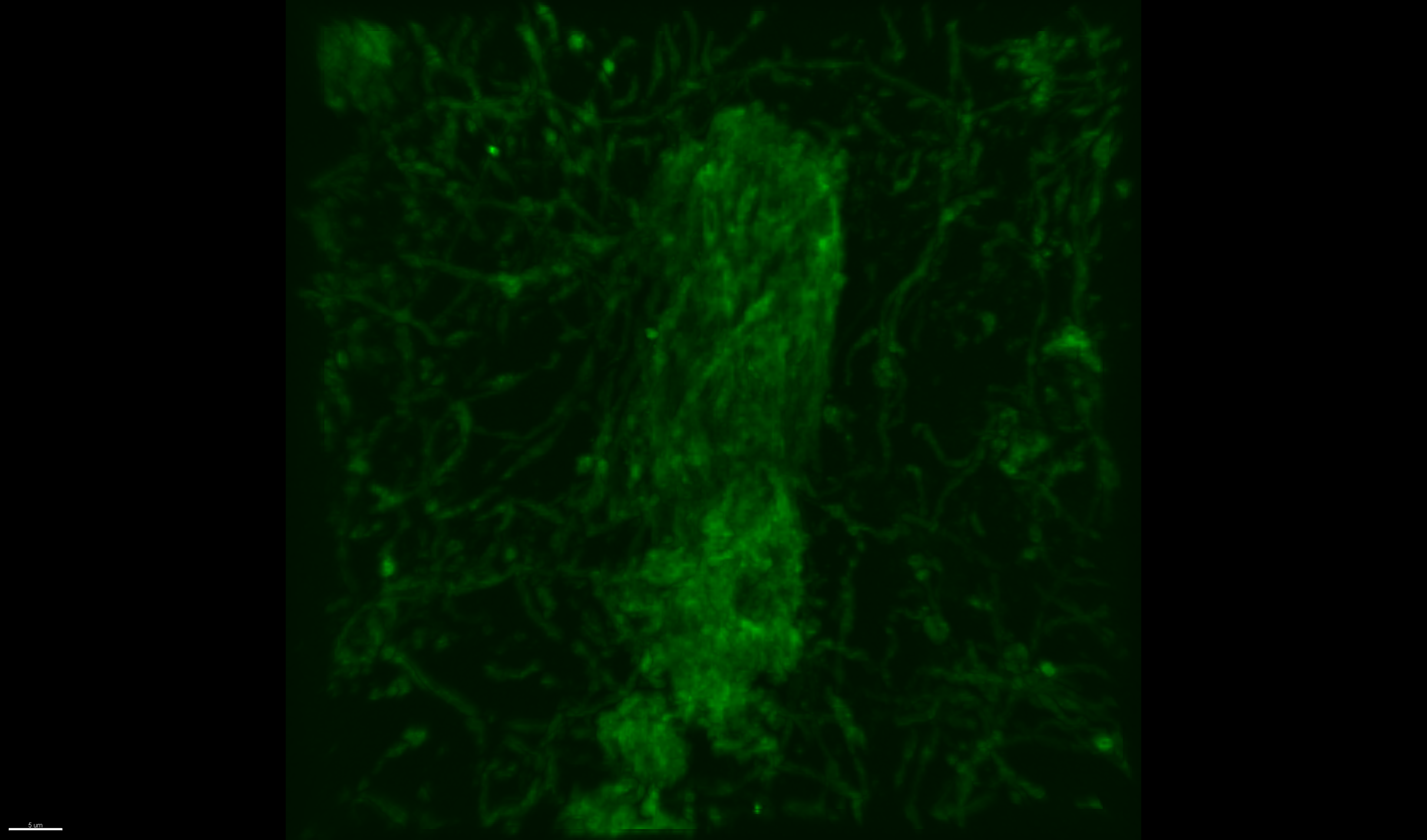

Supplement: Supplementary file 10 — Source data Fig. 5 [file 44319_2026_721_MOESM10_ESM.zip › 5H/KO-Images/MBP.tif]

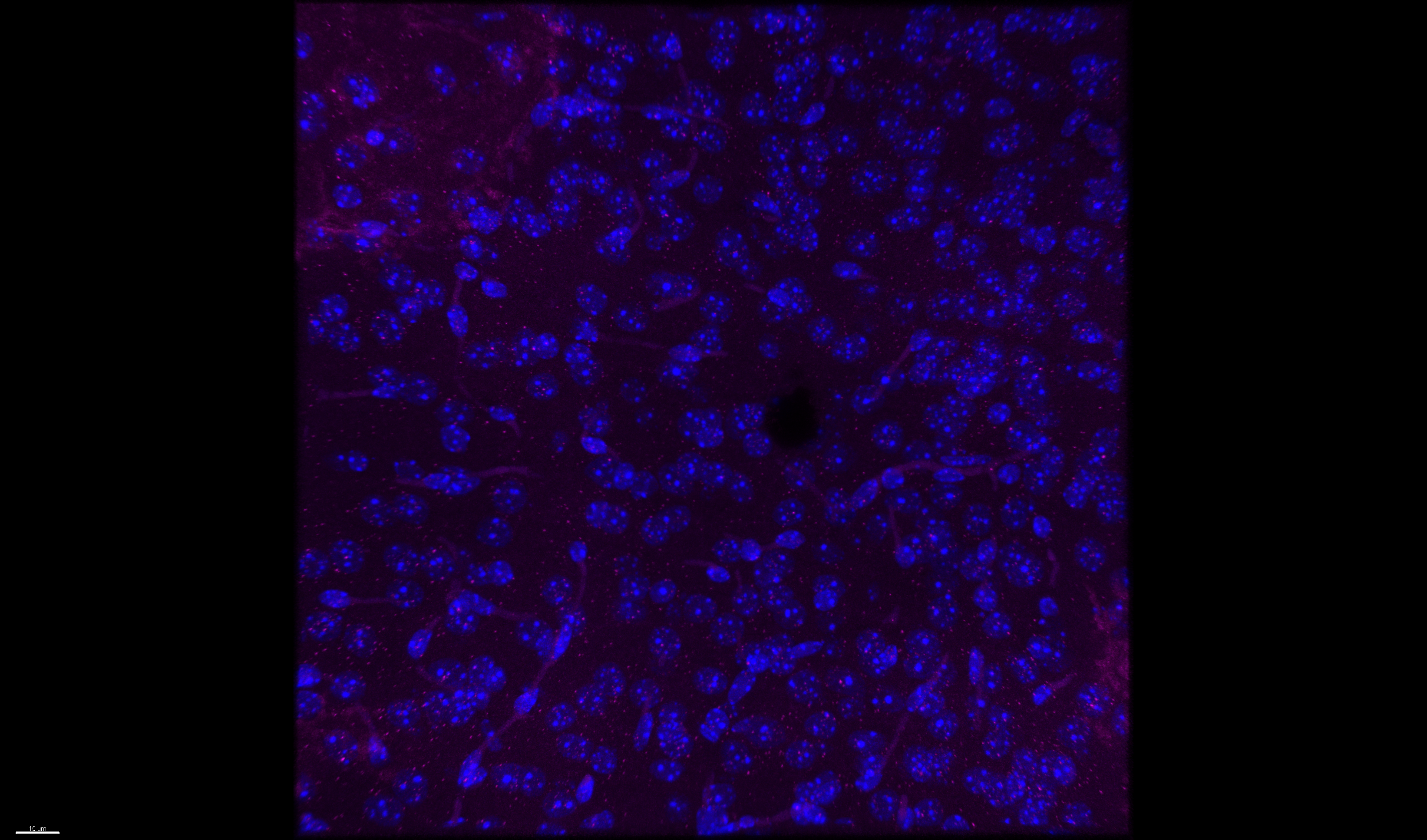

Supplement: Supplementary file 10 — Source data Fig. 5 [file 44319_2026_721_MOESM10_ESM.zip › 5F/Control/Ms4a7_[ii17_48_TileScan_001_Merging_Image_18]Crop1_2025-09-09T11-41-47.816.tif]

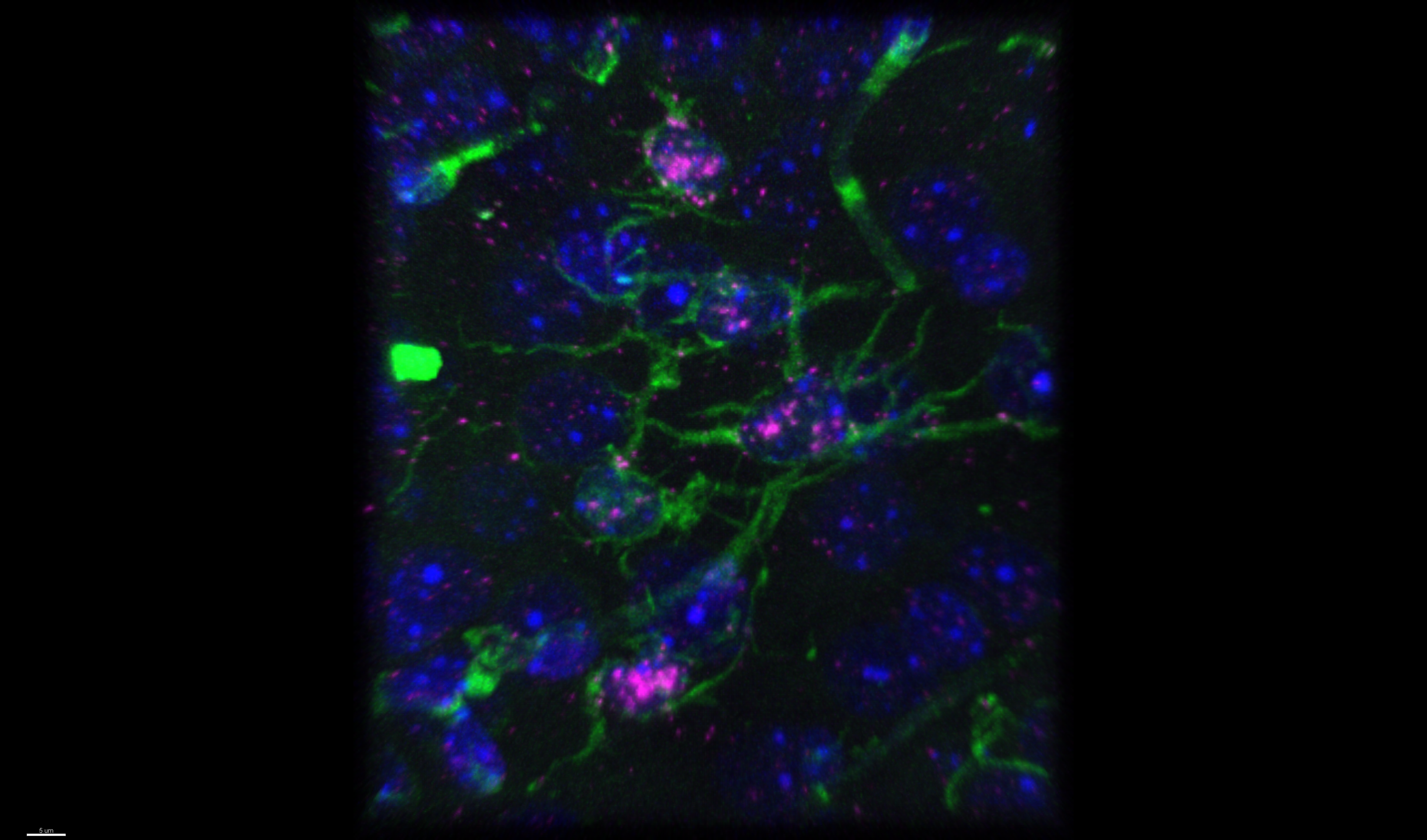

Supplement: Supplementary file 10 — Source data Fig. 5 [file 44319_2026_721_MOESM10_ESM.zip › 5F/KO/Zoom-in/Marge-56_[ii20_TileScan_001_Merging_Image_21]-Crop1_2025-09-09T11-46-25.140.tif]

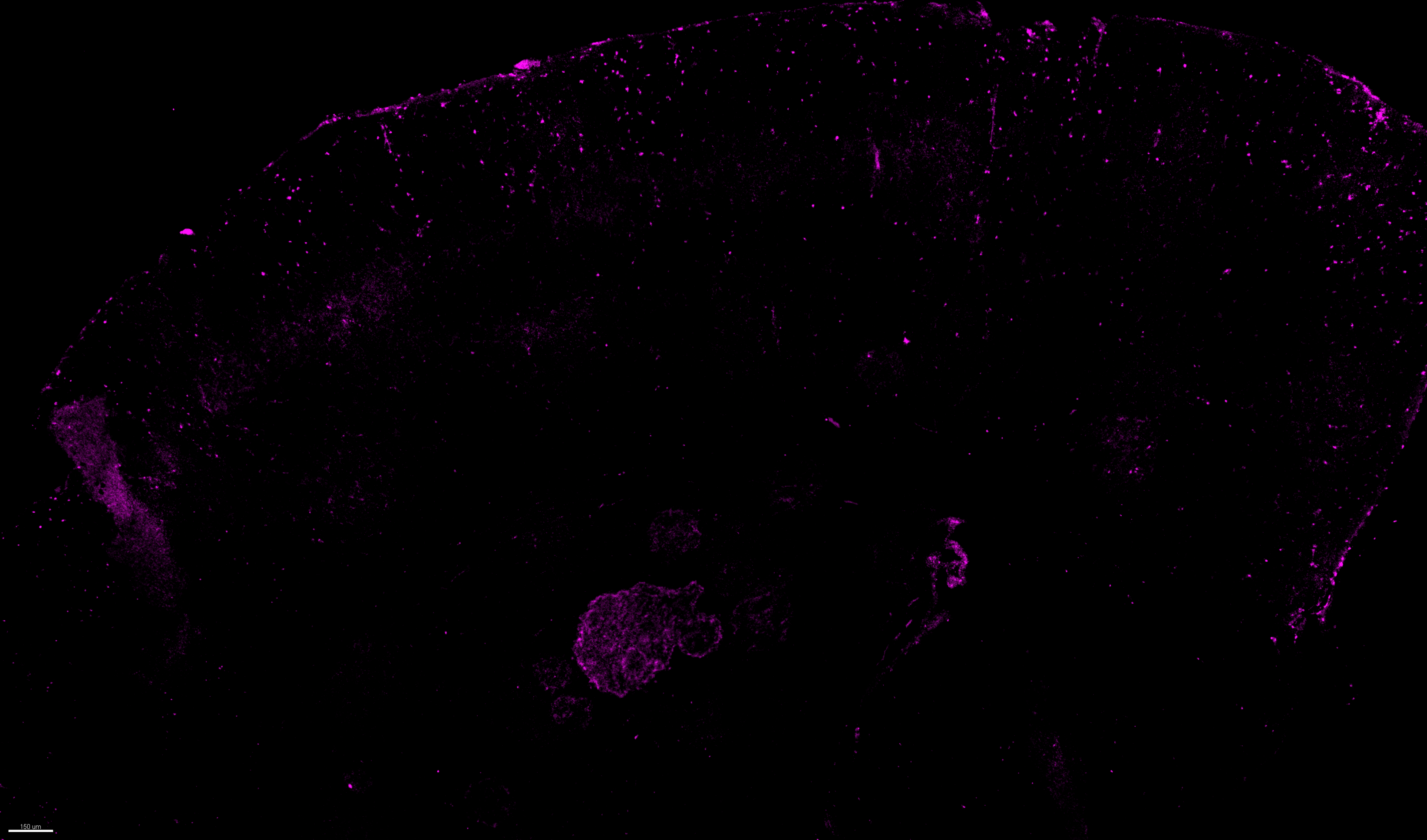

Supplement: Supplementary file 10 — Source data Fig. 5 [file 44319_2026_721_MOESM10_ESM.zip › 5G/Zoom in cortex-Ms4a7.tif]

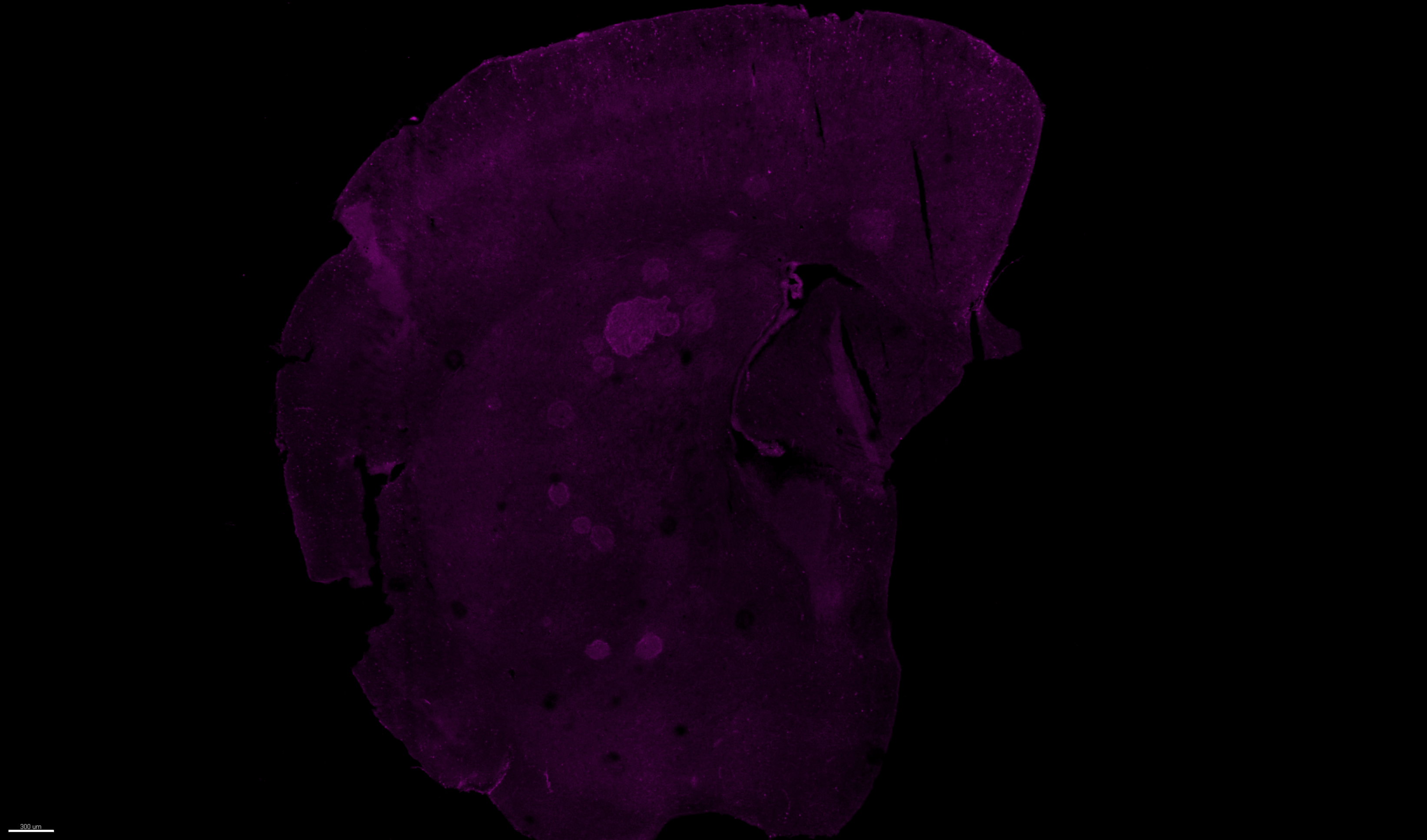

Supplement: Supplementary file 10 — Source data Fig. 5 [file 44319_2026_721_MOESM10_ESM.zip › 5G/Whole brain-original Ms4a7.tif]

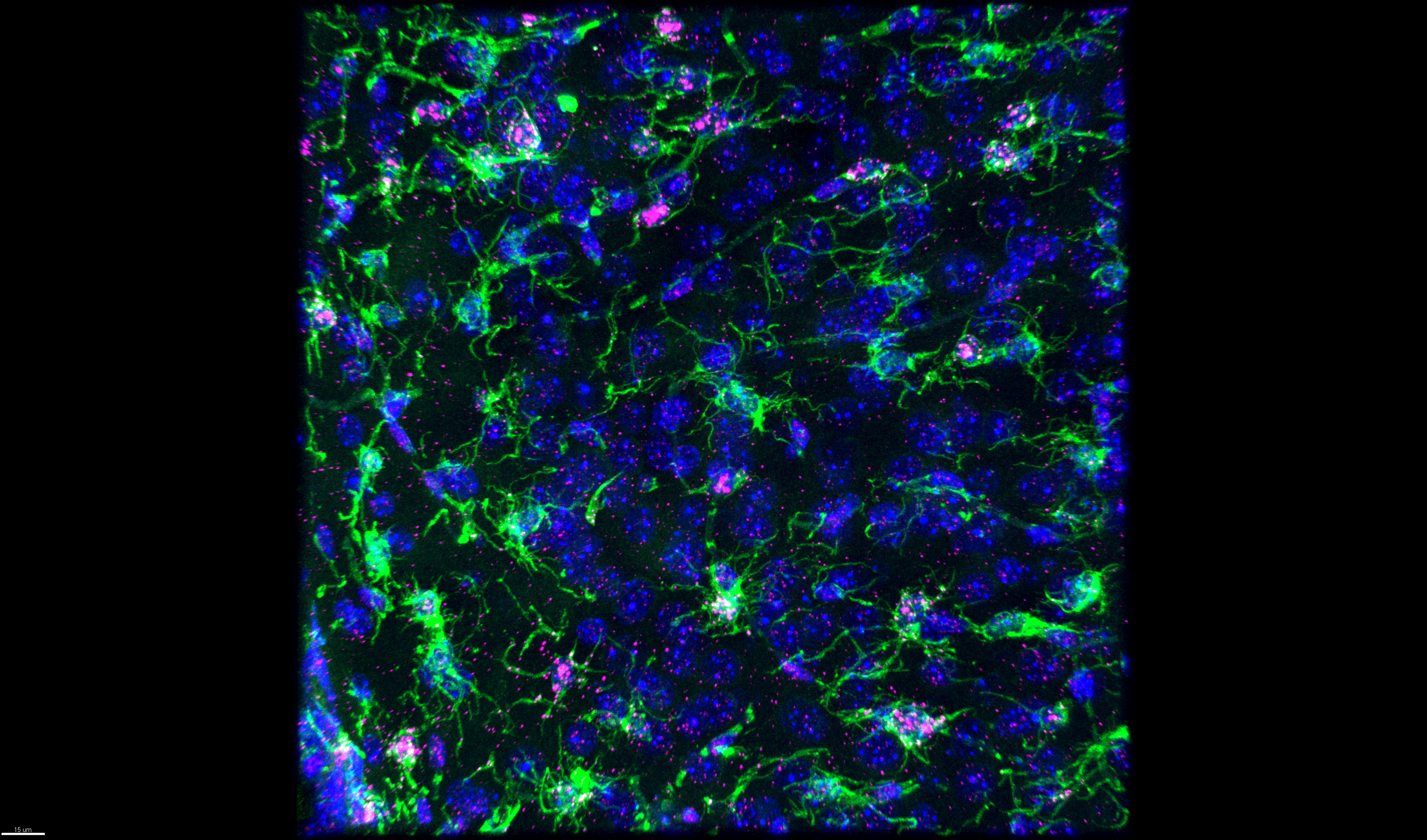

Supplement: Supplementary file 10 — Source data Fig. 5 [file 44319_2026_721_MOESM10_ESM.zip › 5F/KO/Overview/Merge-56_[ii0_TileScan_001_(Stage_1_of_20)_Image_1]_2025-09-09T11-56-59.498.tif]

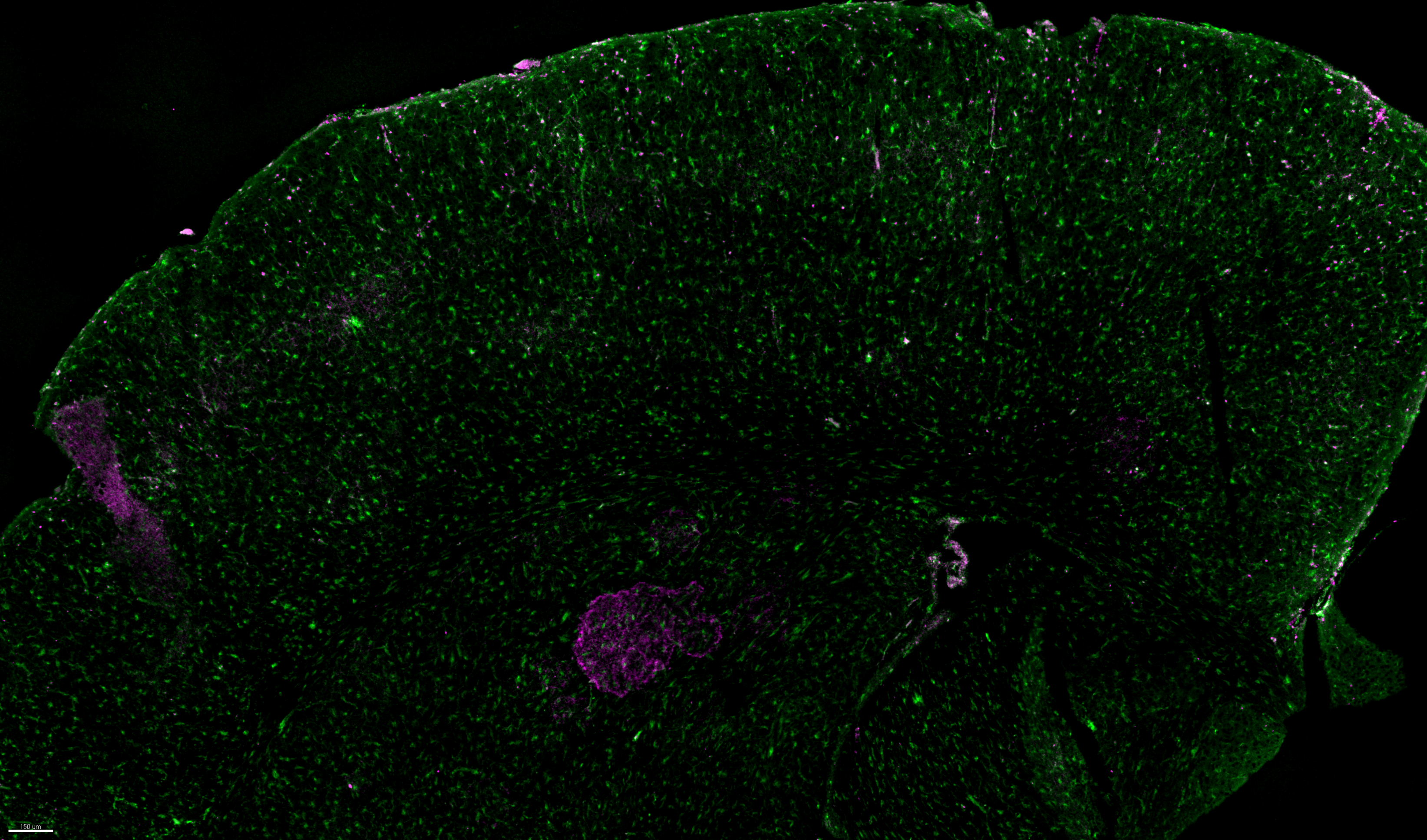

Supplement: Supplementary file 10 — Source data Fig. 5 [file 44319_2026_721_MOESM10_ESM.zip › 5G/Zoom in cortex-IBA1- Ms4a7.tif]

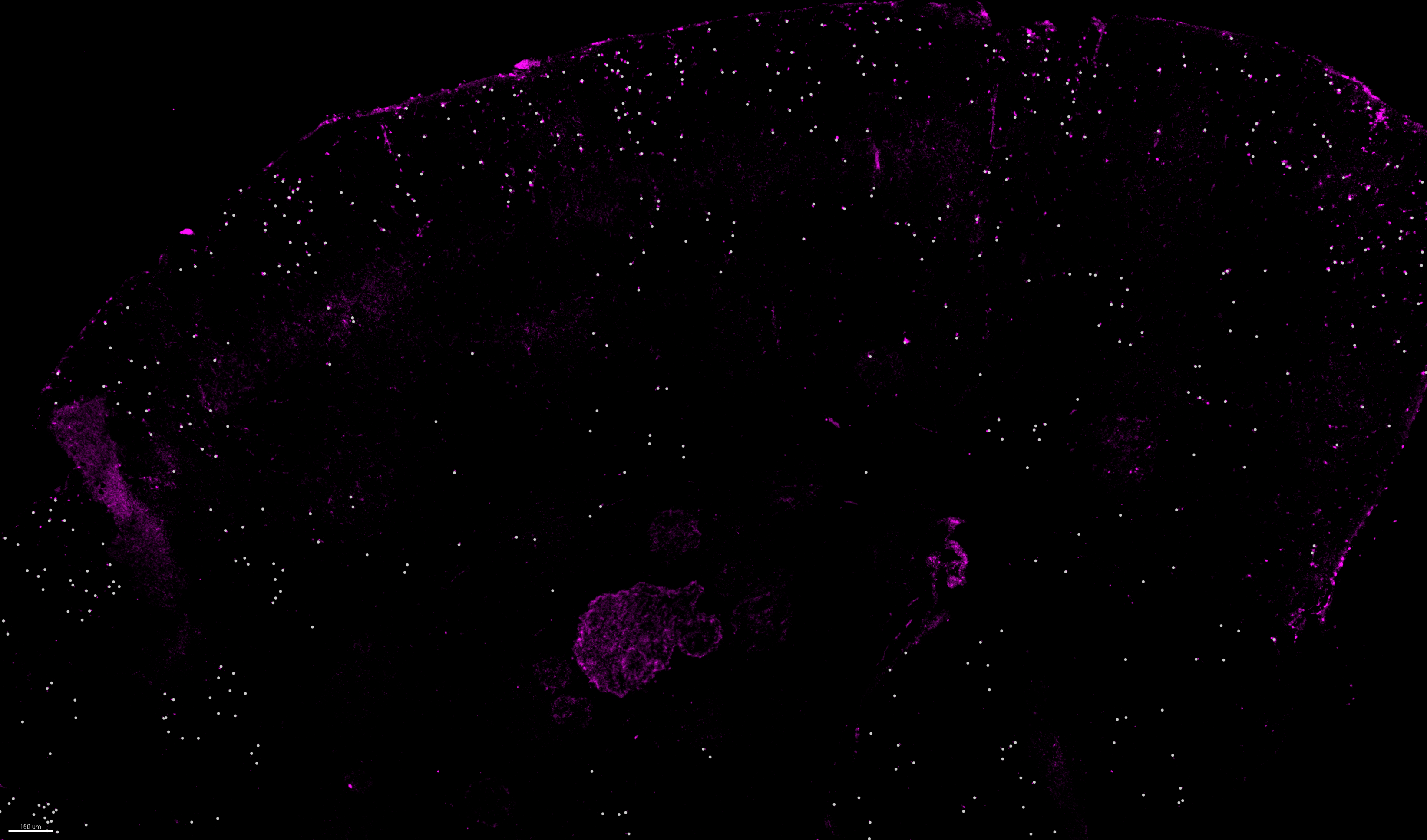

Supplement: Supplementary file 10 — Source data Fig. 5 [file 44319_2026_721_MOESM10_ESM.zip › 5G/Zoom in cortex-spotted Ms4a7.tif]

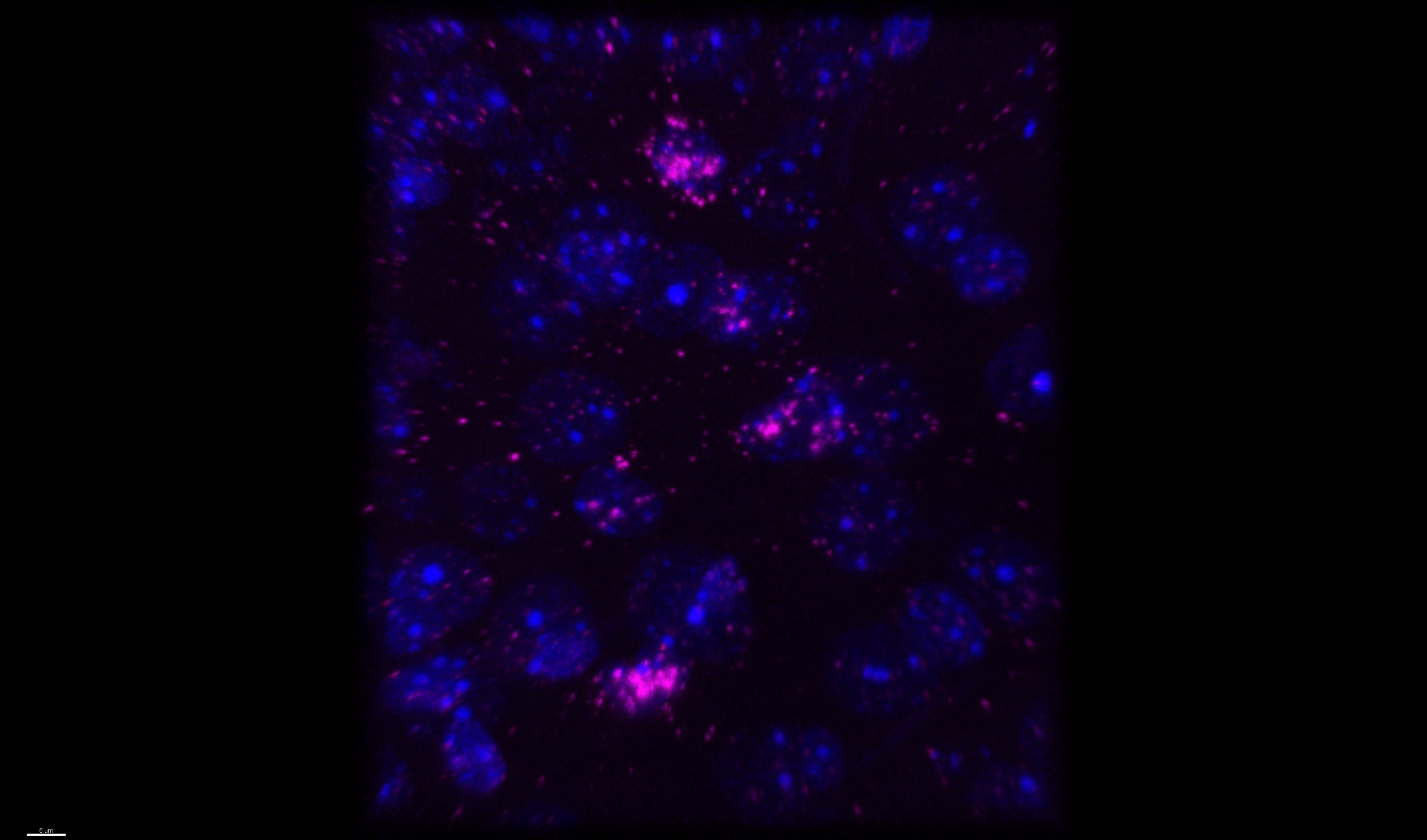

Supplement: Supplementary file 10 — Source data Fig. 5 [file 44319_2026_721_MOESM10_ESM.zip › 5F/KO/Zoom-in/Ms4a7-56_[ii20_TileScan_001_Merging_Image_21]-Crop1_2025-09-09T11-46-17.021.tif]

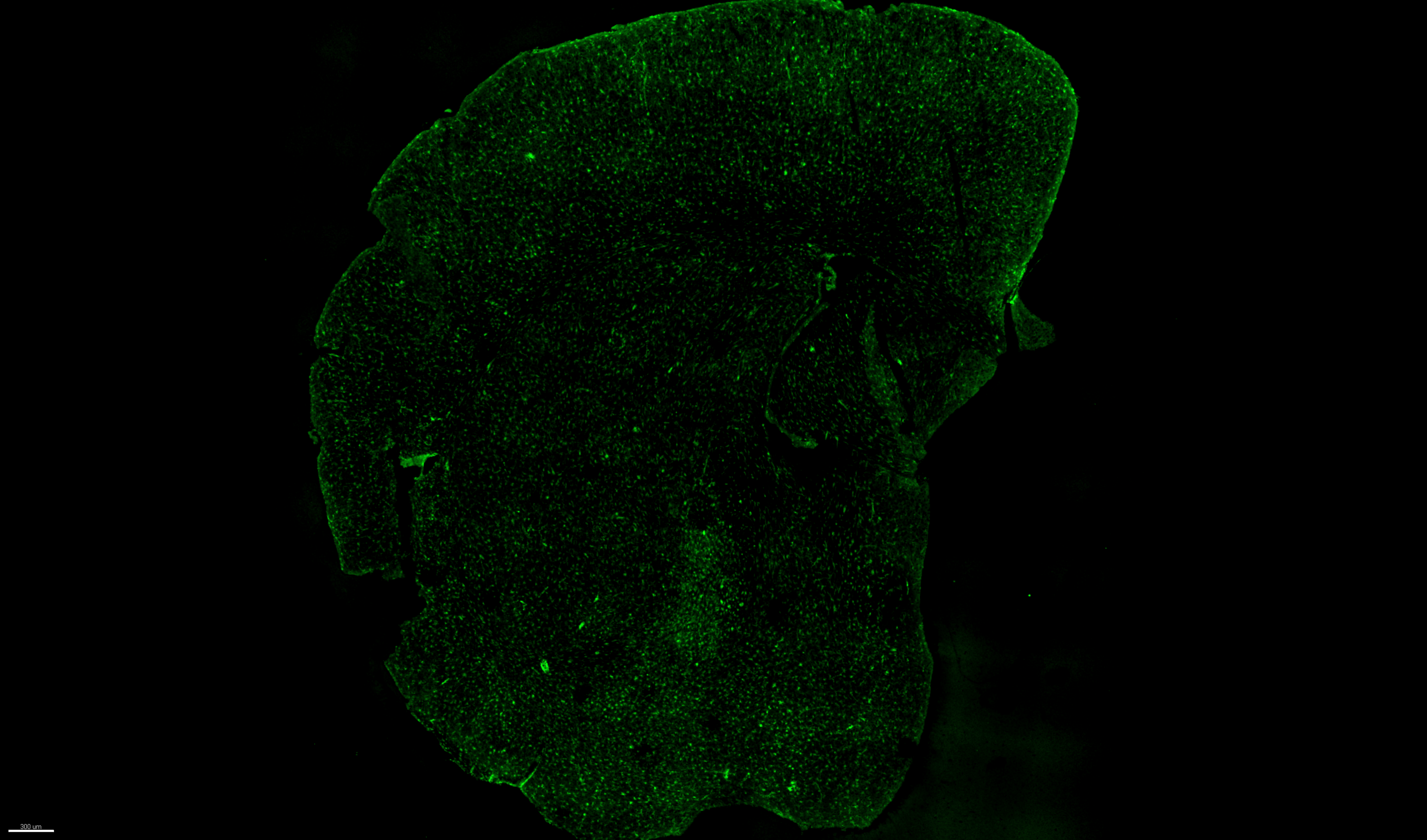

Supplement: Supplementary file 10 — Source data Fig. 5 [file 44319_2026_721_MOESM10_ESM.zip › 5G/Whole brain-IBA1.tif]

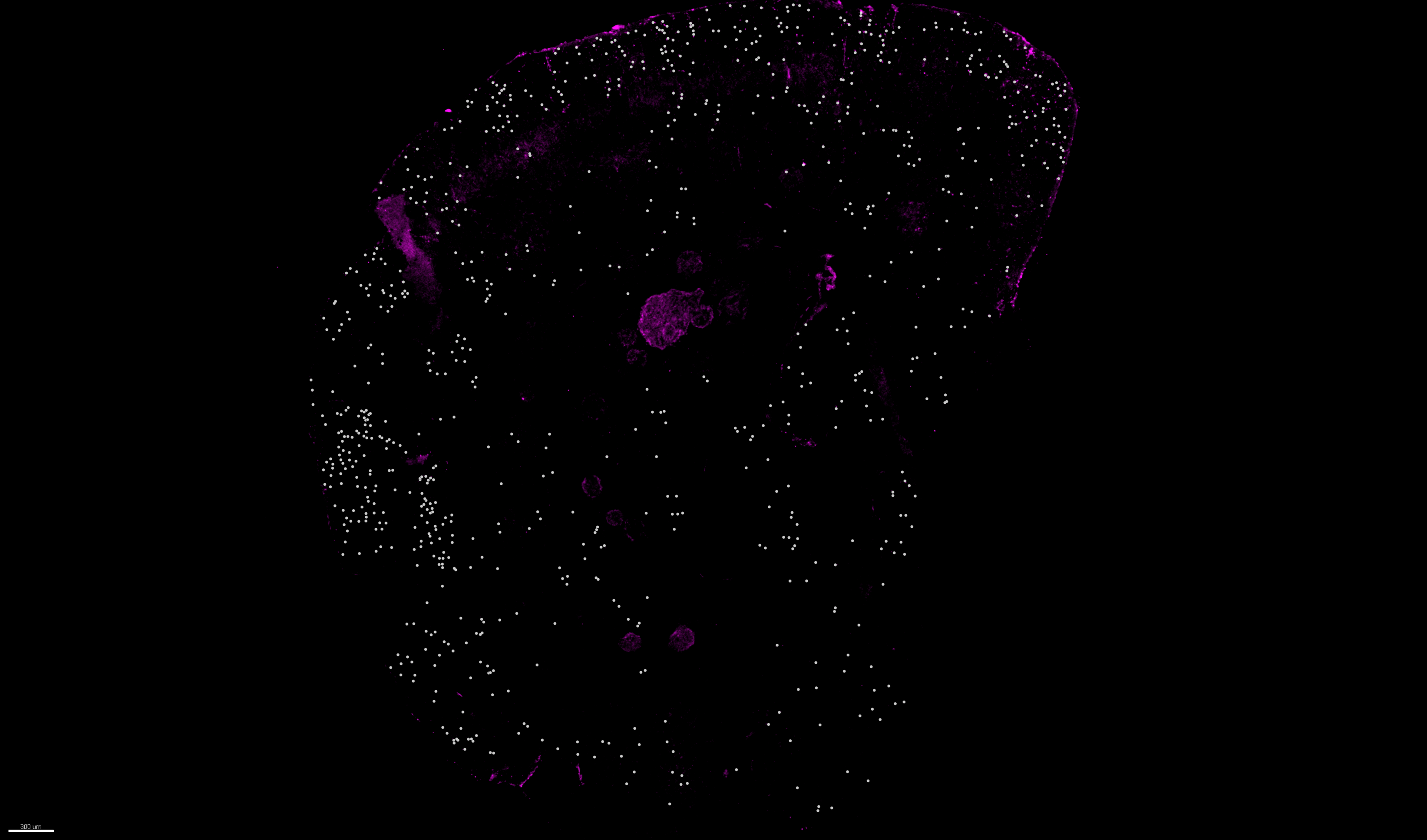

Supplement: Supplementary file 10 — Source data Fig. 5 [file 44319_2026_721_MOESM10_ESM.zip › 5G/Whole brain-spotted Ms4a7.tif]

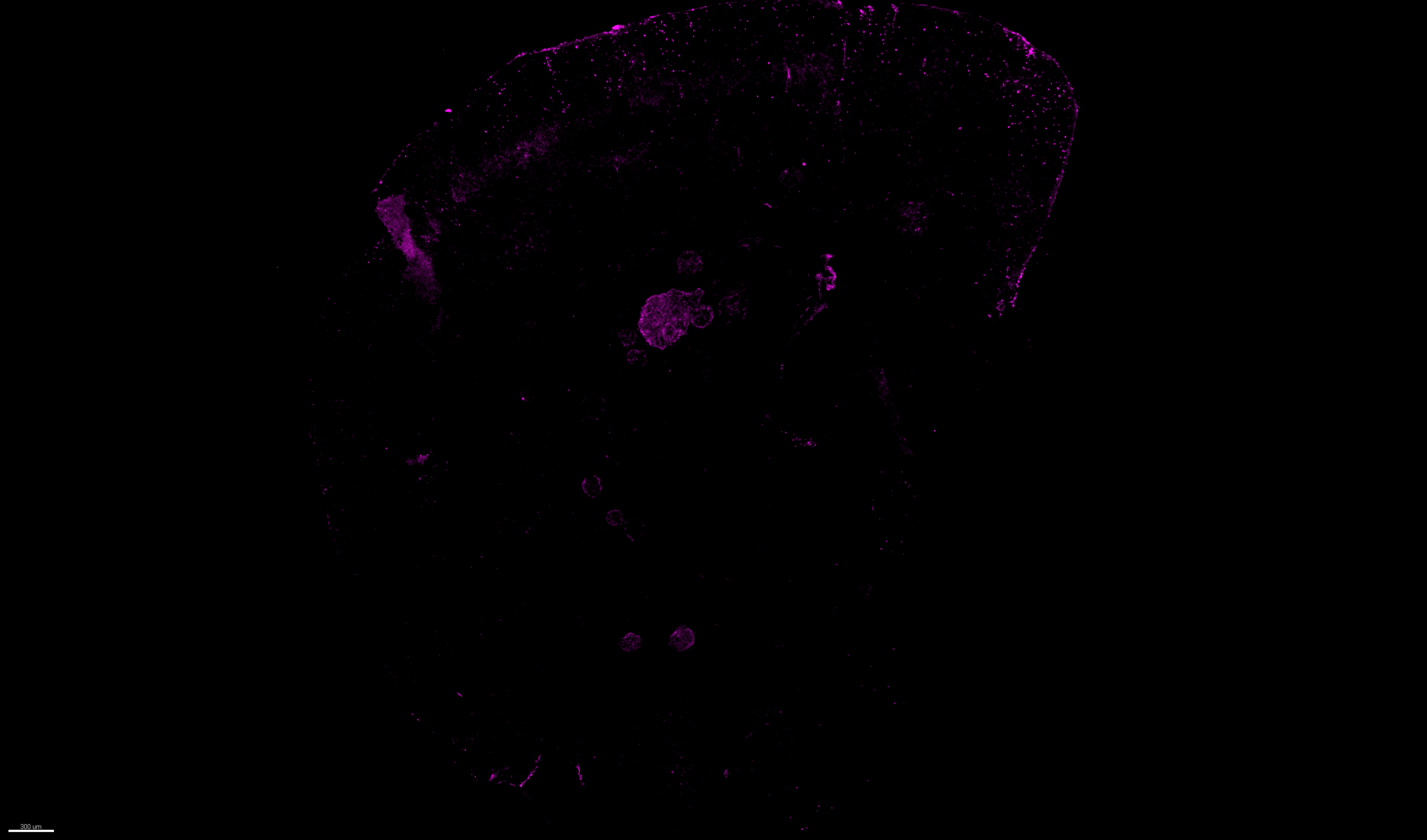

Supplement: Supplementary file 10 — Source data Fig. 5 [file 44319_2026_721_MOESM10_ESM.zip › 5G/Whole brain-Ms4a7.tif]

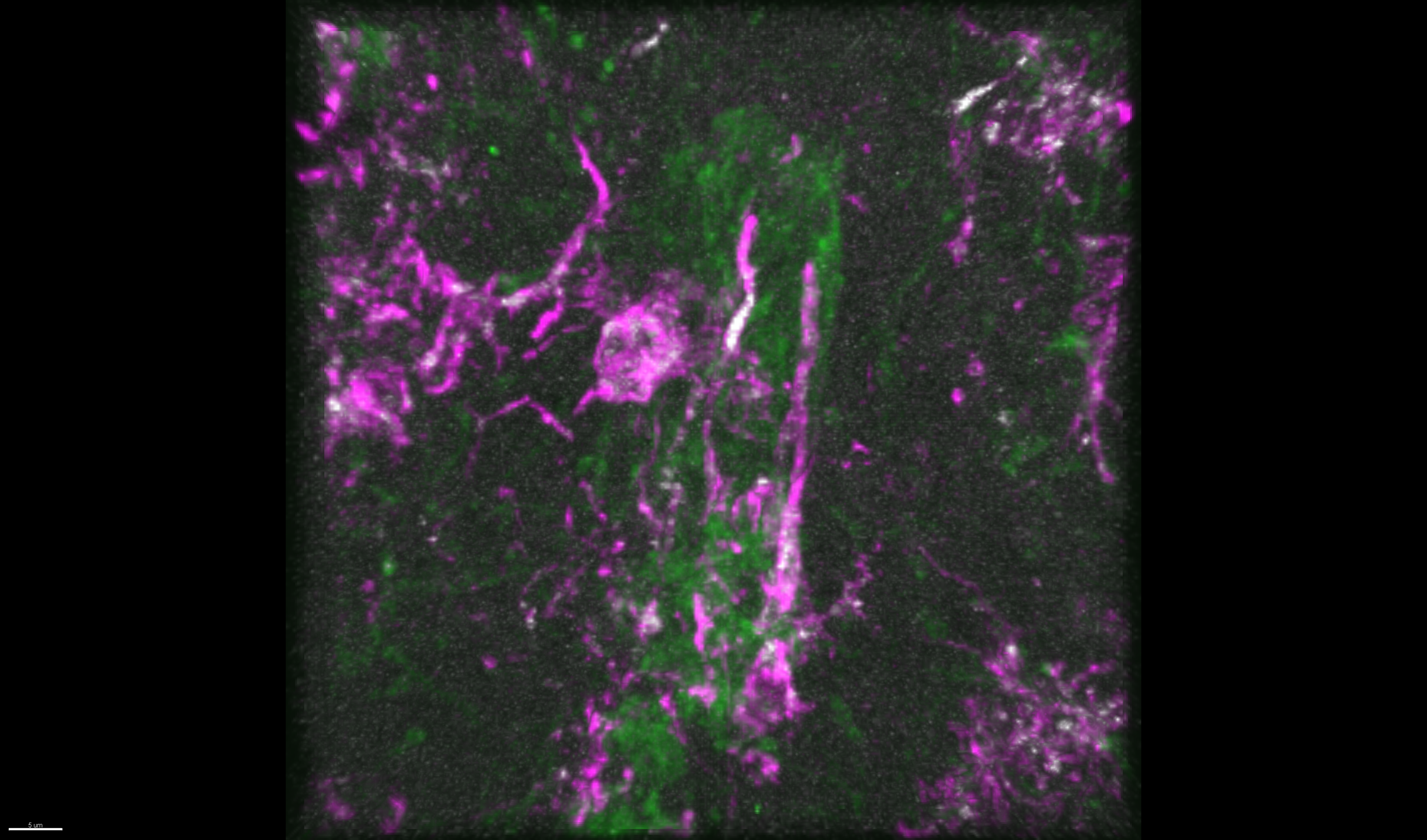

Supplement: Supplementary file 10 — Source data Fig. 5 [file 44319_2026_721_MOESM10_ESM.zip › 5H/KO-Images/Merged.tif]

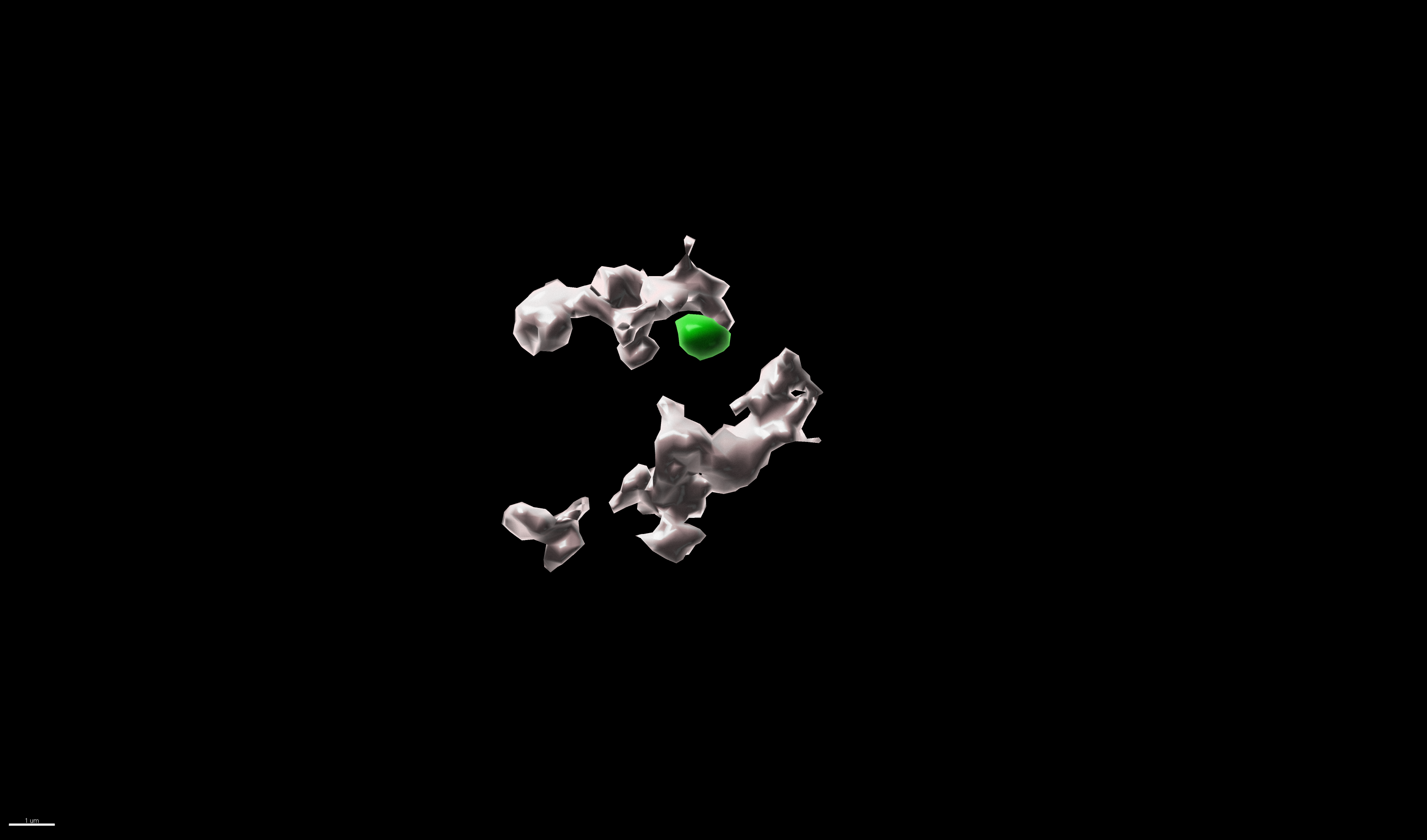

Supplement: Supplementary file 10 — Source data Fig. 5 [file 44319_2026_721_MOESM10_ESM.zip › 5H/KO-Images/3D recunstructions/MBP-CD68-surface.tif]

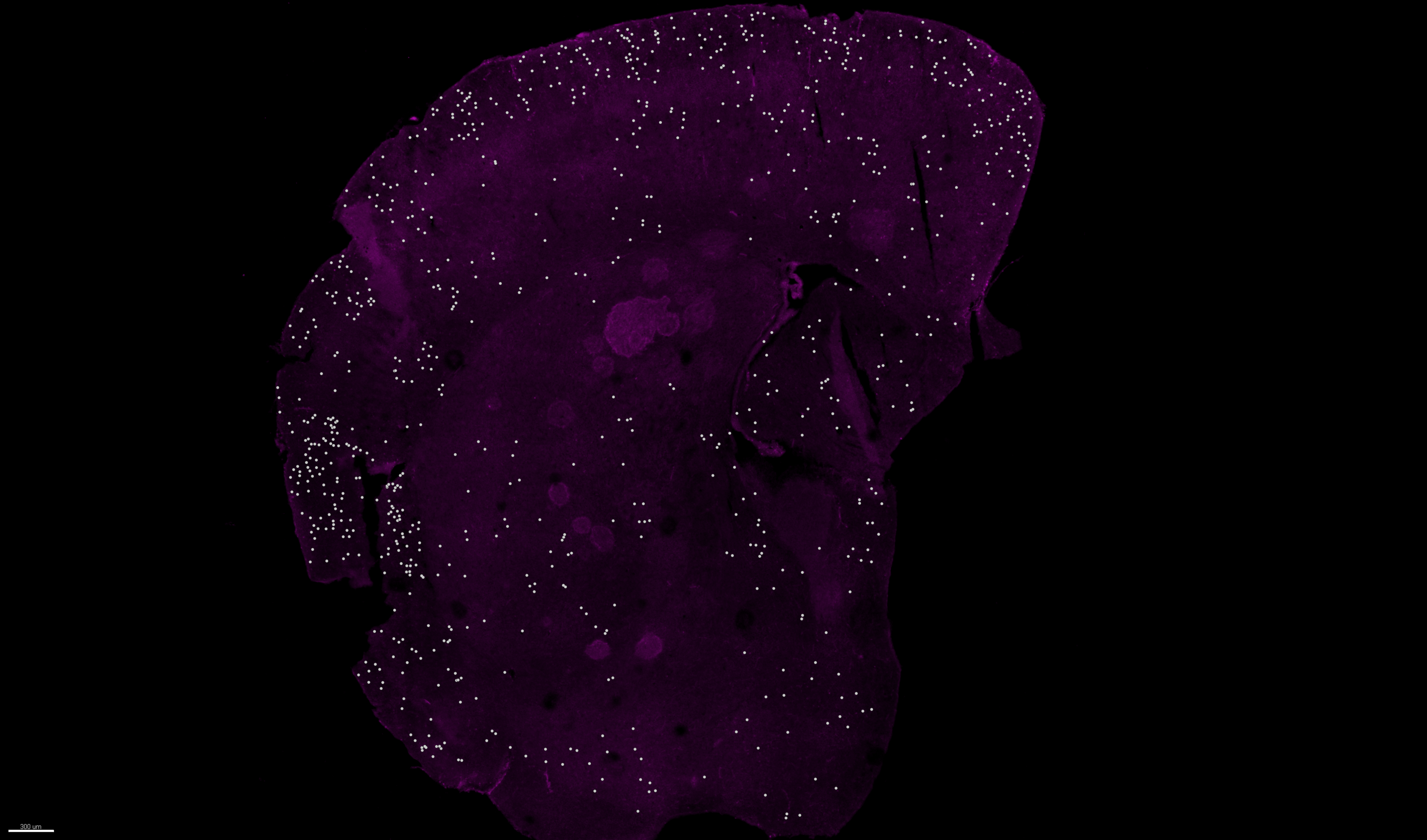

Supplement: Supplementary file 10 — Source data Fig. 5 [file 44319_2026_721_MOESM10_ESM.zip › 5G/Whole brain-original spotted Ms4a7.tif]

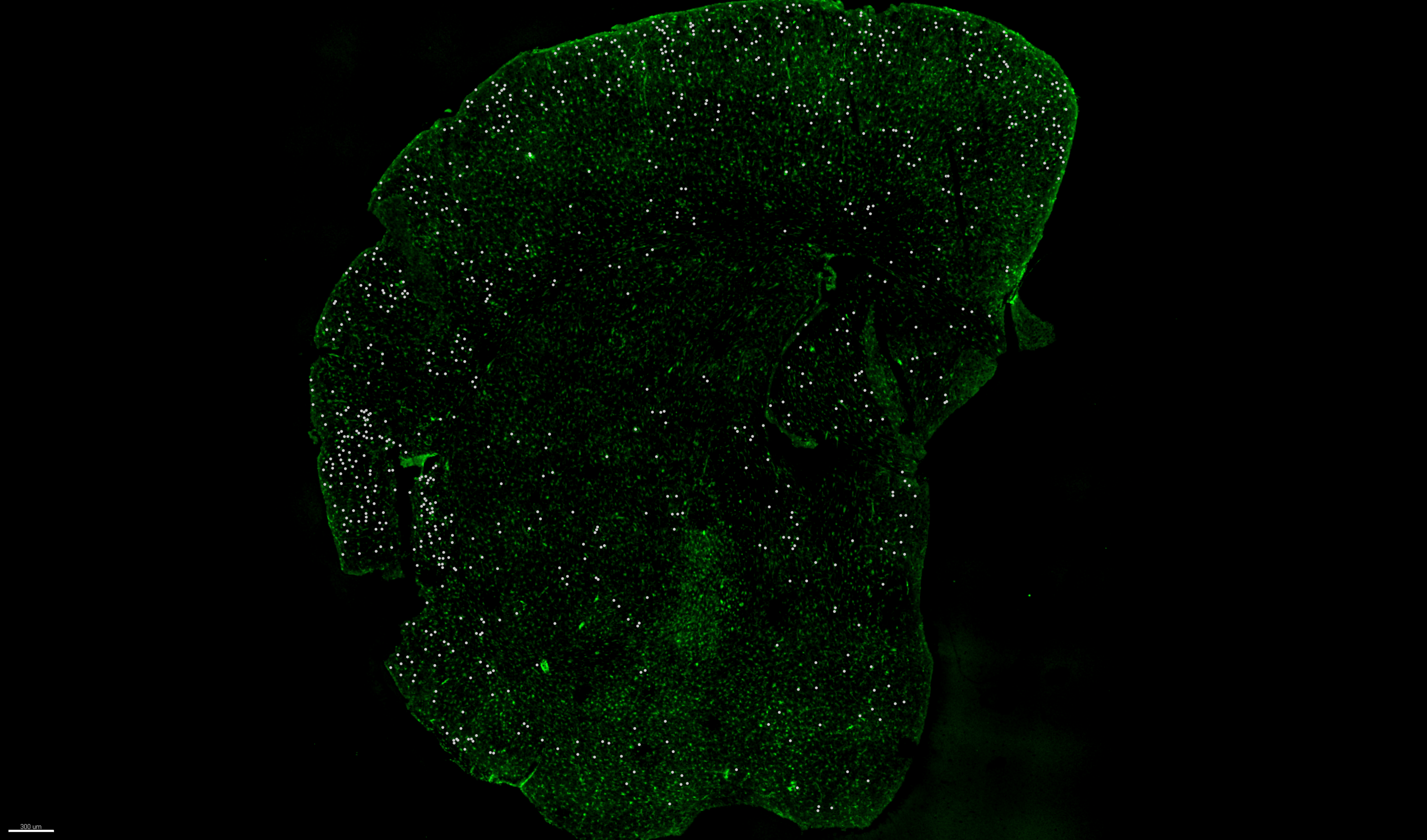

Supplement: Supplementary file 10 — Source data Fig. 5 [file 44319_2026_721_MOESM10_ESM.zip › 5G/Whole brain-IBA1-spotted Ms4a7.tif]

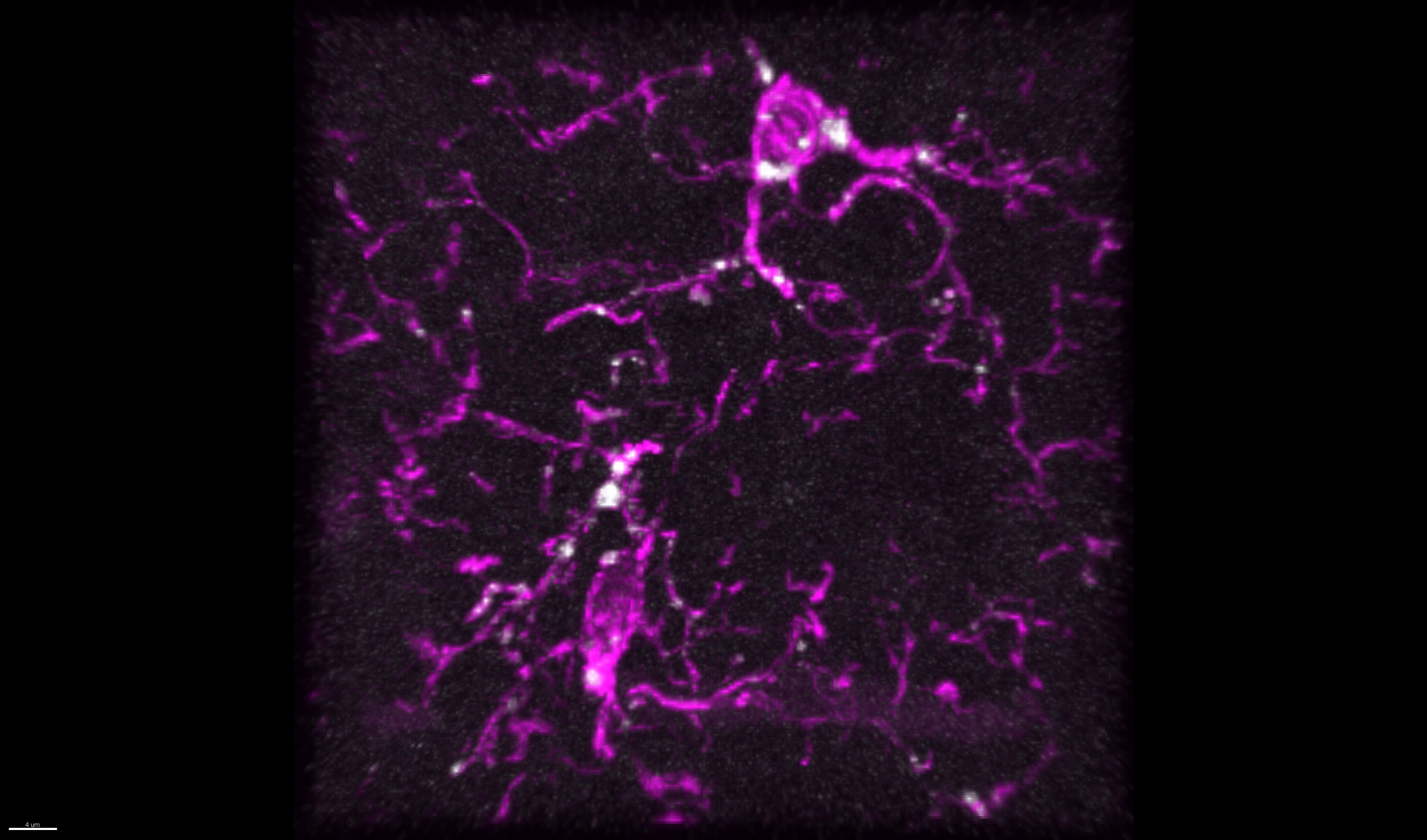

Supplement: Supplementary file 10 — Source data Fig. 5 [file 44319_2026_721_MOESM10_ESM.zip › 5H/Control-Images/IBA1-CD68.tif]

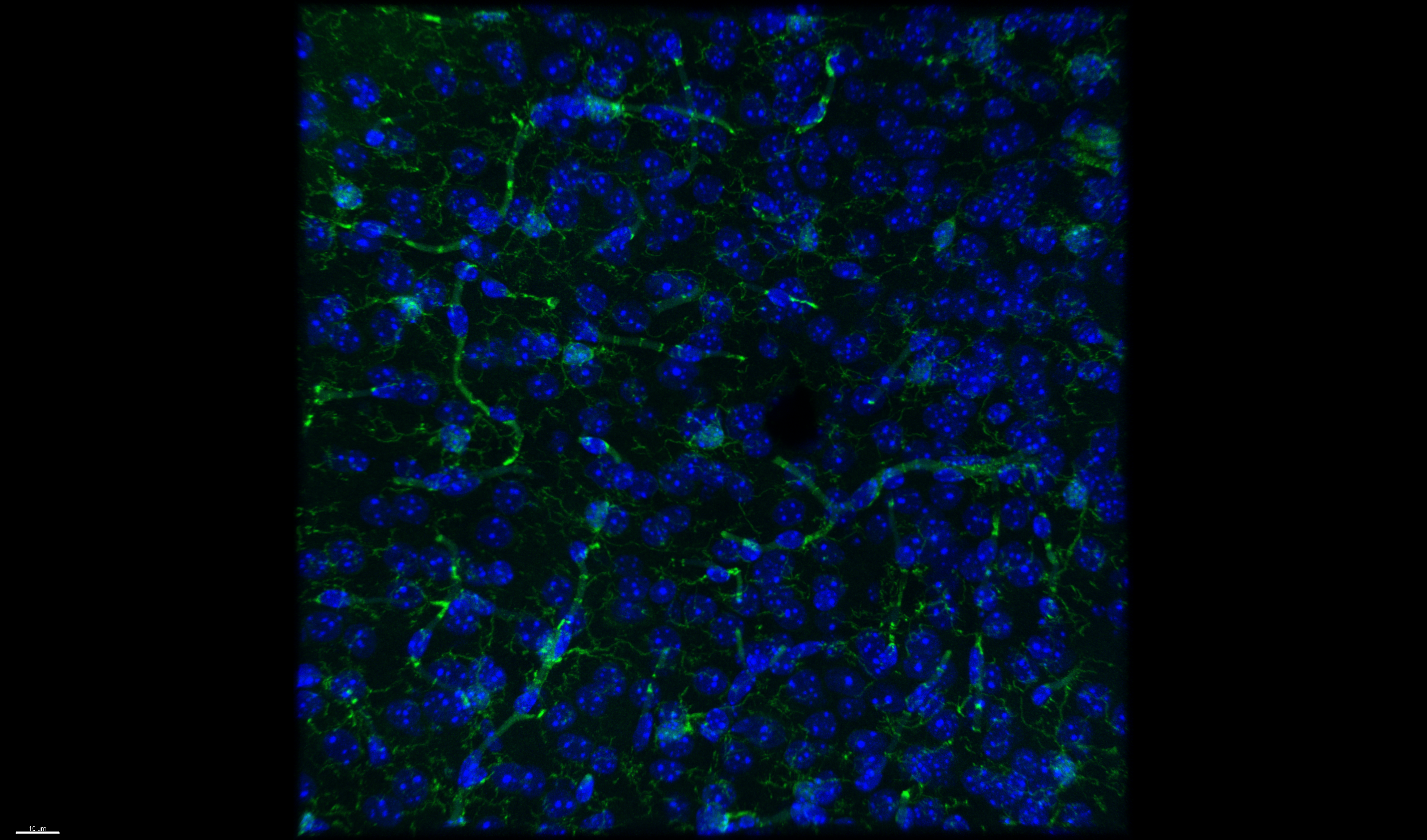

Supplement: Supplementary file 10 — Source data Fig. 5 [file 44319_2026_721_MOESM10_ESM.zip › 5F/Control/IBA1_[ii17_48_TileScan_001_Merging_Image_18]Crop1_2025-09-09T11-41-34.542.tif]

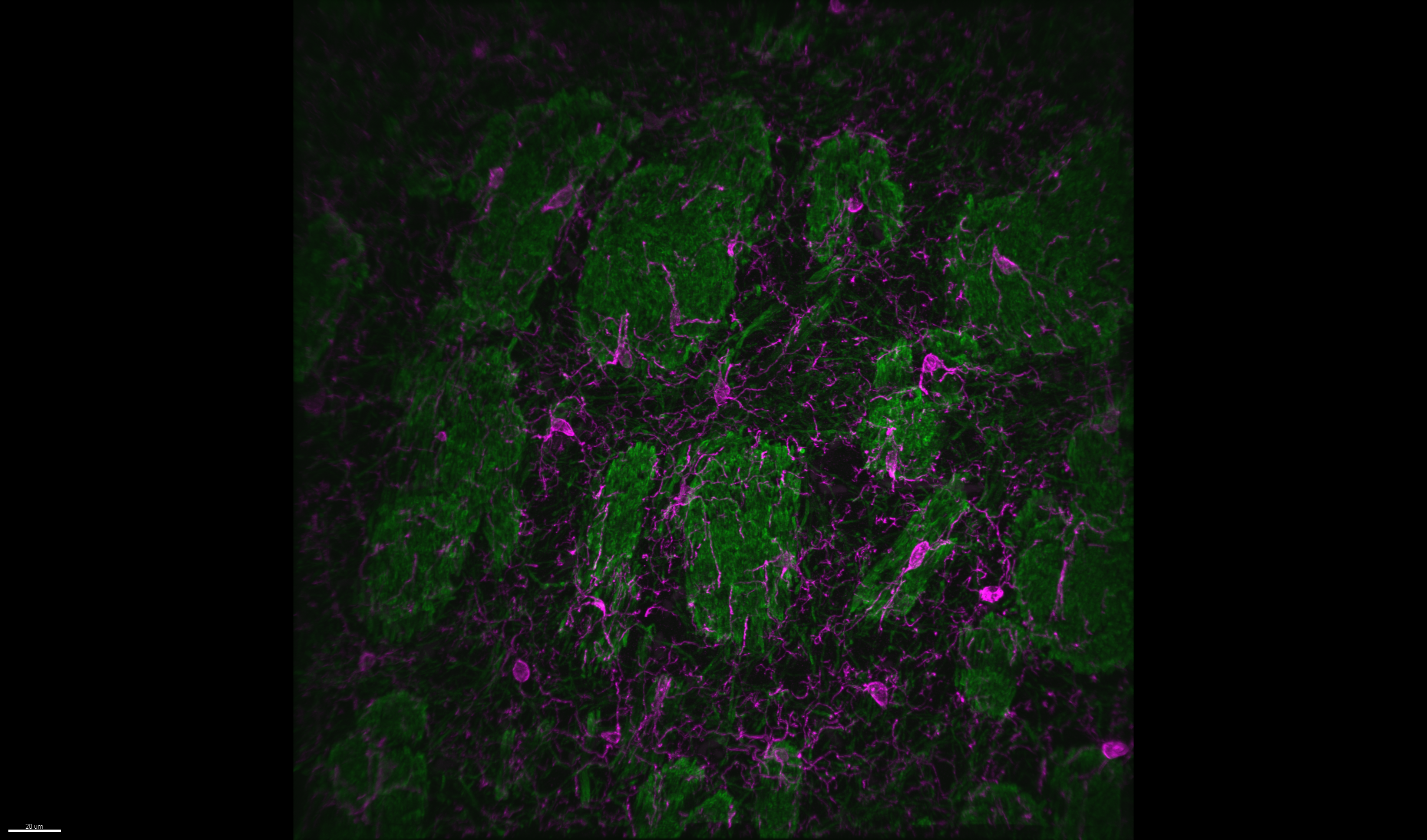

Supplement: Supplementary file 10 — Source data Fig. 5 [file 44319_2026_721_MOESM10_ESM.zip › 5H/Control-Images/Overview-1_01_2025-09-09T10-54-19.369.tif]

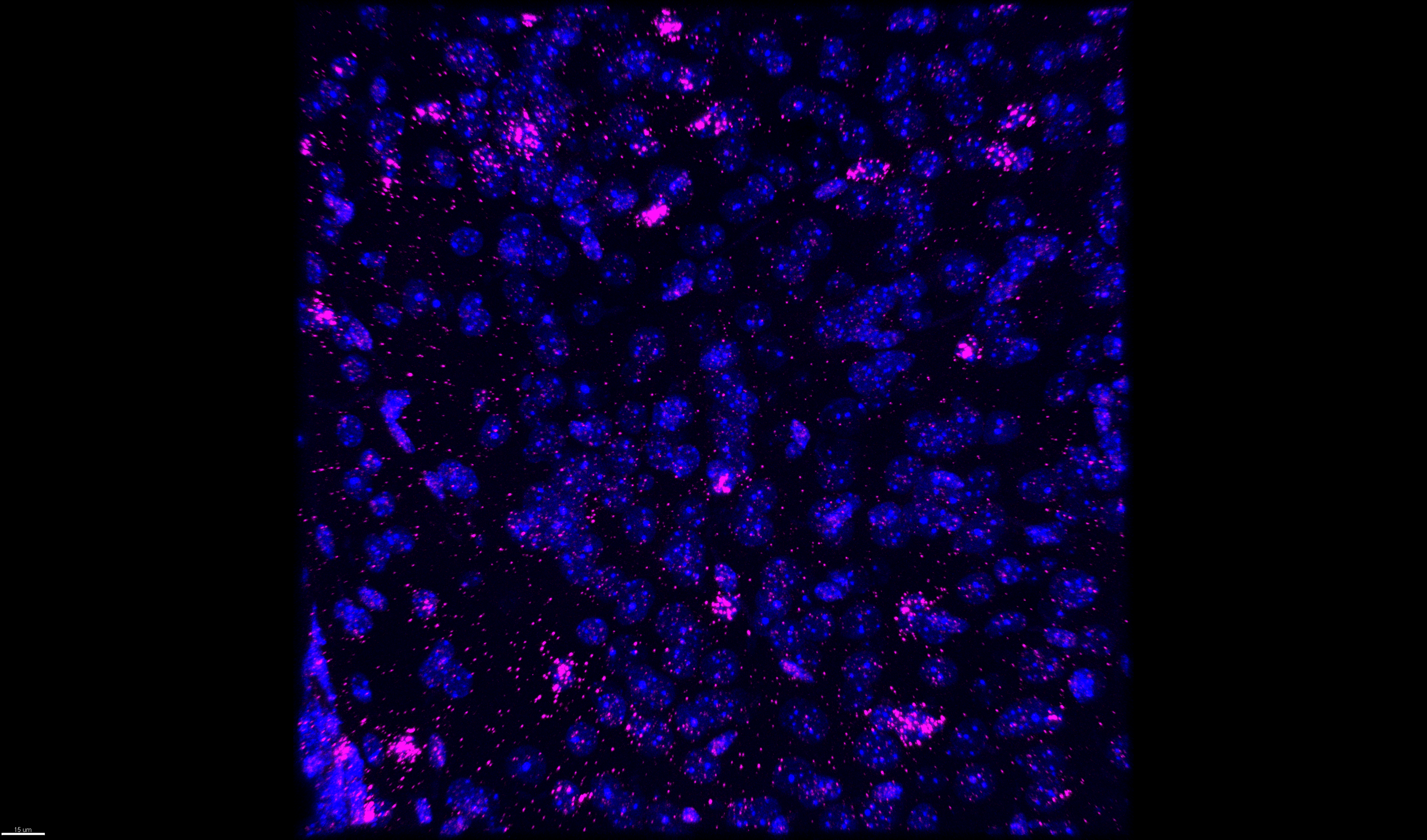

Supplement: Supplementary file 10 — Source data Fig. 5 [file 44319_2026_721_MOESM10_ESM.zip › 5F/KO/Overview/Ms4a7-56_[ii0_TileScan_001_(Stage_1_of_20)_Image_1]_2025-09-09T11-57-14.973.tif]

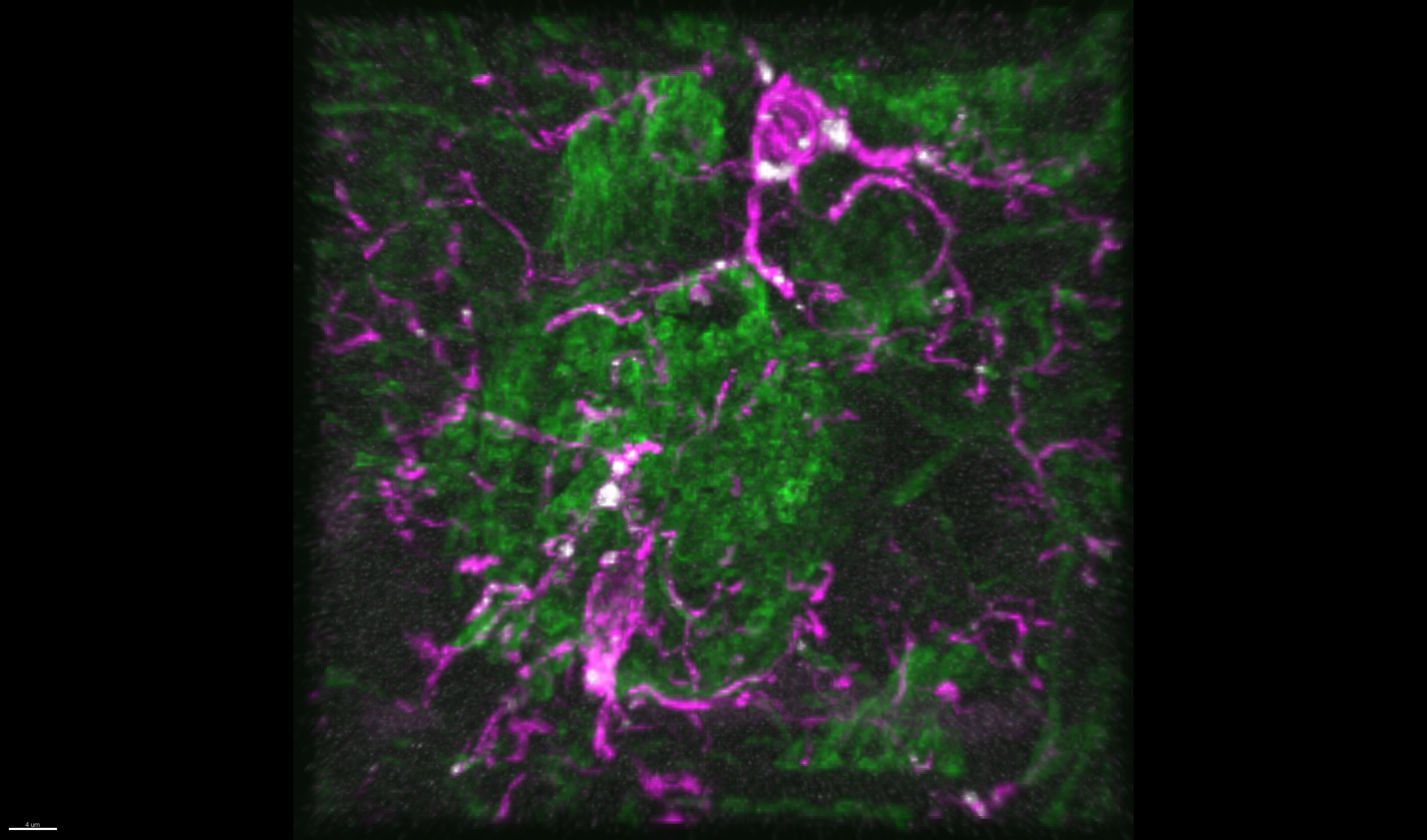

Supplement: Supplementary file 10 — Source data Fig. 5 [file 44319_2026_721_MOESM10_ESM.zip › 5H/Control-Images/Merge.tif]
