# Supplementary figures and images for: The Arp2/3 complex controls the development of homeostatic microglia (part 2 of 2)
Source: EMBO Rep. 2026 Feb 27;27(7):1696–719. doi: 10.1038/s44319-026-00721-8 (PMC13076794; doi:10.1038/s44319-026-00721-8)

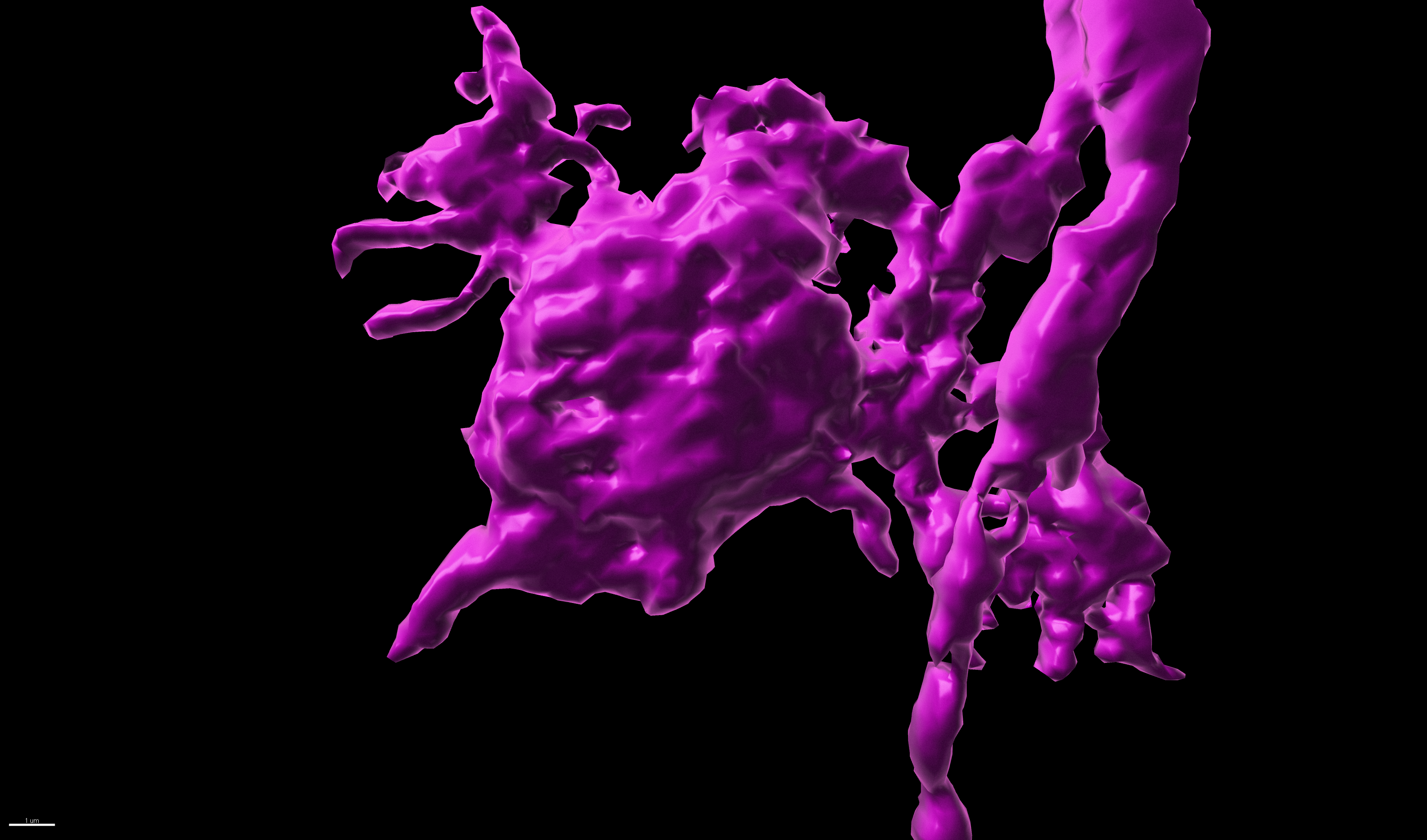

Supplement: Supplementary file 10 — Source data Fig. 5 [file 44319_2026_721_MOESM10_ESM.zip › 5H/KO-Images/3D recunstructions/IBA1-surface.tif]

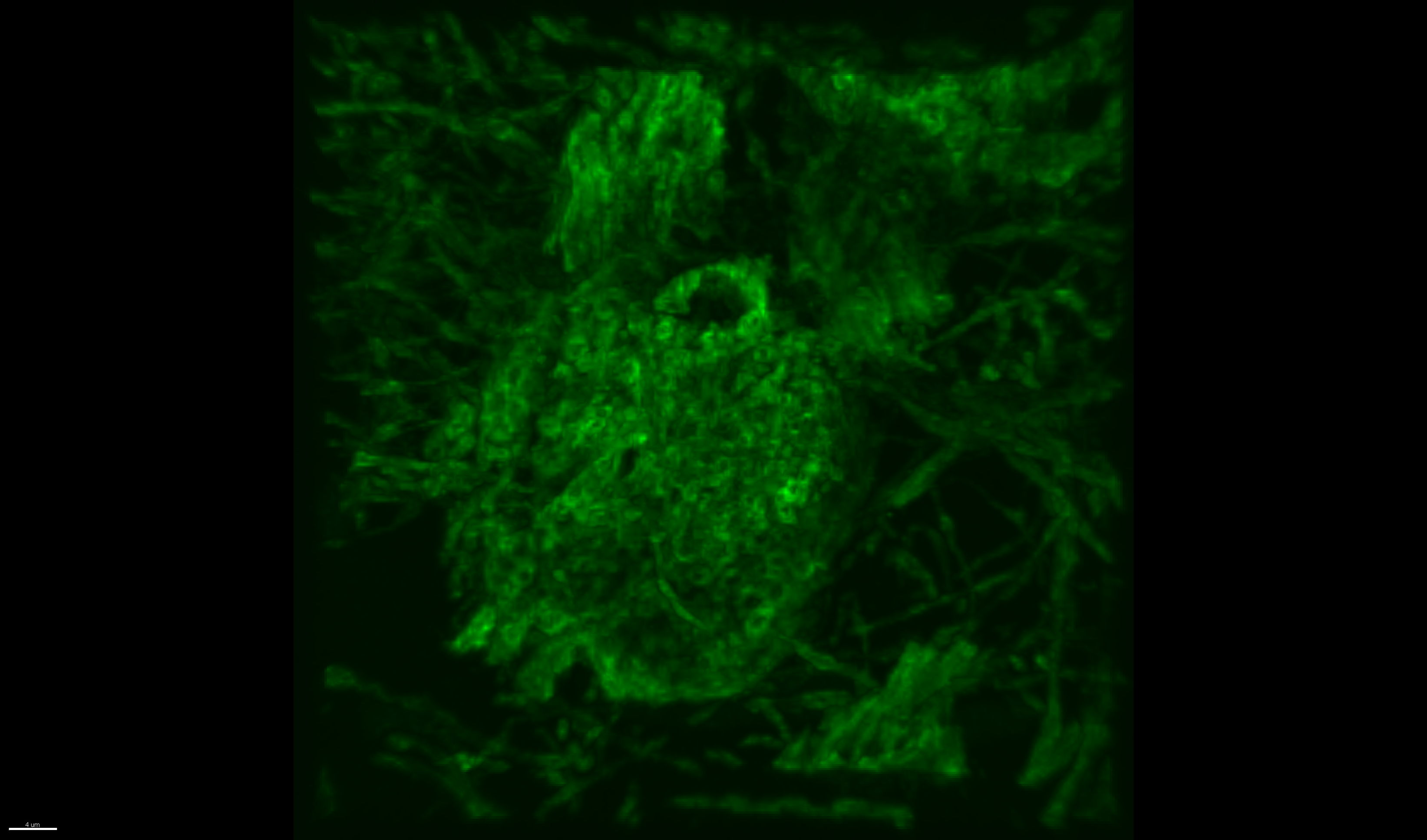

Supplement: Supplementary file 10 — Source data Fig. 5 [file 44319_2026_721_MOESM10_ESM.zip › 5H/Control-Images/MBP.tif]

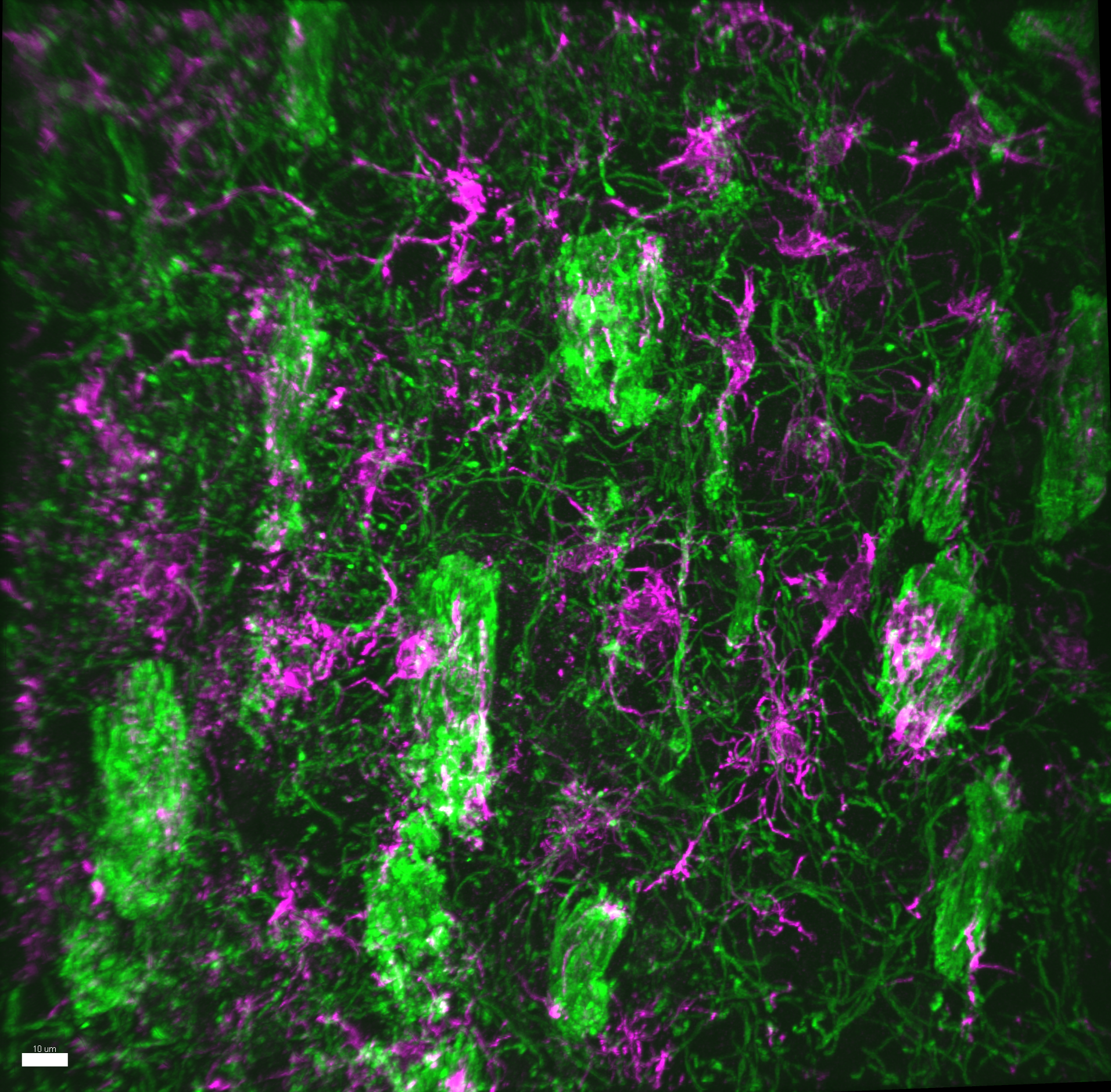

Supplement: Supplementary file 10 — Source data Fig. 5 [file 44319_2026_721_MOESM10_ESM.zip › 5H/KO-Images/Overview_1_03_2025-04-22T11-23-13.361.tif]

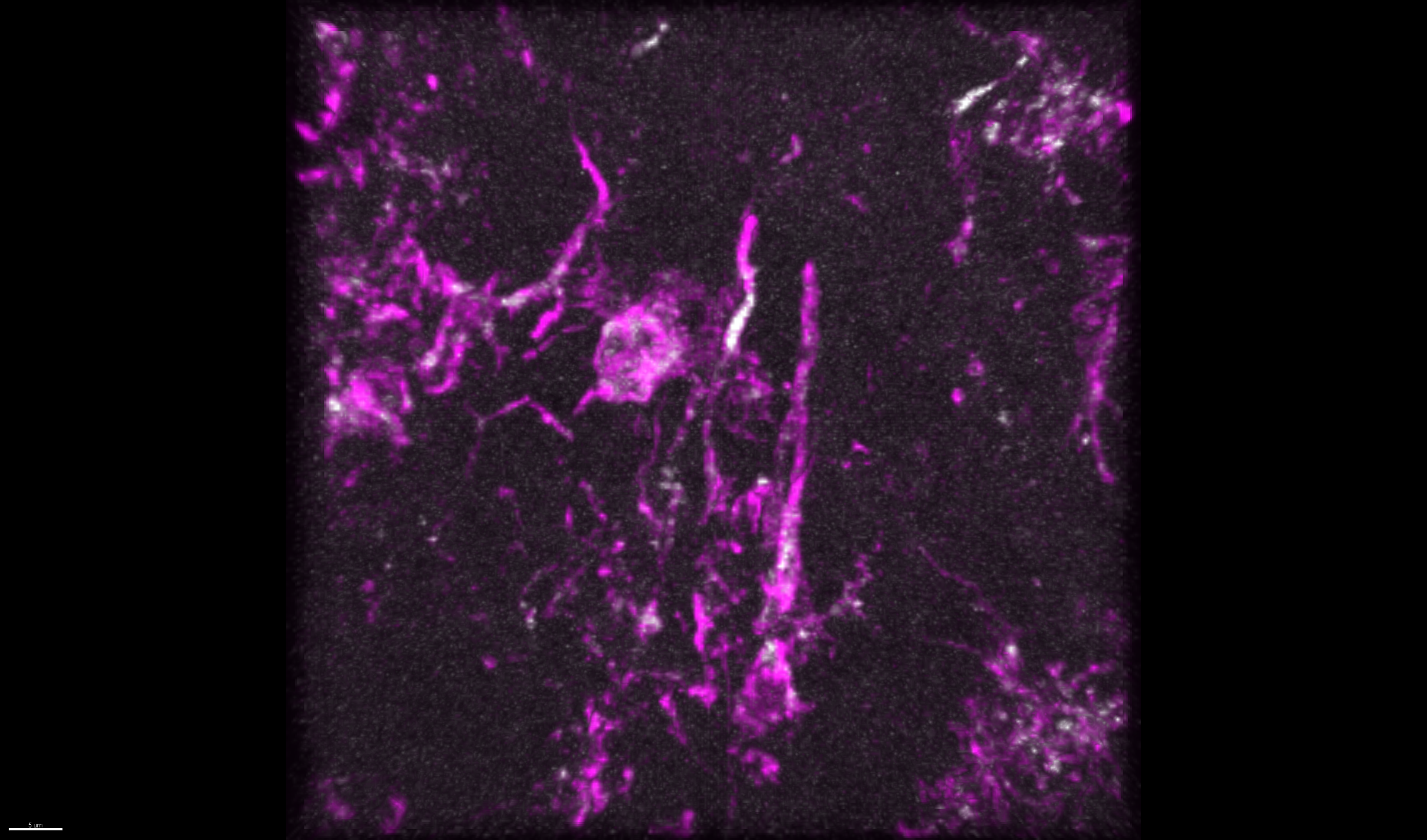

Supplement: Supplementary file 10 — Source data Fig. 5 [file 44319_2026_721_MOESM10_ESM.zip › 5H/KO-Images/IBA1-CD68.tif]

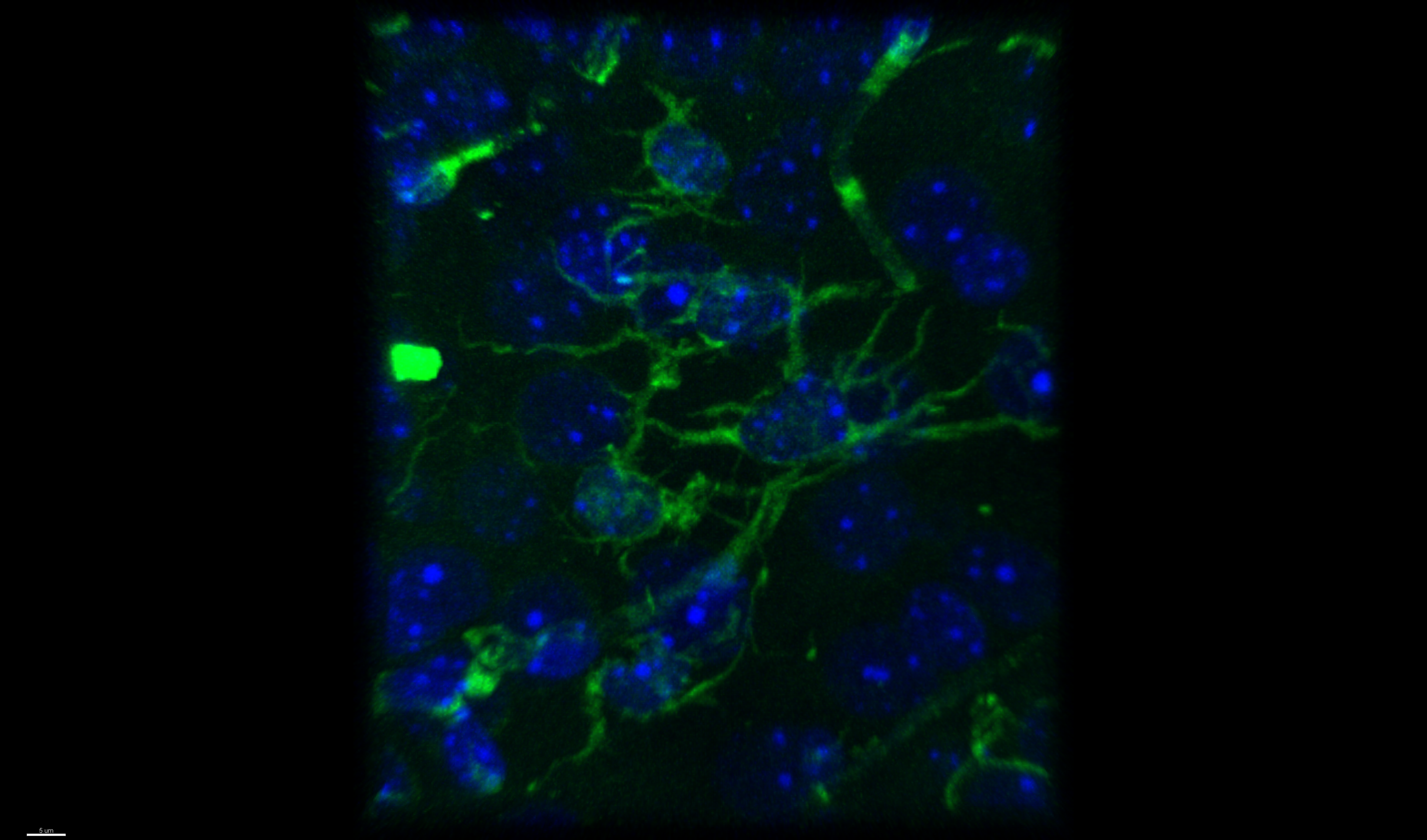

Supplement: Supplementary file 10 — Source data Fig. 5 [file 44319_2026_721_MOESM10_ESM.zip › 5F/KO/Zoom-in/IBA1-56_[ii20_TileScan_001_Merging_Image_21]-Crop1_2025-09-09T11-46-09.145.tif]

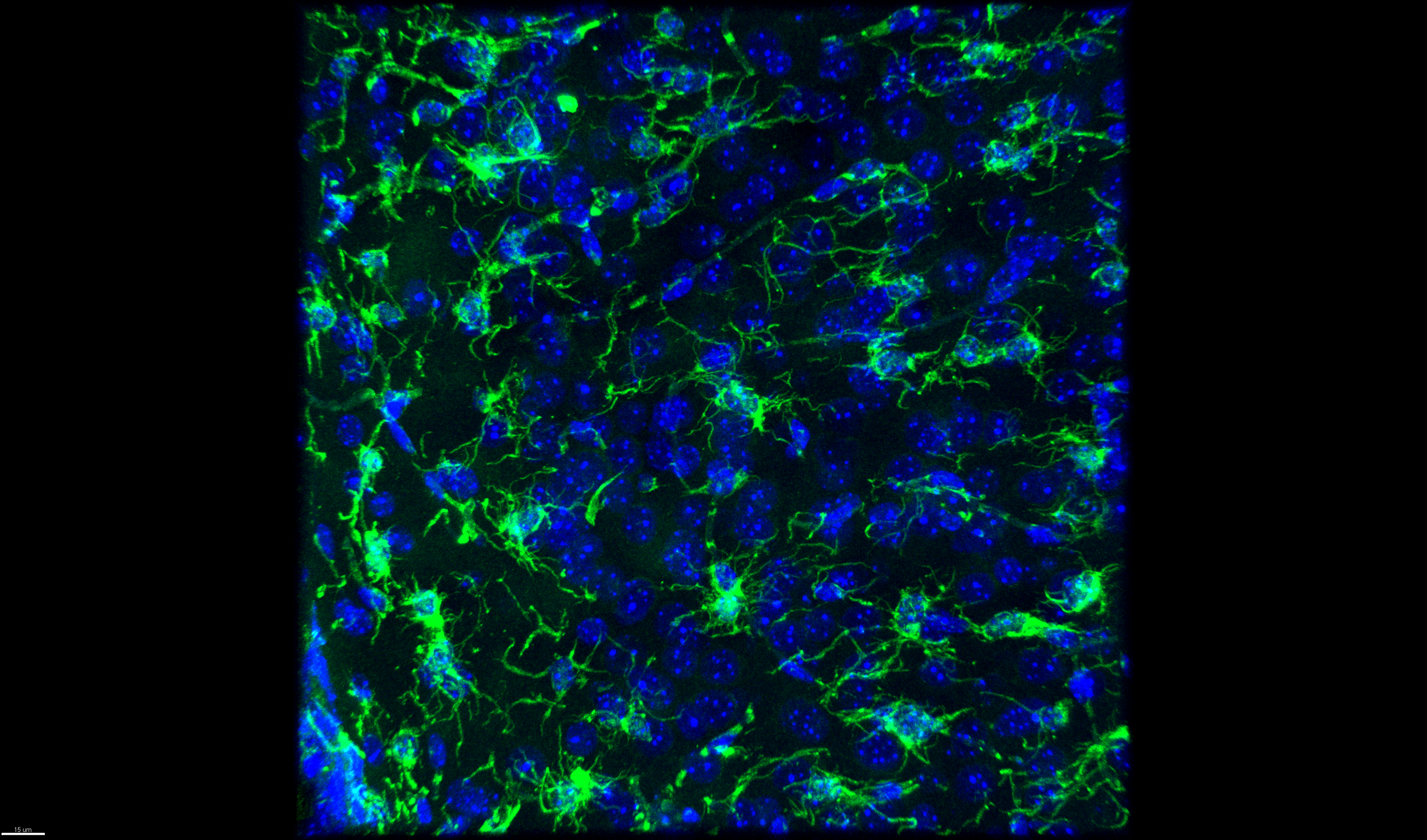

Supplement: Supplementary file 10 — Source data Fig. 5 [file 44319_2026_721_MOESM10_ESM.zip › 5F/KO/Overview/IBA1-56_[ii0_TileScan_001_(Stage_1_of_20)_Image_1]_2025-09-09T11-57-20.767.tif]

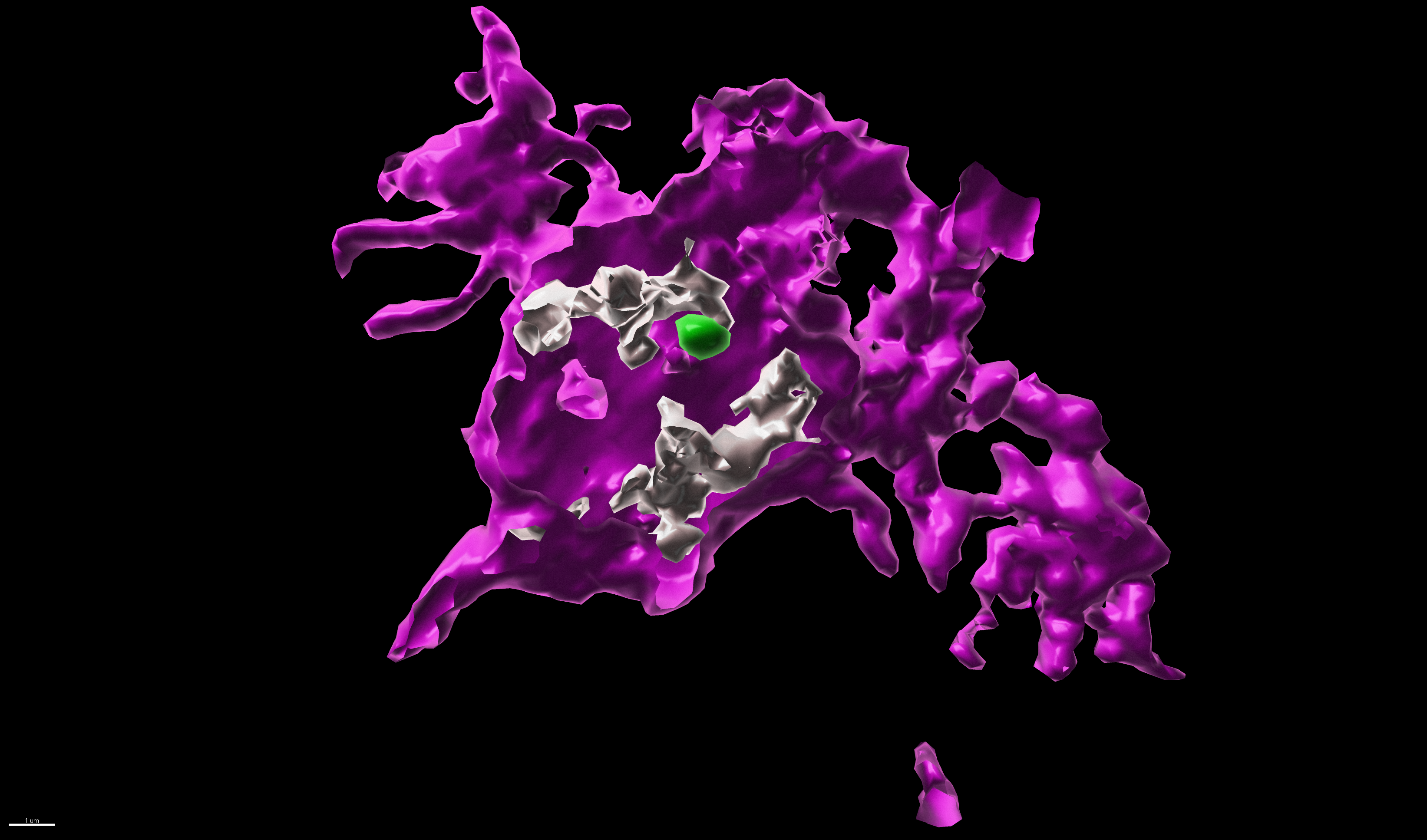

Supplement: Supplementary file 10 — Source data Fig. 5 [file 44319_2026_721_MOESM10_ESM.zip › 5H/KO-Images/3D recunstructions/Merge-Clipped.tif]

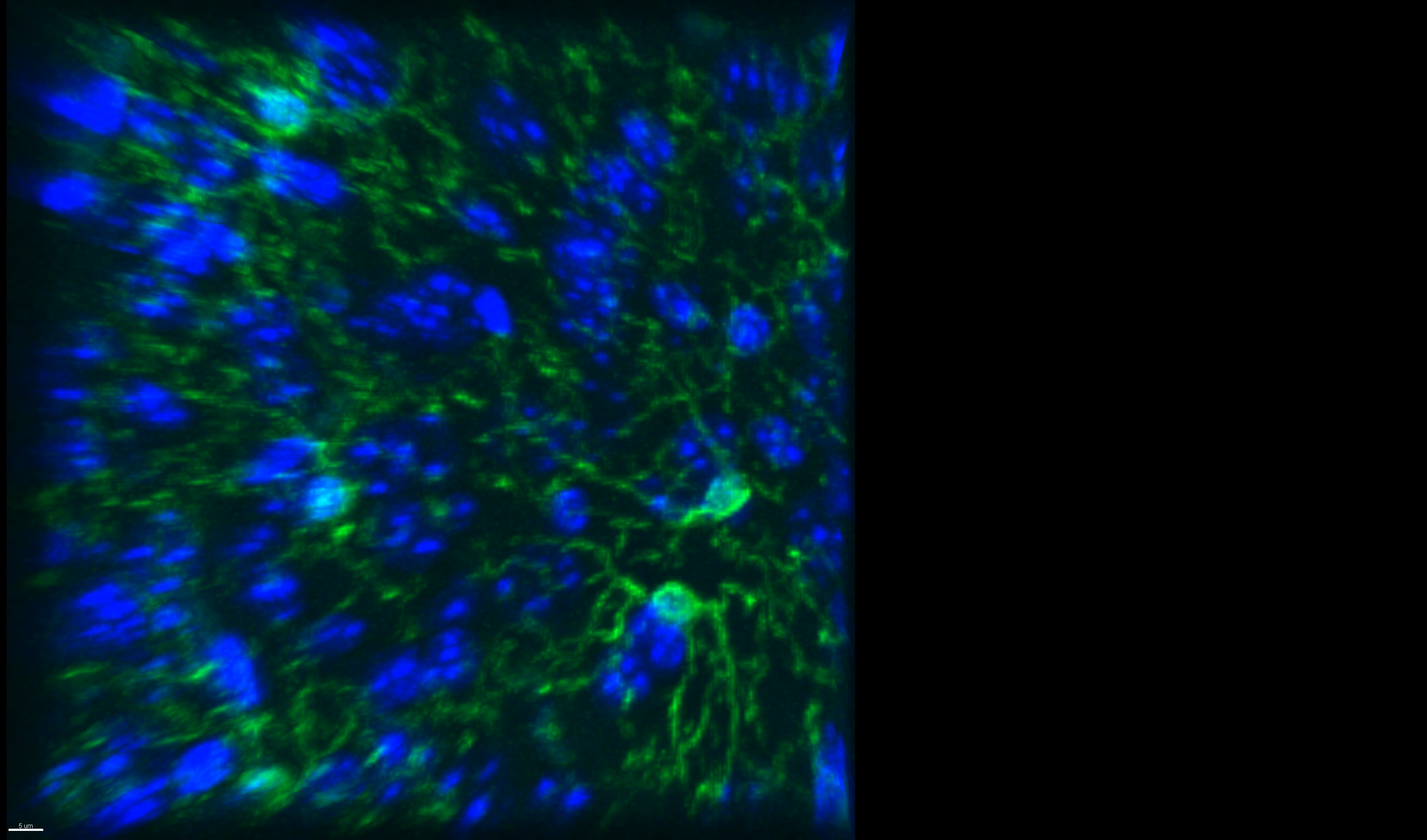

Supplement: Supplementary file 11 — Source data Fig. 6 [file 44319_2026_721_MOESM11_ESM.zip › 6D/Control-Images/IBA1.tif]

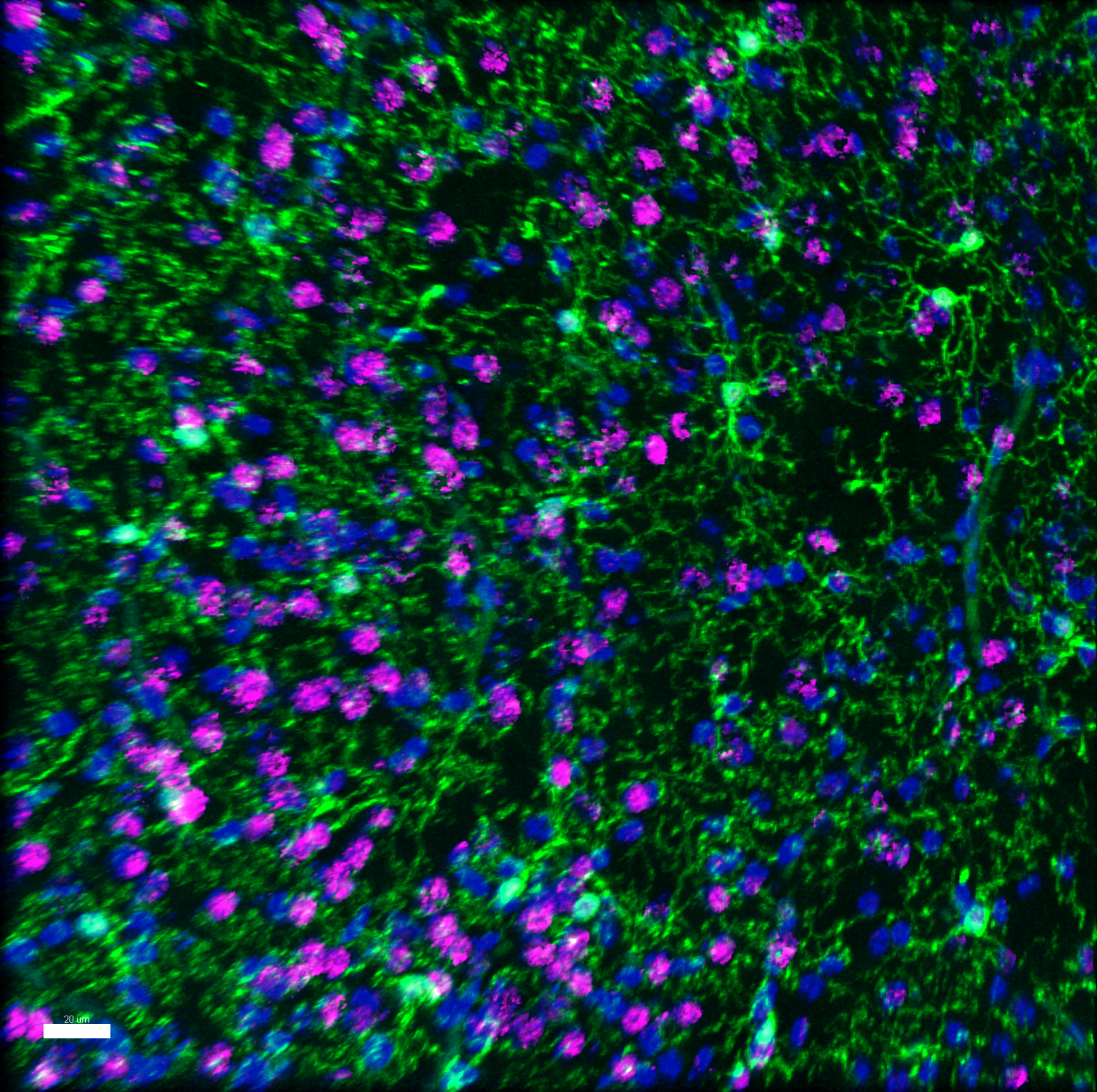

Supplement: Supplementary file 11 — Source data Fig. 6 [file 44319_2026_721_MOESM11_ESM.zip › 6D/Control-Images/Overview-control.tif]

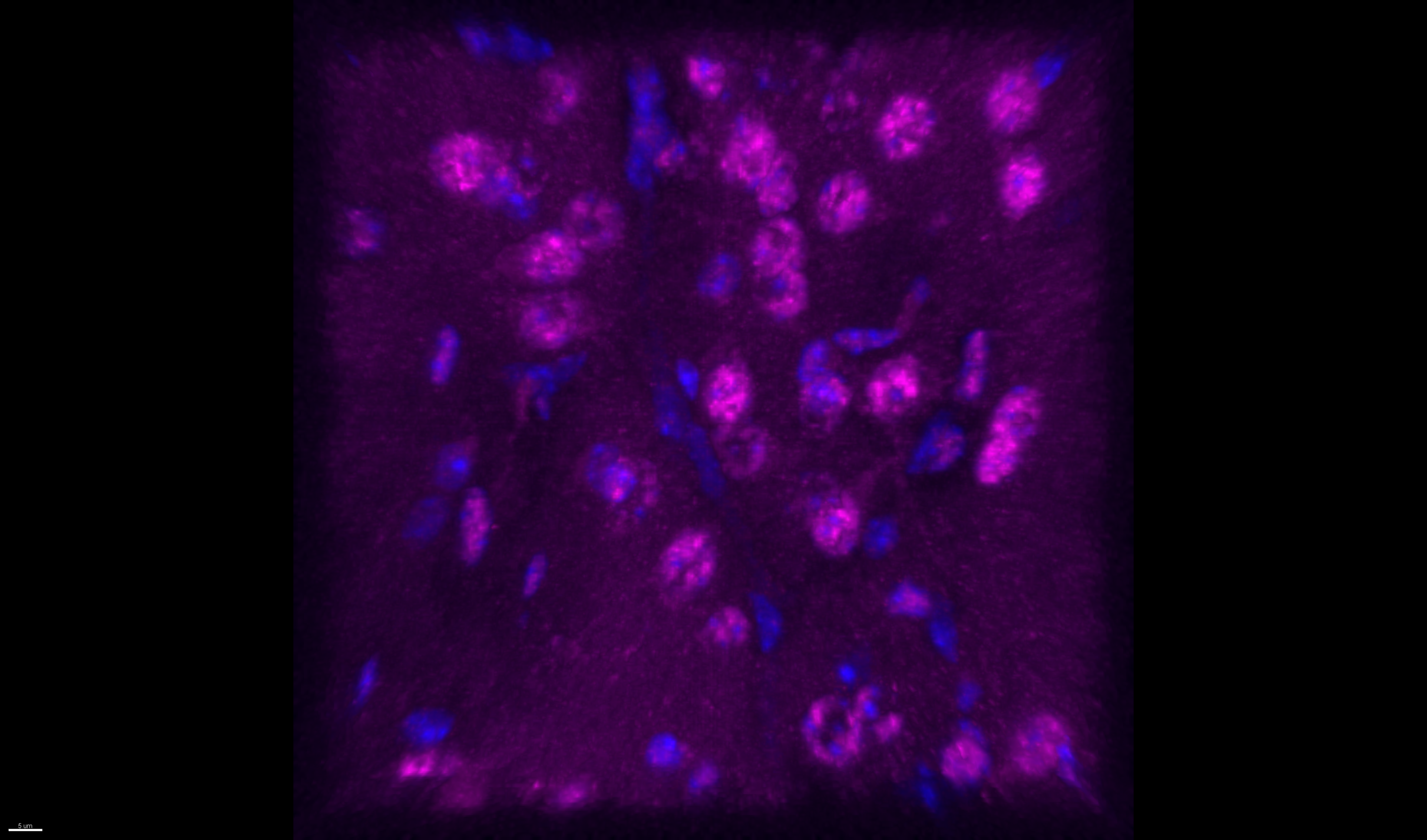

Supplement: Supplementary file 11 — Source data Fig. 6 [file 44319_2026_721_MOESM11_ESM.zip › 6D/KO-Images/pSMAD3.tif]

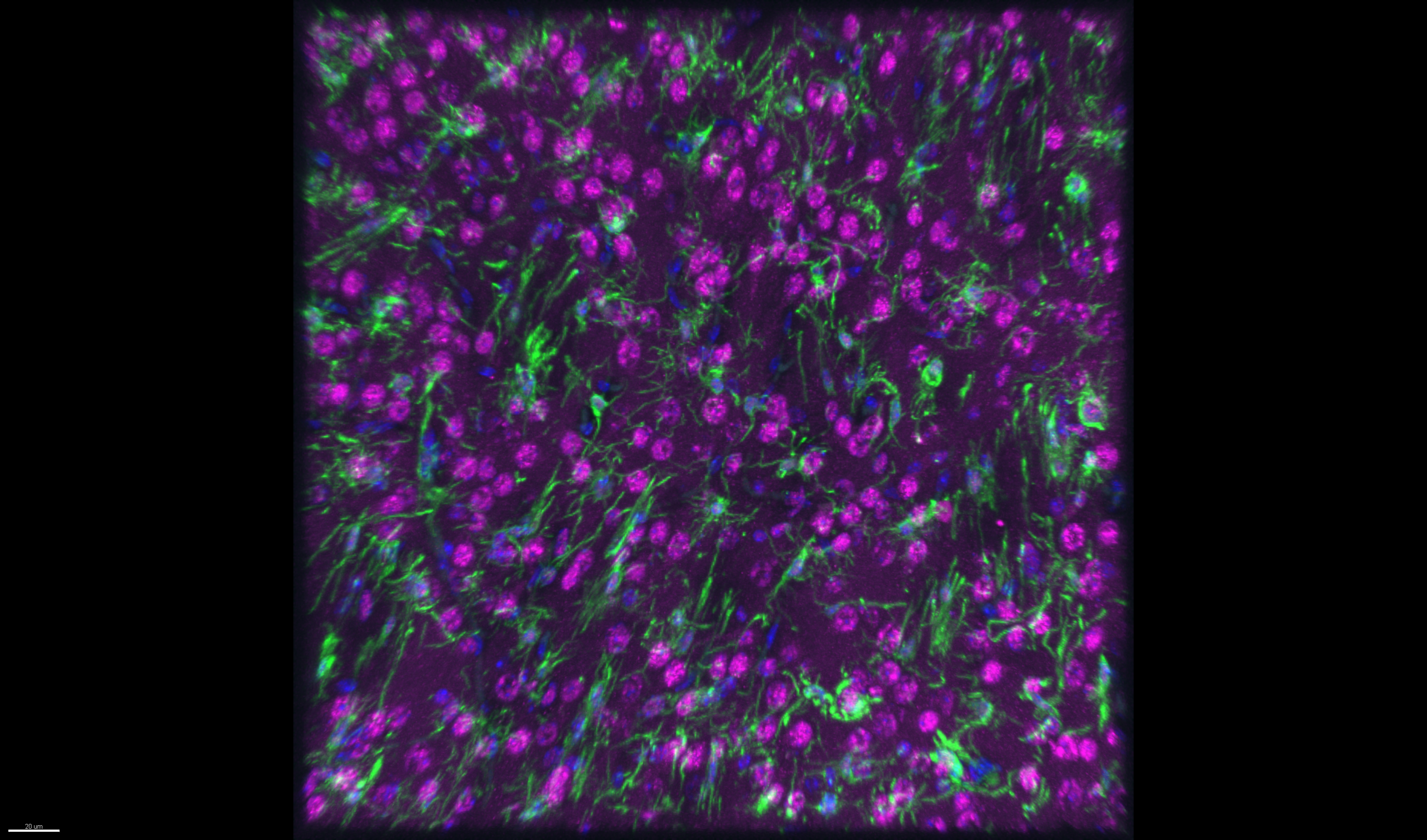

Supplement: Supplementary file 11 — Source data Fig. 6 [file 44319_2026_721_MOESM11_ESM.zip › 6D/KO-Images/Overview-KO.tif]

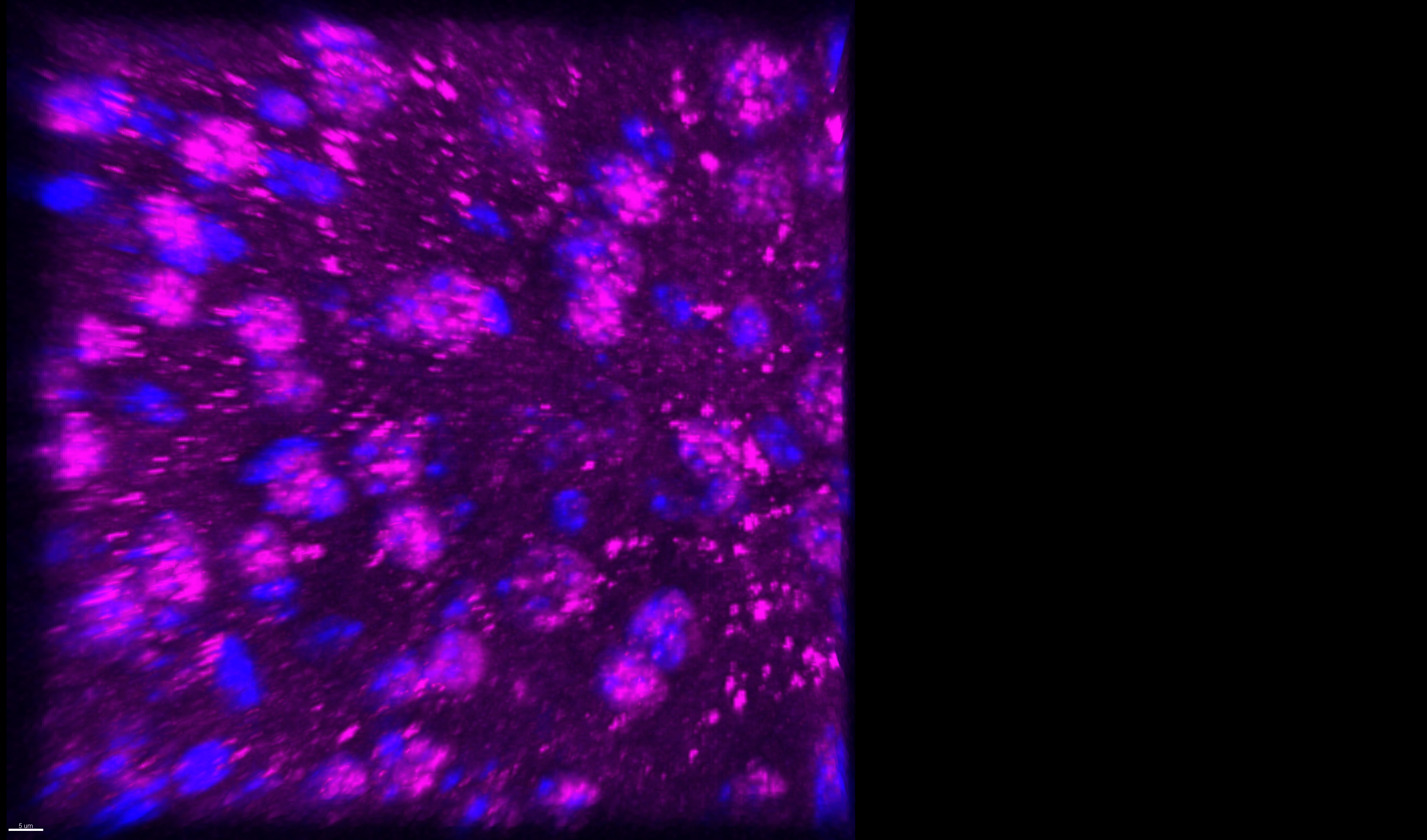

Supplement: Supplementary file 11 — Source data Fig. 6 [file 44319_2026_721_MOESM11_ESM.zip › 6D/Control-Images/pSMAD3original.tif]

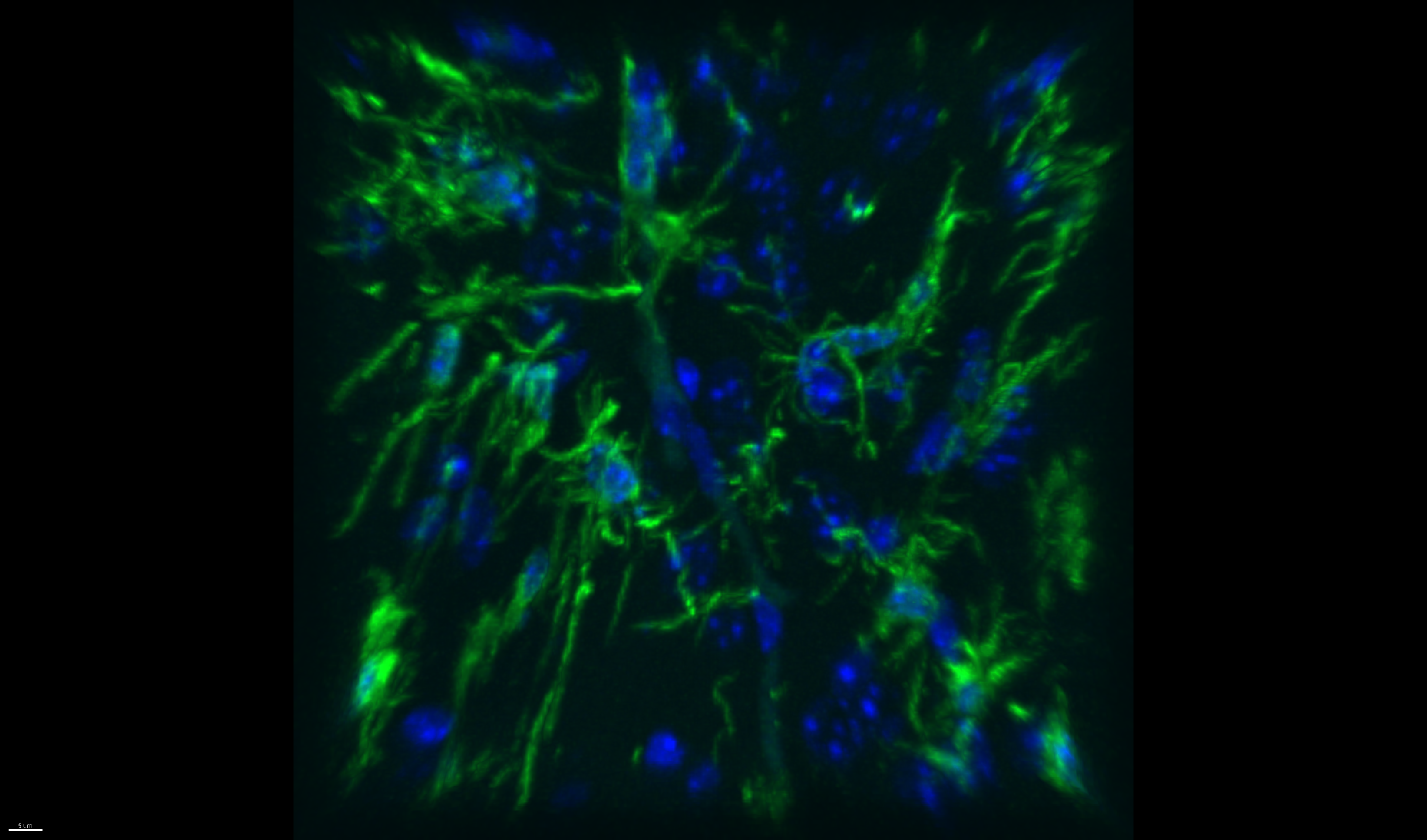

Supplement: Supplementary file 11 — Source data Fig. 6 [file 44319_2026_721_MOESM11_ESM.zip › 6D/KO-Images/IBA1.tif]

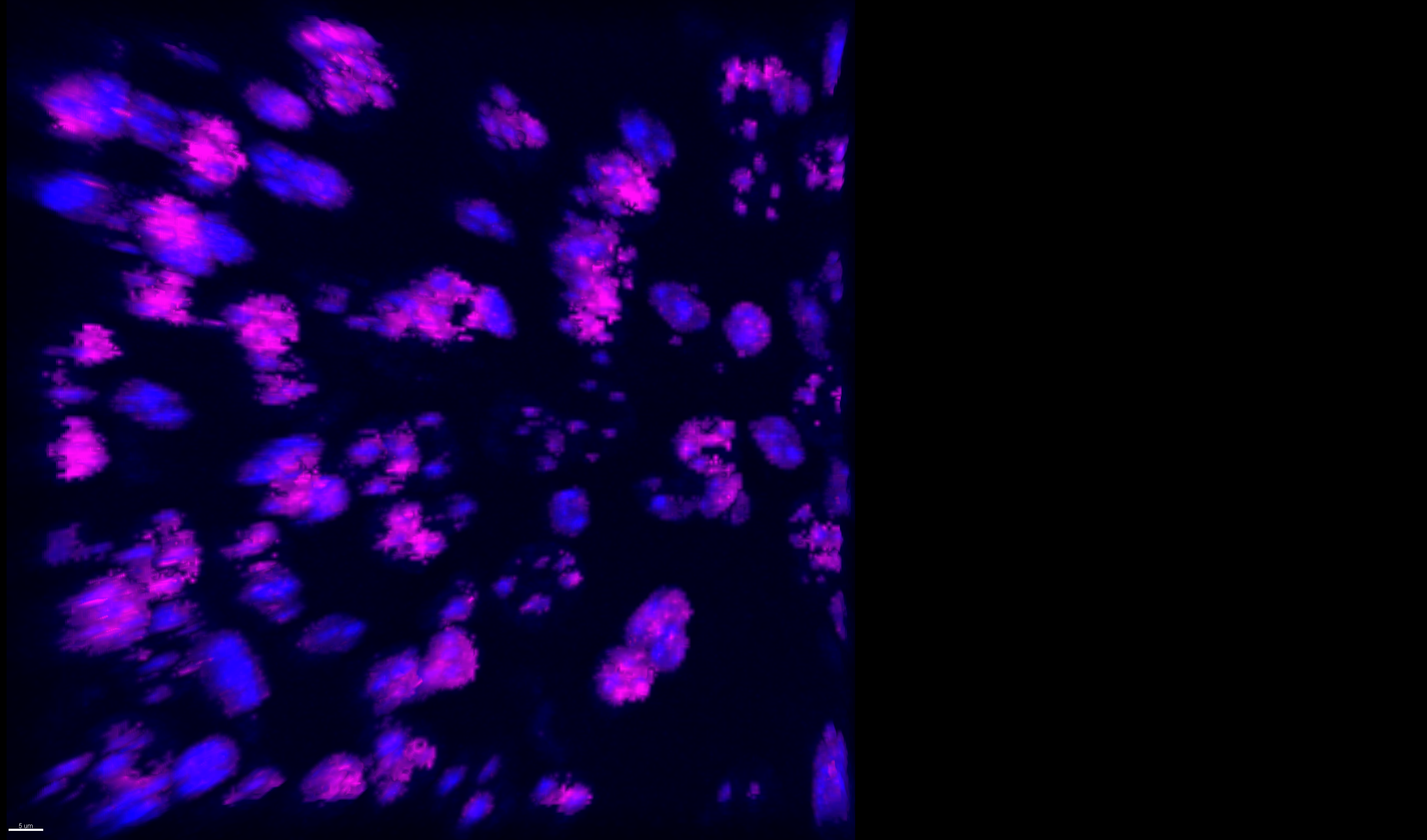

Supplement: Supplementary file 11 — Source data Fig. 6 [file 44319_2026_721_MOESM11_ESM.zip › 6D/Control-Images/pSMAD3-masked.tif]

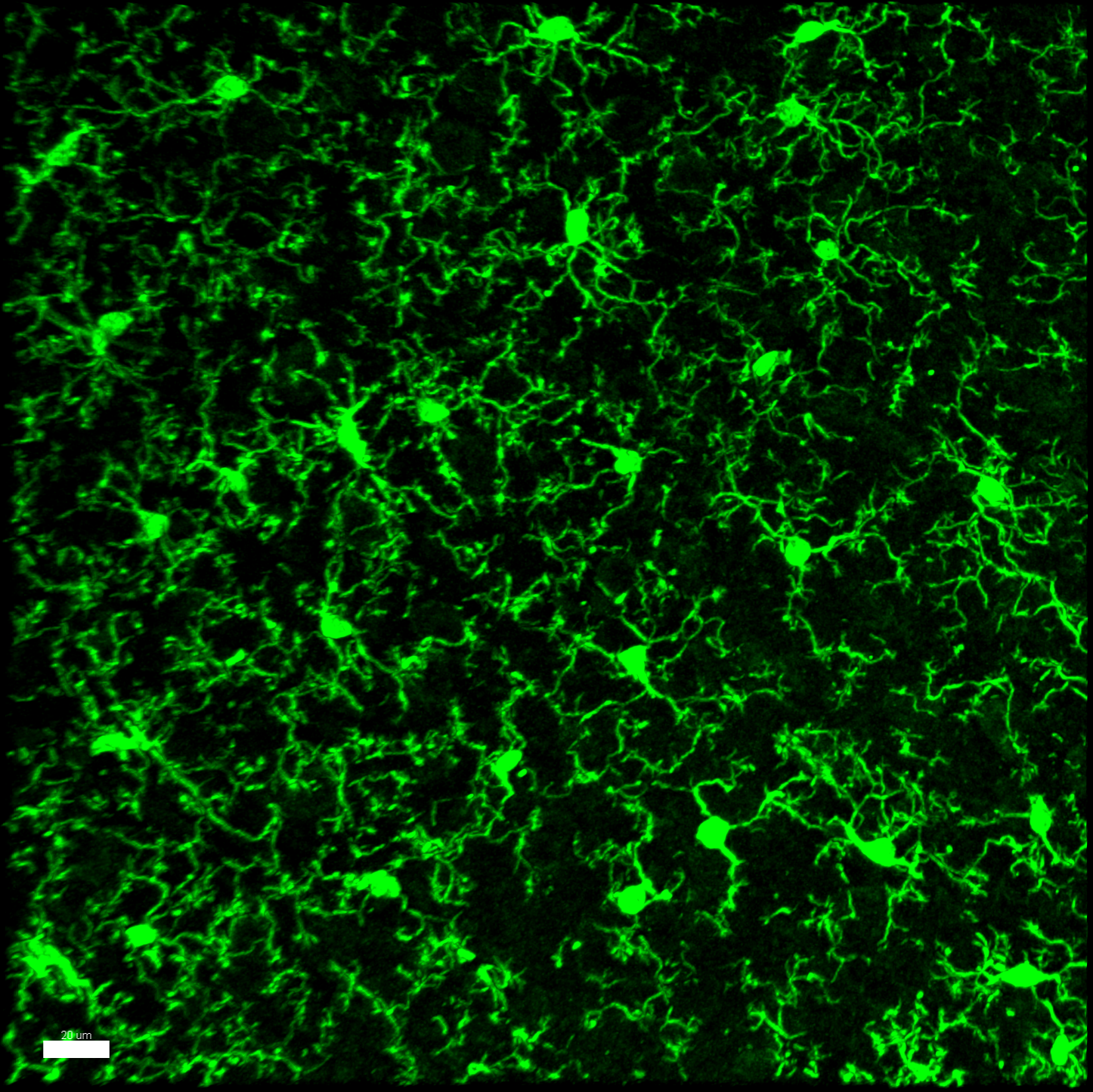

Supplement: Supplementary file 12 — Figure EV1 Source Data [file 44319_2026_721_MOESM12_ESM.zip › Figure EV1/EV1D/Cre-Arpc4floxedoverview.tif]

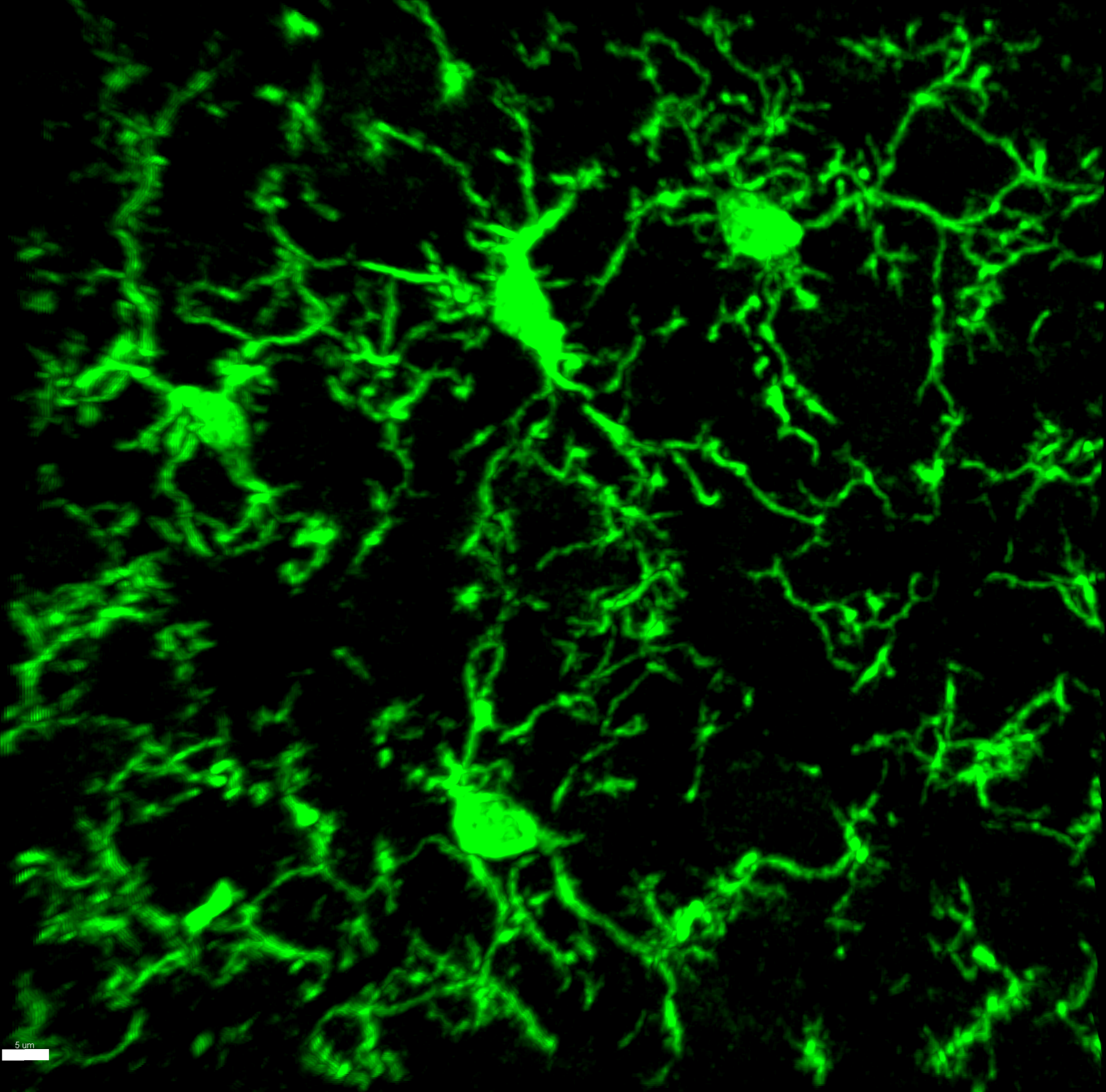

Supplement: Supplementary file 12 — Figure EV1 Source Data [file 44319_2026_721_MOESM12_ESM.zip › Figure EV1/EV1D/Cre-Arpc4floxedZoom-in.tif]

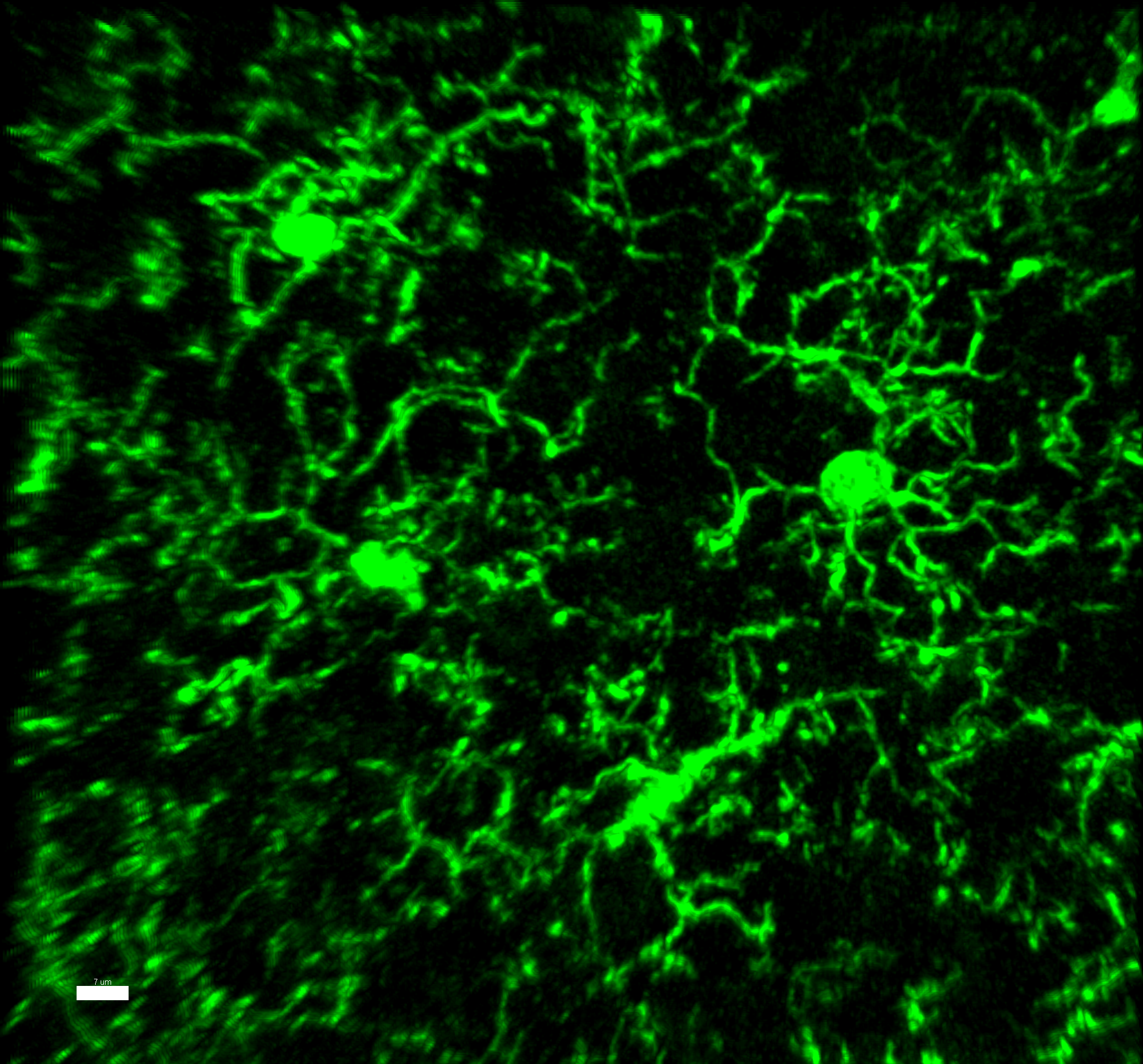

Supplement: Supplementary file 12 — Figure EV1 Source Data [file 44319_2026_721_MOESM12_ESM.zip › Figure EV1/EV1D/Cre+Arpc4+Zoom-in.tif]

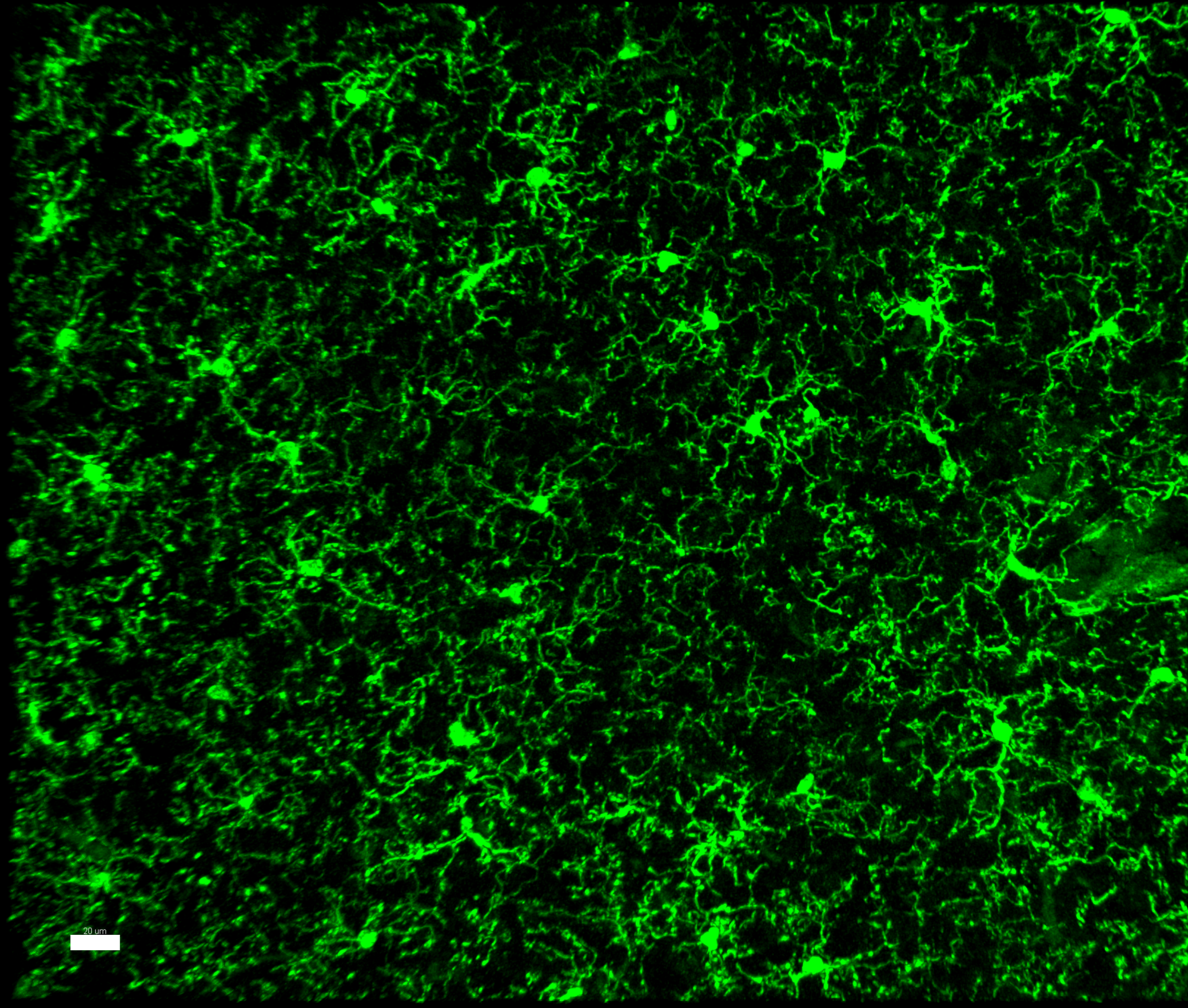

Supplement: Supplementary file 12 — Figure EV1 Source Data [file 44319_2026_721_MOESM12_ESM.zip › Figure EV1/EV1D/Cre+Arpc4+overview.004.tif]

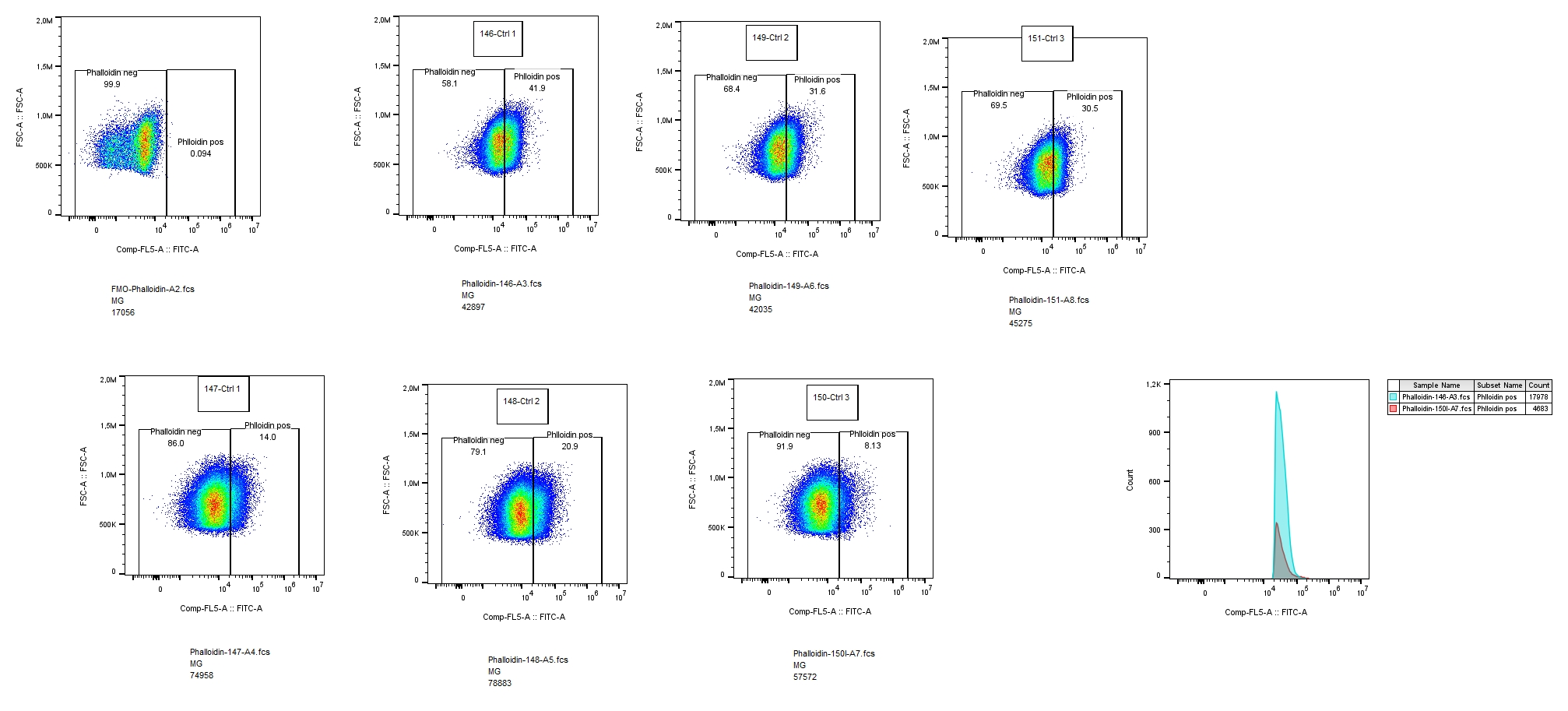

Supplement: Supplementary file 12 — Figure EV1 Source Data [file 44319_2026_721_MOESM12_ESM.zip › Figure EV1/EV1B/Phalloidin-FC analysis.jpg]

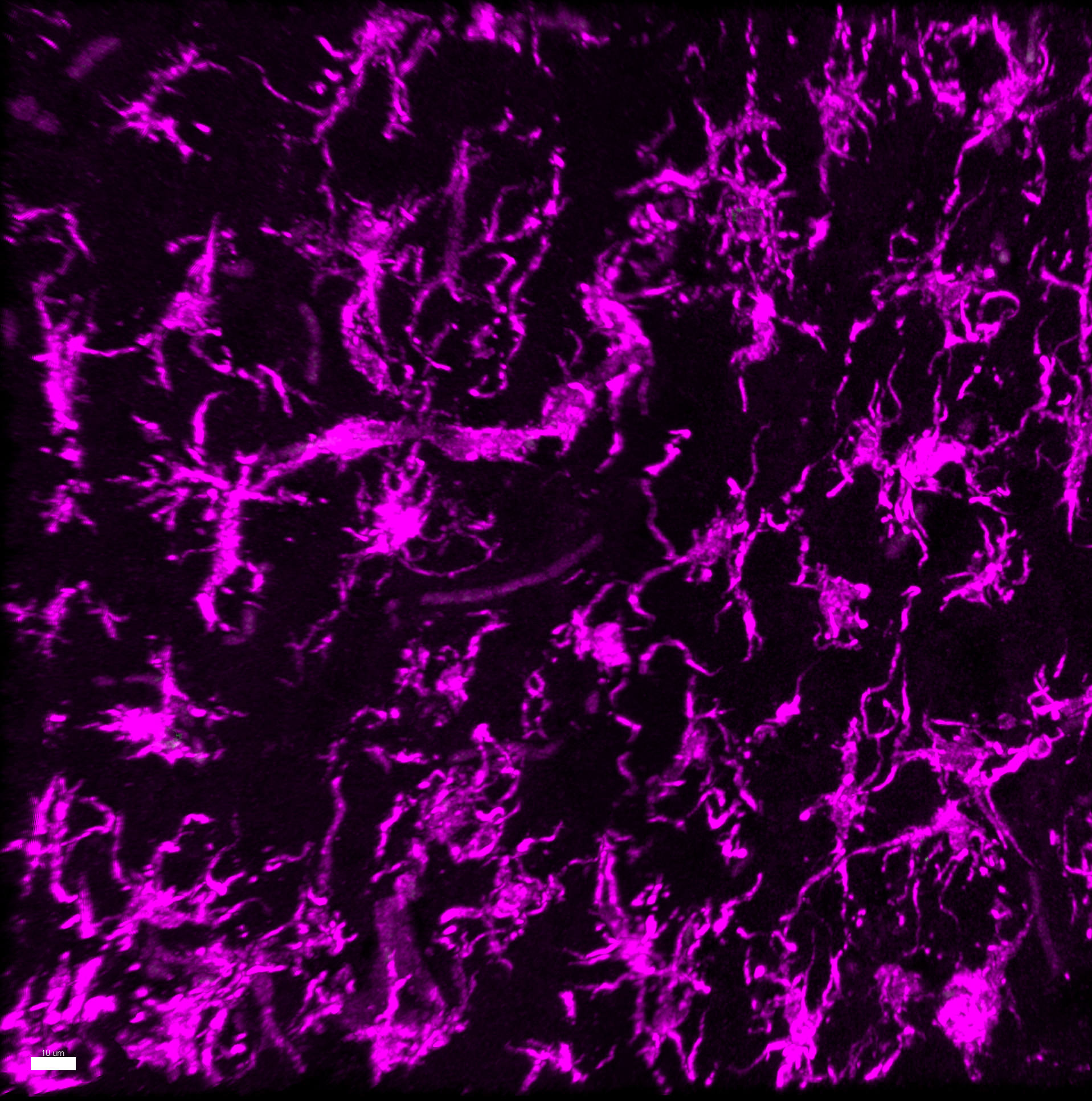

Supplement: Supplementary file 12 — Figure EV1 Source Data [file 44319_2026_721_MOESM12_ESM.zip › Figure EV1/EV1F/Cre+ Arpc4floxed-merge.tif]

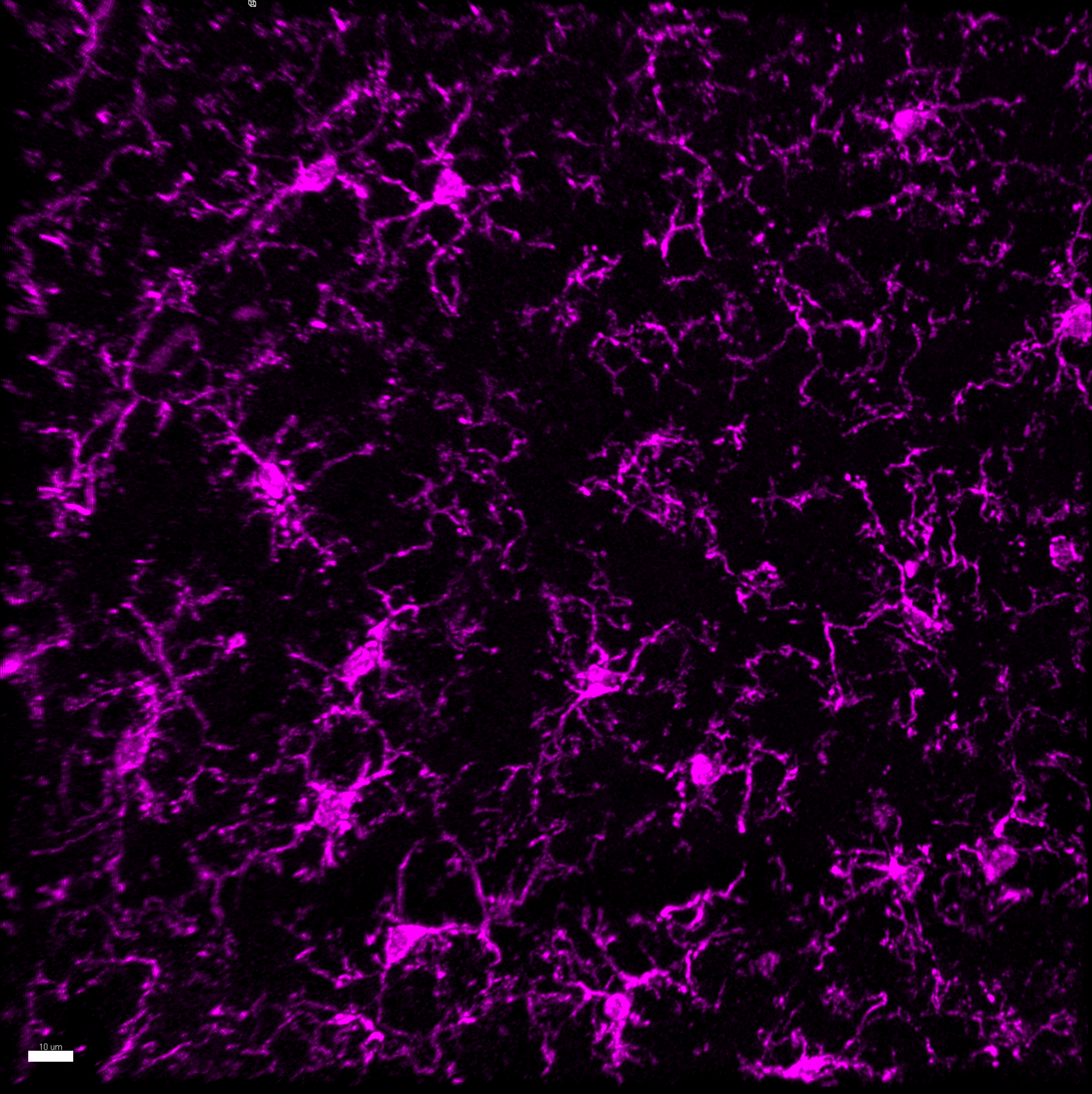

Supplement: Supplementary file 12 — Figure EV1 Source Data [file 44319_2026_721_MOESM12_ESM.zip › Figure EV1/EV1F/Cre-Arpc4floxed-IBA1.tif]

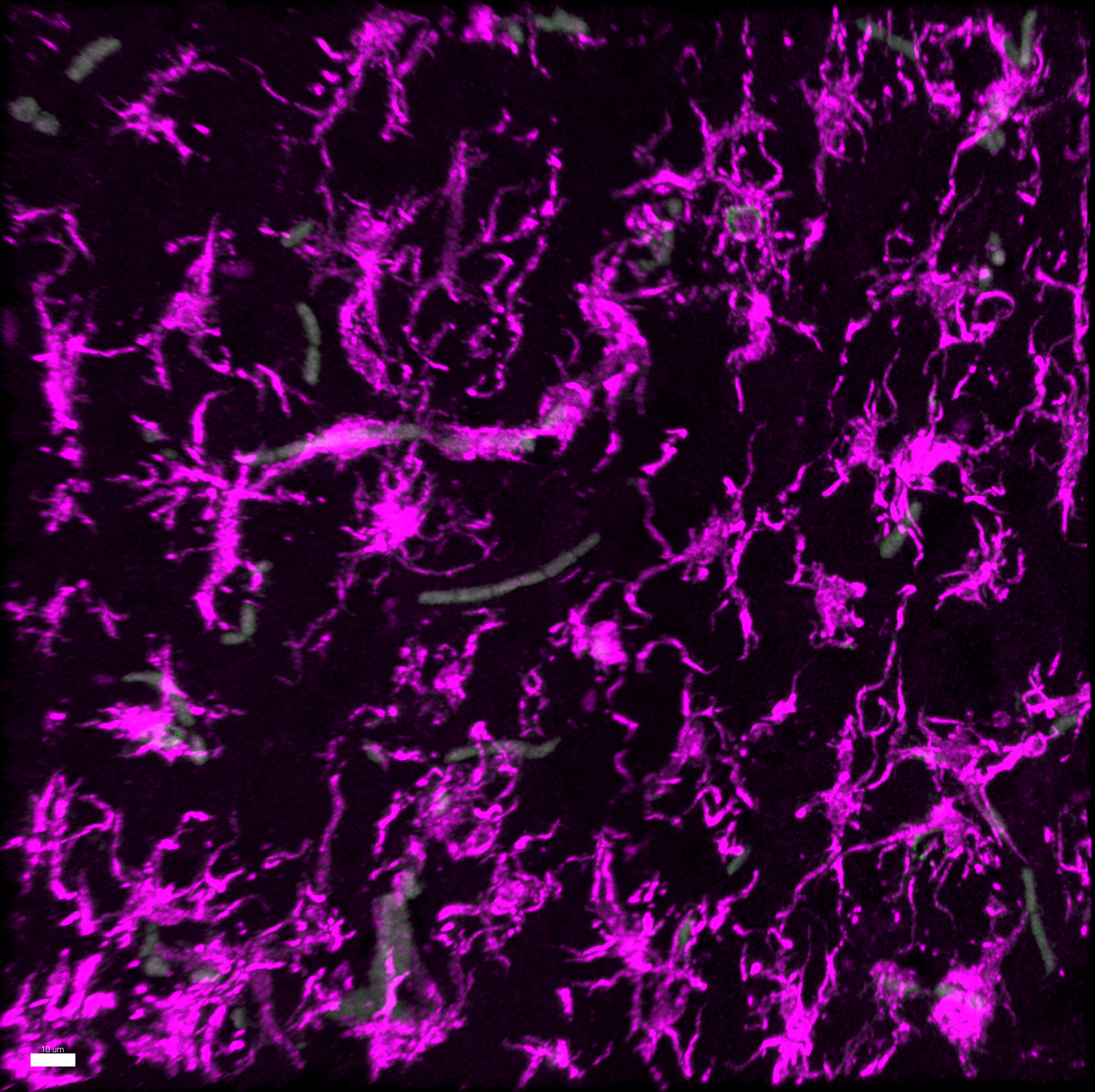

Supplement: Supplementary file 12 — Figure EV1 Source Data [file 44319_2026_721_MOESM12_ESM.zip › Figure EV1/EV1F/Cre+ Arpc4floxed-IBA1.tif]

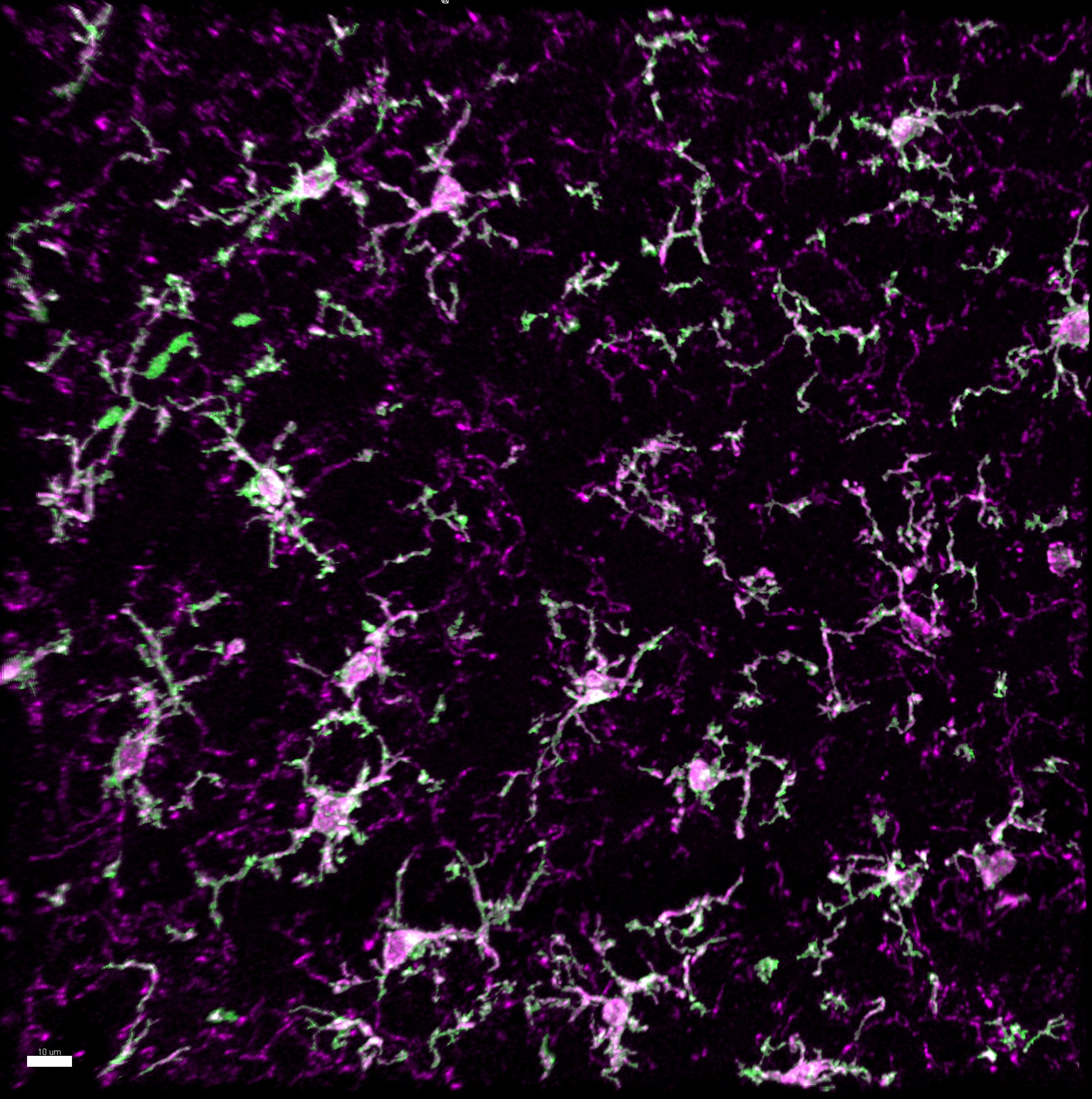

Supplement: Supplementary file 12 — Figure EV1 Source Data [file 44319_2026_721_MOESM12_ESM.zip › Figure EV1/EV1F/Cre- Arpc4floxed-merge.tif]

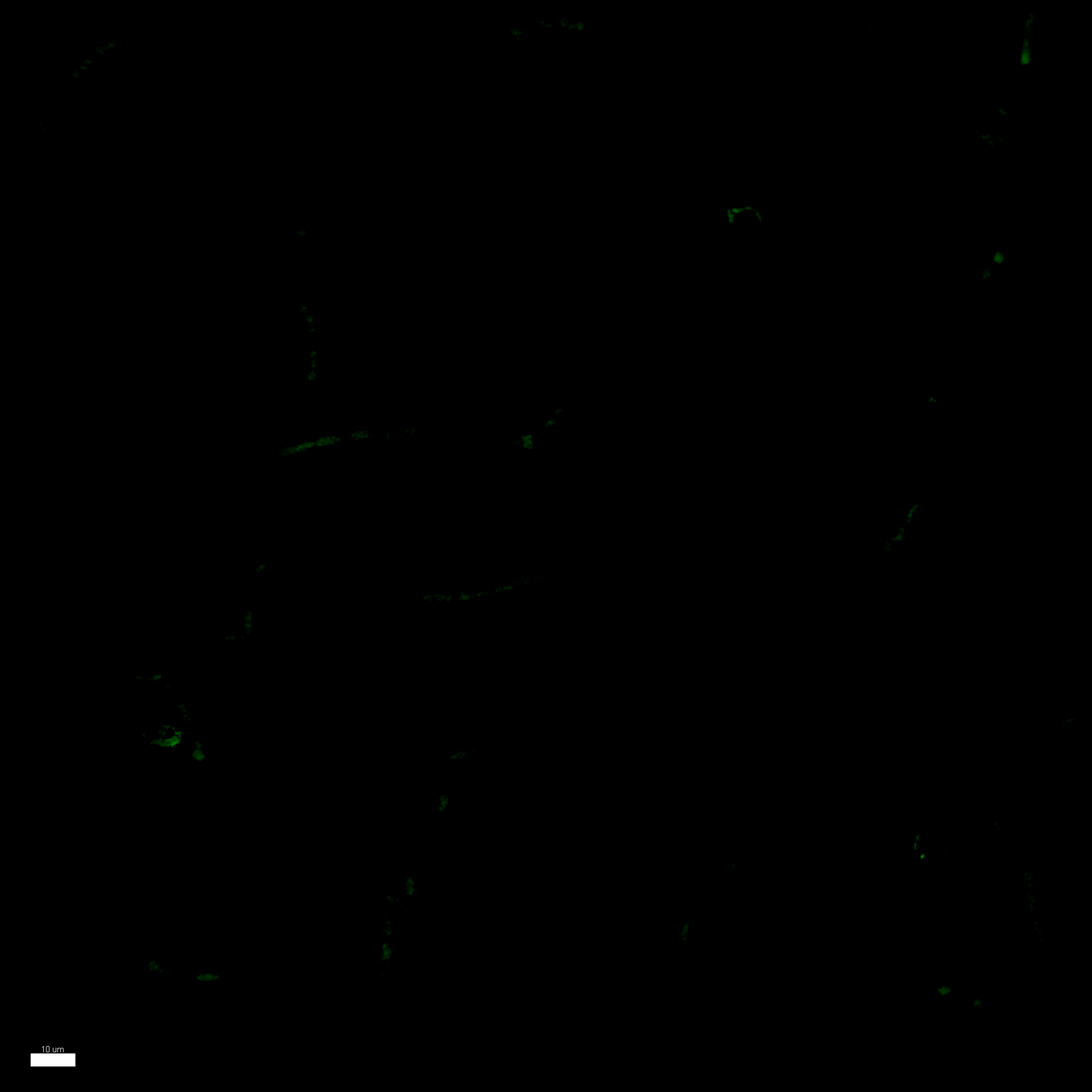

Supplement: Supplementary file 12 — Figure EV1 Source Data [file 44319_2026_721_MOESM12_ESM.zip › Figure EV1/EV1F/Cre+ Arpc4floxed-P2RY12.tif]

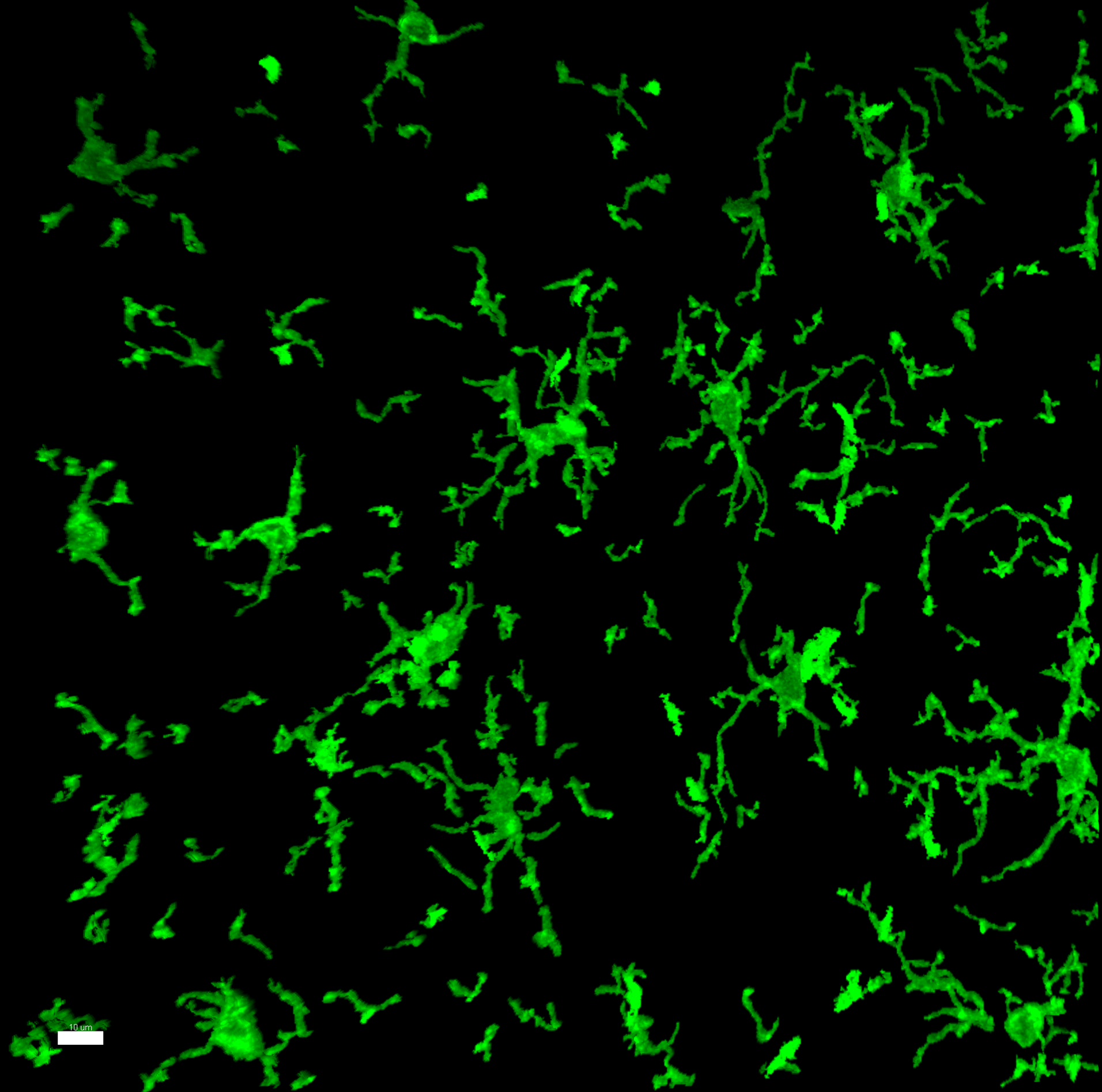

Supplement: Supplementary file 12 — Figure EV1 Source Data [file 44319_2026_721_MOESM12_ESM.zip › Figure EV1/EV1F/Cre+ Arpc4+P2RY12.tif]

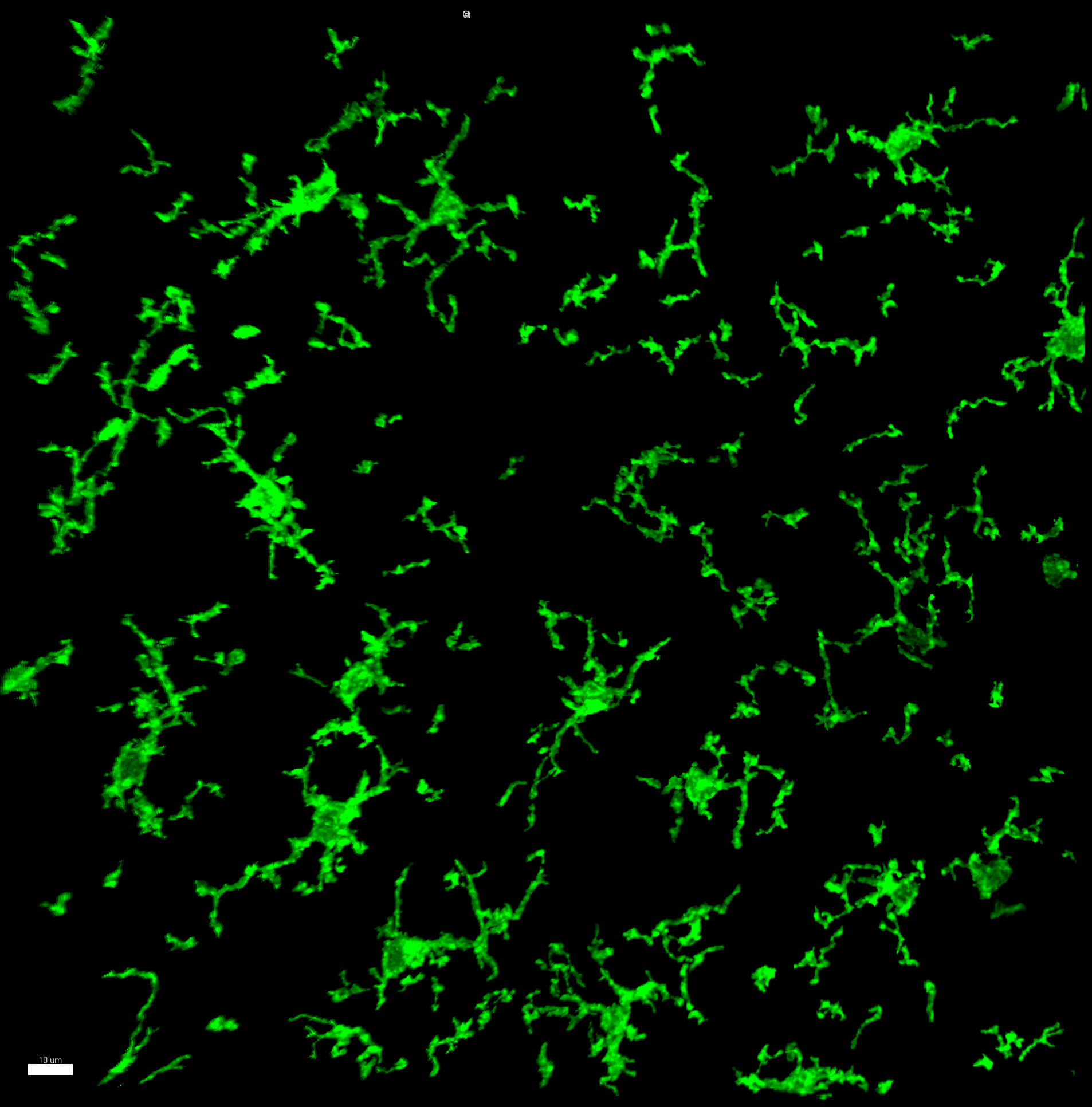

Supplement: Supplementary file 12 — Figure EV1 Source Data [file 44319_2026_721_MOESM12_ESM.zip › Figure EV1/EV1F/Cre-Arpc4floxed-P2RY12.tif]

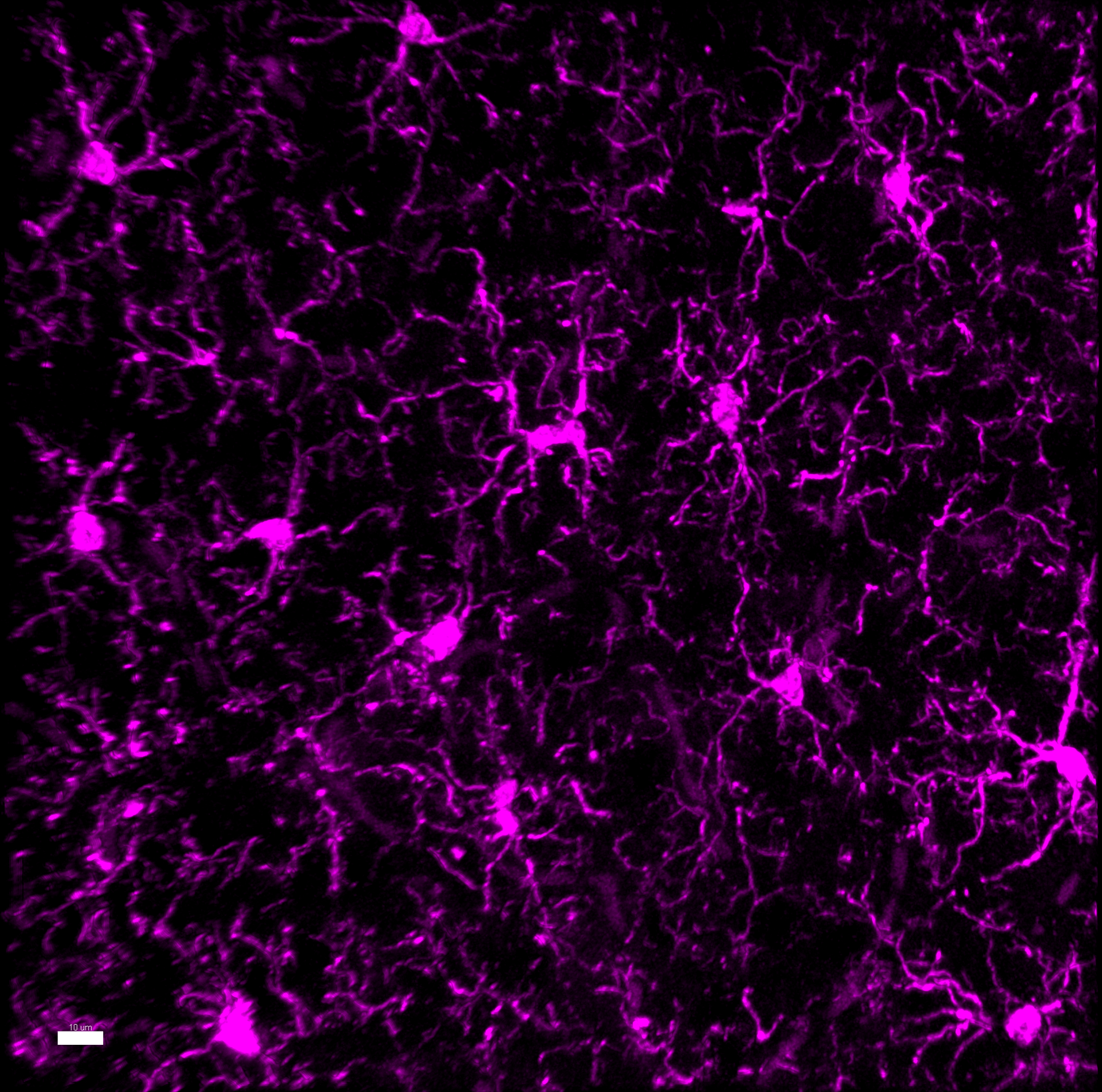

Supplement: Supplementary file 12 — Figure EV1 Source Data [file 44319_2026_721_MOESM12_ESM.zip › Figure EV1/EV1F/Cre+ Arpc4+IBA1.tif]

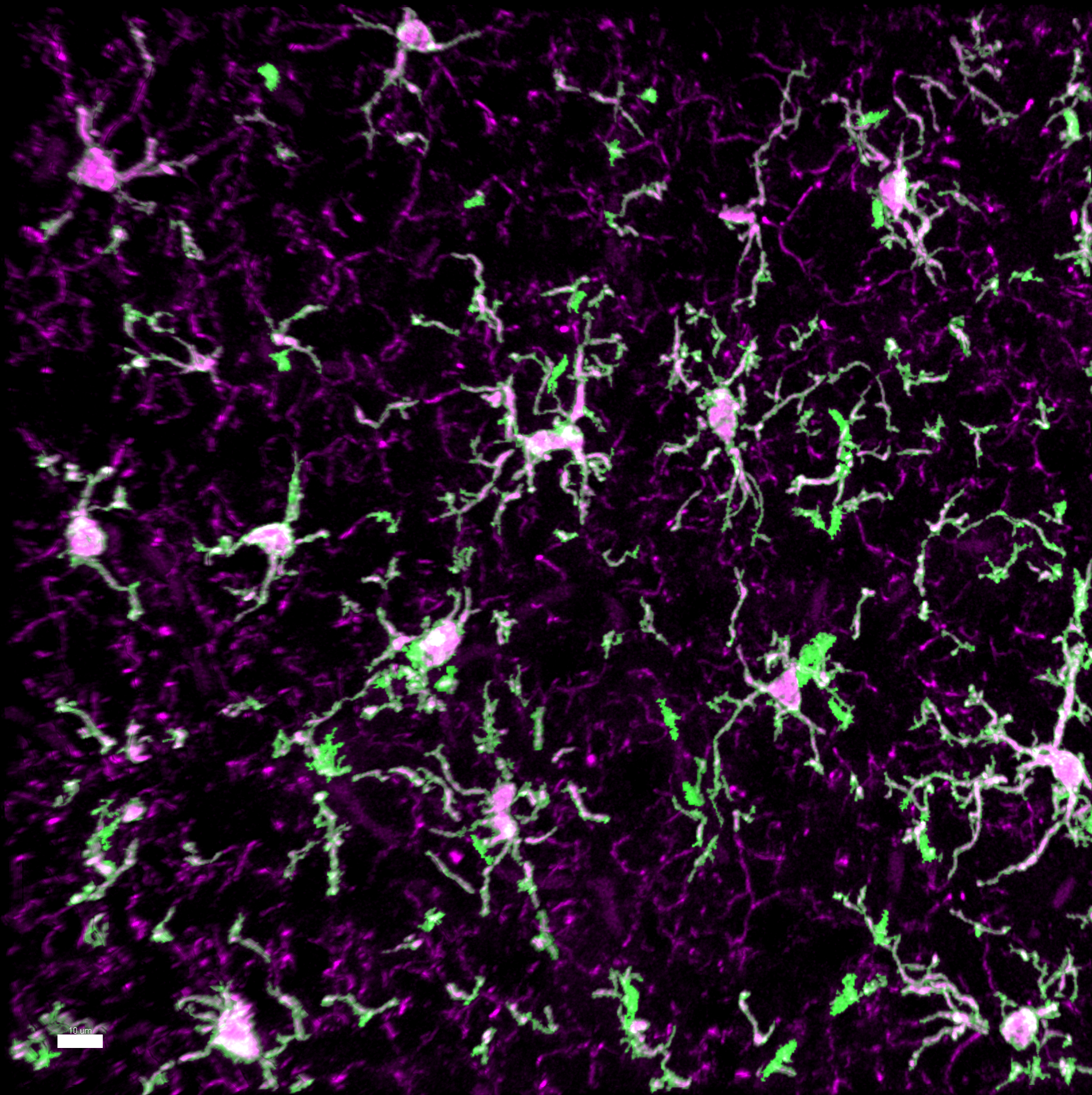

Supplement: Supplementary file 12 — Figure EV1 Source Data [file 44319_2026_721_MOESM12_ESM.zip › Figure EV1/EV1F/Cre+ Arpc4+merge.tif]

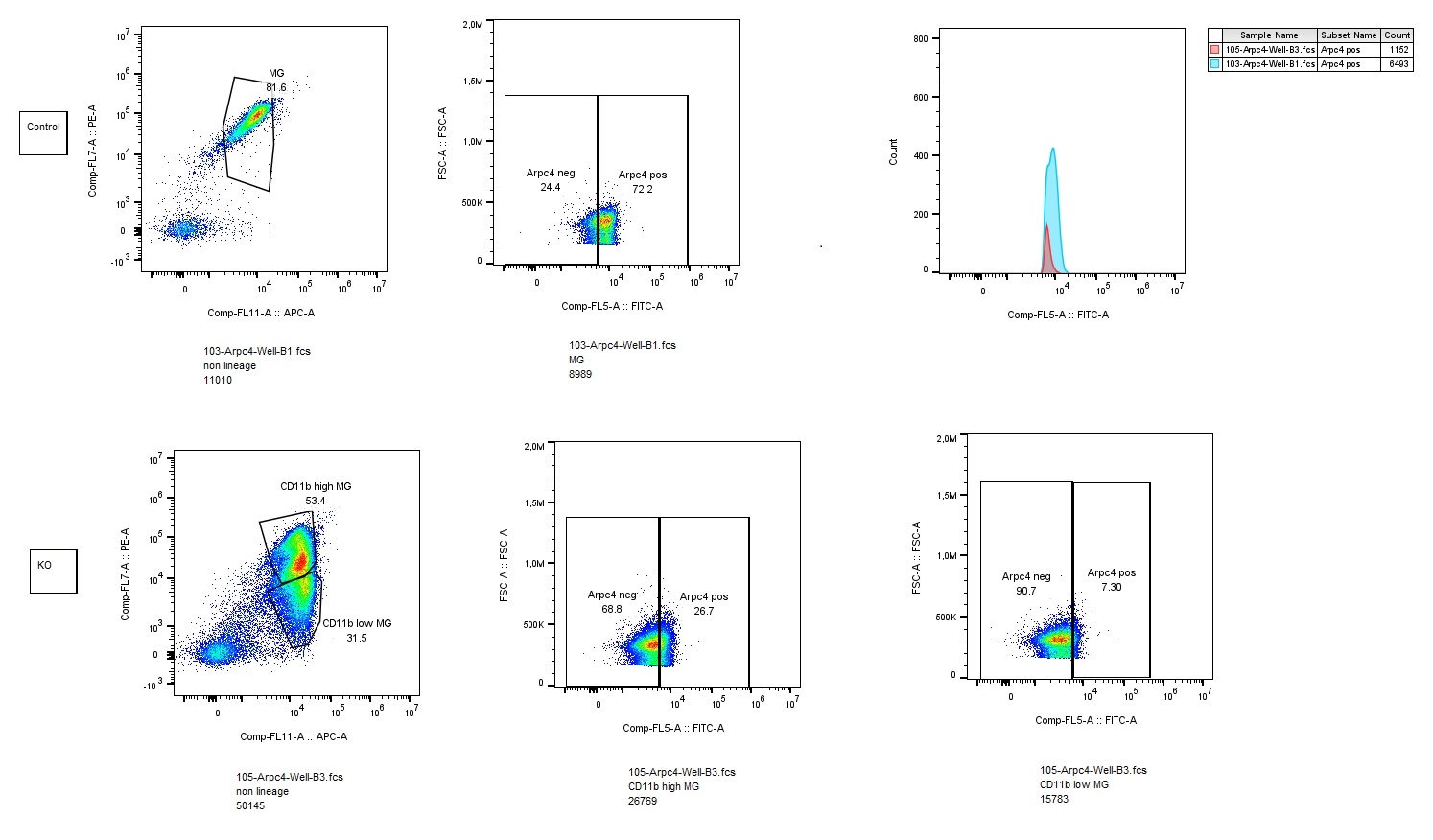

Supplement: Supplementary file 12 — Figure EV1 Source Data [file 44319_2026_721_MOESM12_ESM.zip › Figure EV1/EV1A/ARPC4/ARPC4-FC analysis.jpg]

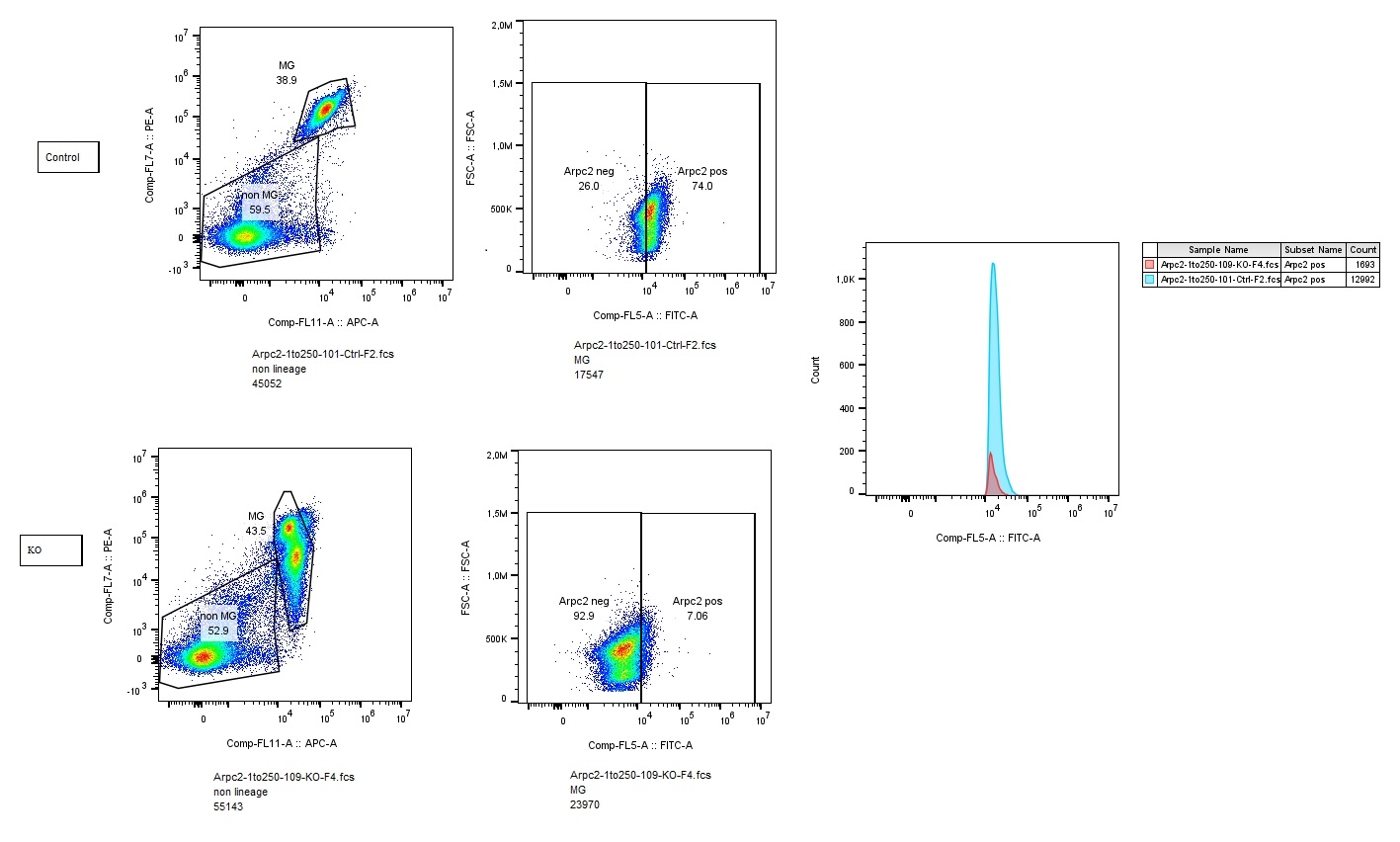

Supplement: Supplementary file 12 — Figure EV1 Source Data [file 44319_2026_721_MOESM12_ESM.zip › Figure EV1/EV1A/ARPC2/ARPC2-FC analysis.jpg]

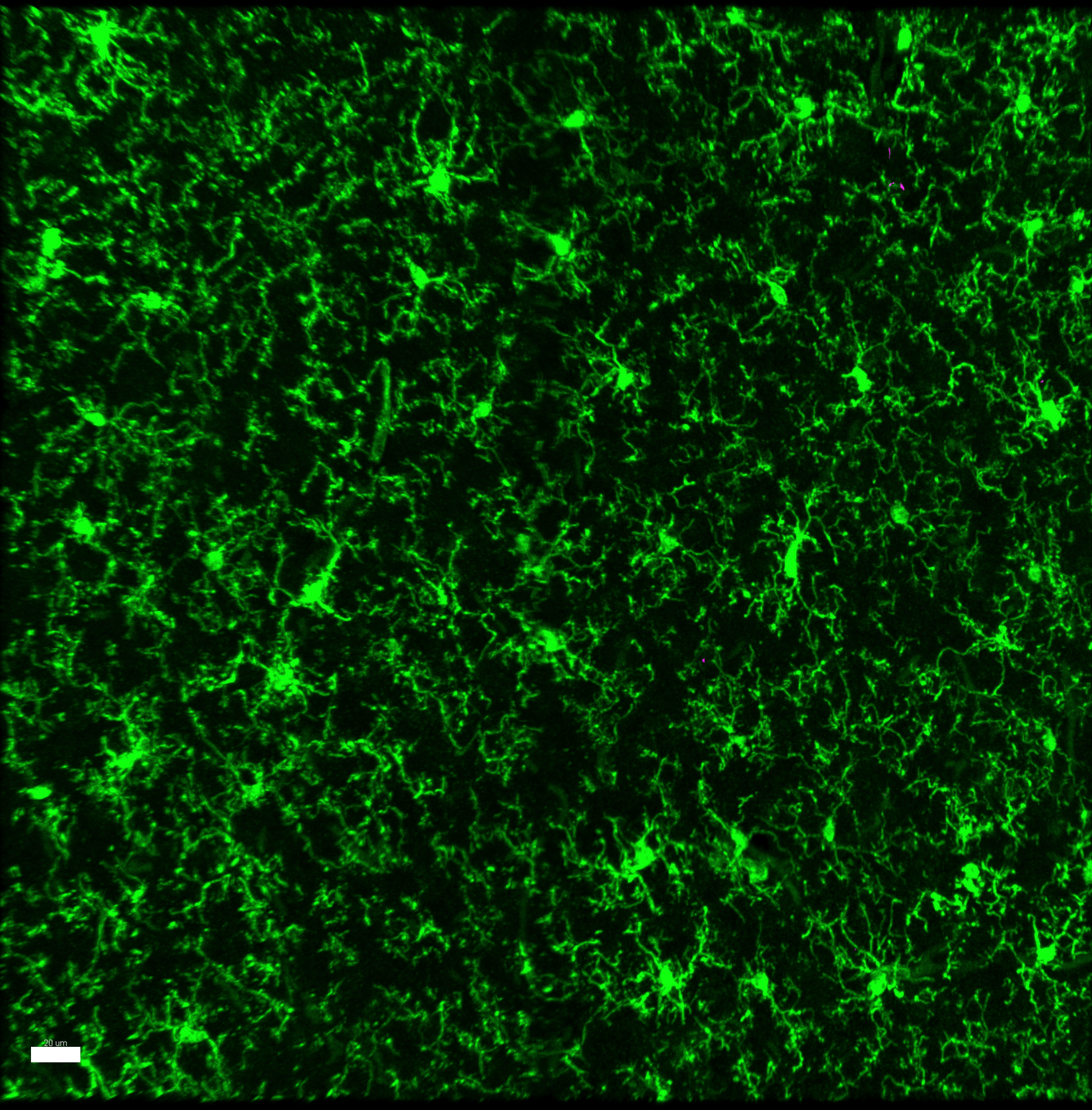

Supplement: Supplementary file 13 — Figure EV2 Source Data [file 44319_2026_721_MOESM13_ESM.zip › Figure EV2/EV2B/AXL/Cre+Arpc4+merge-cortex.tif]

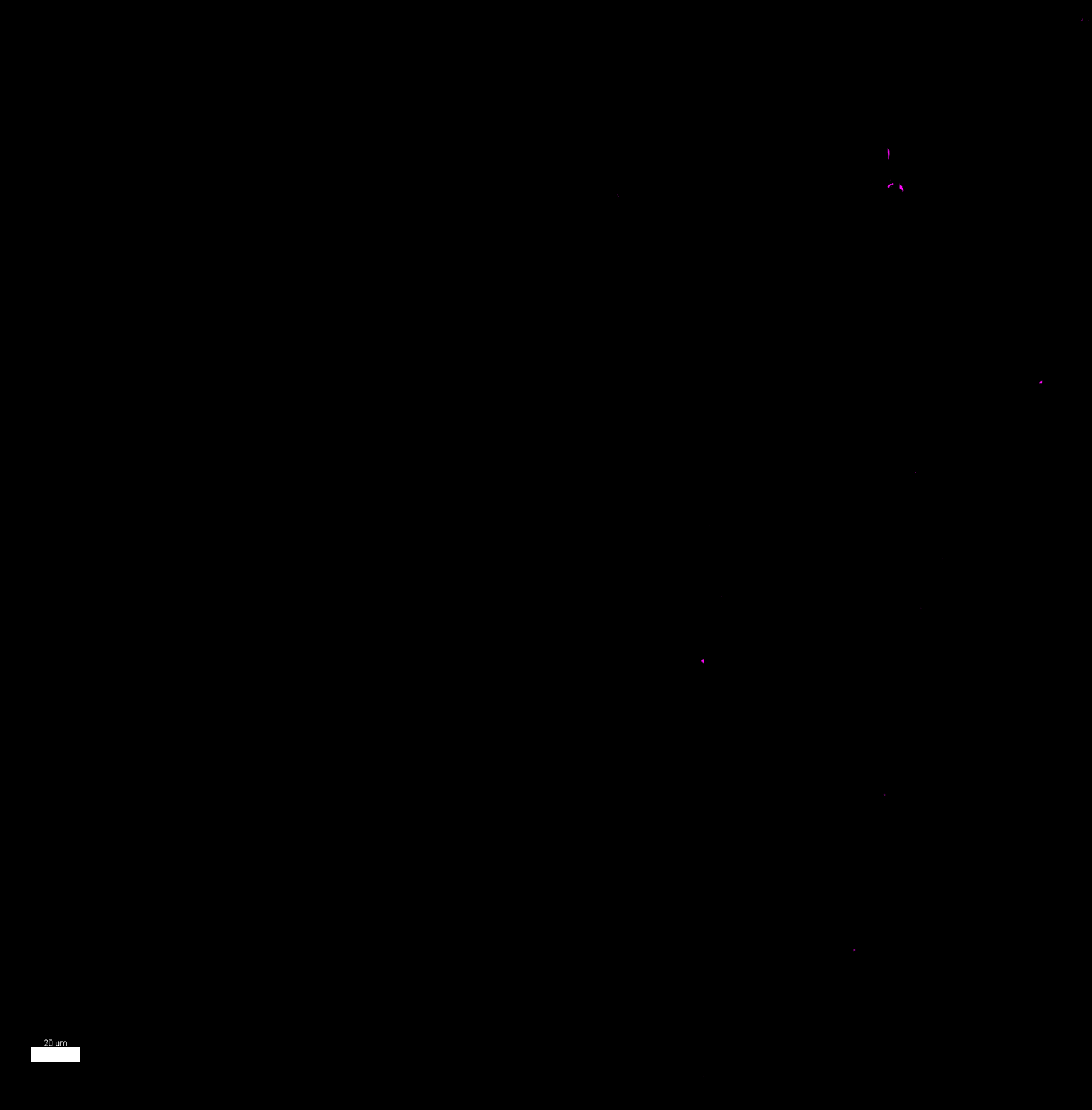

Supplement: Supplementary file 13 — Figure EV2 Source Data [file 44319_2026_721_MOESM13_ESM.zip › Figure EV2/EV2B/AXL/Cre+Arpc4+AXL-cortex.tif]

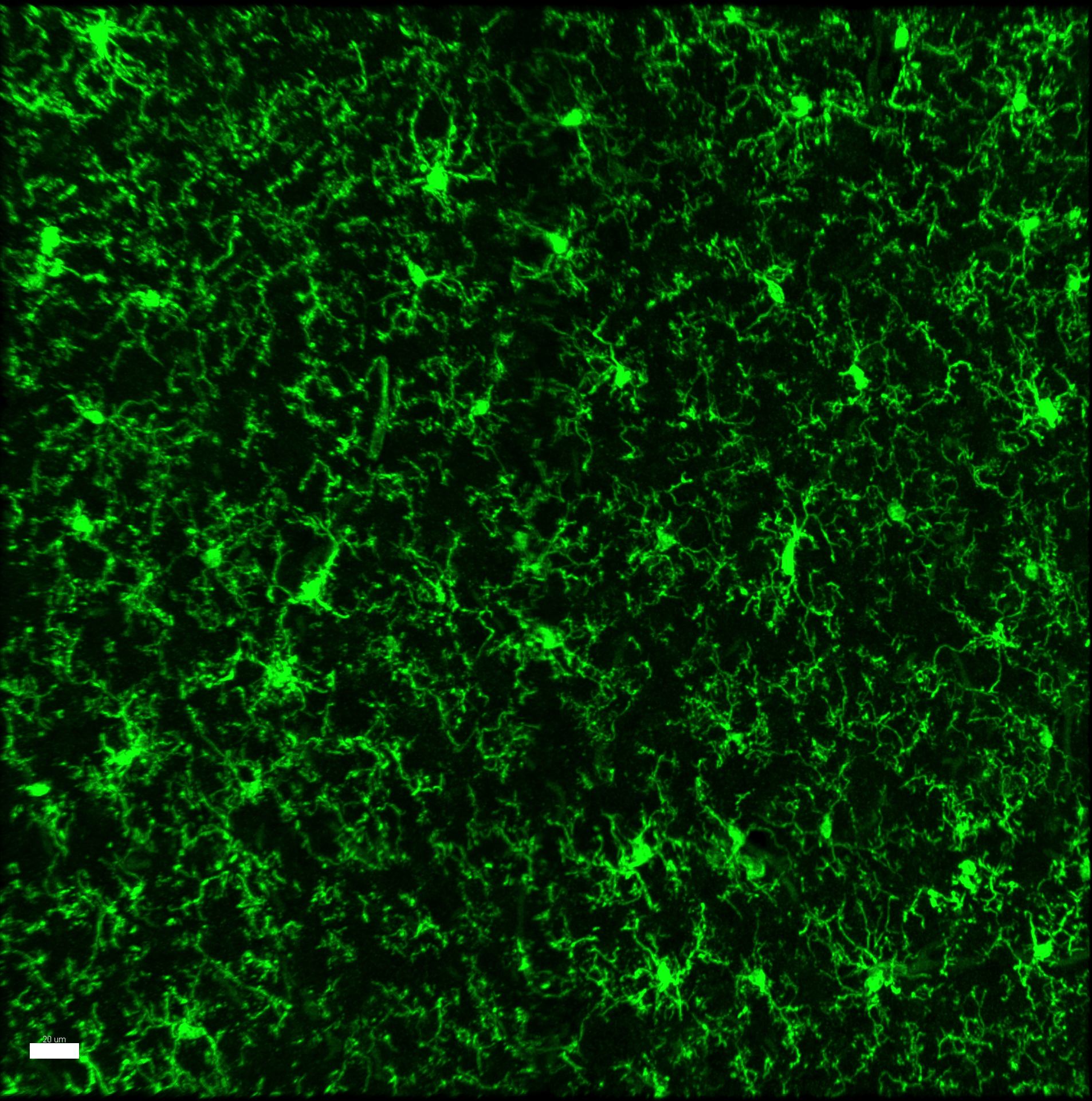

Supplement: Supplementary file 13 — Figure EV2 Source Data [file 44319_2026_721_MOESM13_ESM.zip › Figure EV2/EV2B/AXL/Cre+Arpc4+IBA1-cortex.tif]

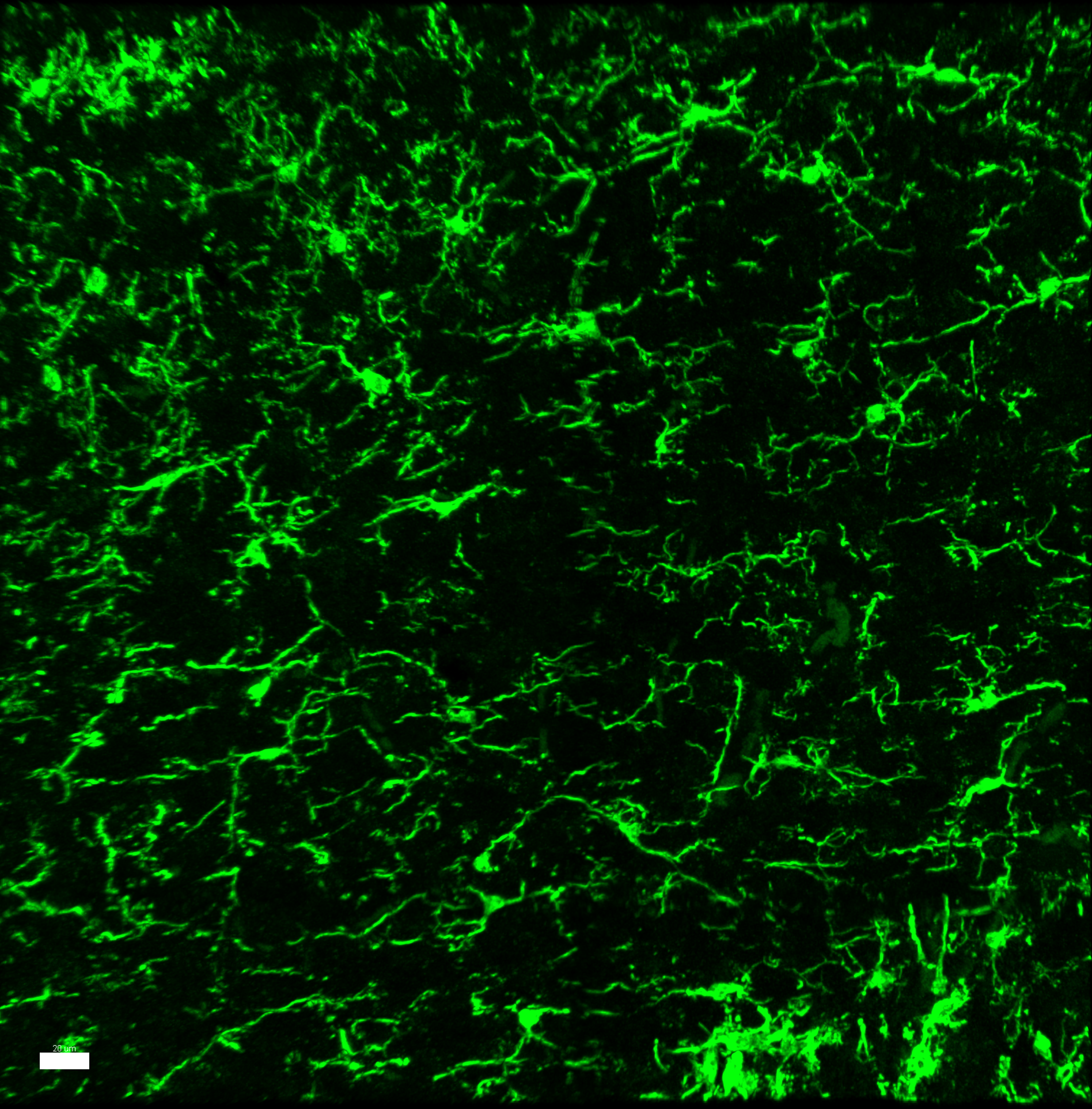

Supplement: Supplementary file 13 — Figure EV2 Source Data [file 44319_2026_721_MOESM13_ESM.zip › Figure EV2/EV2B/AXL/Cre+Arpc4+IBA1-CC.tif]

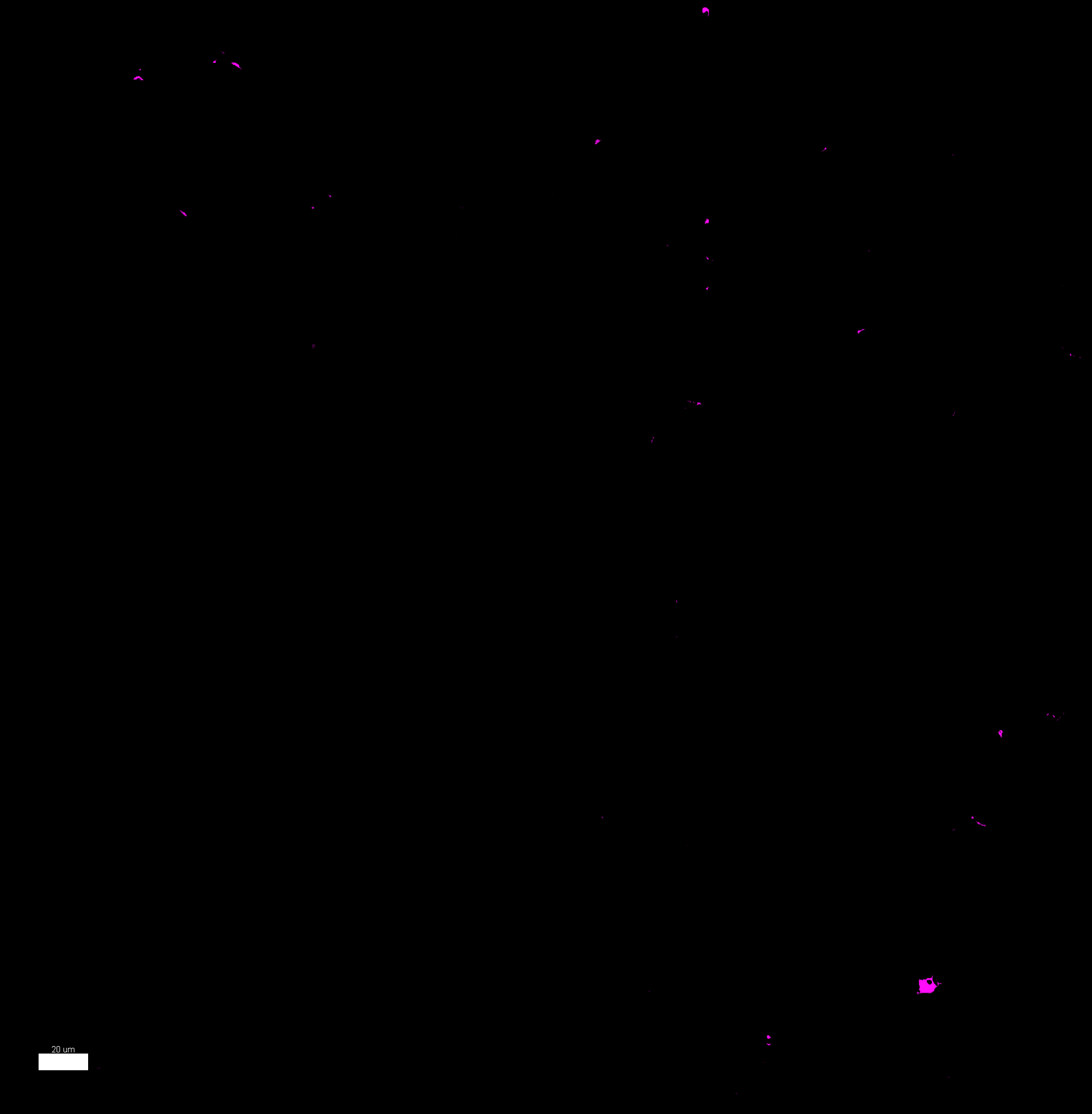

Supplement: Supplementary file 13 — Figure EV2 Source Data [file 44319_2026_721_MOESM13_ESM.zip › Figure EV2/EV2B/AXL/Cre+Arpc4+AXLCC.tif]

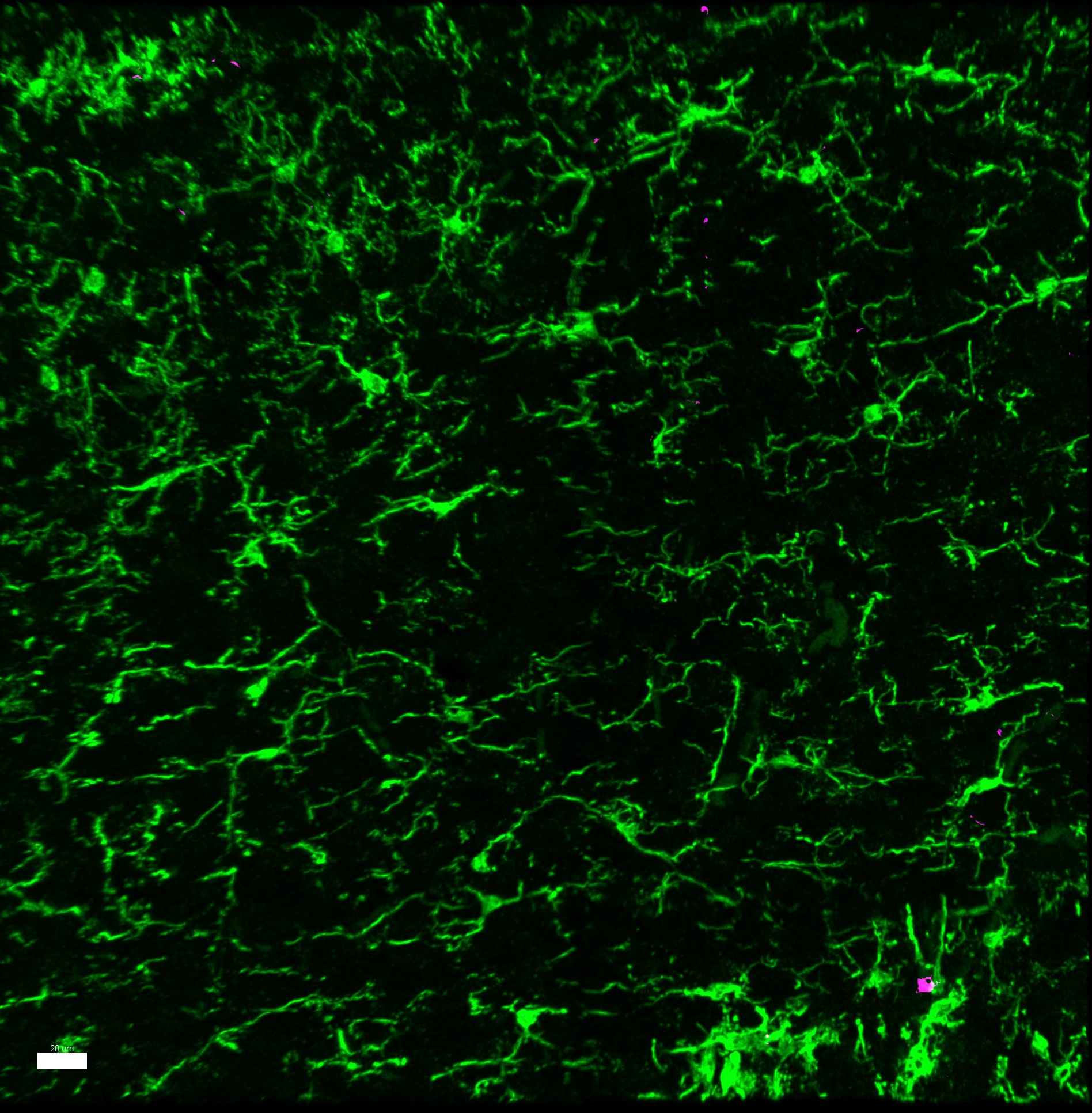

Supplement: Supplementary file 13 — Figure EV2 Source Data [file 44319_2026_721_MOESM13_ESM.zip › Figure EV2/EV2B/AXL/Cre+Arpc4+merge-CC.tif]

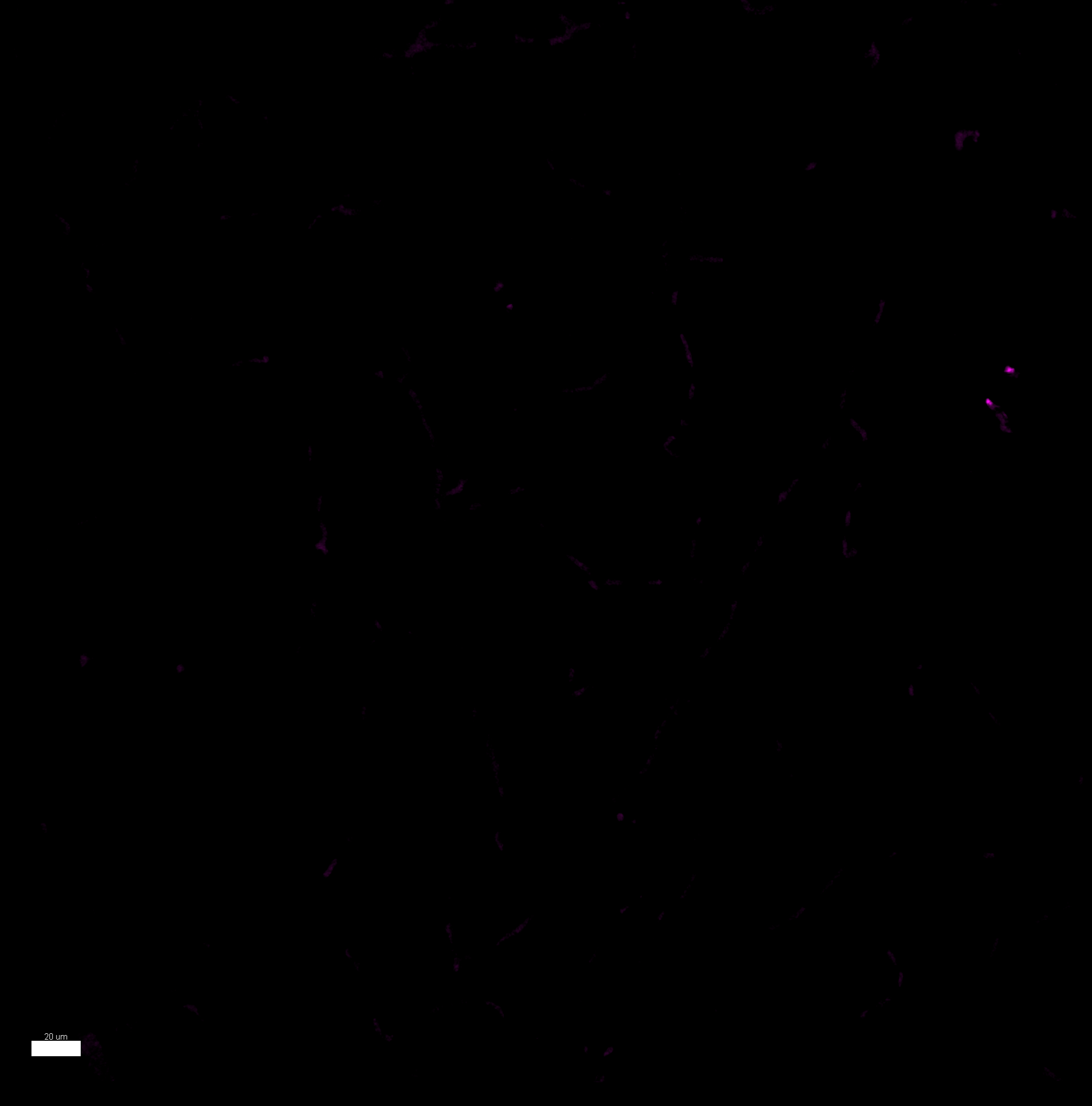

Supplement: Supplementary file 13 — Figure EV2 Source Data [file 44319_2026_721_MOESM13_ESM.zip › Figure EV2/EV2B/Galectin3/Cre+Arpc4+Galectin3-cortex.tif]

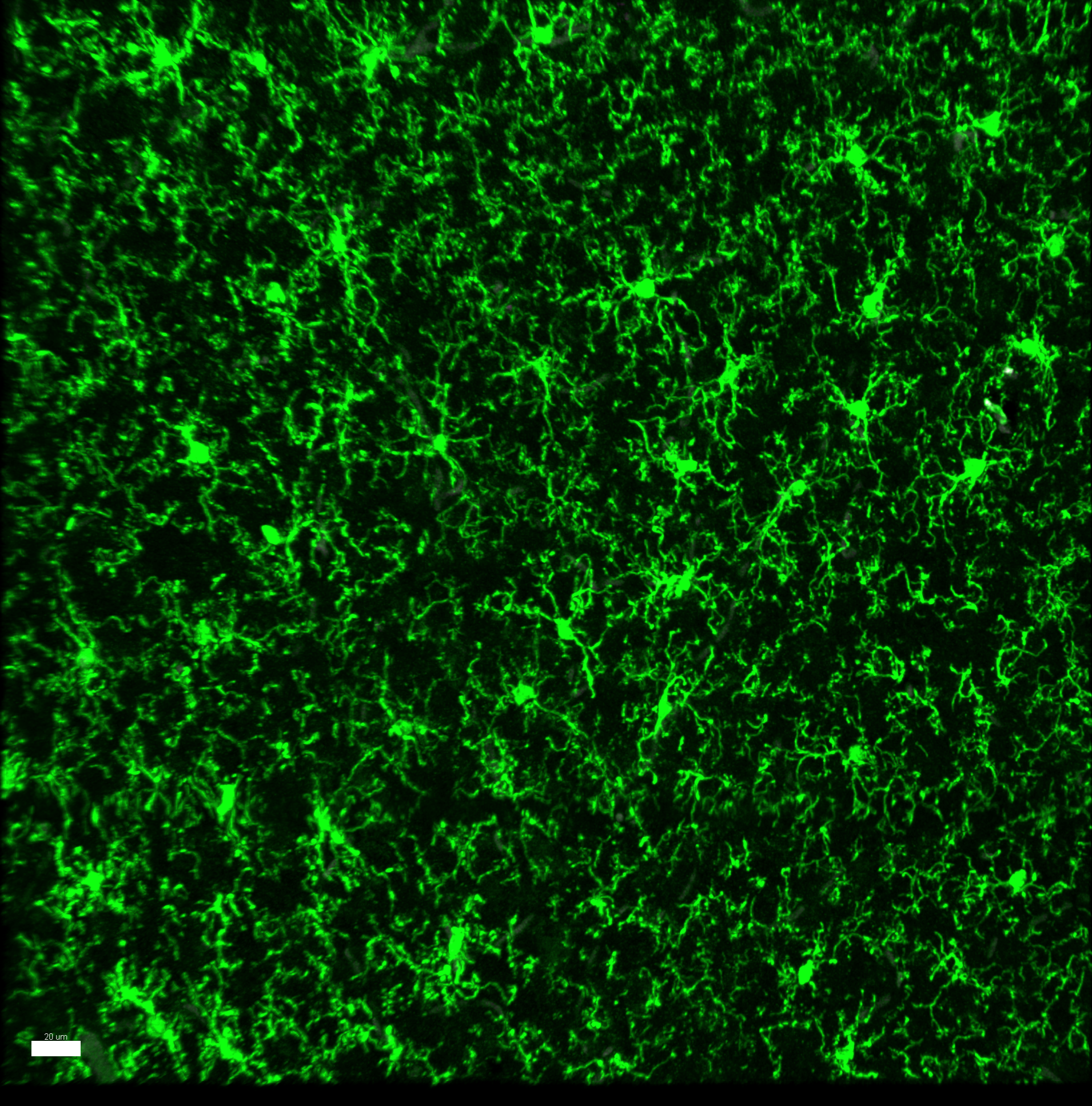

Supplement: Supplementary file 13 — Figure EV2 Source Data [file 44319_2026_721_MOESM13_ESM.zip › Figure EV2/EV2B/Galectin3/Cre+Arpc4+merge-cortex.tif]

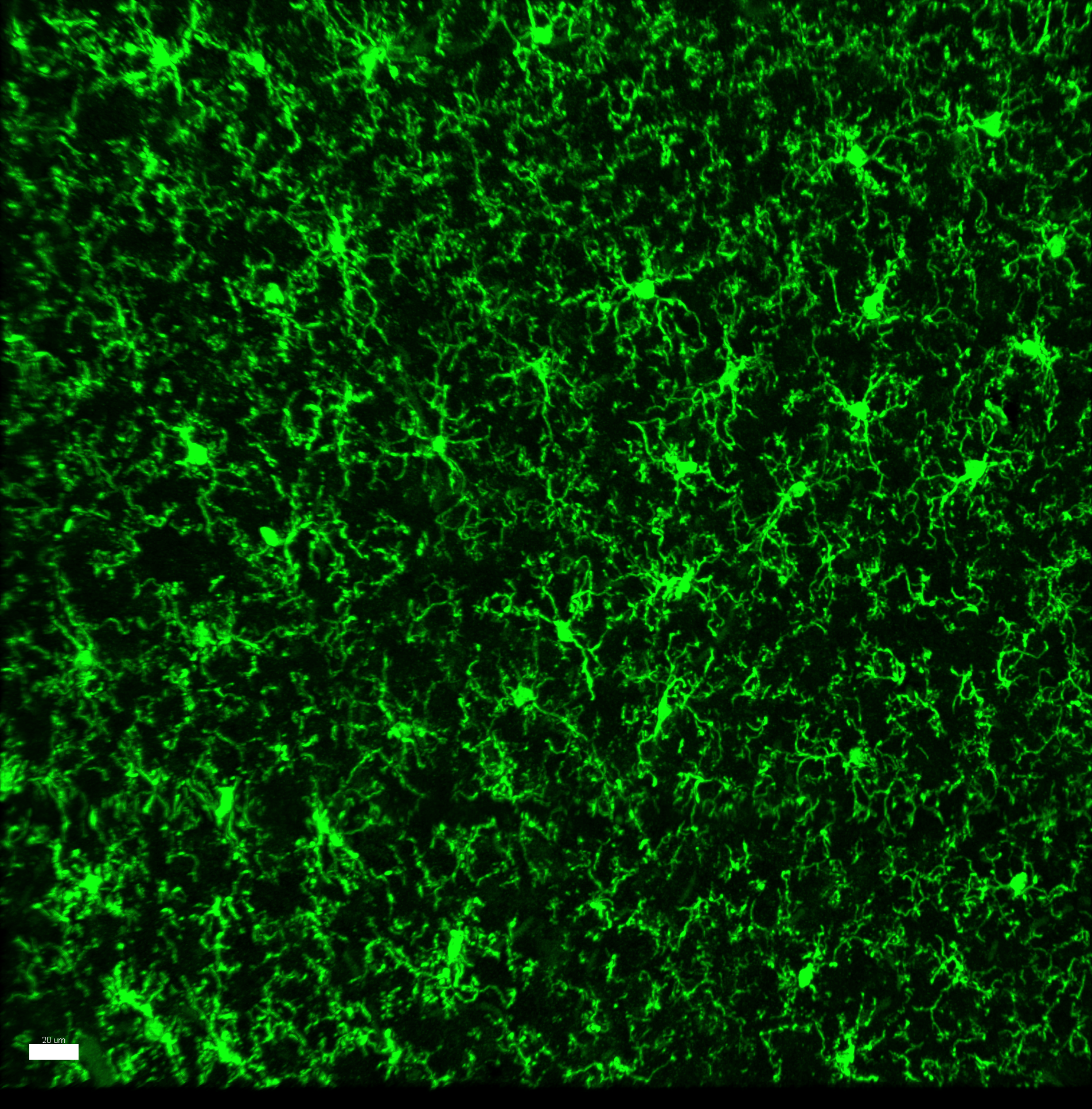

Supplement: Supplementary file 13 — Figure EV2 Source Data [file 44319_2026_721_MOESM13_ESM.zip › Figure EV2/EV2B/Galectin3/Cre+Arpc4+IBA1-cortex.tif]

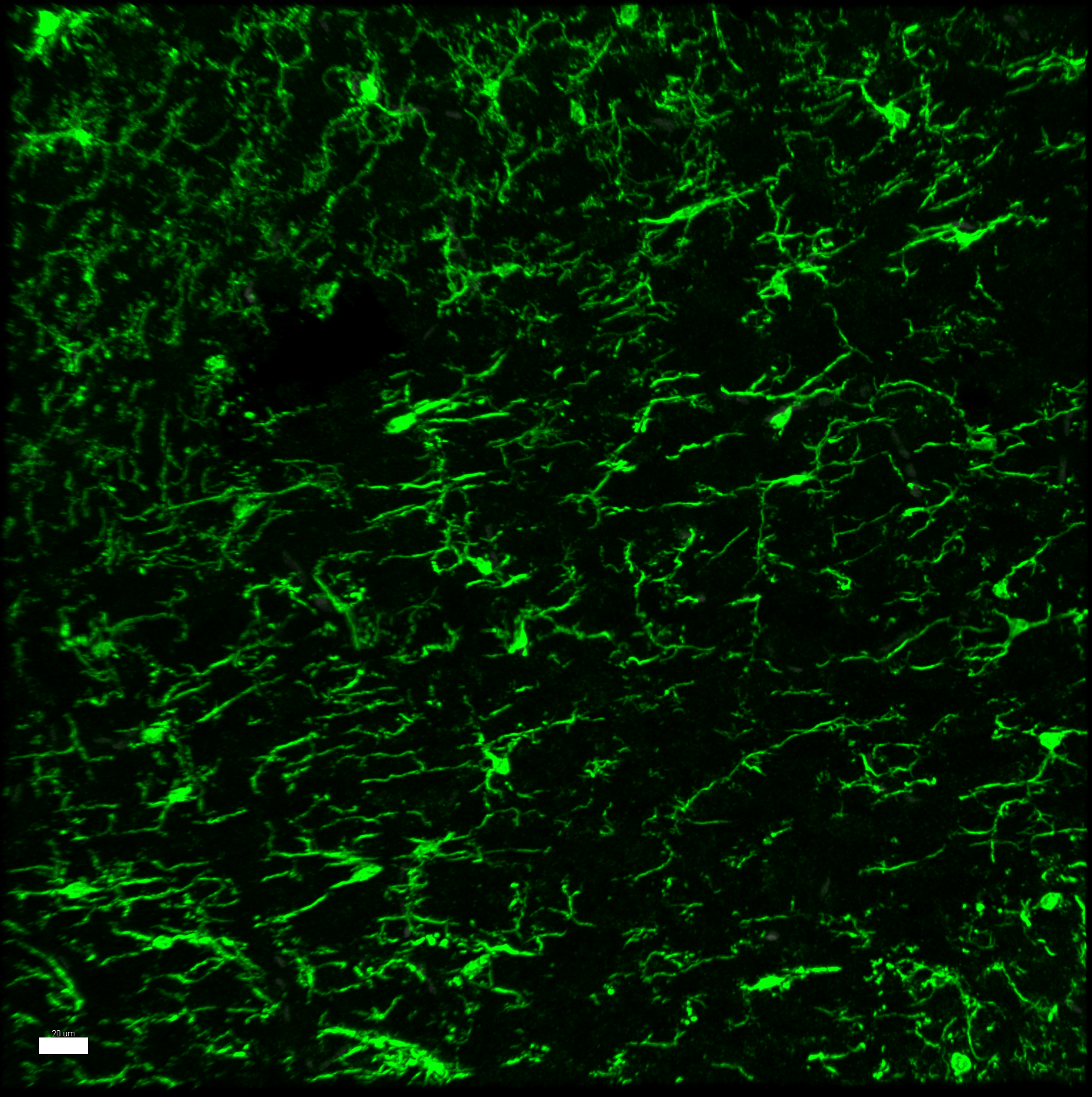

Supplement: Supplementary file 13 — Figure EV2 Source Data [file 44319_2026_721_MOESM13_ESM.zip › Figure EV2/EV2B/Galectin3/Cre+Arpc4+IBA1-CC.tif]

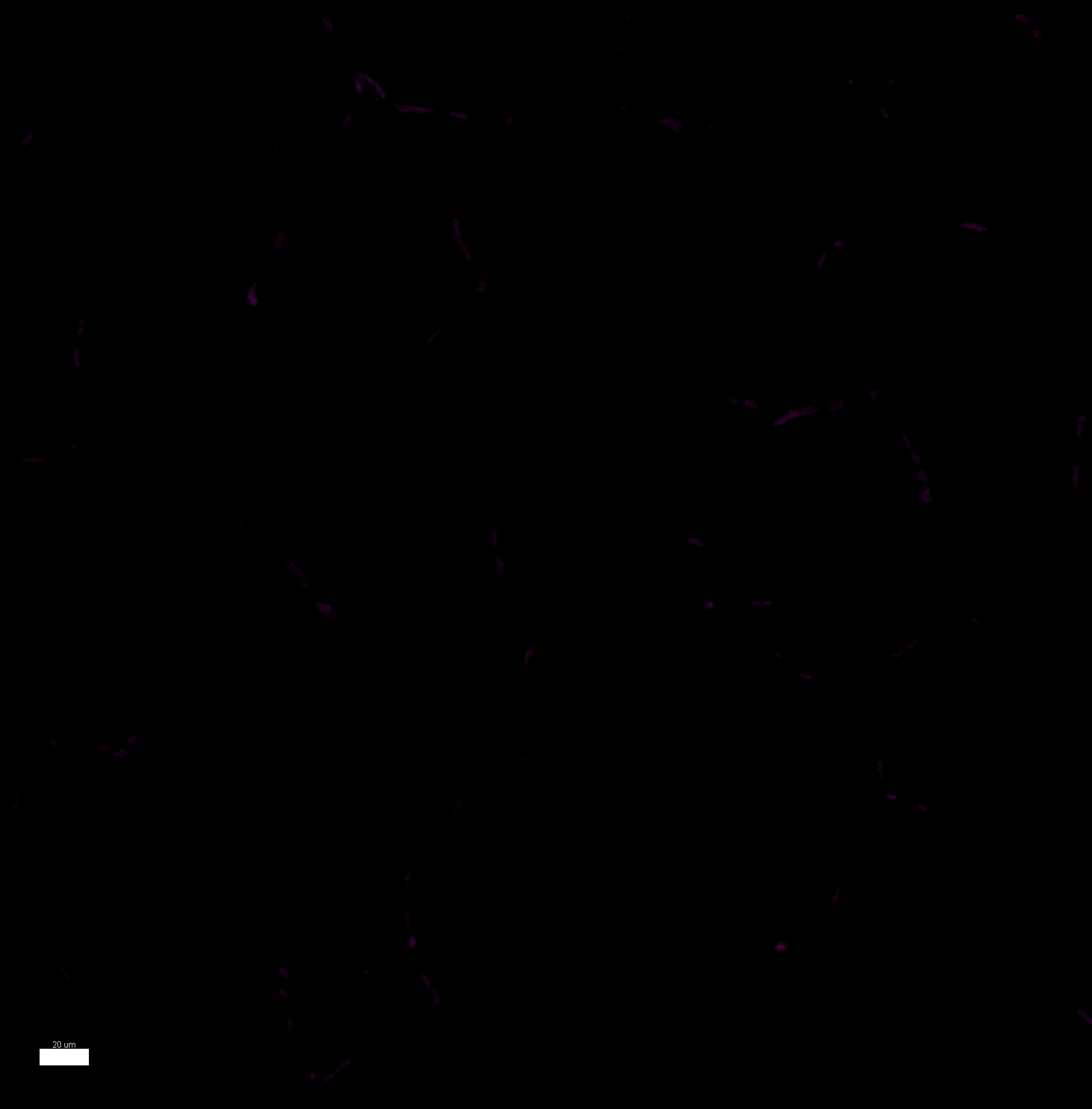

Supplement: Supplementary file 13 — Figure EV2 Source Data [file 44319_2026_721_MOESM13_ESM.zip › Figure EV2/EV2B/Galectin3/Cre+Arpc4+Galectin3-CC.tif]

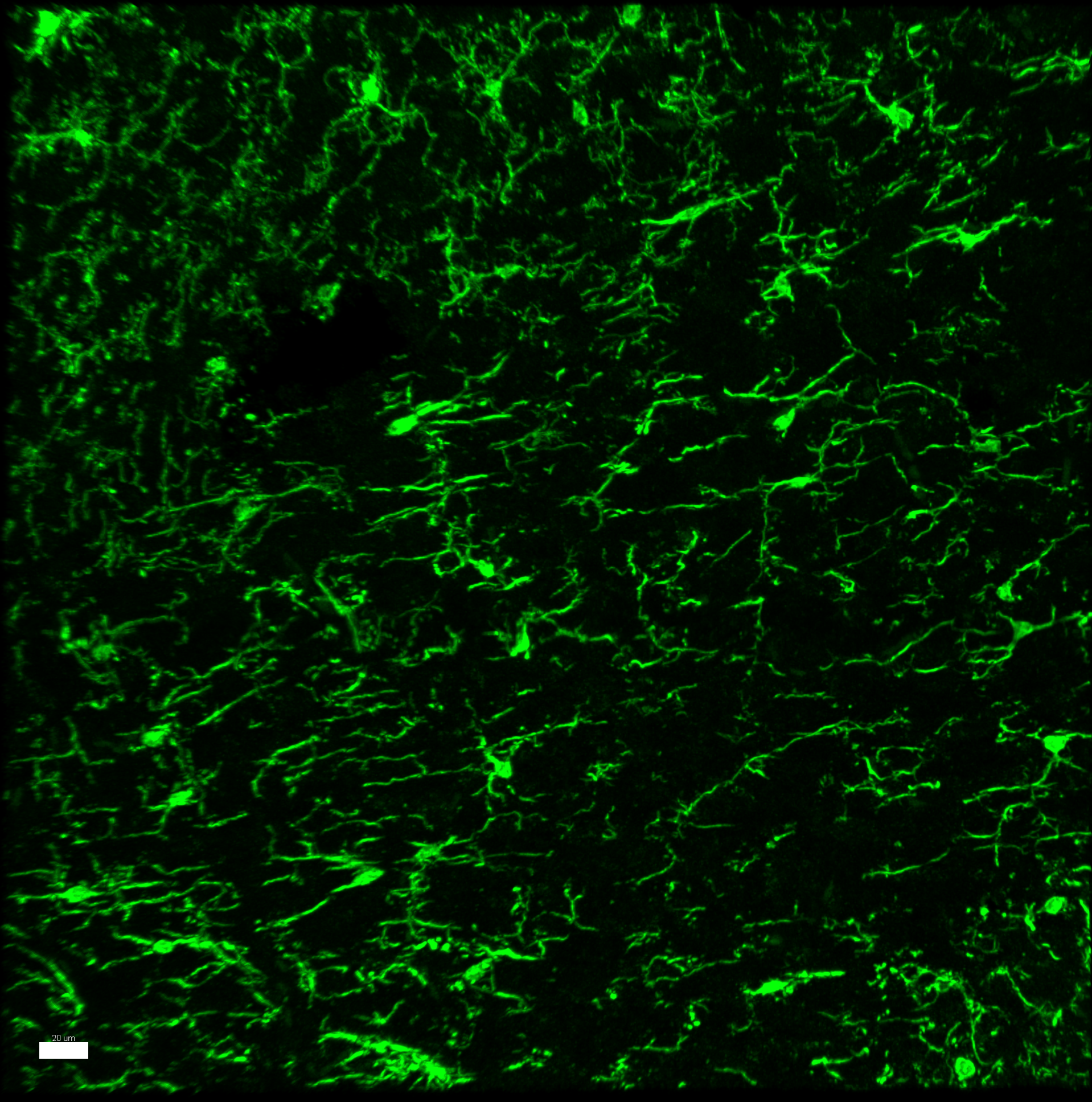

Supplement: Supplementary file 13 — Figure EV2 Source Data [file 44319_2026_721_MOESM13_ESM.zip › Figure EV2/EV2B/Galectin3/Cre+Arpc4+merge-CC.tif]

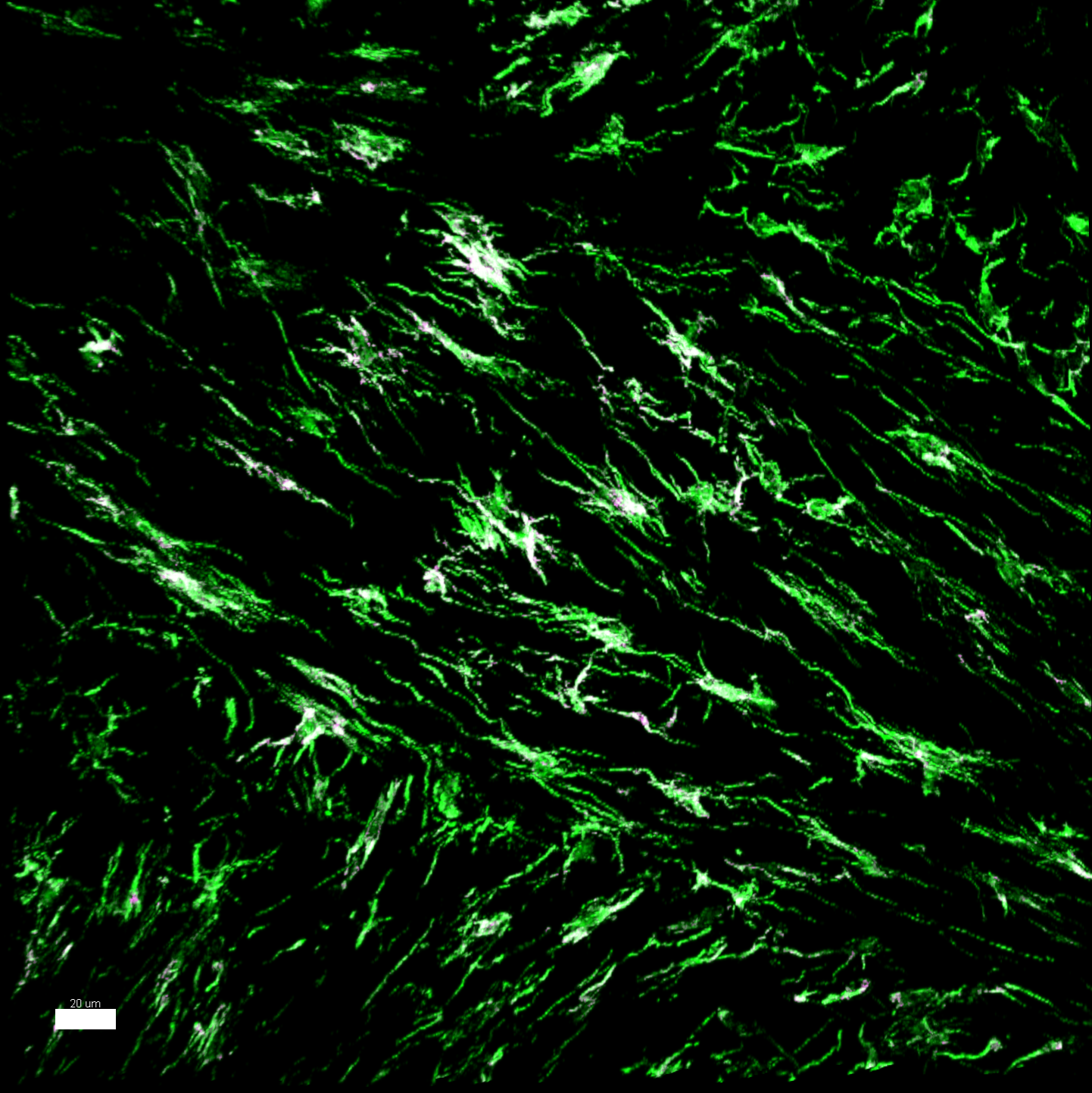

Supplement: Supplementary file 13 — Figure EV2 Source Data [file 44319_2026_721_MOESM13_ESM.zip › Figure EV2/EV2C/AXL/Cre+Arpc4floxed-merge-CC.tif]

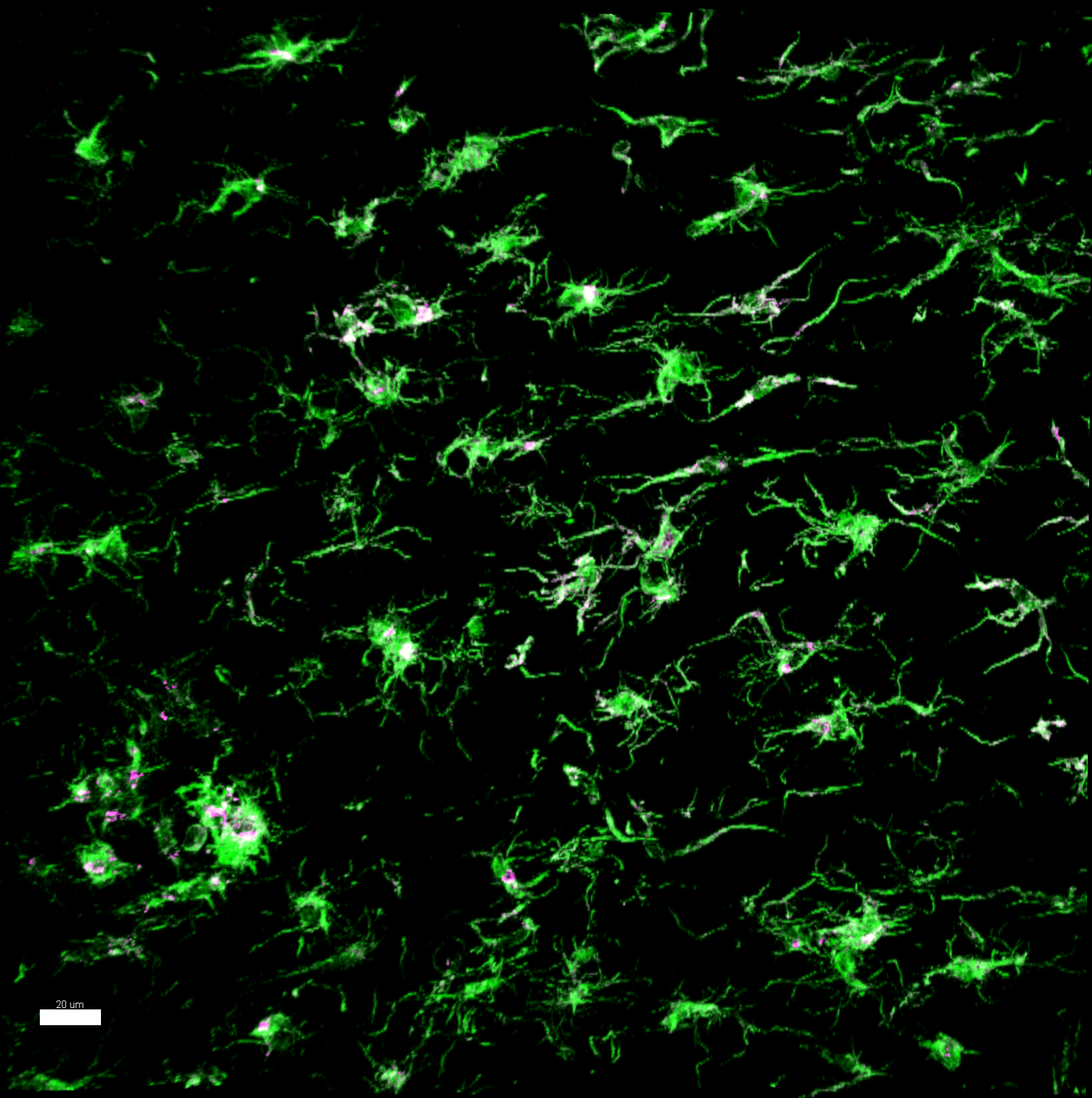

Supplement: Supplementary file 13 — Figure EV2 Source Data [file 44319_2026_721_MOESM13_ESM.zip › Figure EV2/EV2C/AXL/Cre+Arpc4floxed-merge-cortex.tif]

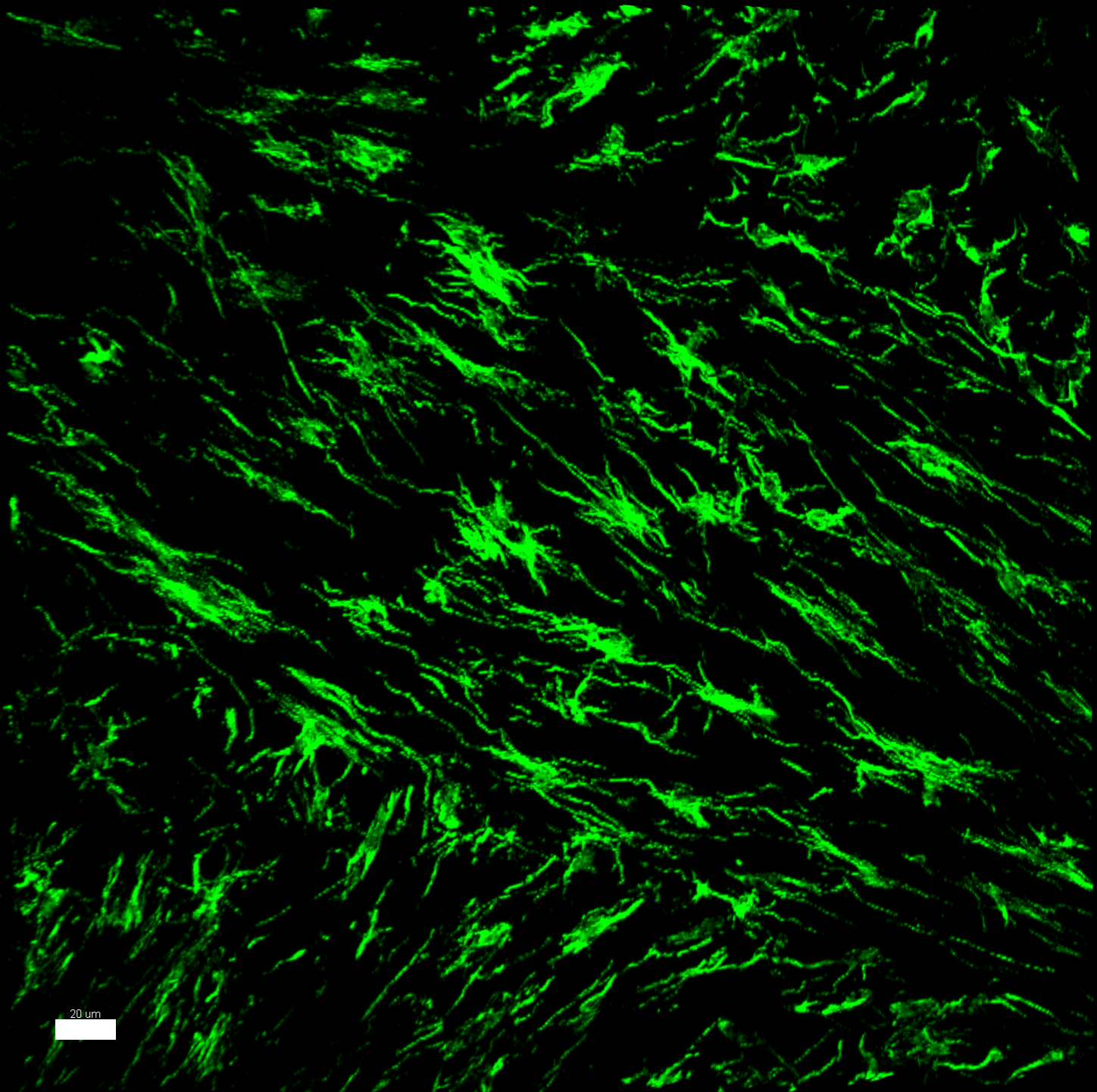

Supplement: Supplementary file 13 — Figure EV2 Source Data [file 44319_2026_721_MOESM13_ESM.zip › Figure EV2/EV2C/AXL/Cre+Arpc4floxed-IBA1-CC.tif]

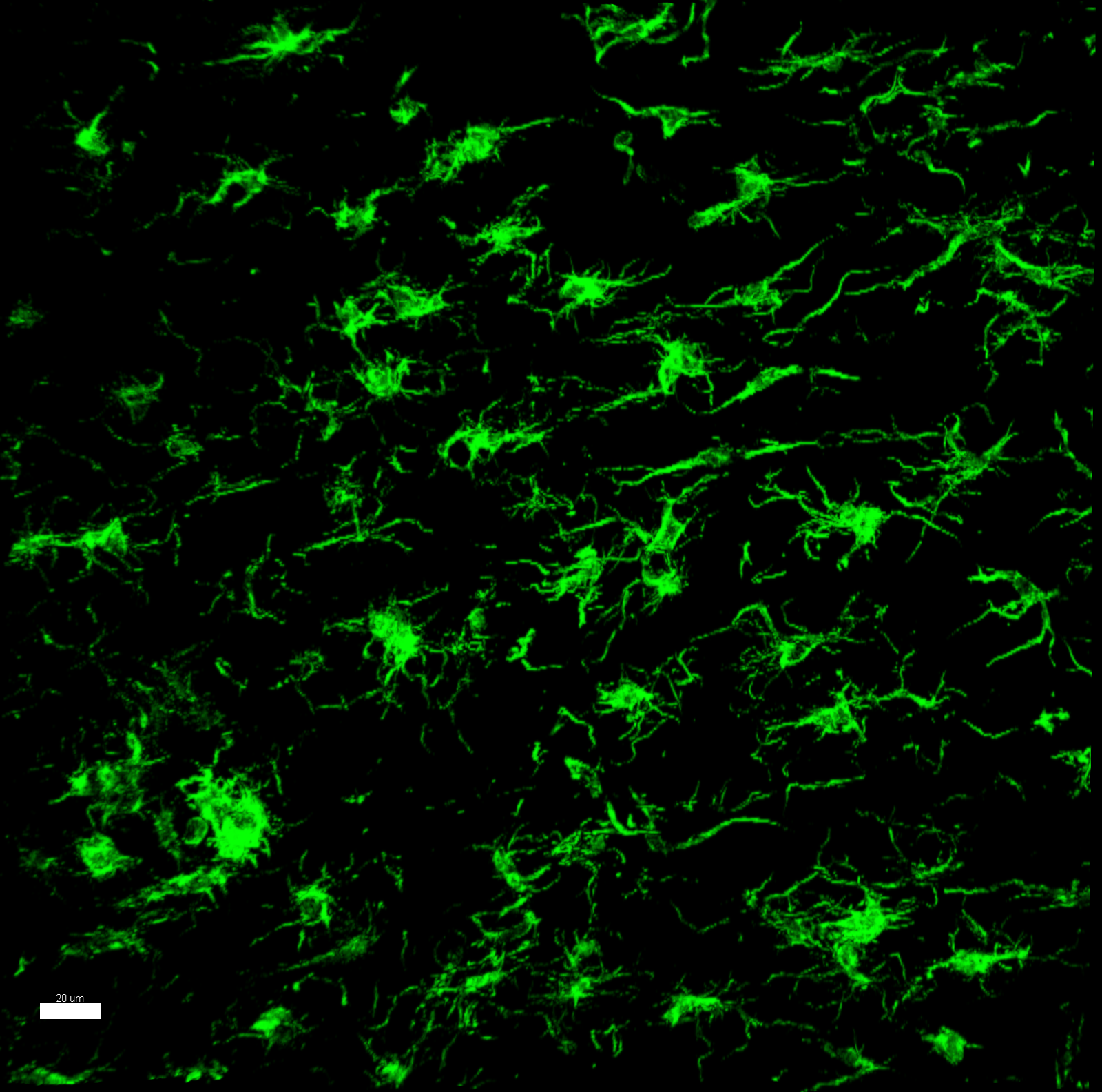

Supplement: Supplementary file 13 — Figure EV2 Source Data [file 44319_2026_721_MOESM13_ESM.zip › Figure EV2/EV2C/AXL/Cre+Arpc4floxed-IBA1-cortex.tif]

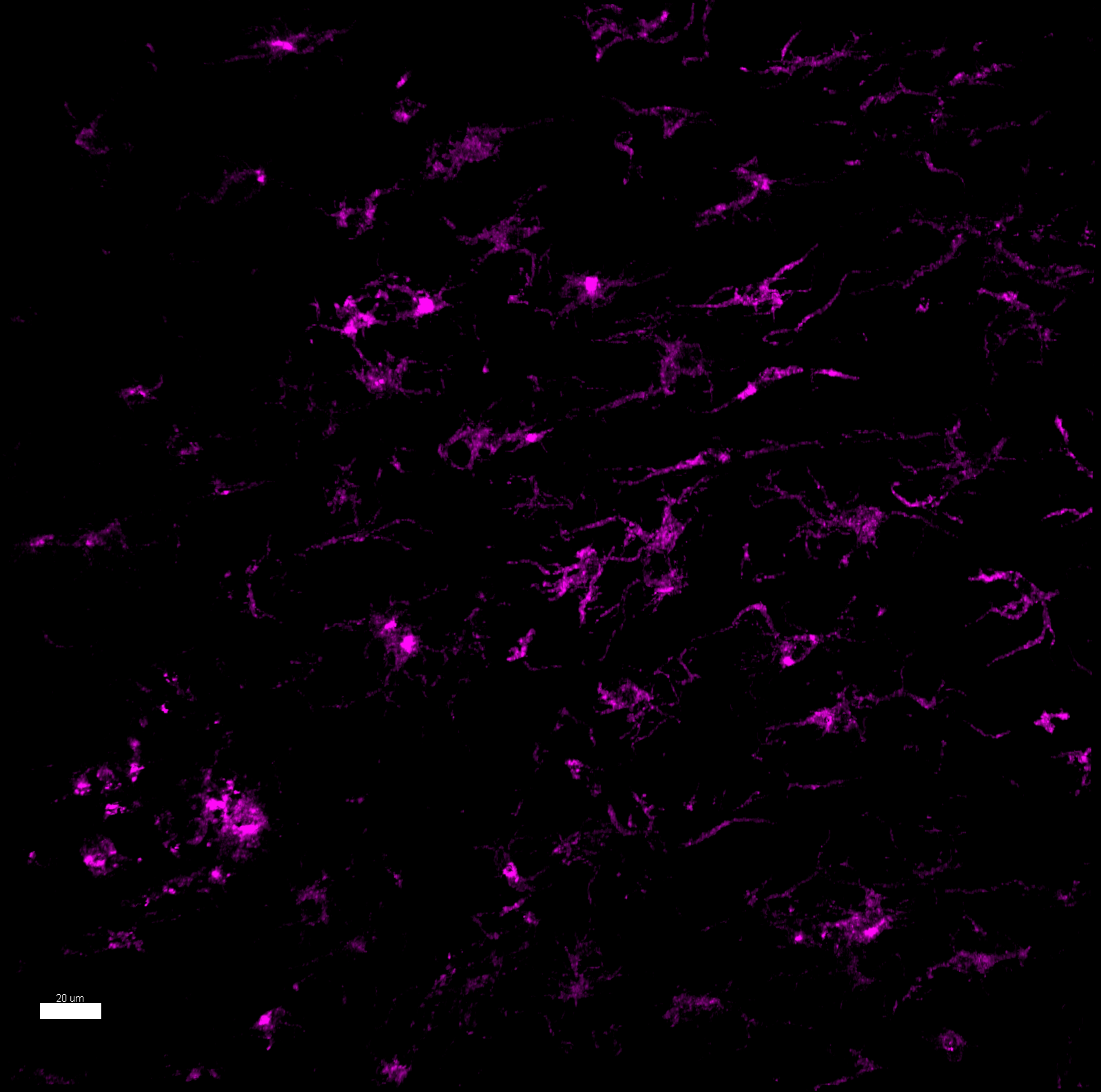

Supplement: Supplementary file 13 — Figure EV2 Source Data [file 44319_2026_721_MOESM13_ESM.zip › Figure EV2/EV2C/AXL/Cre+Arpc4floxed-AXL-cortex.tif]

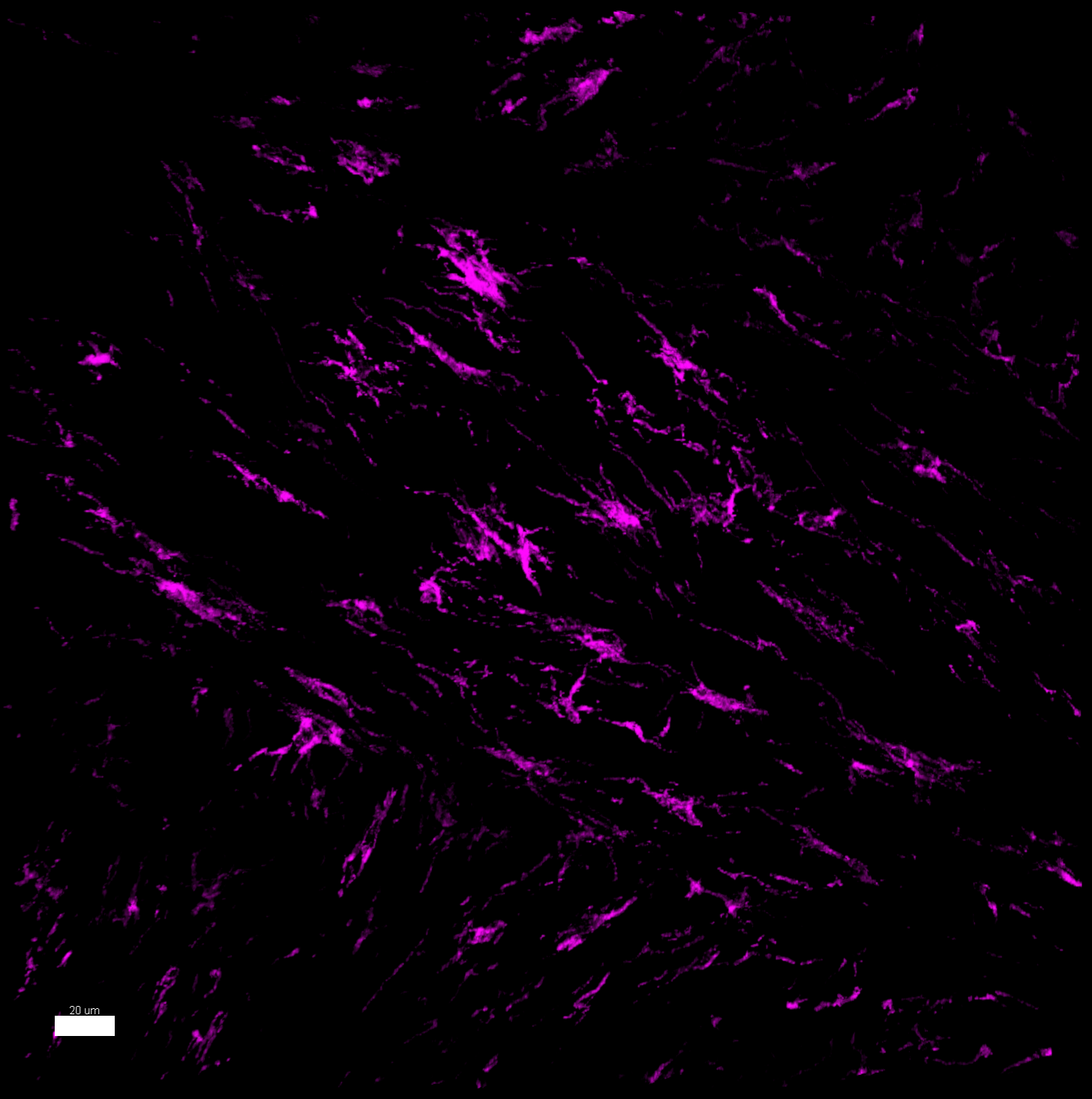

Supplement: Supplementary file 13 — Figure EV2 Source Data [file 44319_2026_721_MOESM13_ESM.zip › Figure EV2/EV2C/AXL/Cre+Arpc4floxed-AXL-CC.tif]

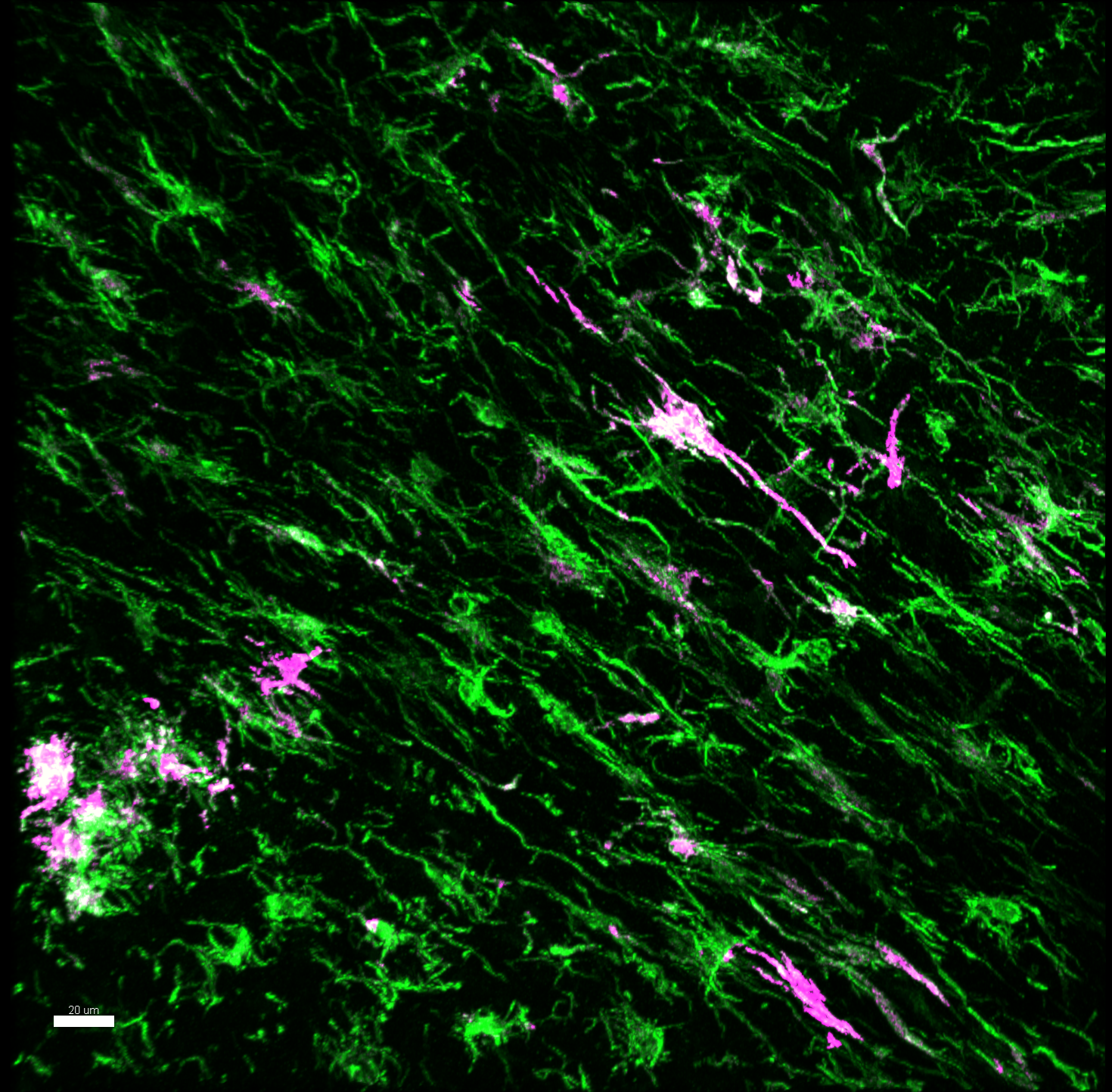

Supplement: Supplementary file 13 — Figure EV2 Source Data [file 44319_2026_721_MOESM13_ESM.zip › Figure EV2/EV2C/Galectin3/Cre+Arpc4floxed-merge-CC.tif]

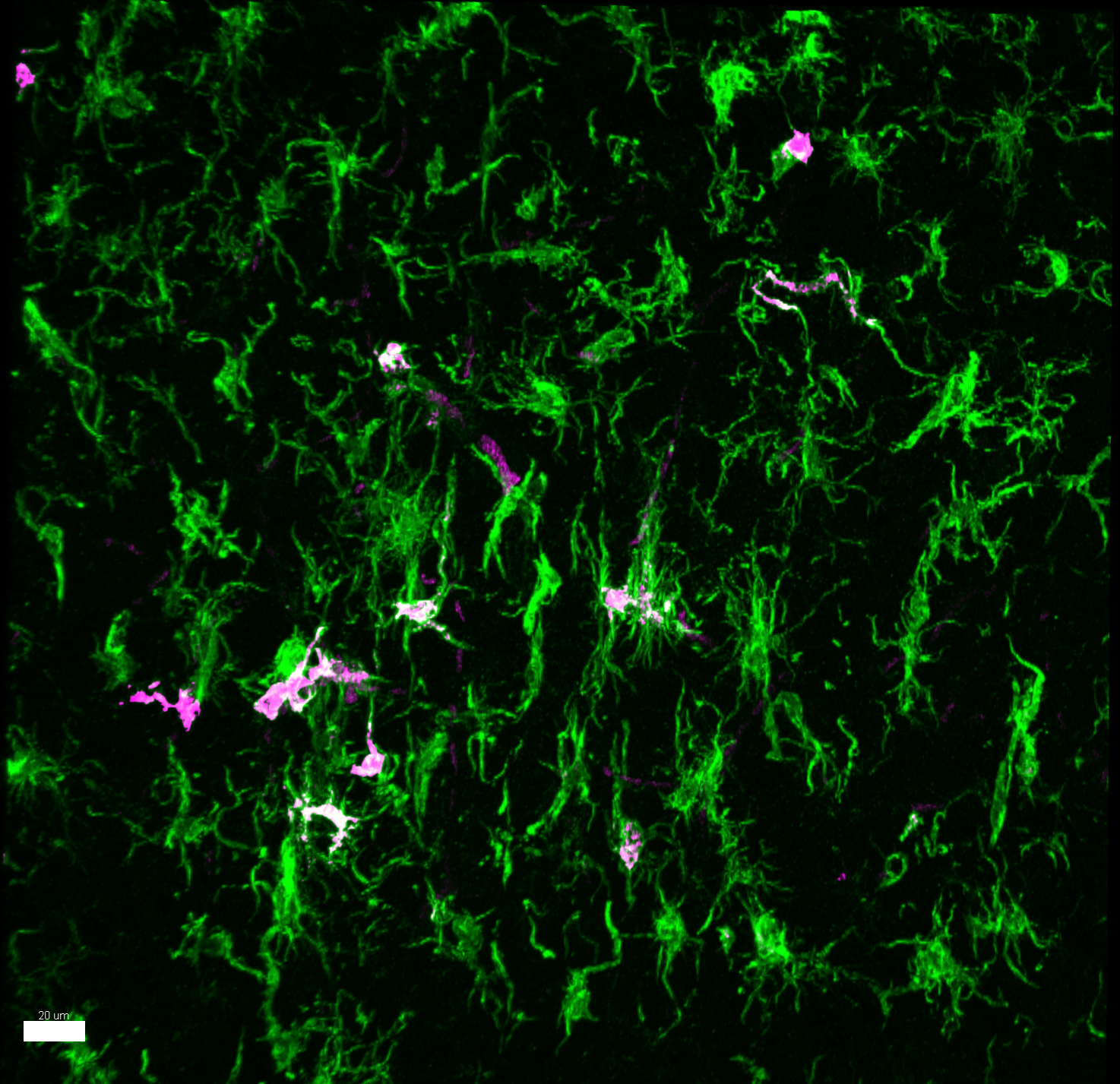

Supplement: Supplementary file 13 — Figure EV2 Source Data [file 44319_2026_721_MOESM13_ESM.zip › Figure EV2/EV2C/Galectin3/Cre+Arpc4floxed-merge-cortex.tif]

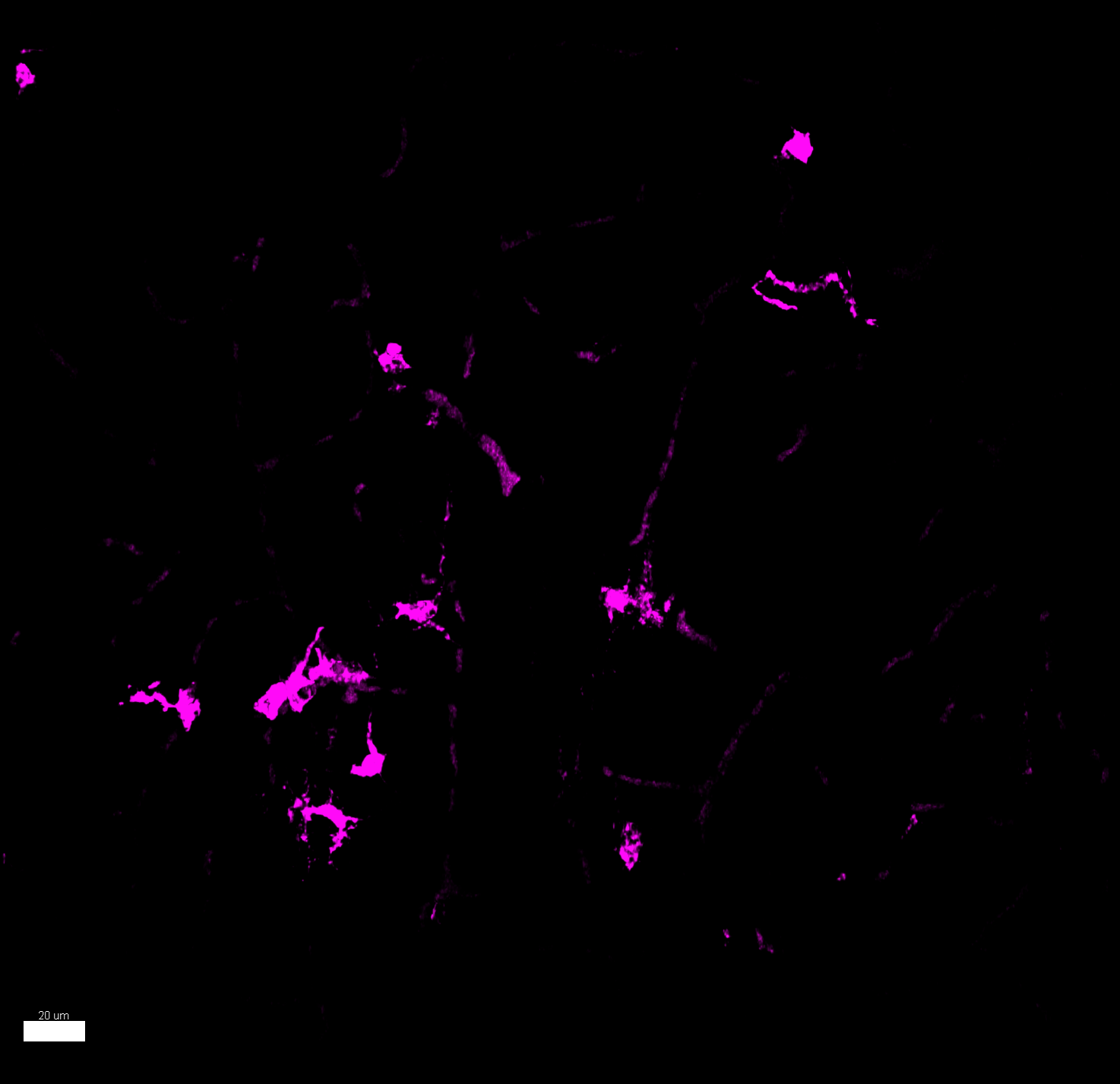

Supplement: Supplementary file 13 — Figure EV2 Source Data [file 44319_2026_721_MOESM13_ESM.zip › Figure EV2/EV2C/Galectin3/Cre+Arpc4floxed-Galectin3-cortex.tif]

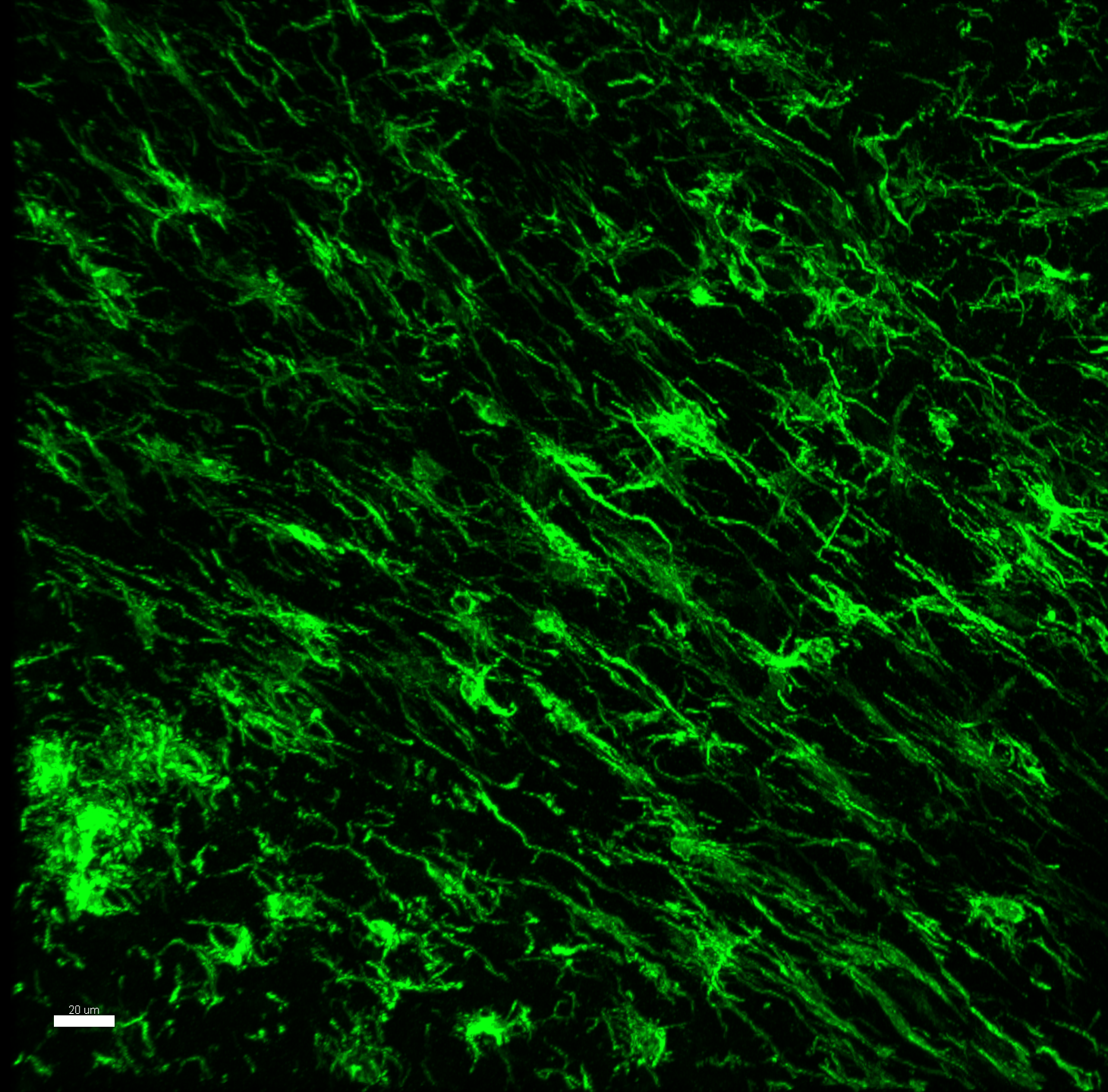

Supplement: Supplementary file 13 — Figure EV2 Source Data [file 44319_2026_721_MOESM13_ESM.zip › Figure EV2/EV2C/Galectin3/Cre+Arpc4floxed-IBA1-CC.tif]

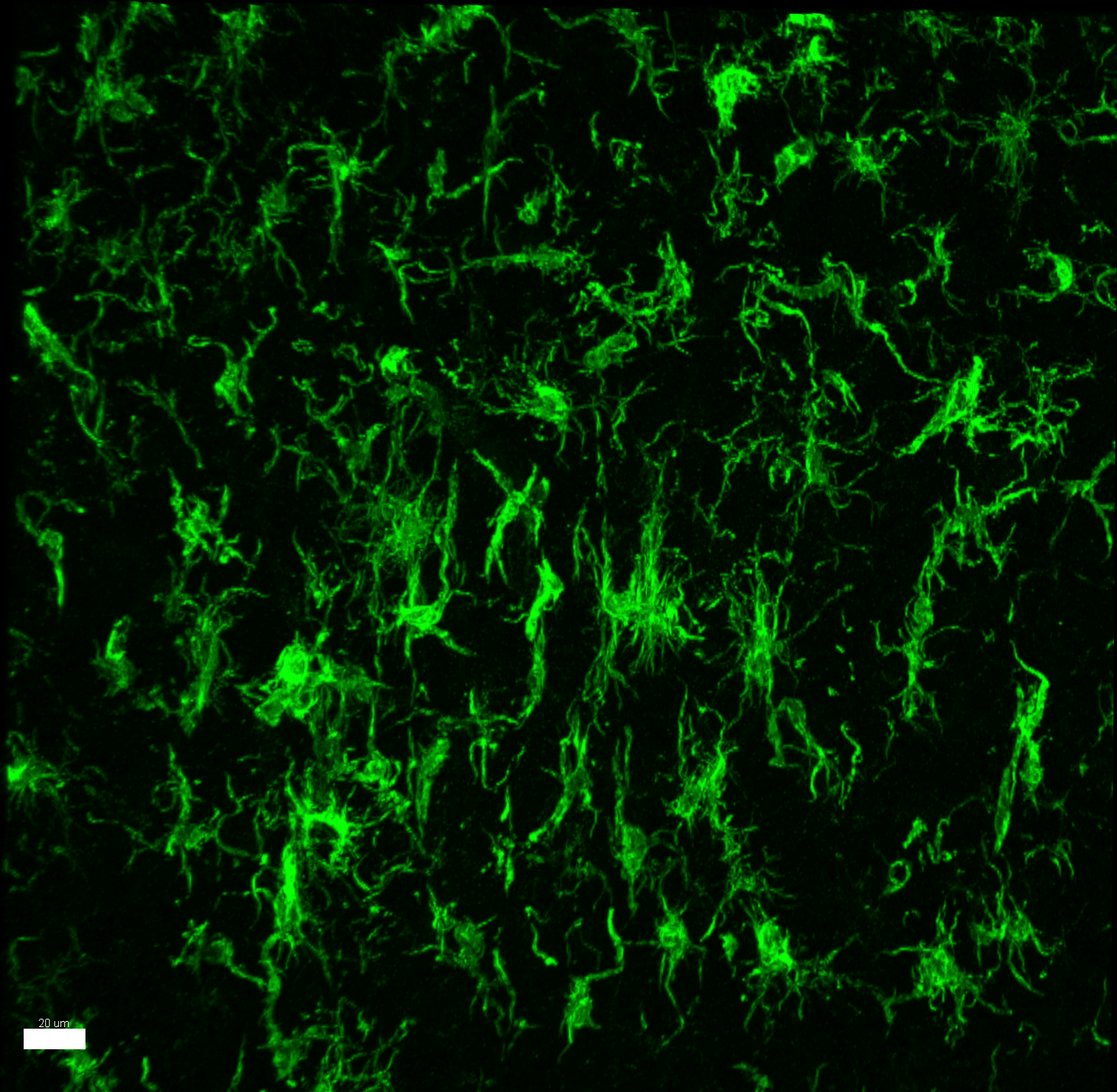

Supplement: Supplementary file 13 — Figure EV2 Source Data [file 44319_2026_721_MOESM13_ESM.zip › Figure EV2/EV2C/Galectin3/Cre+Arpc4floxed-IBA1-cortex.tif]

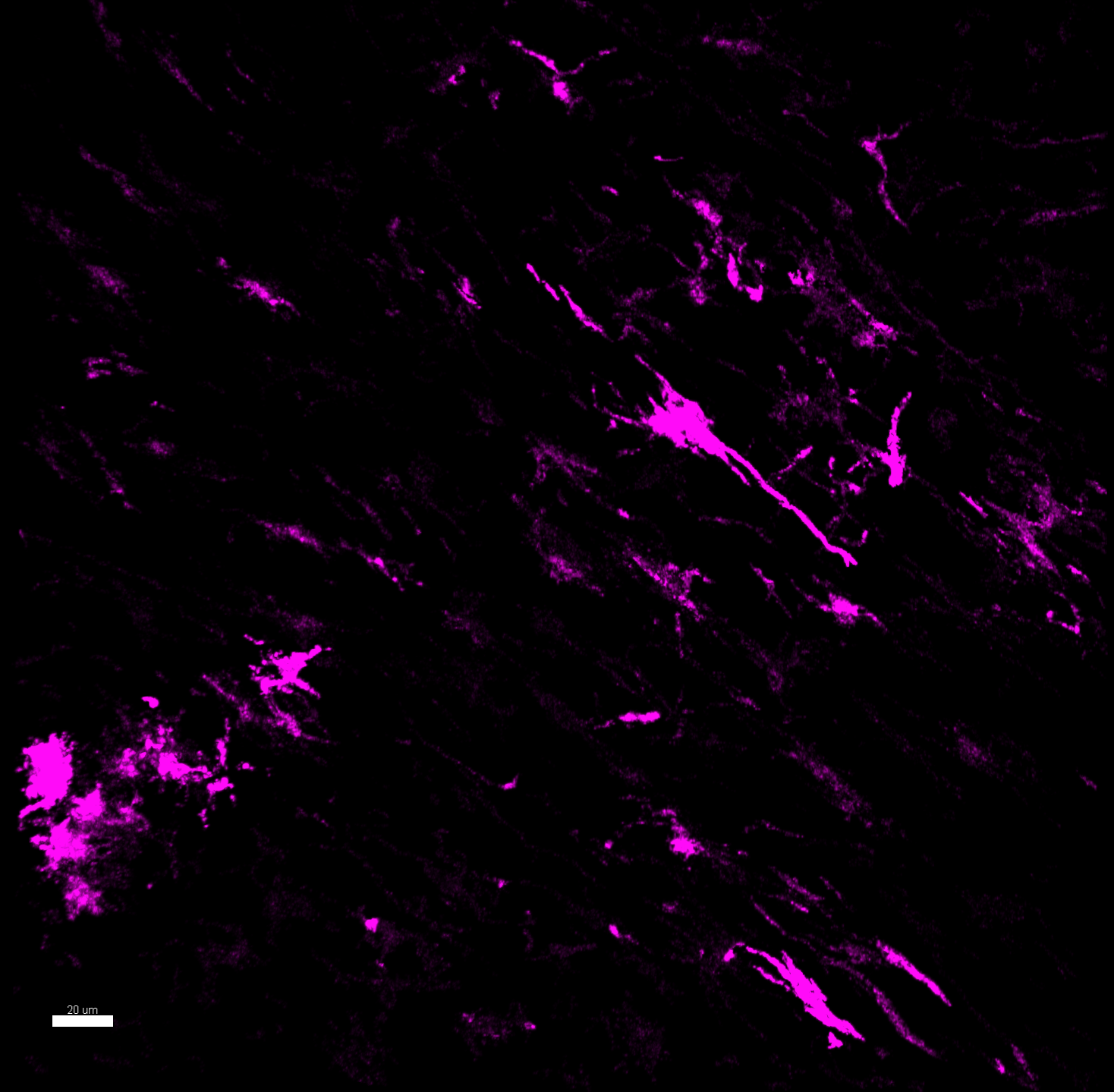

Supplement: Supplementary file 13 — Figure EV2 Source Data [file 44319_2026_721_MOESM13_ESM.zip › Figure EV2/EV2C/Galectin3/Cre+Arpc4floxed-Galectin3-CC.tif]

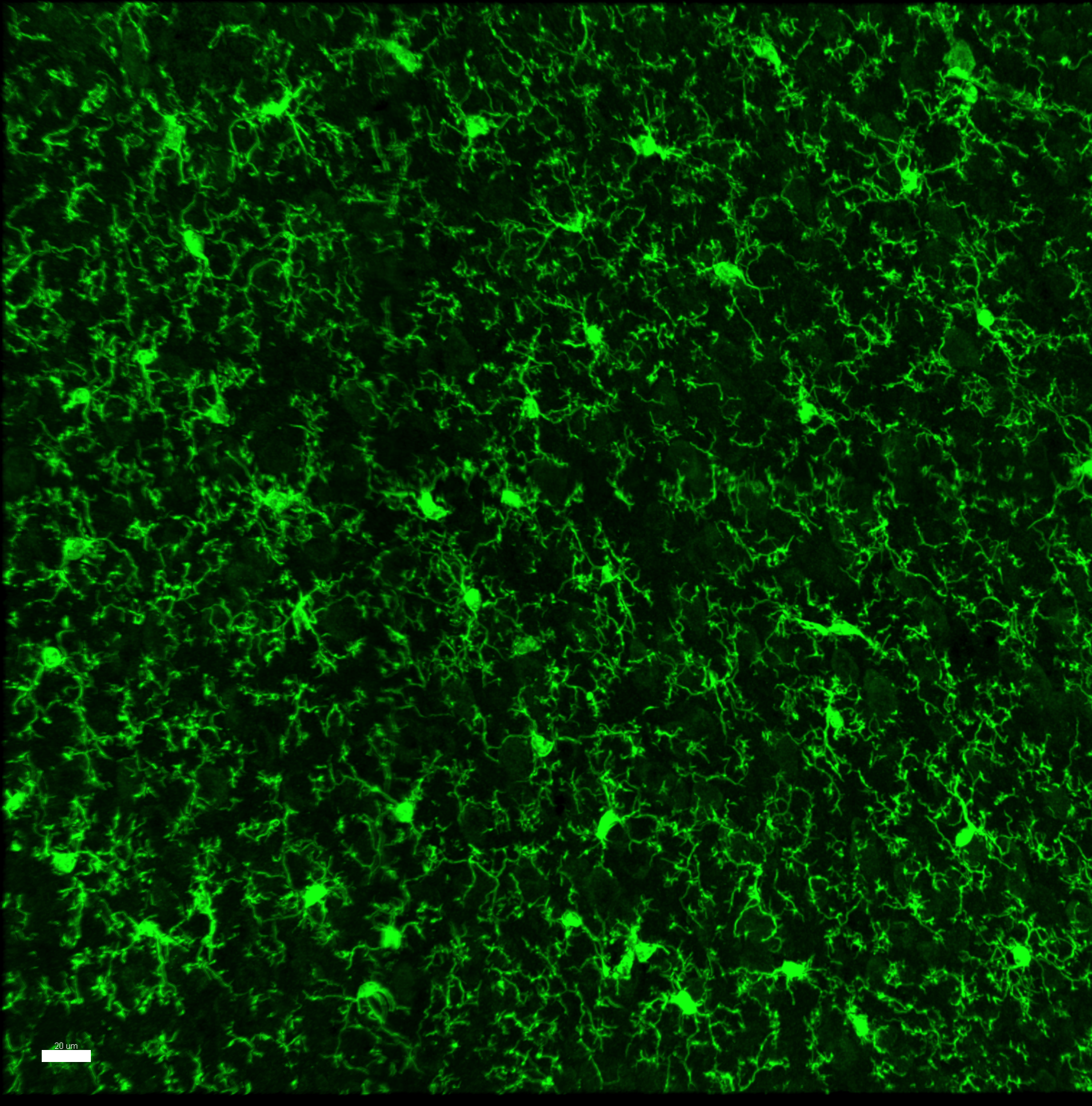

Supplement: Supplementary file 13 — Figure EV2 Source Data [file 44319_2026_721_MOESM13_ESM.zip › Figure EV2/EV2A/AXL/Cre-Arpc4floxed-IBA1-Cortex.tif]

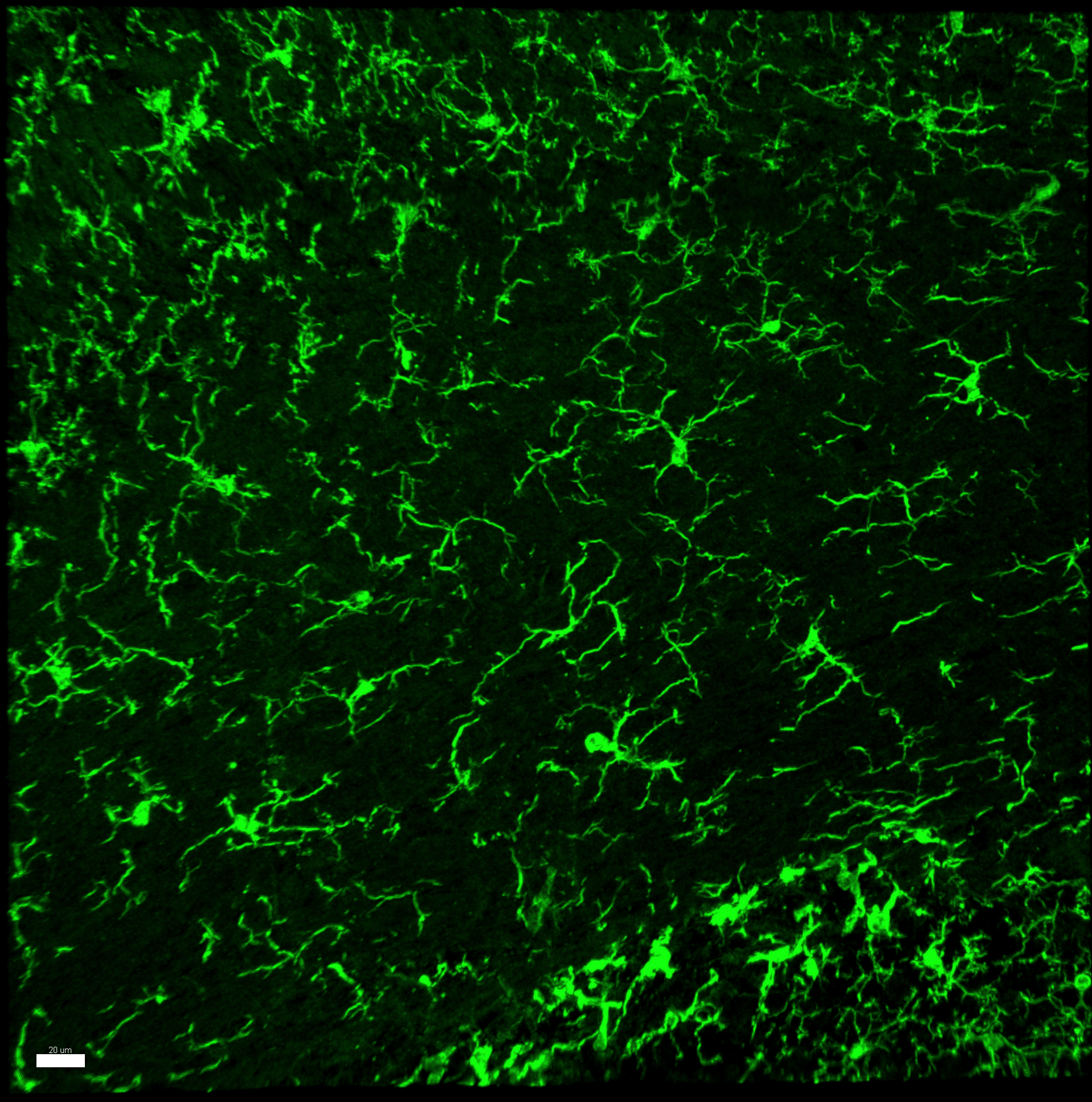

Supplement: Supplementary file 13 — Figure EV2 Source Data [file 44319_2026_721_MOESM13_ESM.zip › Figure EV2/EV2A/AXL/Cre-Arpc4floxed-IBA1-CC.tif]

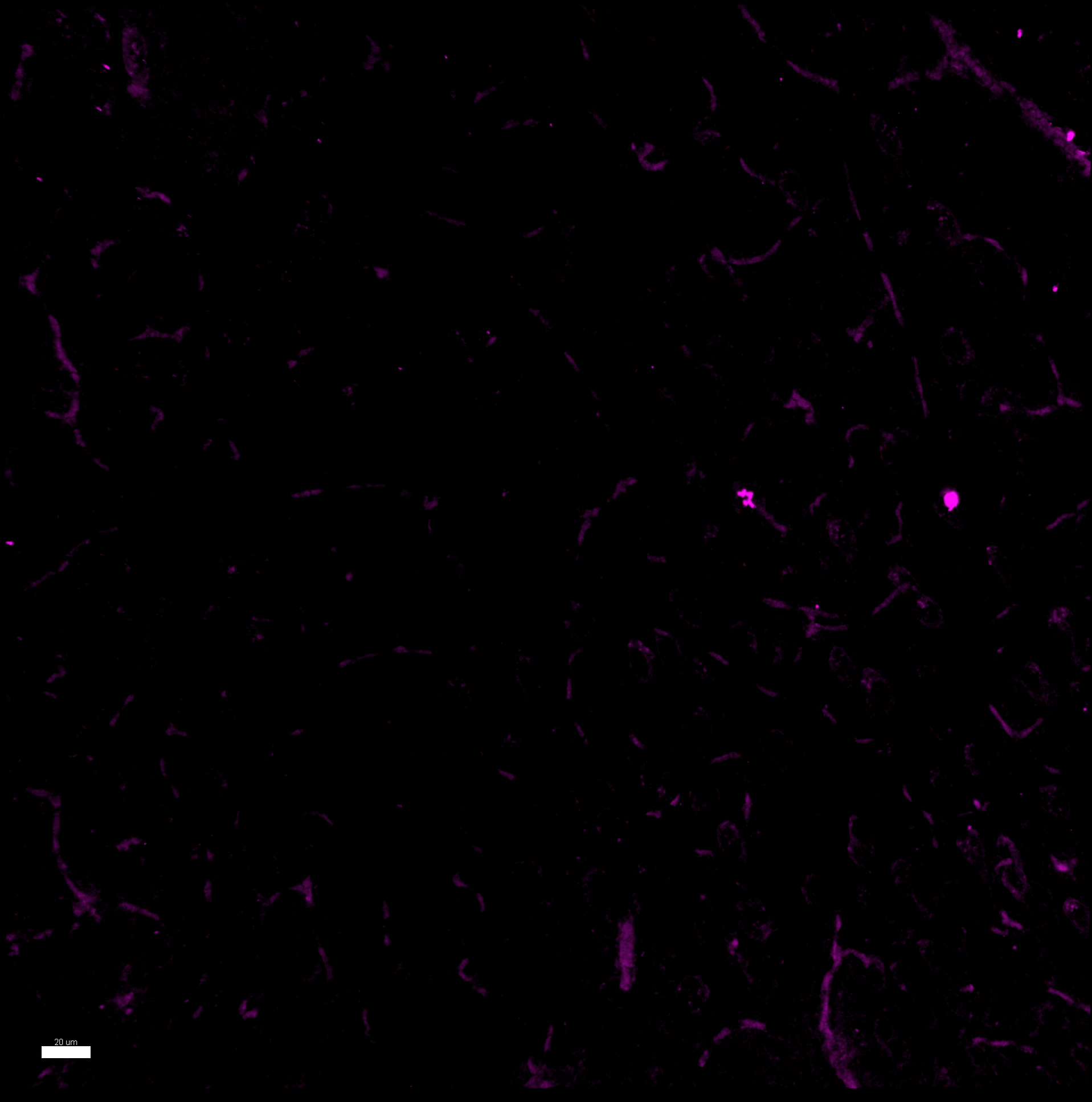

Supplement: Supplementary file 13 — Figure EV2 Source Data [file 44319_2026_721_MOESM13_ESM.zip › Figure EV2/EV2A/AXL/Cre-Arpc4floxed-AXL-Cortex.tif]

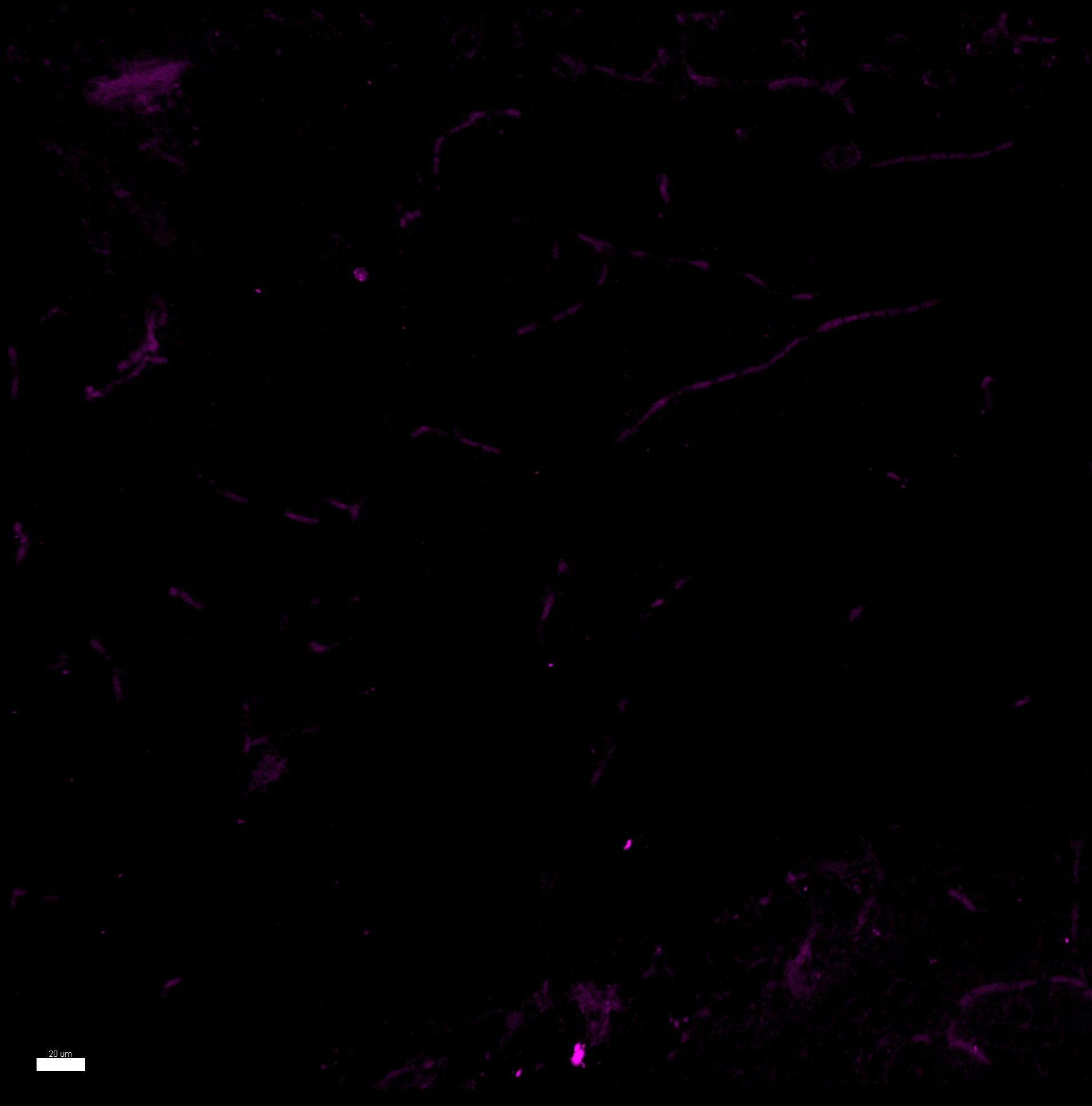

Supplement: Supplementary file 13 — Figure EV2 Source Data [file 44319_2026_721_MOESM13_ESM.zip › Figure EV2/EV2A/AXL/Cre-Arpc4floxed-AXL-CC.tif]

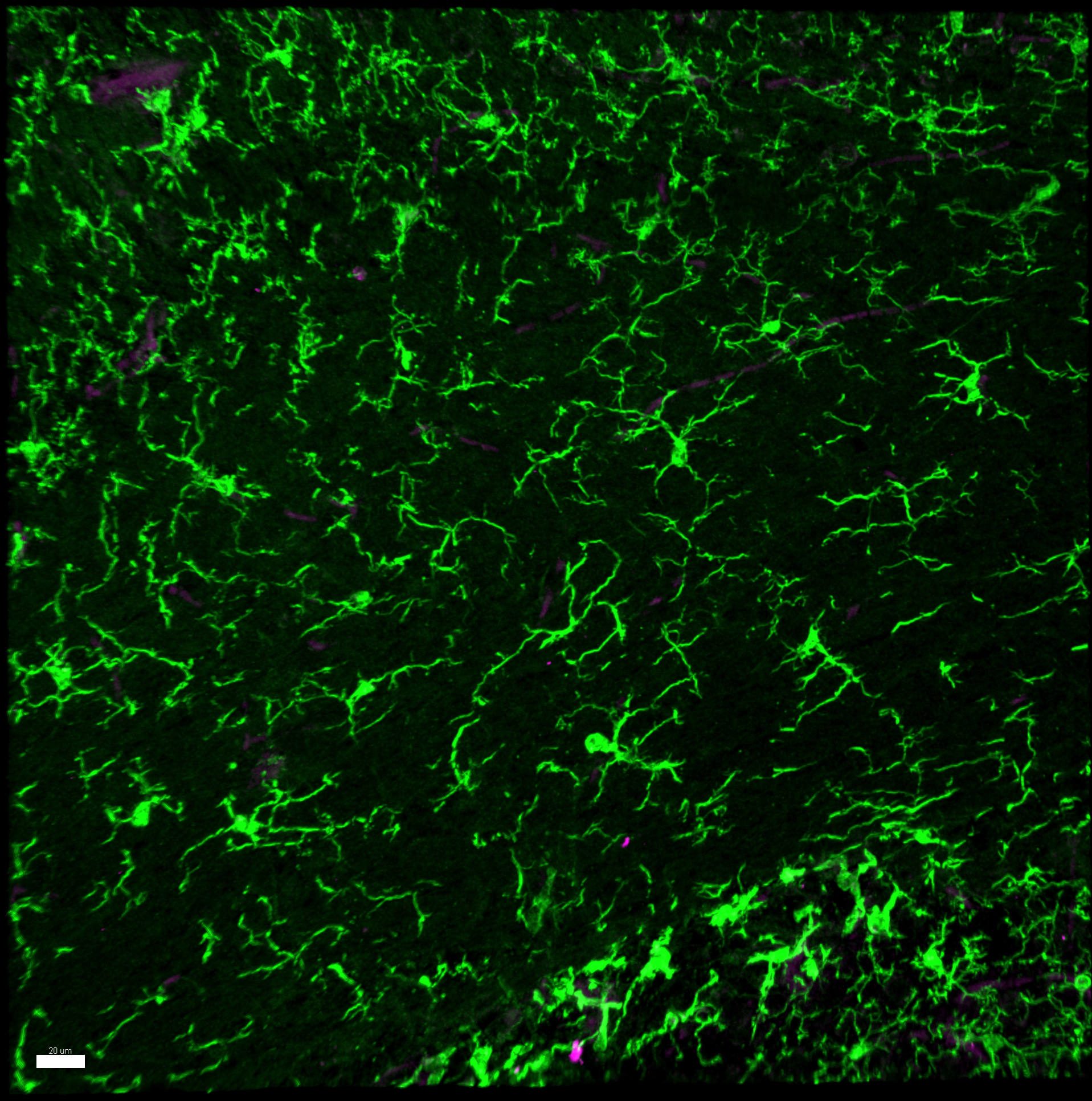

Supplement: Supplementary file 13 — Figure EV2 Source Data [file 44319_2026_721_MOESM13_ESM.zip › Figure EV2/EV2A/AXL/Cre-Arpc4floxed-merge-CC.tif]

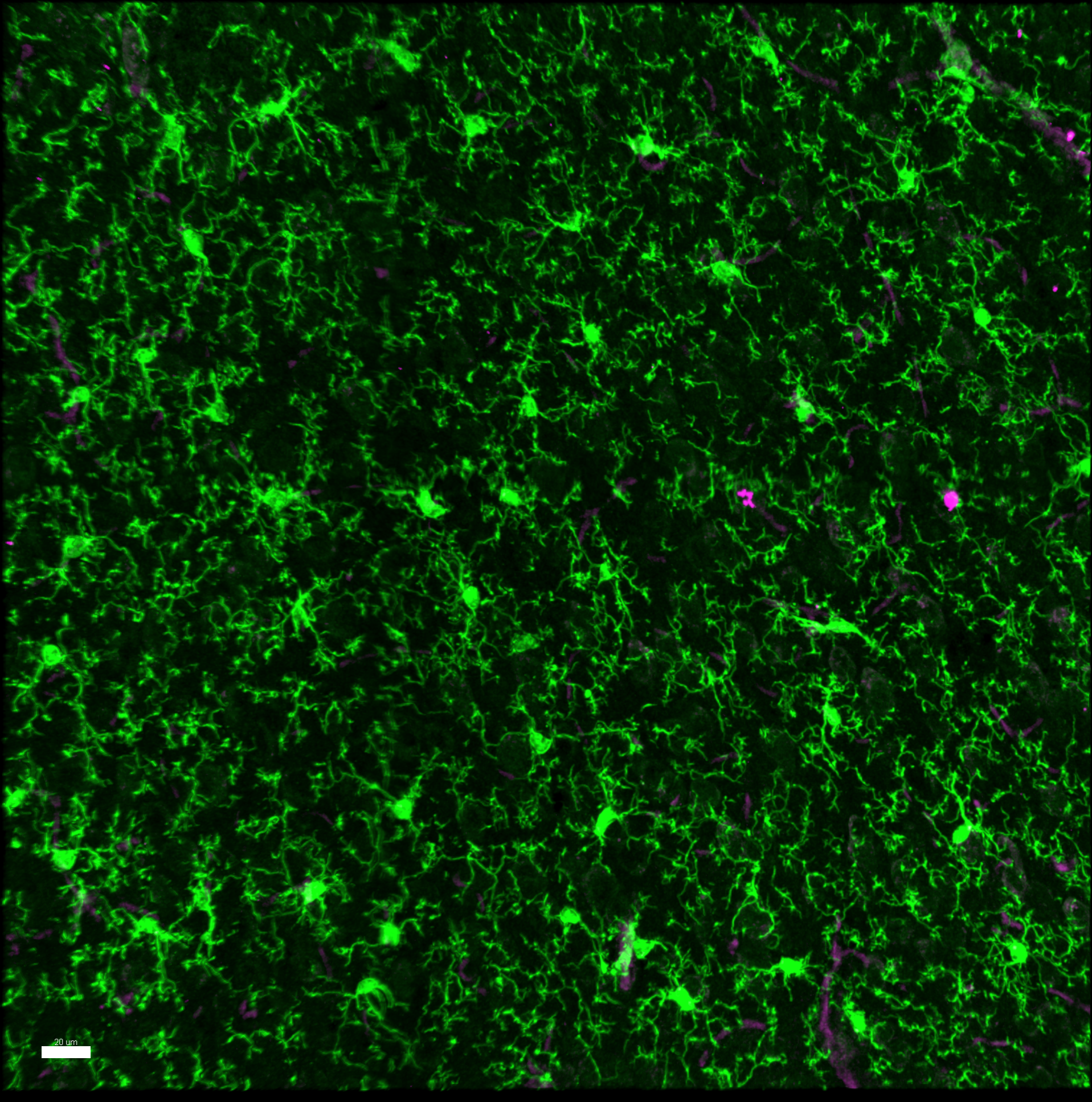

Supplement: Supplementary file 13 — Figure EV2 Source Data [file 44319_2026_721_MOESM13_ESM.zip › Figure EV2/EV2A/AXL/Cre-Arpc4floxed-merge-Cortex.tif]

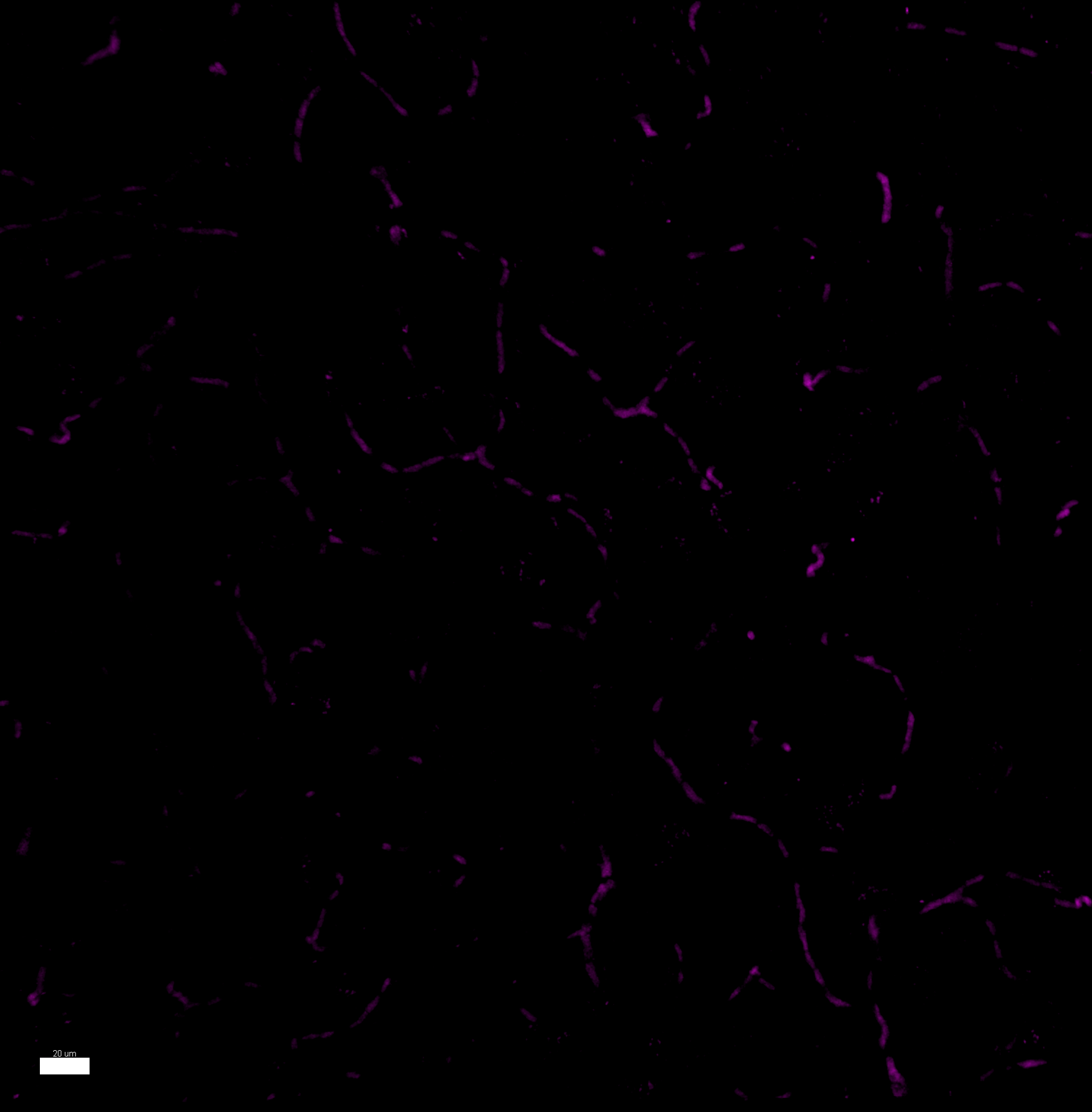

Supplement: Supplementary file 13 — Figure EV2 Source Data [file 44319_2026_721_MOESM13_ESM.zip › Figure EV2/EV2A/Galectin3/Cre- Arpc4floxed-Galectin3-cortex.tif]

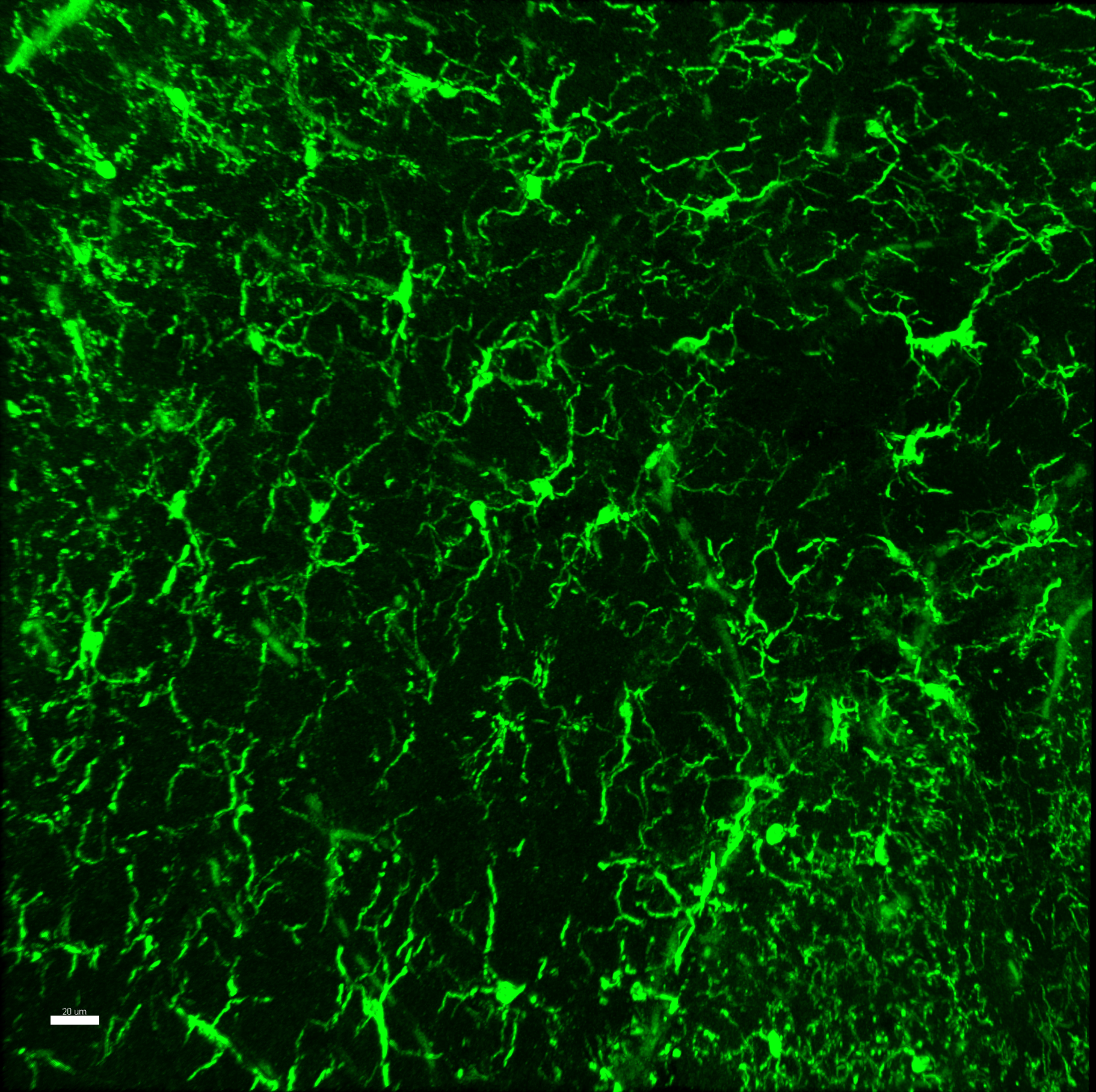

Supplement: Supplementary file 13 — Figure EV2 Source Data [file 44319_2026_721_MOESM13_ESM.zip › Figure EV2/EV2A/Galectin3/Cre- Arpc4floxed-IBA1-CC.tif]

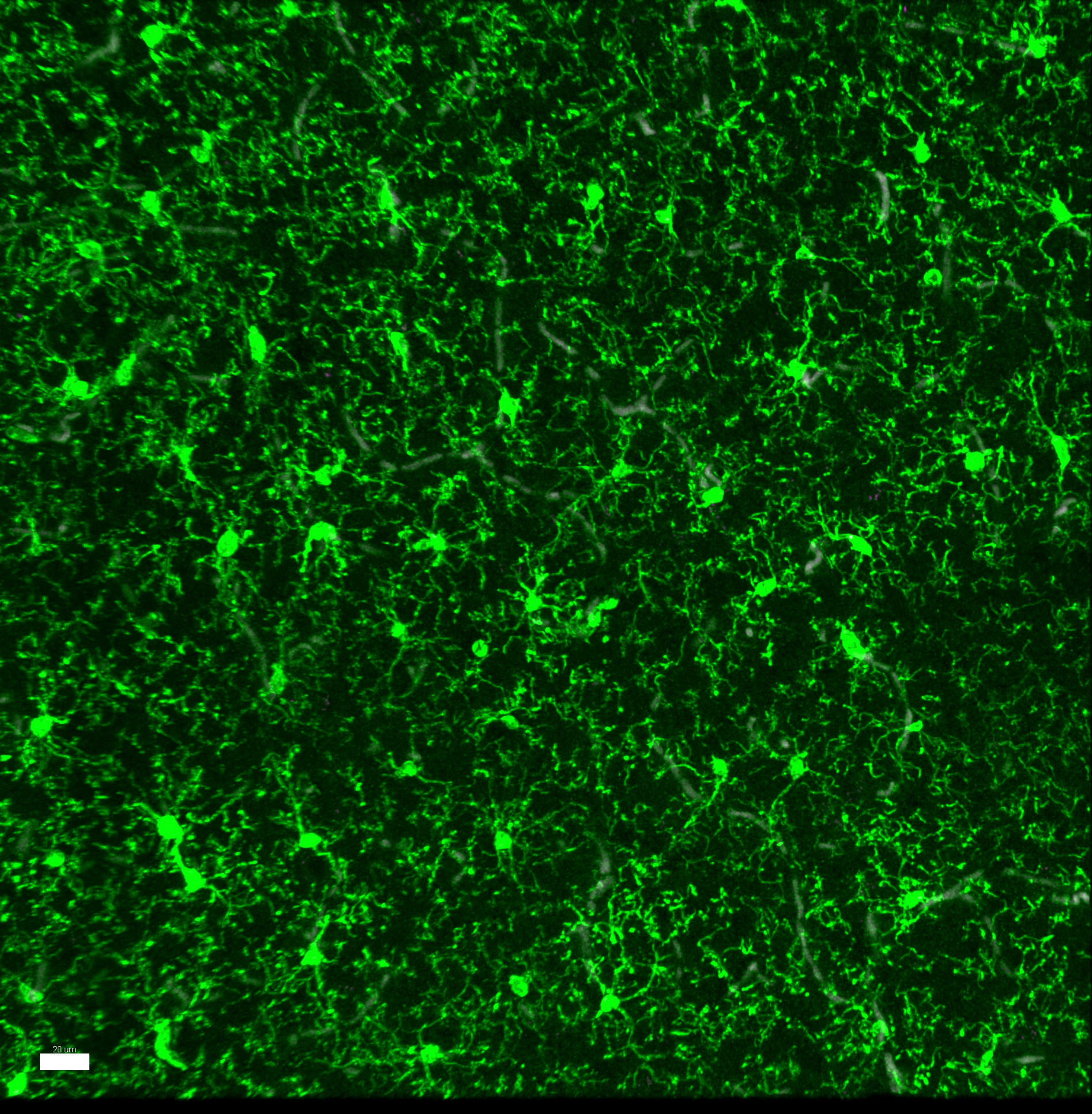

Supplement: Supplementary file 13 — Figure EV2 Source Data [file 44319_2026_721_MOESM13_ESM.zip › Figure EV2/EV2A/Galectin3/Cre- Arpc4floxed-merge-cortex.tif]

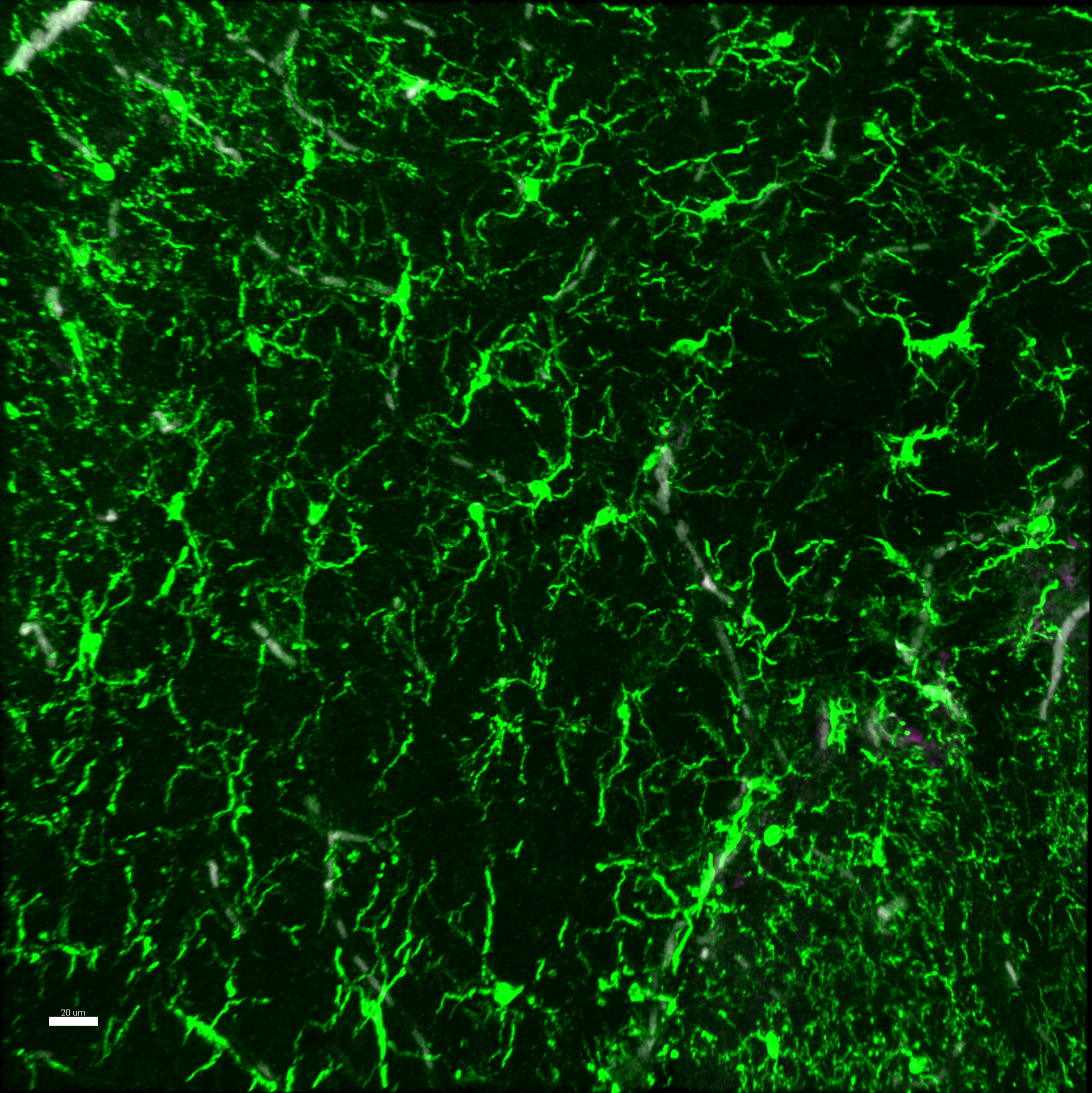

Supplement: Supplementary file 13 — Figure EV2 Source Data [file 44319_2026_721_MOESM13_ESM.zip › Figure EV2/EV2A/Galectin3/Cre- Arpc4floxed-merge-CC.tif]

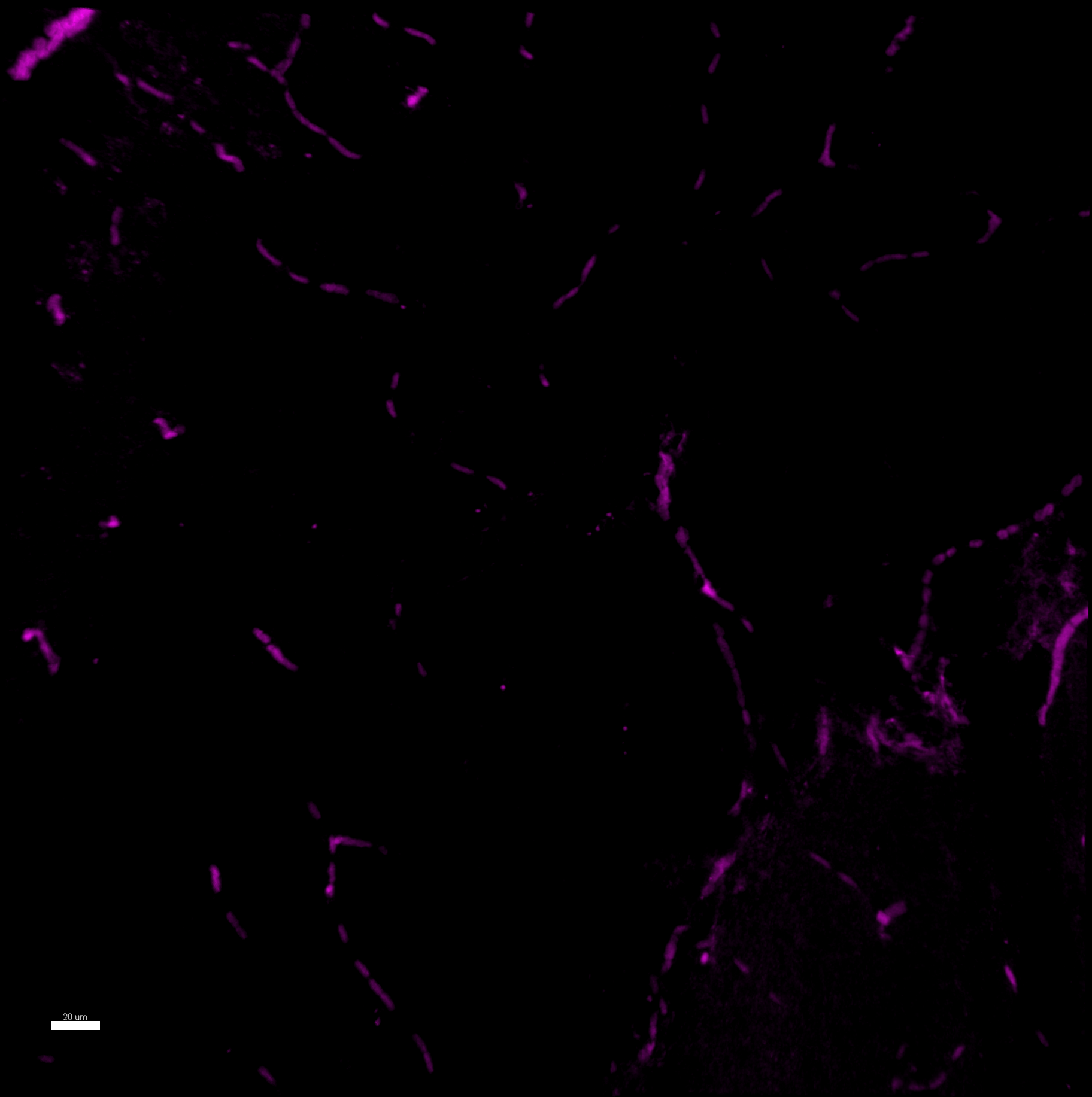

Supplement: Supplementary file 13 — Figure EV2 Source Data [file 44319_2026_721_MOESM13_ESM.zip › Figure EV2/EV2A/Galectin3/Cre- Arpc4floxed-Galectin3-CC.tif]

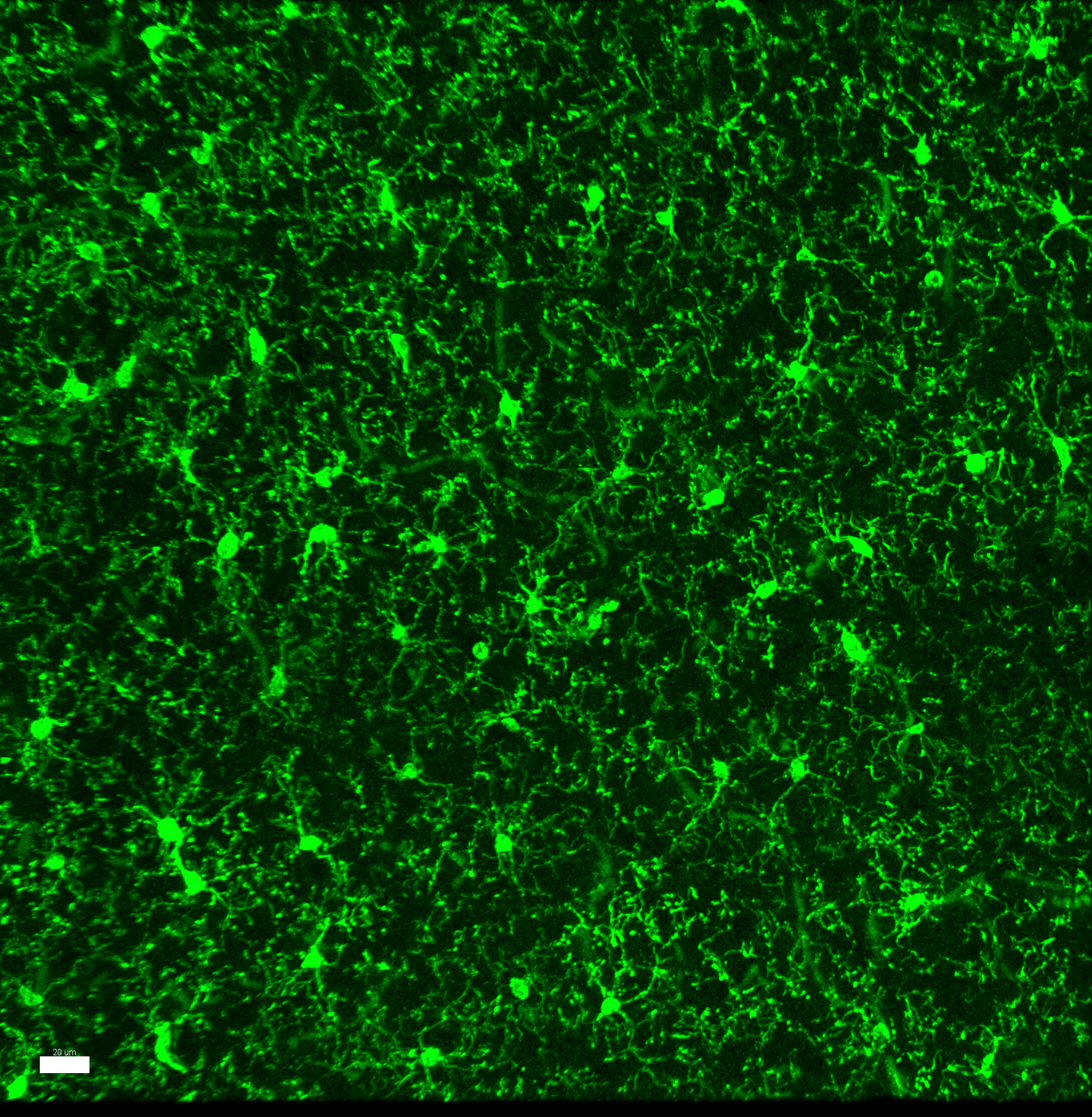

Supplement: Supplementary file 13 — Figure EV2 Source Data [file 44319_2026_721_MOESM13_ESM.zip › Figure EV2/EV2A/Galectin3/Cre- Arpc4floxed-IBA1-cotrex.tif]

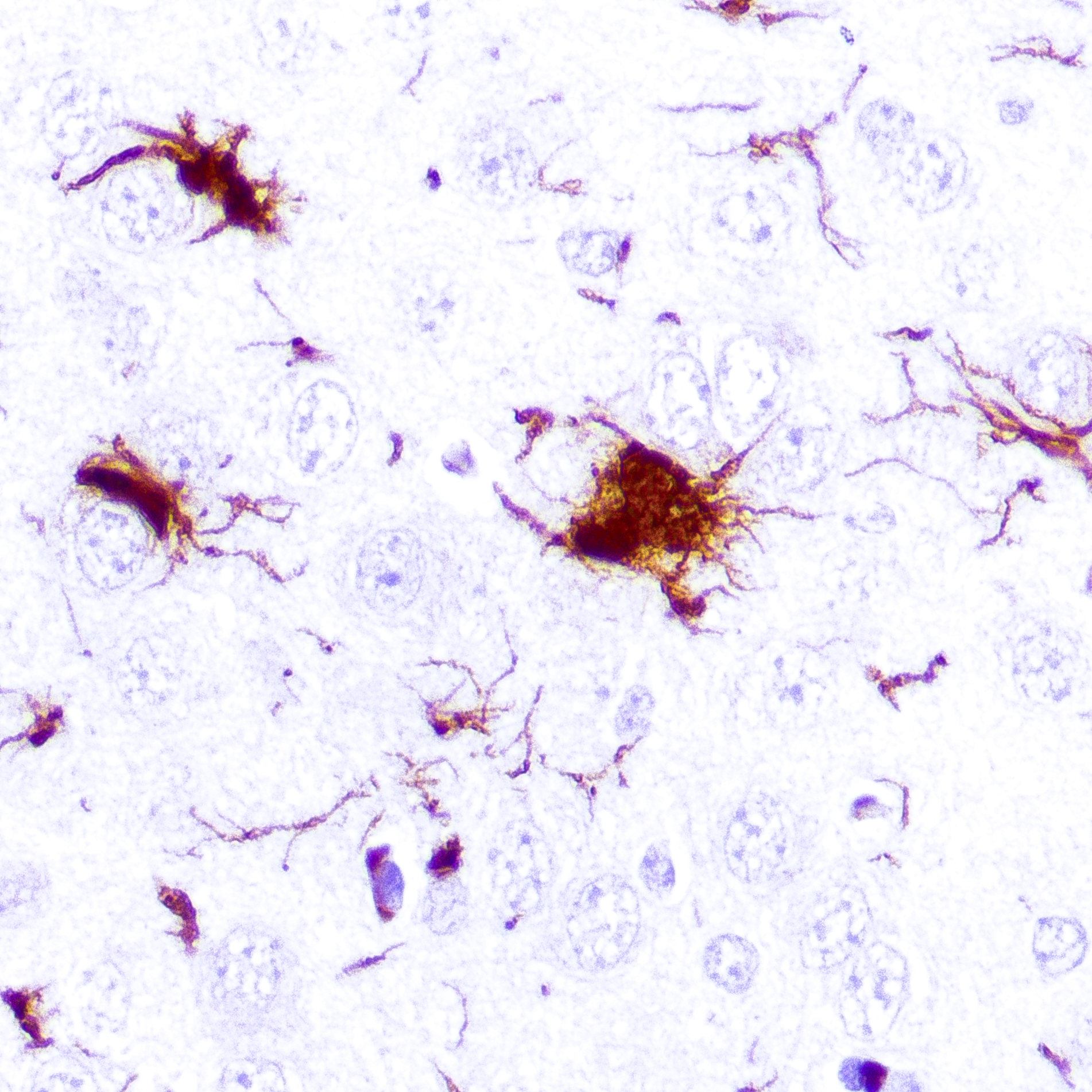

Supplement: Supplementary file 14 — Figure EV3 Source Data [file 44319_2026_721_MOESM14_ESM.zip › Figure EV3/EV3A/KO/Ctx_LayerV-VI-2.tif]

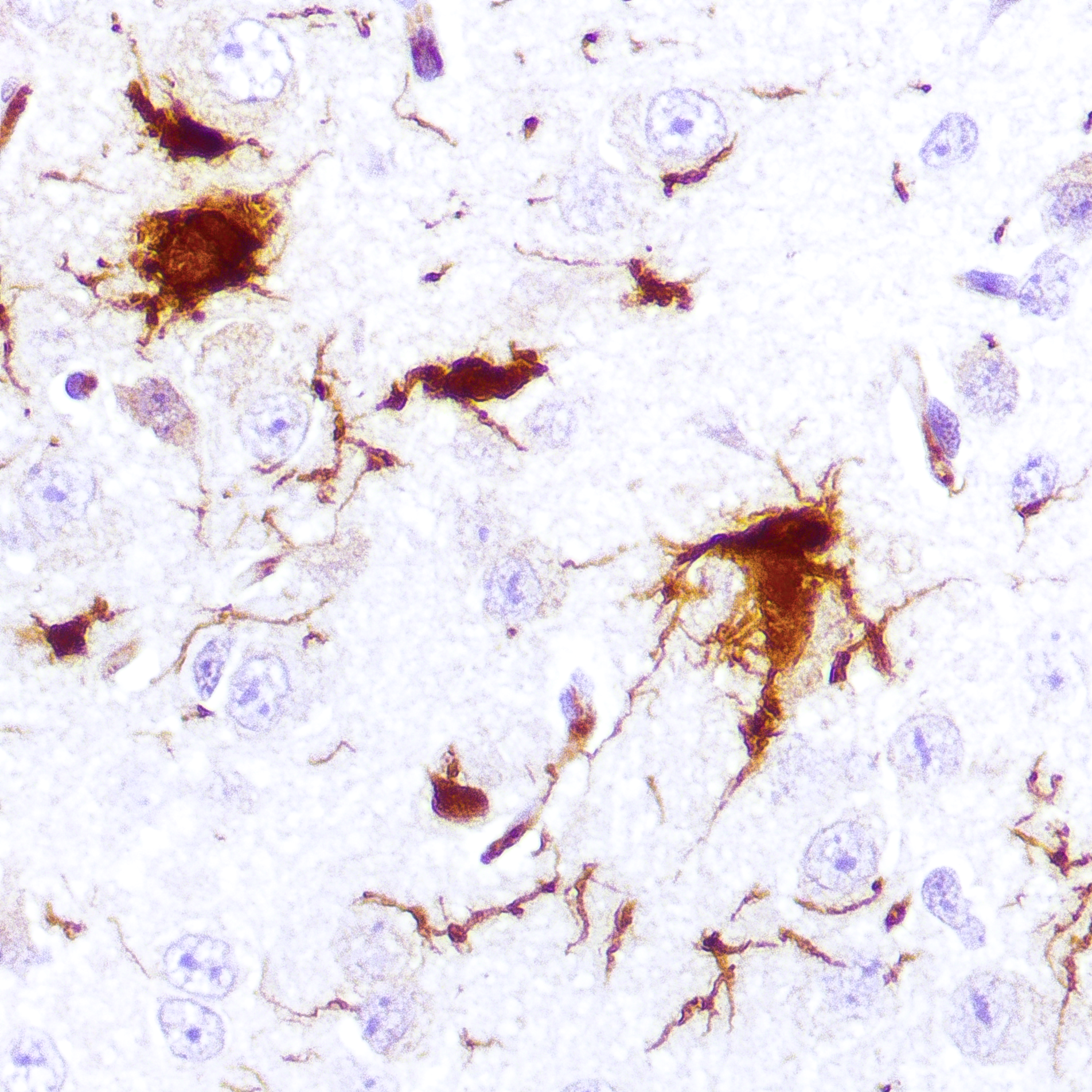

Supplement: Supplementary file 14 — Figure EV3 Source Data [file 44319_2026_721_MOESM14_ESM.zip › Figure EV3/EV3A/KO/Striatum-2.tif]

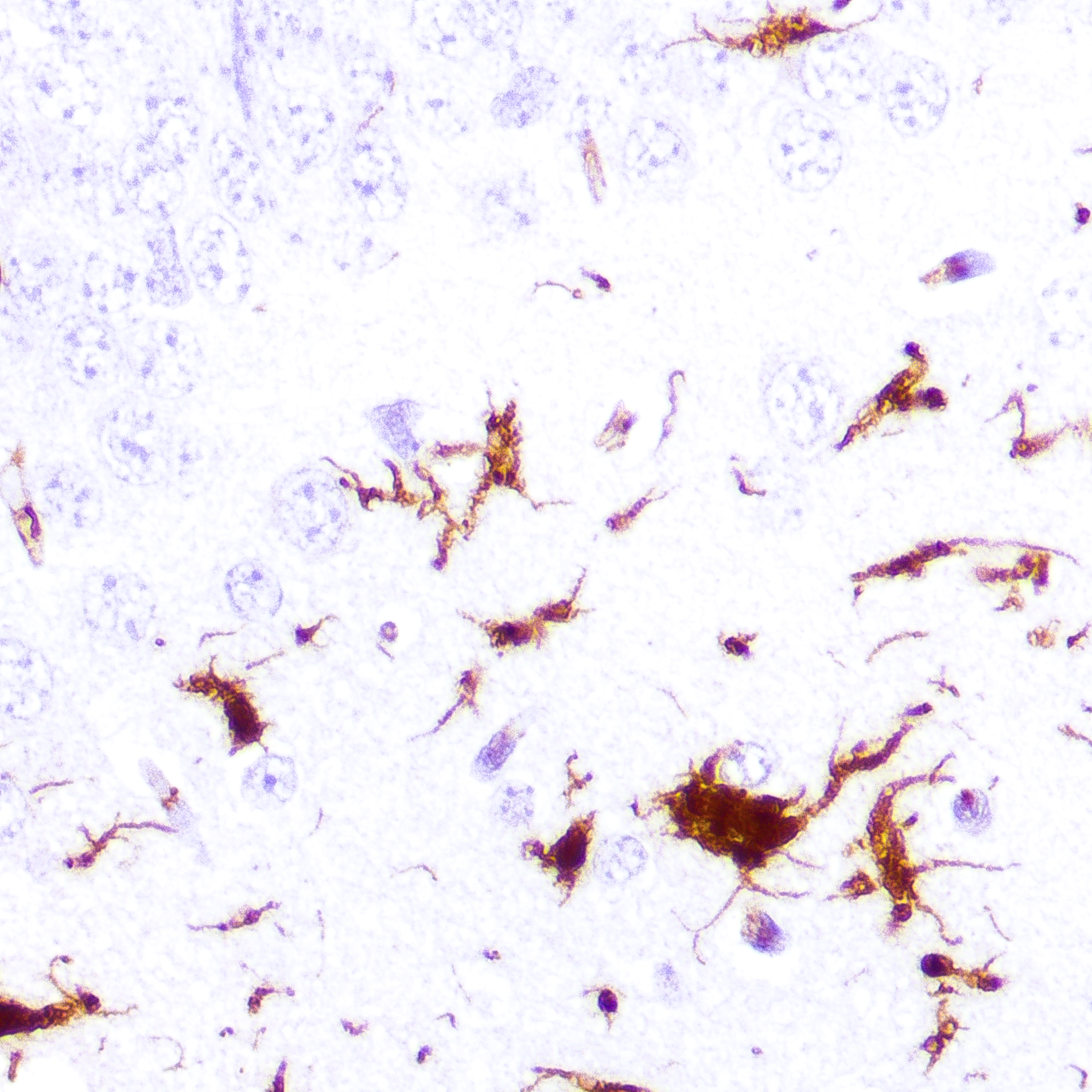

Supplement: Supplementary file 14 — Figure EV3 Source Data [file 44319_2026_721_MOESM14_ESM.zip › Figure EV3/EV3A/KO/Hippocampus.tif]

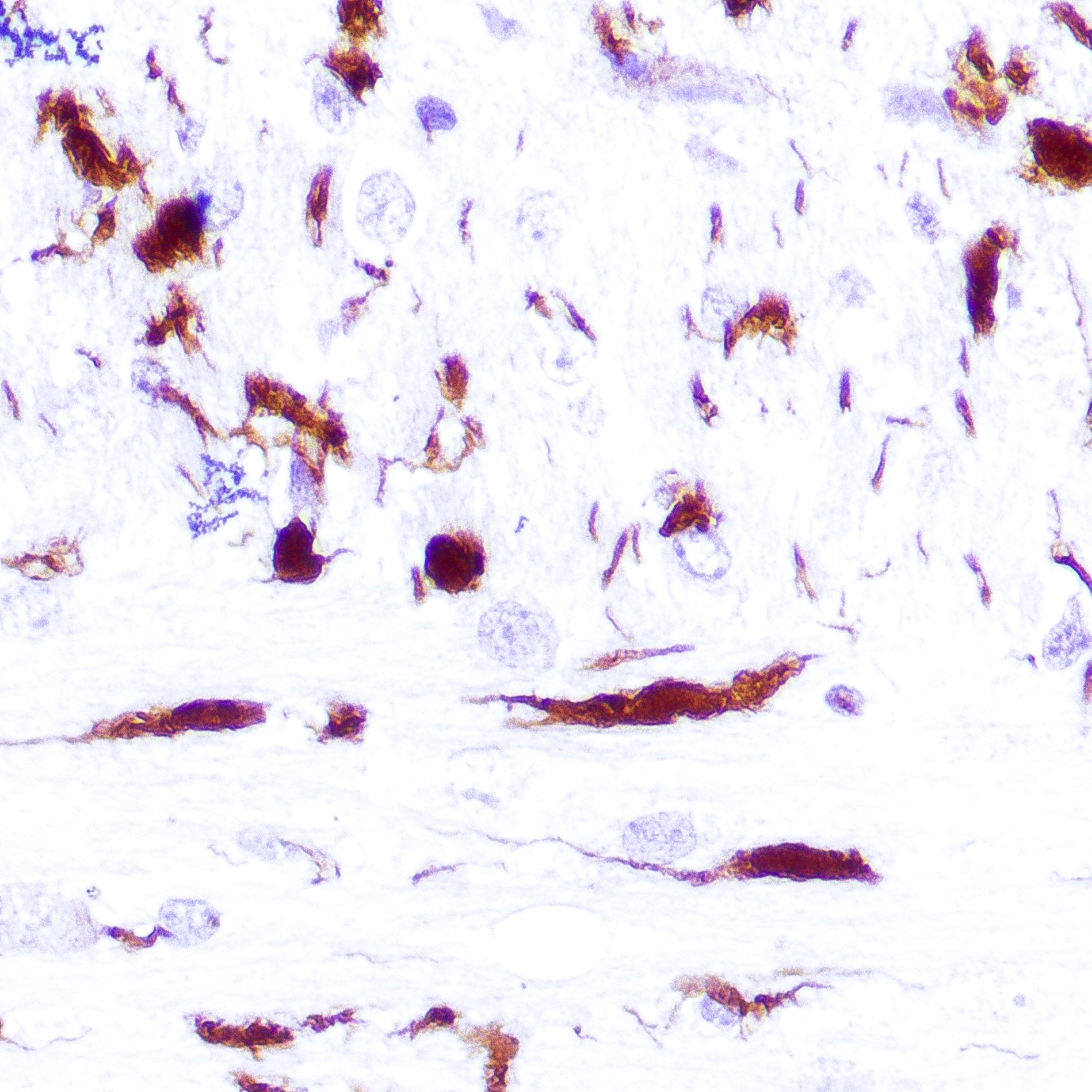

Supplement: Supplementary file 14 — Figure EV3 Source Data [file 44319_2026_721_MOESM14_ESM.zip › Figure EV3/EV3A/KO/CC-2.tif]

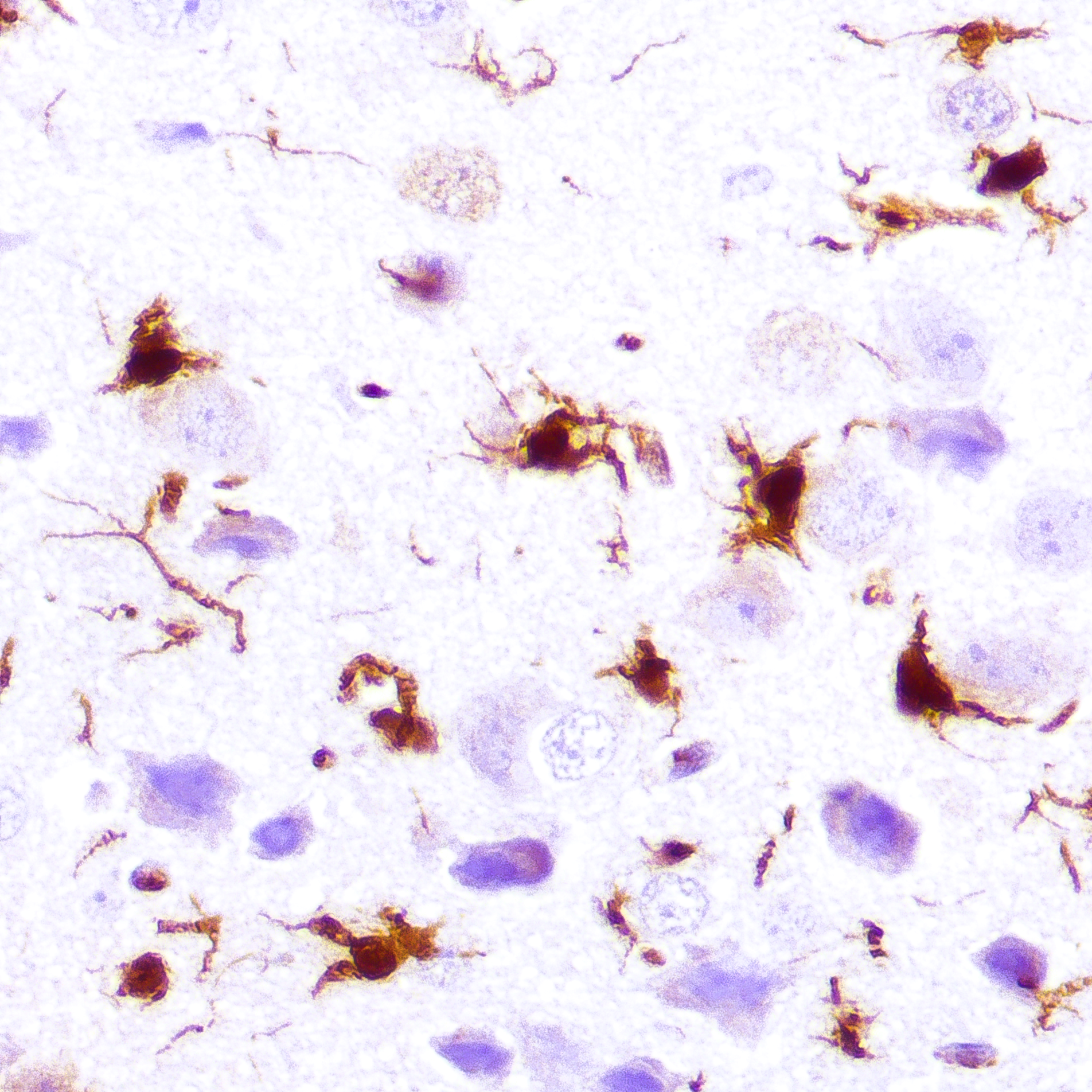

Supplement: Supplementary file 14 — Figure EV3 Source Data [file 44319_2026_721_MOESM14_ESM.zip › Figure EV3/EV3A/KO/Ctx_Layer I.tif]

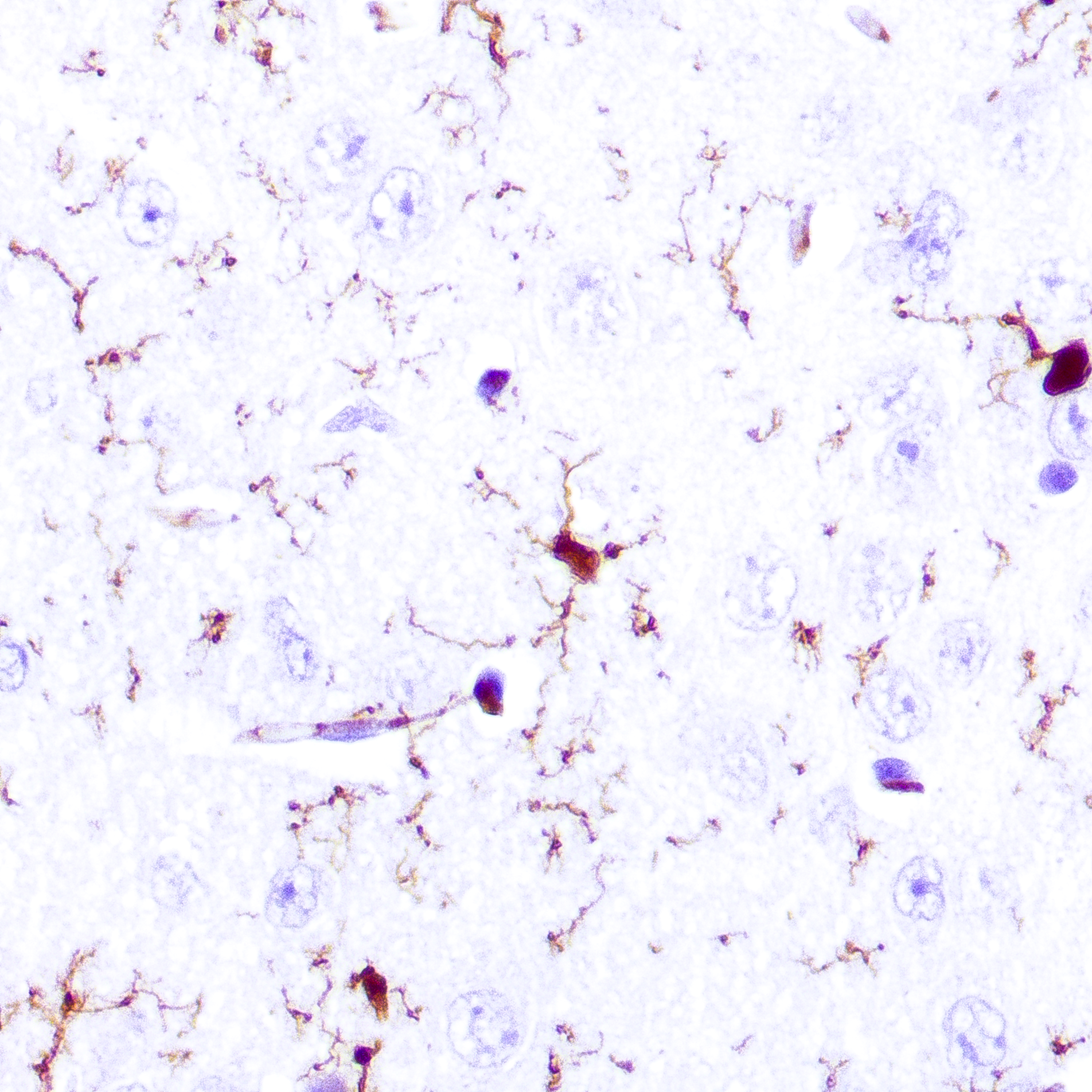

Supplement: Supplementary file 14 — Figure EV3 Source Data [file 44319_2026_721_MOESM14_ESM.zip › Figure EV3/EV3A/Control/Striatum.tif]

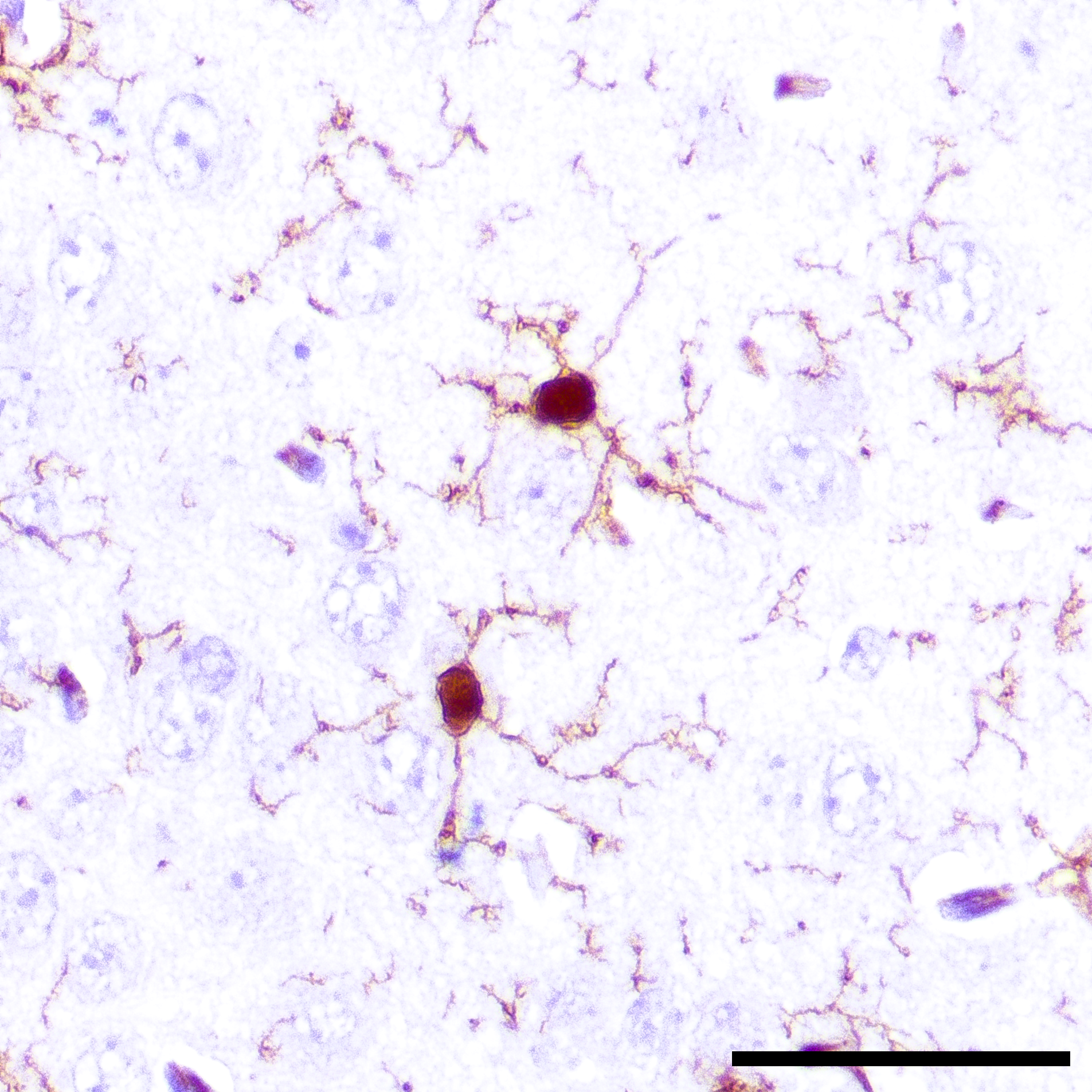

Supplement: Supplementary file 14 — Figure EV3 Source Data [file 44319_2026_721_MOESM14_ESM.zip › Figure EV3/EV3A/Control/Ctx_LayerV-VI_scalebar.tif]

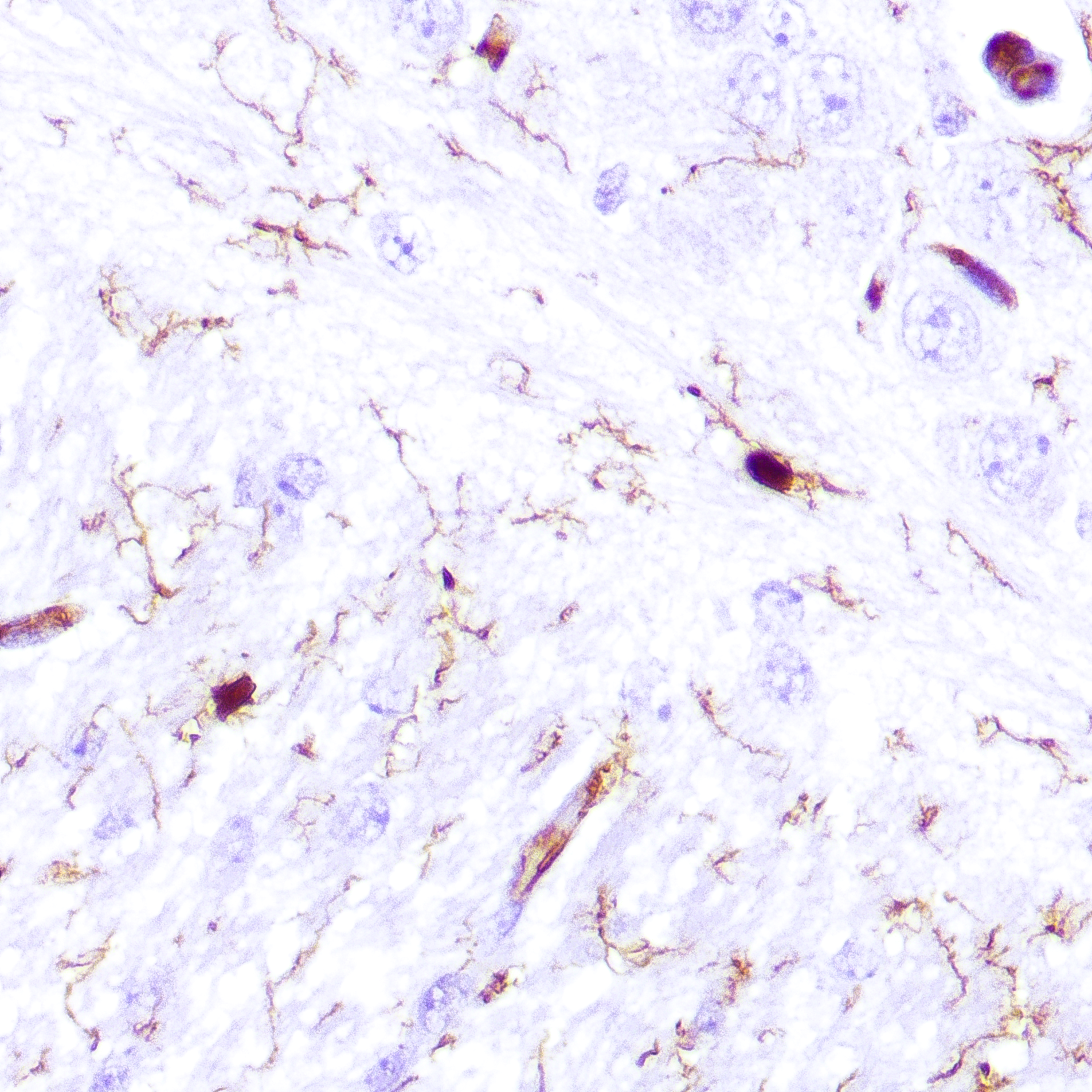

Supplement: Supplementary file 14 — Figure EV3 Source Data [file 44319_2026_721_MOESM14_ESM.zip › Figure EV3/EV3A/Control/CC.tif]

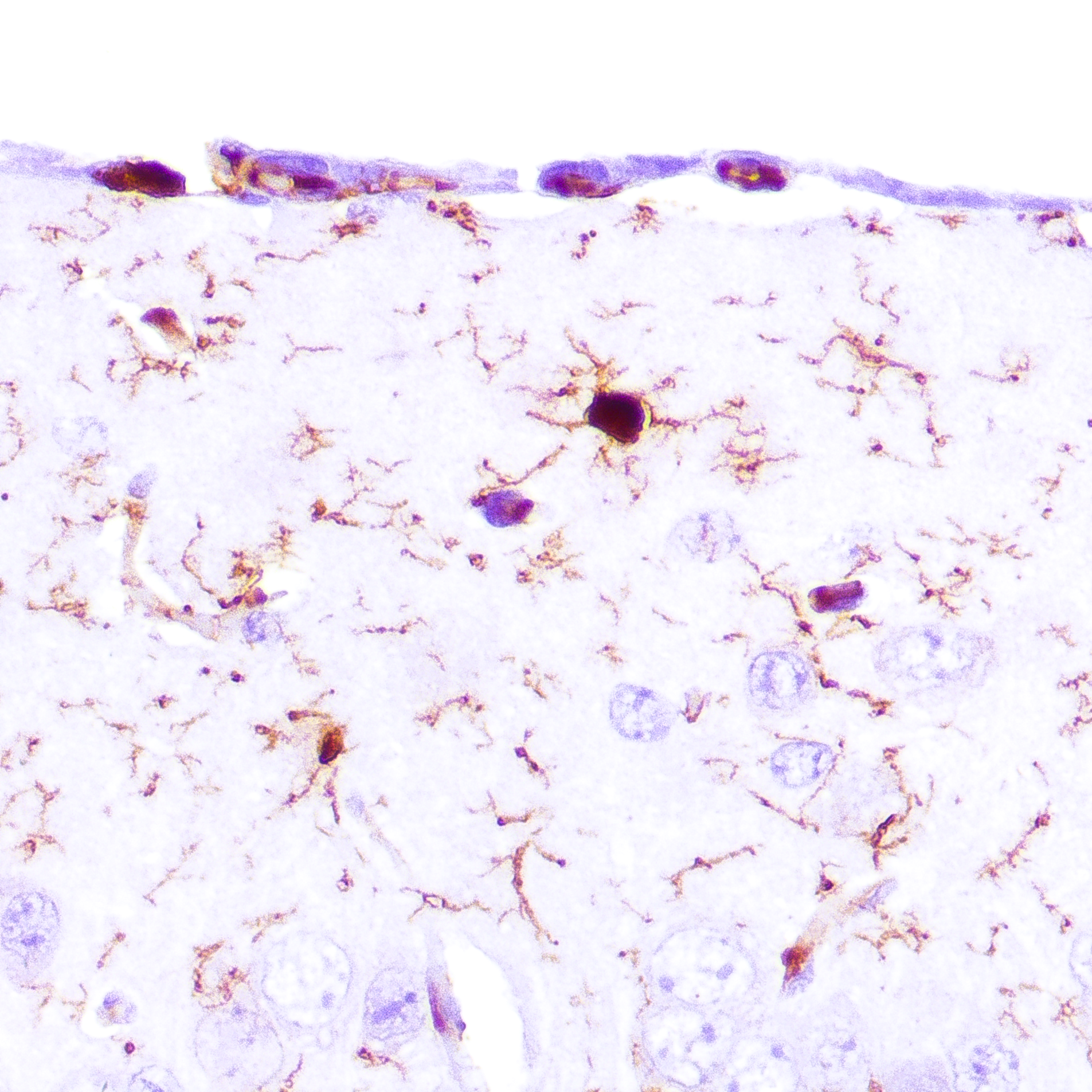

Supplement: Supplementary file 14 — Figure EV3 Source Data [file 44319_2026_721_MOESM14_ESM.zip › Figure EV3/EV3A/Control/Ctx_LayerI.tif]

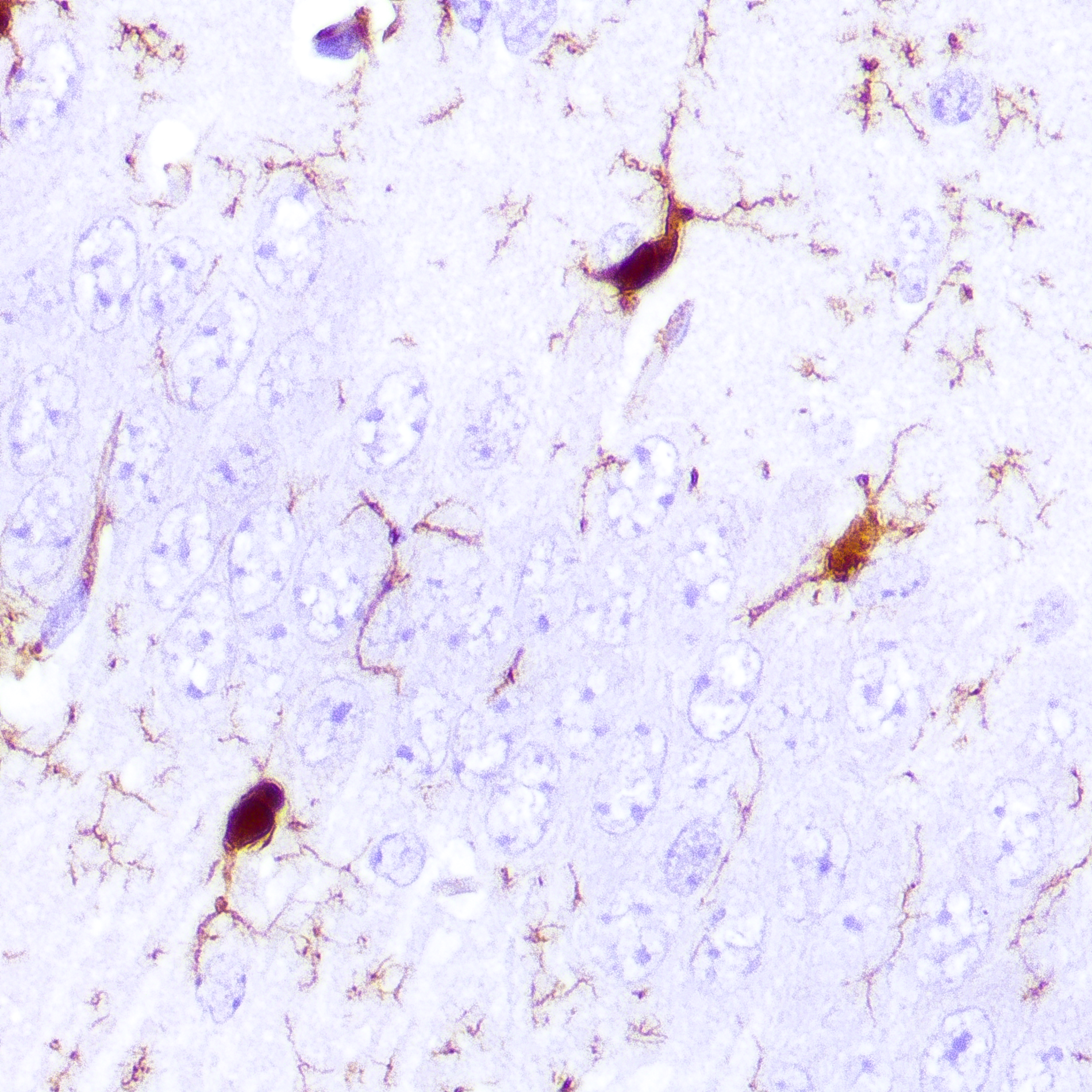

Supplement: Supplementary file 14 — Figure EV3 Source Data [file 44319_2026_721_MOESM14_ESM.zip › Figure EV3/EV3A/Control/hippocampus.tif]

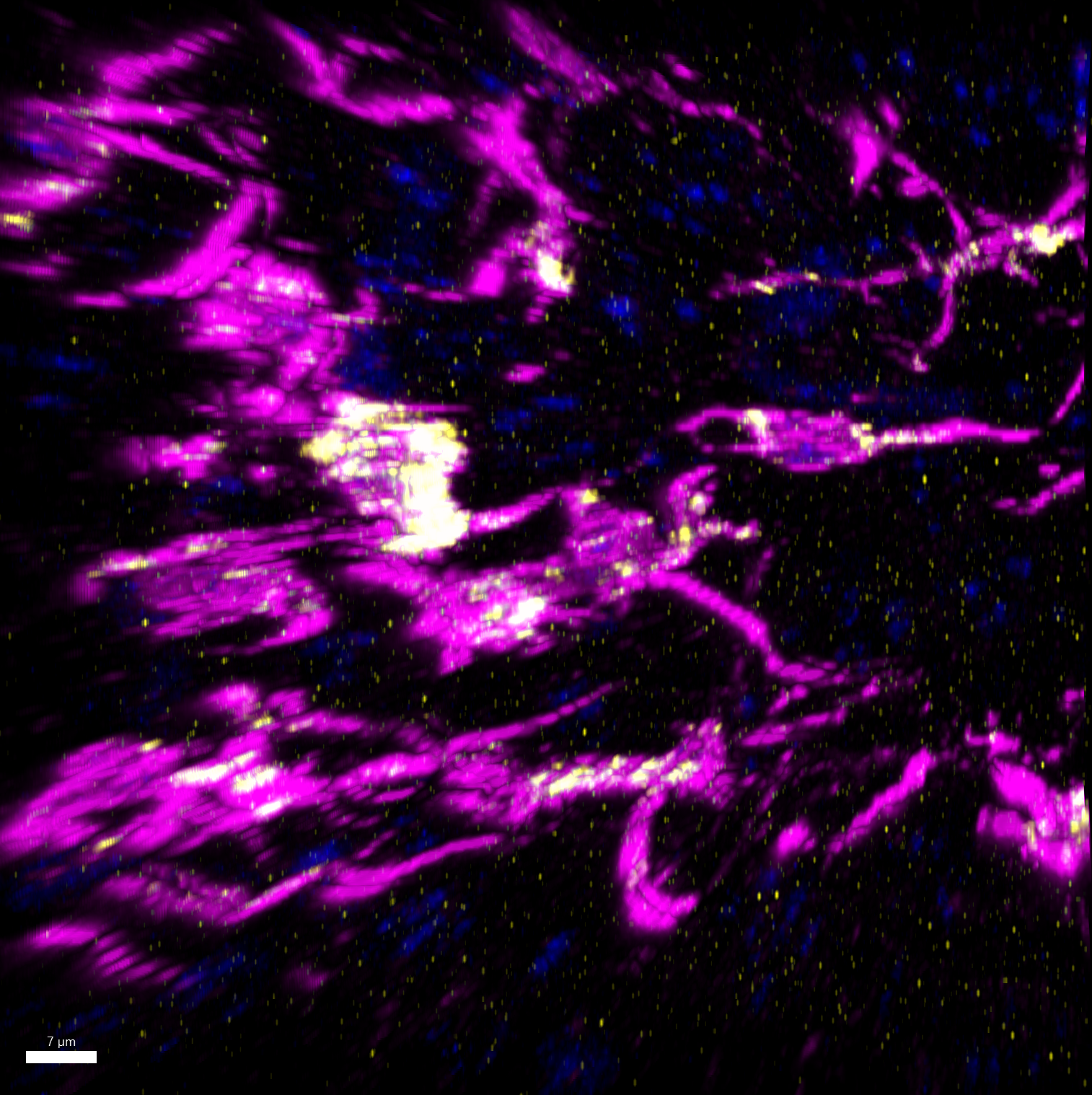

Supplement: Supplementary file 14 — Figure EV3 Source Data [file 44319_2026_721_MOESM14_ESM.zip › Figure EV3/EV3E/KO/CD68-IBA1.tif]

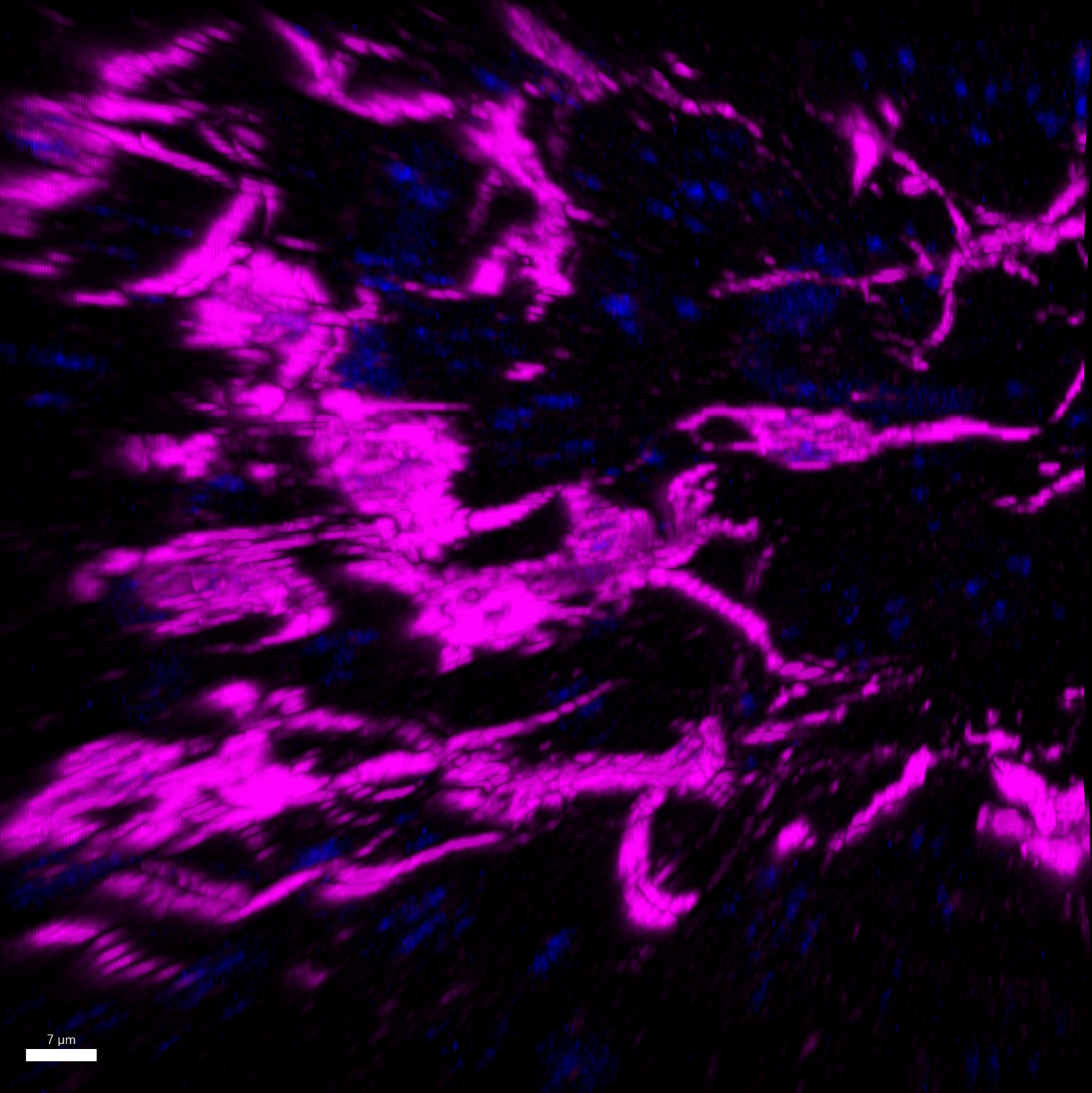

Supplement: Supplementary file 14 — Figure EV3 Source Data [file 44319_2026_721_MOESM14_ESM.zip › Figure EV3/EV3E/KO/IBA1.tif]

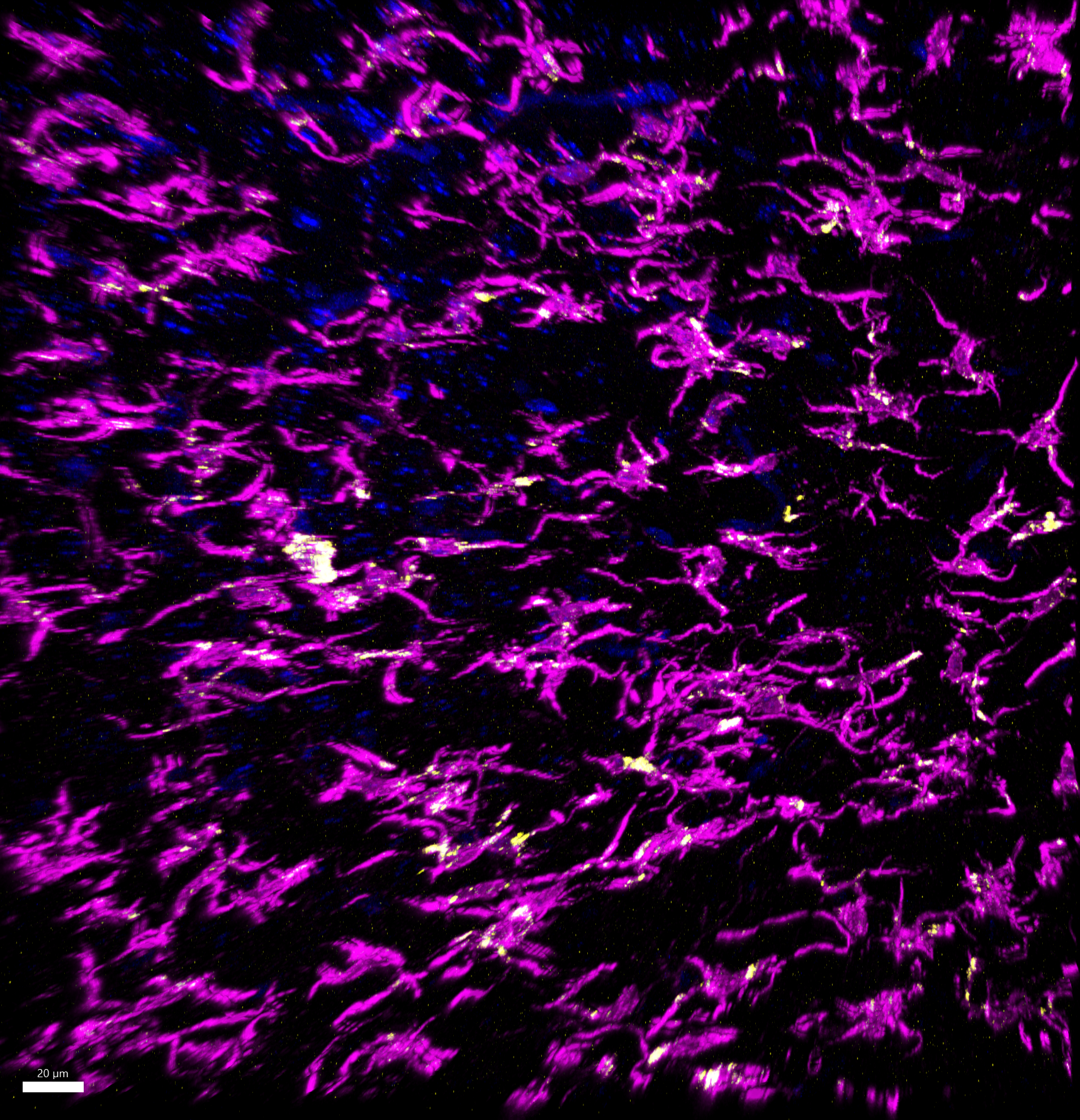

Supplement: Supplementary file 14 — Figure EV3 Source Data [file 44319_2026_721_MOESM14_ESM.zip › Figure EV3/EV3E/KO/overview.tif]

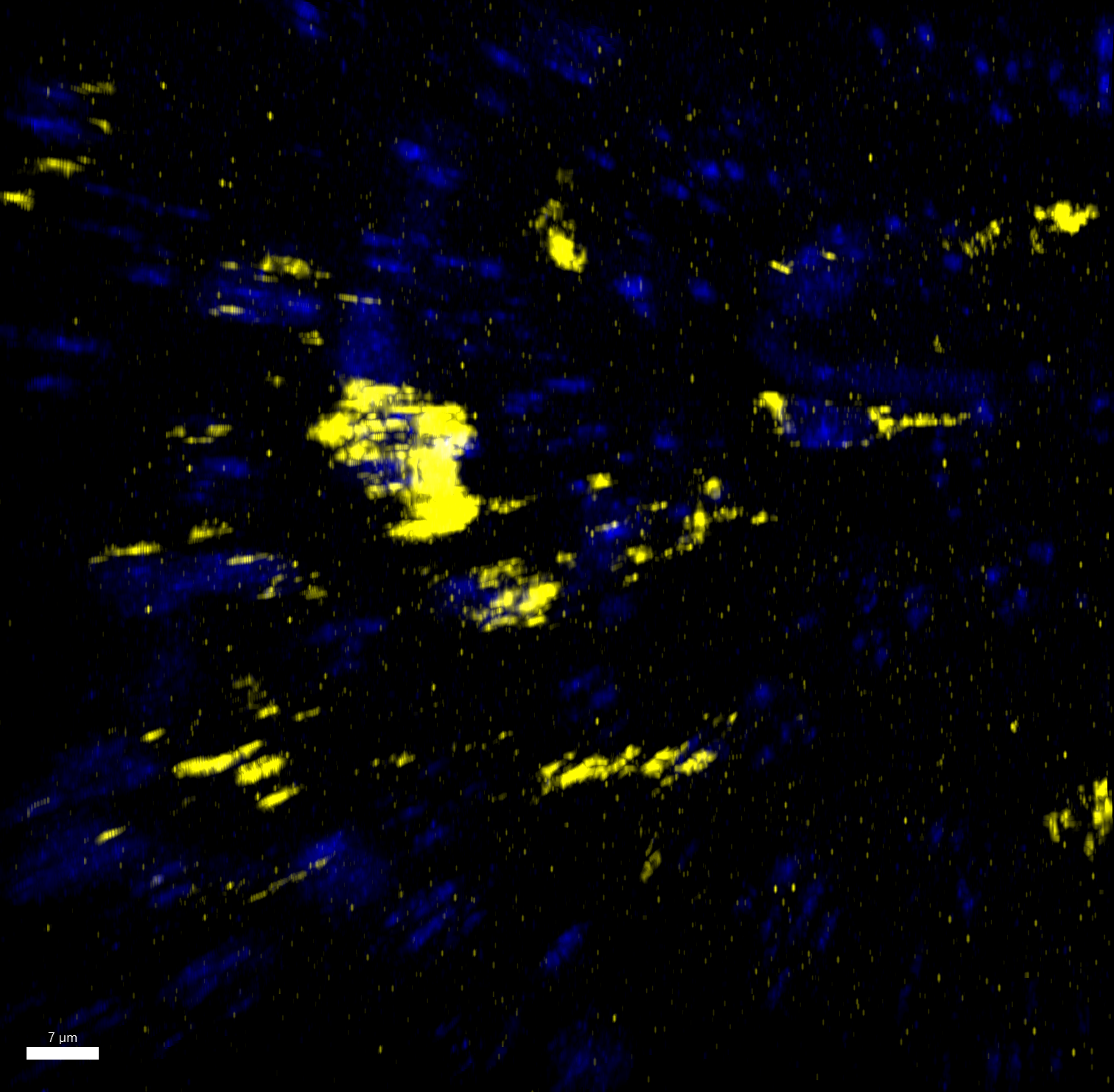

Supplement: Supplementary file 14 — Figure EV3 Source Data [file 44319_2026_721_MOESM14_ESM.zip › Figure EV3/EV3E/KO/CD68.tif]

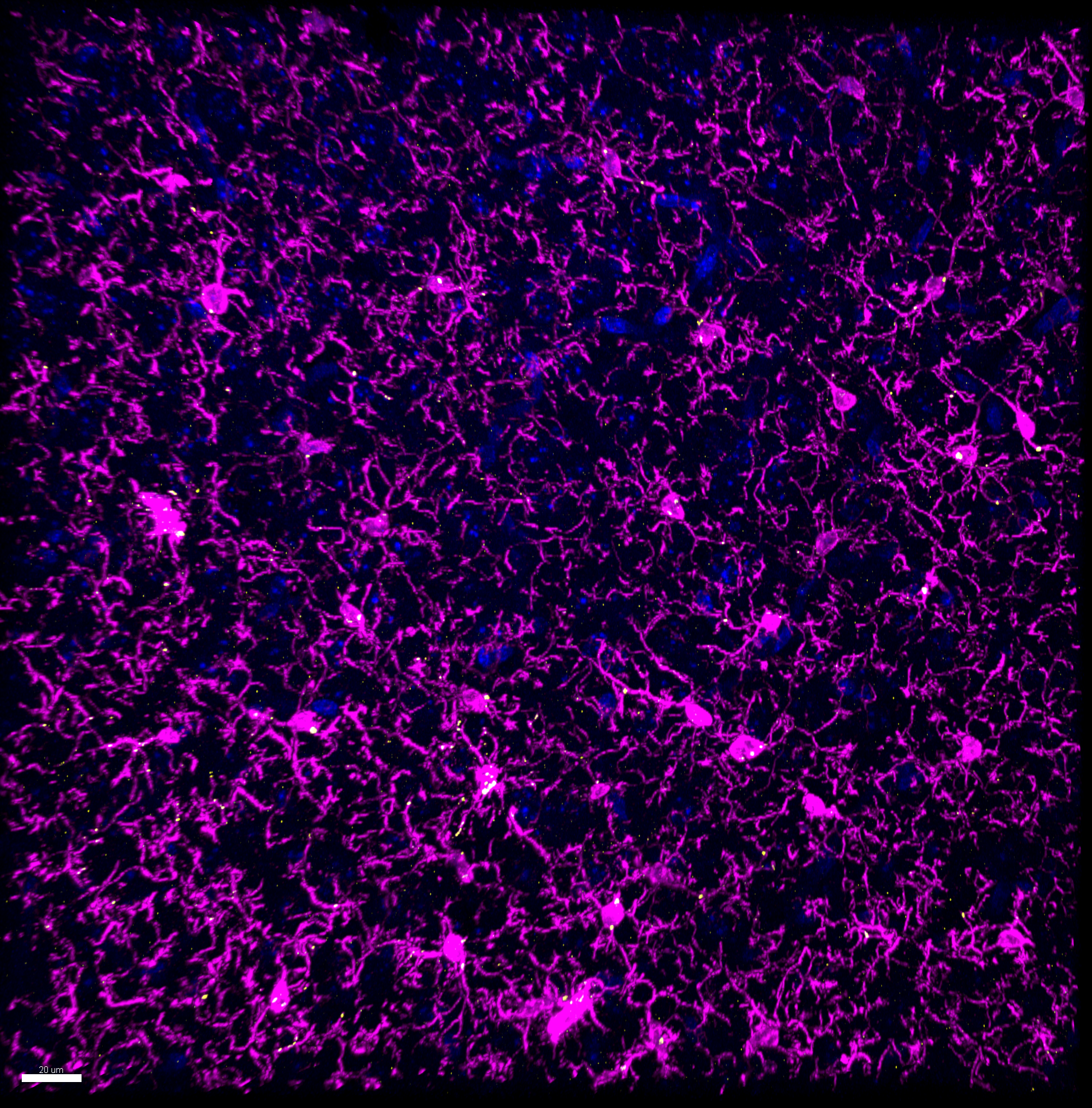

Supplement: Supplementary file 14 — Figure EV3 Source Data [file 44319_2026_721_MOESM14_ESM.zip › Figure EV3/EV3E/Control/overview.tif]

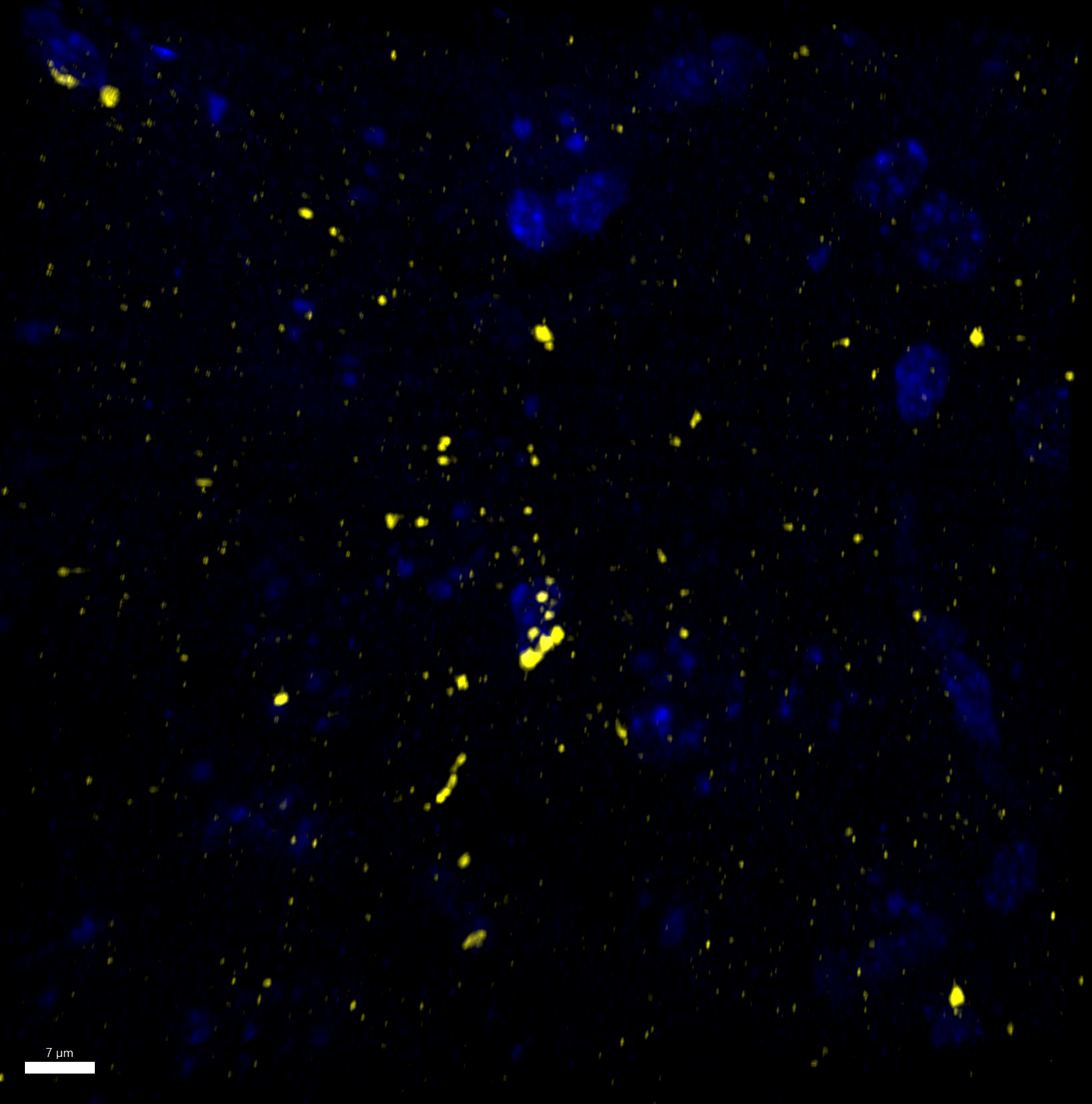

Supplement: Supplementary file 14 — Figure EV3 Source Data [file 44319_2026_721_MOESM14_ESM.zip › Figure EV3/EV3E/Control/CD68-DAPI.tif]

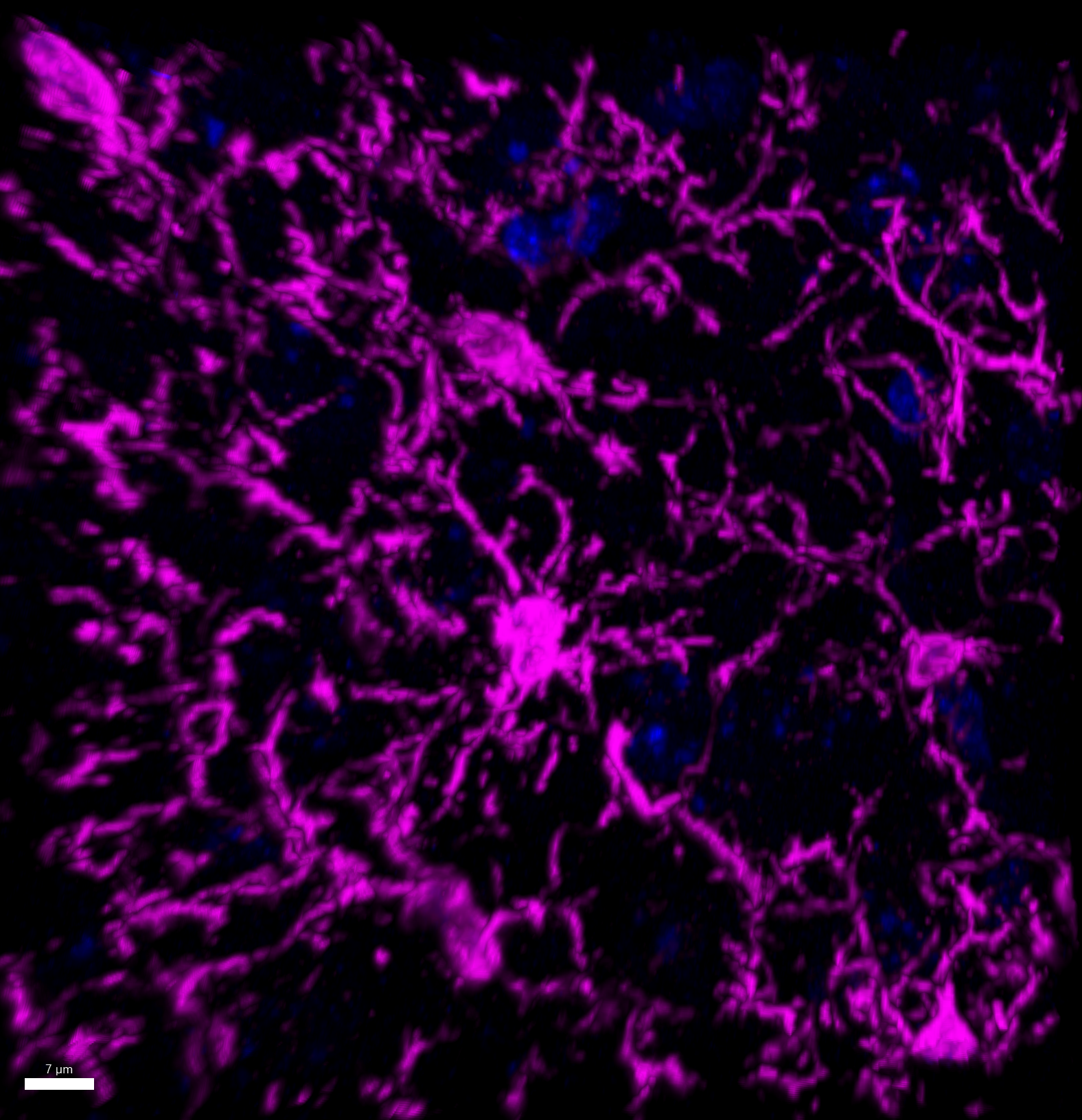

Supplement: Supplementary file 14 — Figure EV3 Source Data [file 44319_2026_721_MOESM14_ESM.zip › Figure EV3/EV3E/Control/IBA1tif.tif]

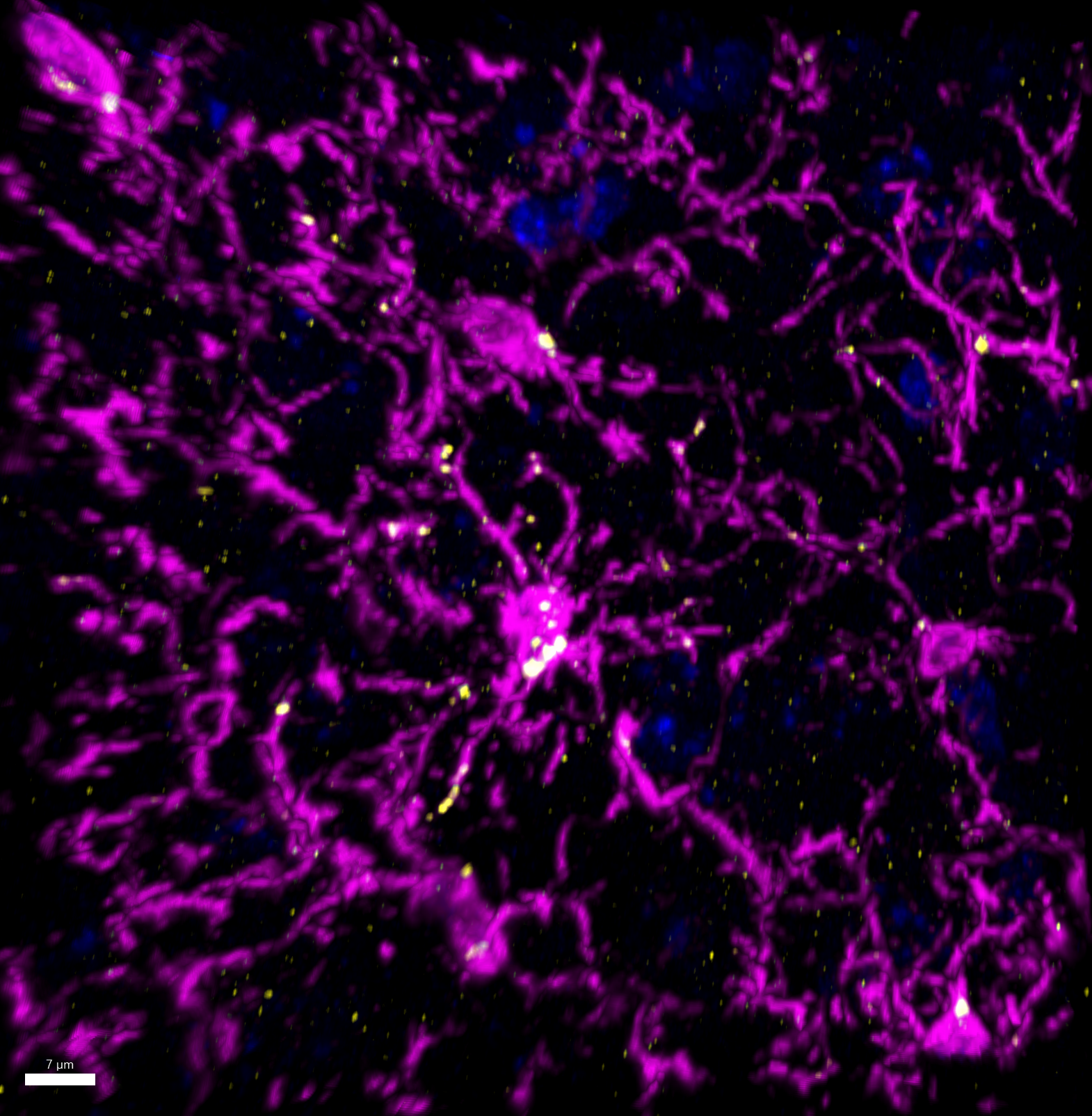

Supplement: Supplementary file 14 — Figure EV3 Source Data [file 44319_2026_721_MOESM14_ESM.zip › Figure EV3/EV3E/Control/CD68-IBA1tif.tif]
