# Supplementary material for: The Arp2/3 complex controls the development of homeostatic microglia
Source: EMBO Rep. 2026 Feb 27;27(7):1696–719. doi: 10.1038/s44319-026-00721-8 (PMC13076794; doi:10.1038/s44319-026-00721-8)
Supplement: Supplementary file 15 — Expanded View Figures [file 44319_2026_721_MOESM15_ESM.pdf]

Expanded View Figures

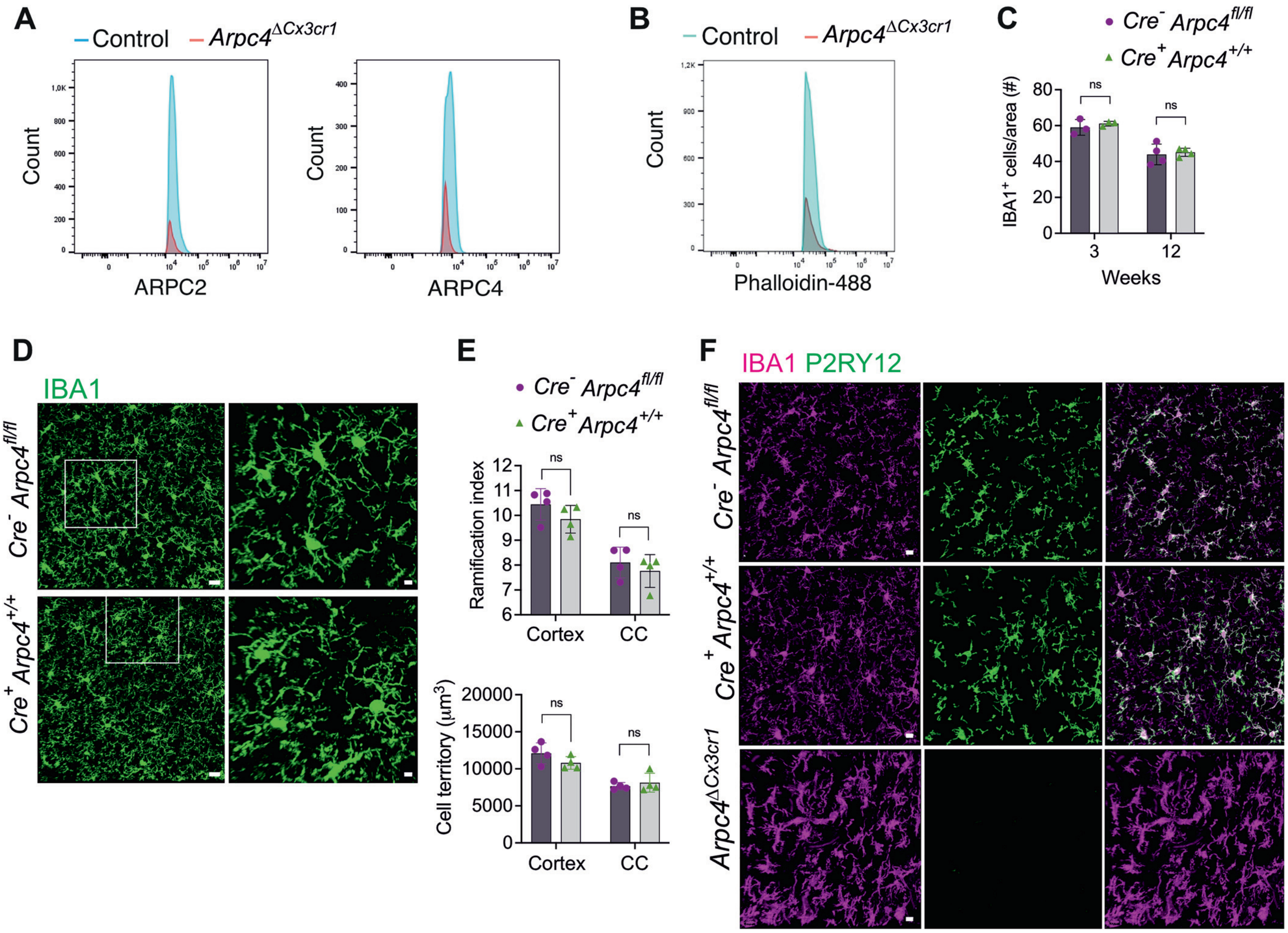

Figure EV1. Microglia characteristics in *Tg(Cx3cr1-Cre)*-expressing and *Tg(Cx3cr1-Cre)*-negative $Arpc4^{+/+}$ mice.

(**A**) Flow cytometric analysis of ARPC2 and ARPC4 expression in 12-week-old *Tg(Cx3cr1-Cre)* $Arpc4^{fl/fl}$ ($Arpc4^{\Delta Cx3cr1}$) and $Arpc4^{fl/fl}$ control mice (Biological replicates: $n = 4$ mice per group, Technical replicates: $n = 3$ experiments). (**B**) Flow cytometric analysis of Alexa Fluor 488-conjugated phalloidin, indicating global levels of F-actin in microglia from 12-week-old $Arpc4^{\Delta Cx3cr1}$ and control mice (Biological replicates: $n = 4$ mice per group, Technical replicates: $n = 2$ experiments). (**C**) Quantification of microglial cell number in the cortex of 3- and 12-week-old Cre^{+} $Arpc4^{+/+}$ and Cre^{-} $Arpc4^{fl/fl}$ mice ($n = 3$–4 mice per group, data were presented as mean ± s.d, two-tailed Student's *t*-test, ns: $P > 0.9999$). (**D**, **E**) Representative images of $IBA1^{+}$ microglia morphology and quantification of microglia ramification and cell territory in 12-week-old *Tg(Cx3cr1-Cre)* $Arpc4^{+/+}$ (Cre^{+} $Arpc4^{+/+}$) and Cre-negative $Arpc4^{fl/fl}$ (Cre^{-} $Arpc4^{fl/fl}$) mice. Scale bar: 20 µm (left) and 5 µm (right) ($n = 4$ mice per group, data are presented as mean ± s.d., two-tailed Student's *t*-test, ns not significant). (**F**) Representative images showing $IBA1^{+}$/$P2RY12^{+}$ microglia in 12-weeks-old Cre^{+} $Arpc4^{+/+}$, Cre^{-} $Arpc4^{fl/fl}$ and *Tg(Cx3cr1-Cre)* $Arpc4^{fl/fl}$ ($Arpc4^{\Delta Cx3cr1}$) mice, scale bar: 20 µm. Source data are available online for this figure.

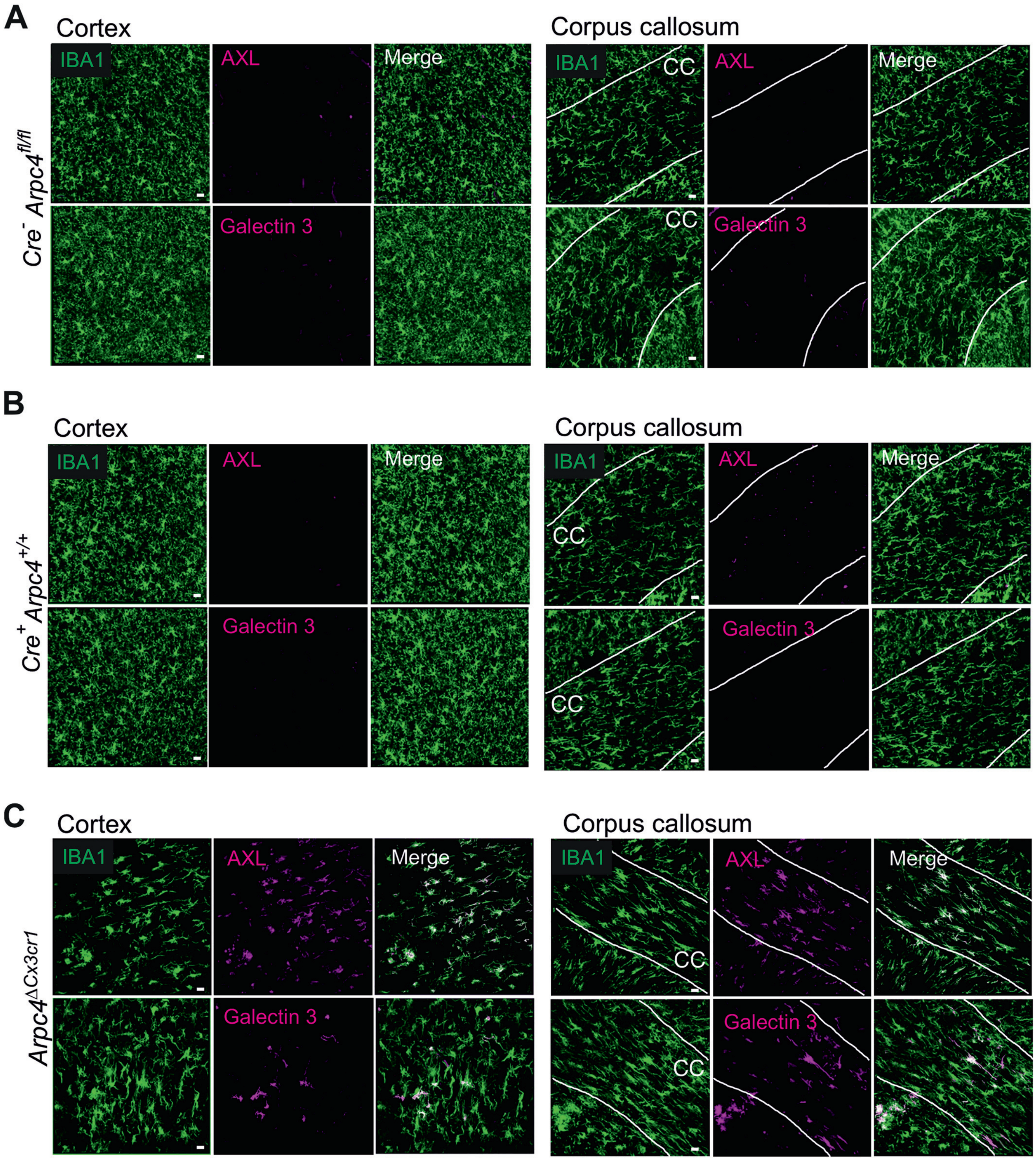

Figure EV2. Microglial activation in Cre^{+} *Arpc4*$^{+/+}$ compared to the control (Cre^{-} *Arpc4*$^{fl/fl}$) and *Arpc4*$^{\Delta Cx3cr1}$ mice.

Representative images of AXL and Galectin-3 expression in IBA1^{+} microglia in the cortex and corpus callosum in 12-weeks-old *Cre-negative Arpc4*$^{fl/fl}$ *(Cre*$^{-}$ *Arpc4*$^{fl/fl}$) (**A**), *Tg(Cx3cr1-Cre) Arpc4*$^{+/+}$ (Cre^{+} *Arpc4*$^{+/+}$) (**B**), *and Tg(Cx3cr1-Cre) Arpc4*$^{+/+}$ (*Arpc4*$^{\Delta Cx3cr1}$) (**C**) mice. Scale bar: 20 µm. Confocal microscopy overview images come from stitched tile scans of immunostained brain tissues. Source data are available online for this figure.

 © The Author(s)

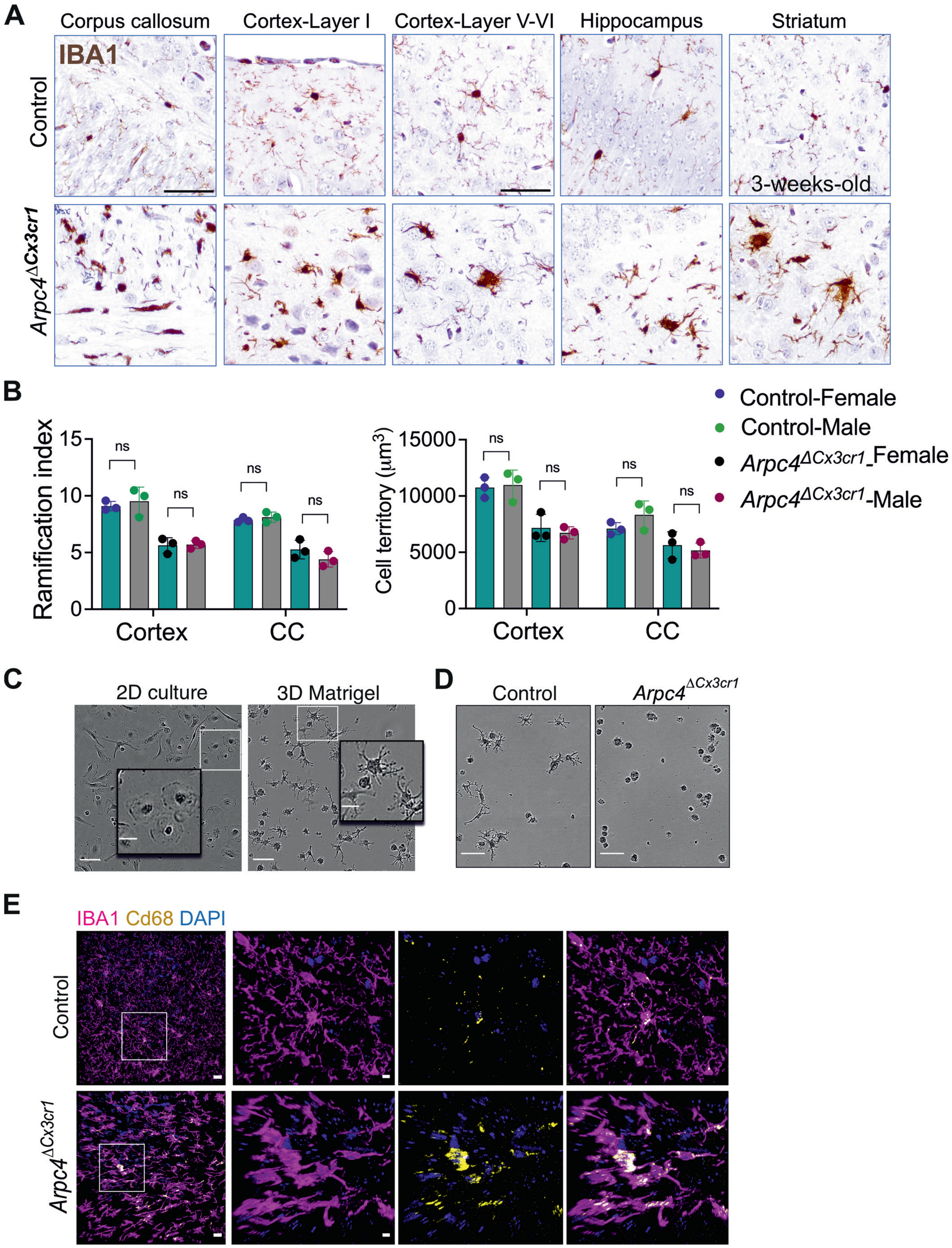
A
Corpus callosum
Cortex-Layer I
Cortex-Layer V-VI
Hippocampus
Striatum
IBA1
Control
Arpc4ΔCx3cr1
3-weeks-old
B
Ramification index
Cell territory (μm3)
ns
Cortex
CC
Control-Female
Control-Male
Arpc4ΔCx3cr1_Female
Arpc4ΔCx3cr1-Male
C
2D culture
3D Matrigel
D
Control
Arpc4ΔCx3cr1
E
IBA1 Cd68 DAPI
Control
Arpc4ΔCx3cr1

Figure EV3. Microglial morphology upon *Arpc4* depletion in vivo and in vitro.

(**A**) Representative DAB-stained images of microglial morphology in different brain regions in 3-week-old *Arpc4*$^{\Delta Cx3cr1}$ and control mice. Scale bar: 100 µm. (**B**) Quantification of microglia ramification and cell territory in 12-week-old female and male *Arpc4*$^{fl/fl}$ (control) and *Tg(Cx3cr1-Cre) Arpc4*$^{fl/fl}$ (*Arpc4*$^{\Delta Cx3cr1}$) mice ($n = 3$ mice per group, data are presented as mean ± s.d., two-tailed Student's *t*-test, ns: $P > 0.9999$). (**C**) Comparison of wild-type microglia morphology in 2D and Matrigel 3D culture systems. Scale bar: 50 µm. (**D**) *Arpc4*$^{\Delta Cx3cr1}$ and control microglia in the Matrigel 3D culture after 10 h. Scale bar: 50 µm. (**E**) Representative images of CD68 immunolabeling of IBA1^{+} microglia in 3-week-old *Arpc4*$^{\Delta Cx3cr1}$ and control mice. Scale bar: 20 µm (overview), 7 µm (zoom-in). Source data are available online for this figure.

© The Author(s)

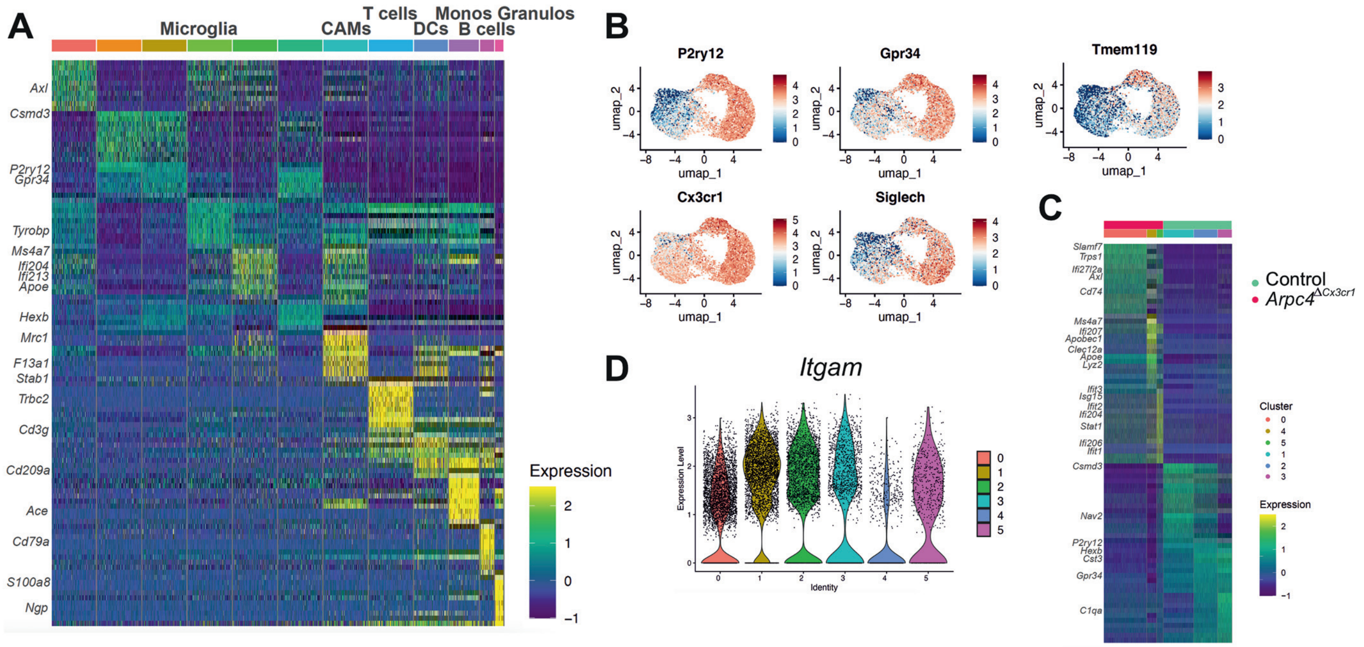

Figure EV4. Downregulation of TGFβ-dependent microglial homeostatic signature, their reactive phenotype in *$Arpc4^{\Delta Cx3cr1}$* mice.

(**A**) Heat map of genes (rows) of all the cell types that are highlighted in Fig. 3A, B. Key genes are highlighted. Colors in the heat map correspond to normalized, scaled expression. (**B**) Expression levels of microglial homeostatic genes, *P2ry12, Gpr34, Cx3cr1, Siglech,* and *Tmem119* in microglia clusters. (**C**) Heat map of genes (rows) of microglia clusters that are highlighted in Fig. 3C. Key genes are highlighted. Colors in the heat map correspond to normalized, scaled expression. (**D**) Violin plots showing the expression level of *Itgam* in the microglial clusters in *$Arpc4^{\Delta Cx3cr1}$* and control mice ($n = 4$ control and 3 *$Arpc4^{\Delta Cx3cr1}$* mice).

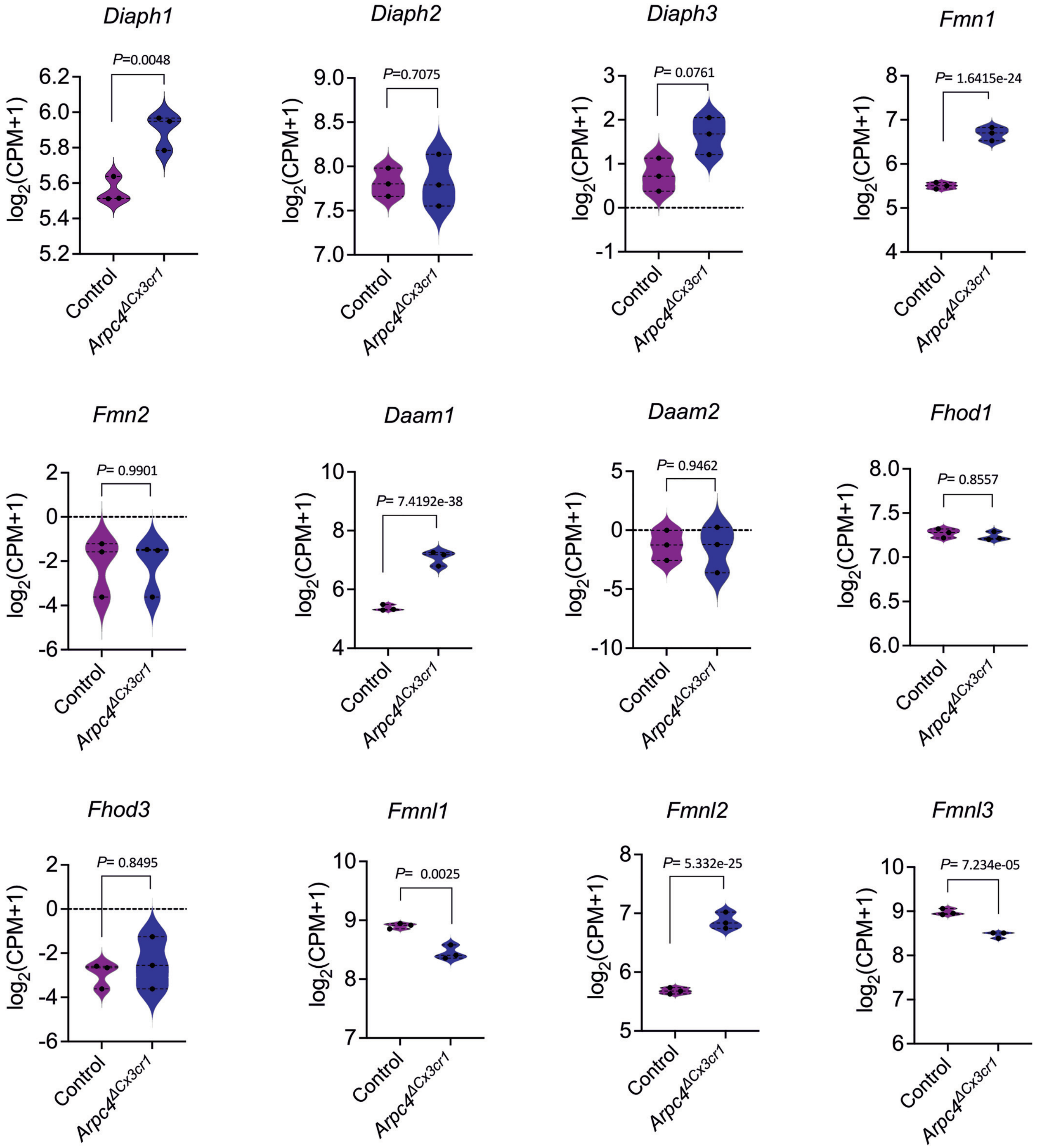

Figure EV5. Expression levels of formin/diaphanous family of proteins.

Violin plots showing gene expression levels of formin/diaphanous family of proteins in microglia isolated from *Arpc4*$^{\Delta Cx3cr1}$ and control mice ($n = 4$ control and 3 *Arpc4*$^{\Delta Cx3cr1}$ mice). Data were retrieved from bulk RNA-seq data sets from CNS-isolated microglia, analyzed in Fig. 3C–F. CPM counts per million.

 © The Author(s)
